# Transition-Metal-Catalyzed C–H Bond Activation for the Formation of C–C Bonds in Complex Molecules

**DOI:** 10.1021/acs.chemrev.2c00888

**Published:** 2023-05-10

**Authors:** Jamie H. Docherty, Thomas M. Lister, Gillian Mcarthur, Michael T. Findlay, Pablo Domingo-Legarda, Jacob Kenyon, Shweta Choudhary, Igor Larrosa

**Affiliations:** School of Chemistry, University of Manchester, Oxford Road, Manchester M13 9PL, United Kingdom

## Abstract

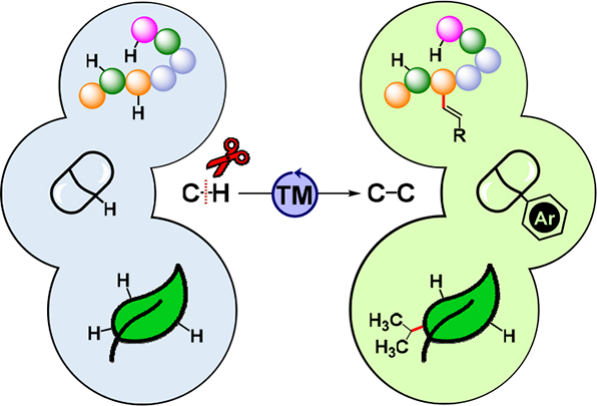

Site-predictable and chemoselective C–H bond functionalization reactions offer synthetically powerful strategies for the step-economic diversification of both feedstock and fine chemicals. Many transition-metal-catalyzed methods have emerged for the selective activation and functionalization of C–H bonds. However, challenges of regio- and chemoselectivity have emerged with application to highly complex molecules bearing significant functional group density and diversity. As molecular complexity increases within molecular structures the risks of catalyst intolerance and limited applicability grow with the number of functional groups and potentially Lewis basic heteroatoms. Given the abundance of C–H bonds within highly complex and already diversified molecules such as pharmaceuticals, natural products, and materials, design and selection of reaction conditions and tolerant catalysts has proved critical for successful direct functionalization. As such, innovations within transition-metal-catalyzed C–H bond functionalization for the direct formation of carbon–carbon bonds have been discovered and developed to overcome these challenges and limitations. This review highlights progress made for the direct metal-catalyzed C–C bond forming reactions including alkylation, methylation, arylation, and olefination of C–H bonds within complex targets.

## 1.0 Introduction

Chemical synthesis underpins the evolution and advancement of broad areas of science from materials to medicines.^1−6^ Transition-metal-catalyzed synthetic transformations offer broadly applicable strategies for the discovery and development of new and useful molecules that have served in diverse applications.^7−11^ However, the typical use of modern transition-metal-catalyzed synthetic methods has relied on the presence of prefunctionalized starting materials to control both chemo- and regio-selectivity.^12−15^ This has presented an inherent challenge given that the pool of prefunctionalized precursors is significantly smaller than that of unfunctionalized feedstock chemicals.^16,17^ Additionally, the requirement of prefunctionalization before a specific synthetic transformation is enabled limits applicability to the vast selection of already complex and heavily diversified molecules such as natural products or pharmaceuticals. To address this limitation a broad range of methods for the installation of synthetically useful functional handles have been developed, many of which rely on multistep procedures and conjointly lead to limited overall sustainability due to reduced step-economy, synthetic inefficiency, and waste.^18−25^

Therefore, the opportunity has arisen for the development of new and efficient catalytic methods that allow for the direct functionalization of typically inert C–H bonds. As highlighted by Goldberg and Goldman, the C–H bond is commonly considered inert to direct functionalization and therefore termed as an “unfunctional group” and this view is representative of the challenge and effort required to successfully functionalize C–H bonds.^26^ However, this opportunity and challenge has been widely engaged and has led to the development of a range of synthetic methods that are capable of direct C–H bond functionalization with a broad selection of coupling partners.^27−33^ The ability for the direct synthetic transformation of C–H bonds allows for improved step-economy and overall enhanced sustainability. New strategic disconnections and innovative synthetic routes can also be enabled when the C–H bond can be considered as a useful synthetic handle. However, many of the developed methods have been limited to relatively simple substrates bearing only few functional groups or with limited overall molecular complexity.

Molecular complexity is a fundamental intrinsic property of a chemical structure and provides a proxy for the synthetic effort and energy required to produce a given structure.^34−37^ However, the term has yet to be unambiguously defined despite decades of discussion within the scientific literature. Several efforts have been made to calculate or determine numerical values as a descriptor for the complexity of any given structure with no broad consensus.^38−41^ Principally, molecular complexity is likely a function of the number of rings, fraction sp^3^ carbons [F(sp^3^)], number of heteroatoms, and molecular weight of a structure. These factors are important to consider in the context of drug-discovery and are therefore relevant to the development of new synthetic platforms for molecular diversification.

Late-stage functionalization (LSF) has emerged as a useful descriptor for reactions that take place on intuitively complex molecular frameworks. As defined by Ritter: “*LSF is a desired chemoselective transformation on a complex molecule to provide at least one analog in sufficient quantity and purity for a given purpose without the necessity for installation of a functional group that exclusively serves the purpose to enable said transformation*”.^31^ This definition succinctly encapsulates the ideality for synthetic procedures to have excellent levels of chemoselectivity without the requirement of preintroduction of additional specific functional groups to control selectivity. While optimal for efficiency, these goals as defined exclude any procedure whereby a temporary (removable) directing group is required. Additionally, LSF precludes the inclusion of examples whereby a complex fragment is strategically prefunctionalized for diversification with a subsequent catalogue of reaction partners.

### 1.1 C–H Bond Functionalization Challenges

**Scheme 1:**
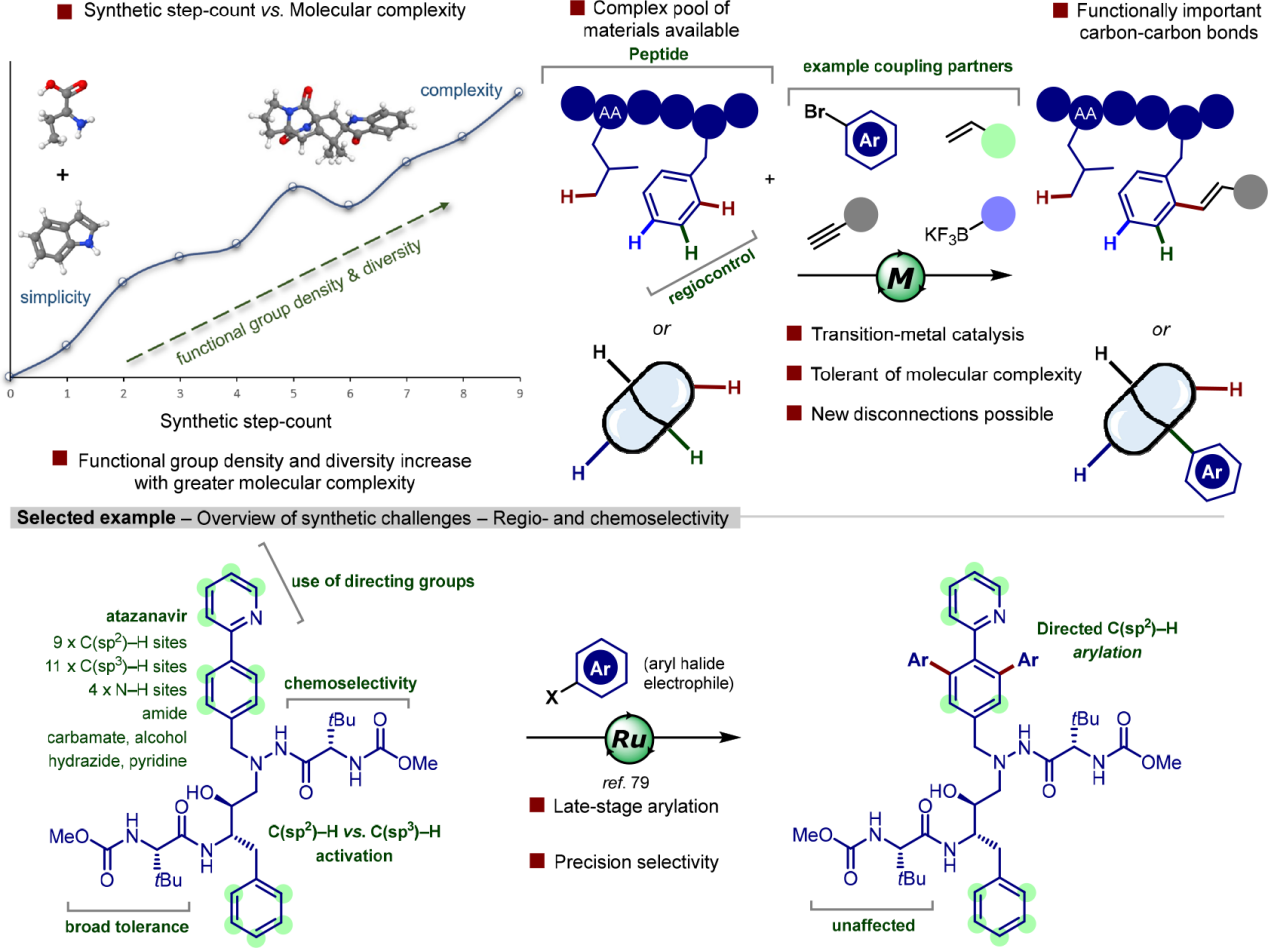
Challenges Associated with Functionalization of Targets Bearing Increasing Levels of Molecular Complexity: Direct C–H Bond Functionalization Provides a Powerful Synthetic Strategy for Rapid Diversification

The relative paucity of methods for the direct transition-metal-catalyzed C–H bond functionalization of complex molecules is likely a reflection of the challenge encountered when new catalytic methods are applied to high-complexity environments. Specifically, as molecular complexity increases, the number of functional groups and number of available C–H bonds within a molecule typically increase (Scheme 1). Both scenarios raise unique challenges and considerations. For example, as the number of functional groups increase, the risks of inhibitory and unproductive catalyst coordination by Lewis-basic functional groups increases. Furthermore, as the functional group density and diversity increase with increasing molecular complexity, the prospect of catalyst intolerance of a specific functional group within the target molecule may limit applicability.^42^ If the selected substrate for functionalization possesses units with strongly coordinating groups these can compete for catalyst binding, and either partially or completely inhibit the desired reactivity by competitive catalyst coordination or complete sequestration.^43−52^ Robustness screening of a wide selection of additive reagents added into optimized reaction procedures using simple substrates has allowed for elucidation of inhibitory and intolerable functional groups within established procedures and thus has served as a guide before the application to high-value complex molecules is attempted.^53^

### 1.2 Directing Group-Based C–H Bond Activation Strategies

The typically large availability of C–H bonds within highly complex molecules introduces challenges of regioselectivity. This has raised the important question: *How can the synthetic chemist target specific and individual C–H bonds within a molecule that contains many potentially reactive C–H bonds*? The reliable and site-predictable functionalization of high complexity substrates represents a major challenge within synthetic chemistry. Directing groups have emerged to orient catalyst species toward specific C–H sites.^54−56^ Generally, Lewis-basic groups have proven effective at catalyst coordination and therefore C–H bonds proximal to the Lewis-base functionality have provided access to highly site-selective C–H bond functionalization methods. Synthetic transformations reliant on directing groups for C–H bond activation have used a broad spectrum of functional groups to perform this role. These have included both strong coordinating groups (e.g., pyridine, oxazoline, imine) and comparatively weaker directing groups, for example: ketones, carbamates, carboxylic acids, aldehydes, and ethers.^57−60^ Correspondingly, such directing groups have been established for the selective *ortho-*, *meta-*, and *para*-functionalization of arenes and for both proximal and distal C(sp^3^)–H bond functionalization.^61−65^ In the context of complex molecule C–H bond functionalization, diverse directing-group compatibility offers the prospect of general applicability of many developed procedures and catalytic manifolds in the widest chemical settings.

Although directing group strategies have broadly emerged with high levels of regiocontrol, many natural products, pharmaceuticals and complex molecules do not inherently possess the necessary directing groups for strategic synthetic manipulation using established metal-catalyzed methods. Therefore, C–H bond functionalization of molecules devoid of directing groups raises difficult challenges with respect to regioselectivity.^66−68^ This can be prohibitive in the context of complex molecules that contain several potentially reactive C–H bond sites and has required innovative solutions to address this challenge.

Taken together, these challenges and considerations highlight the need for the synthetic community to discover and develop general catalytic methods that are both tolerant of molecular diversity and that are both chemo- and regioselective in the presence of diverse functional groups and many potentially reactive C–H bonds.

### 1.3 Applications of C–H Functionalization in Complex Molecules

The synthetic ability for site-predictable and robust diversification of individual C–H bonds within a complex environment allows for the step-economic syntheses of large compound libraries with minimal synthetic effort and significant time savings.^69−71^ Similarly, if reacted with highly modular and composable coupling partners this can allow for efficient combinatorial approaches in structure activity relationship and chemical space exploration.^72,73^ Incorporation of small discrete molecular changes by C–H functionalization reactions can additionally enable adjustment of pharmacokinetic properties alongside physicochemical drug properties (e.g., potency, selectivity, solubility and stability).^74,75^ Emergence of innovations such as high-throughput experimentation and modern chemoinformatics provides synergistic and powerful opportunities with C–H functionalization reactions for the discovery and development of new molecules that are useful within biological contexts. Additionally, the broad tolerance and applicability of the state-of-the-art transition-metal-catalyzed C–H functionalization reactions demonstrate promise for future innovations within emerging technologies such as “on-DNA” chemical transformations and synthetic procedures within other biologically complex scenarios that will enable further innovation within medicinal applications and beyond.^76^

Synthetic applications of late-stage functionalization have been broadly reviewed from a medicinal chemistry perspective.^27−33^ This review provides an overview of the state-of-the-art transition-metal-catalyzed C–H bond functionalization methods that have proven to be applicable to complex molecules for the formation of a diverse range of carbon–carbon bonds. Examples discussed include LSF procedures, use of temporary directing groups, undirected C–H activation and strategies involving prefunctionalized complex molecules. A wide selection of C(sp^2^)–H bond and C(sp^3^)–H bond functionalizations are discussed, including alkylations, methylations, arylations, alkenylations, and alkynylations. Examples tolerant of a broad range of functional group diversity and those that retain high levels of regioselectivity have been highlighted.

## 2.0 C(sp^2^)–H Bond Functionalizations

### 2.1 Directed C–H Alkylation

The high bond dissociation enthalpy of C–H bonds and their ubiquitous nature in organic molecules present a challenge for their efficient functionalization. A common method that addresses these issues involves the use of a directing group, typically a weakly coordinating Lewis-basic group, which can coordinate to the transition-metal catalyst to guide reactivity to a specific C–H bond. Although template directing groups are known that can direct reactivity to more distal C–H bonds, typically the use of this method permits functionalization at the position *ortho-* to the directing group, or the closest C–H bond to the directing group substituent.

An early example of this type of reactivity was reported by Murai in 1993, with the *ortho-*alkylation of arylketone substrates using ruthenium catalysis.^77^ Since then, numerous reports of directed C–H activation have been published, utilizing a variety of transition-metals, directing groups and coupling partners, greatly expanding the range of chemistry that is possible with directed C–H functionalization. More recently, an increased focus has been placed on developing methodologies that tolerate sensitive functional groups and greater structural complexity, in order to utilize this chemistry for the late-stage functionalization and diversification of molecules. This section will discuss reports of directed C–H alkylation that involved examples of late-stage functionalization and diversification and are grouped by coupling partner.

C–H activation for the formation of C(sp^2^)–C(sp^3^) bonds has been underexplored in comparison to C(sp^2^)–C(sp^2^) bond formation, particularly for secondary alkyl halides. While advances in *ortho*-secondary alkylation had been reported with palladium, nickel, cobalt, manganese, and iron,^78^ the requirement for elevated temperatures, and superstoichiometric quantities of Grignard reagents with some metals, have limited the functional-group compatibility of these procedures. In addition, ruthenium-catalyzed procedures for secondary alkylations were limited to the formation of *meta*-alkylation products.

Work performed in our group on the ruthenium-catalyzed arylation of directing group containing arenes led us to identify the *para*-cymene ligand commonly present on ruthenium as an inhibitor of these reactions.^79^ Consequently, the development of new η^6^-arene-free monocyclometalated ruthenium catalysts then allowed C–H arylation to proceed under significantly milder temperatures than was previously possible, resulting in an increased functional group tolerance and enabling late-stage arylation using aryl halide coupling partners.

**Scheme 2:**
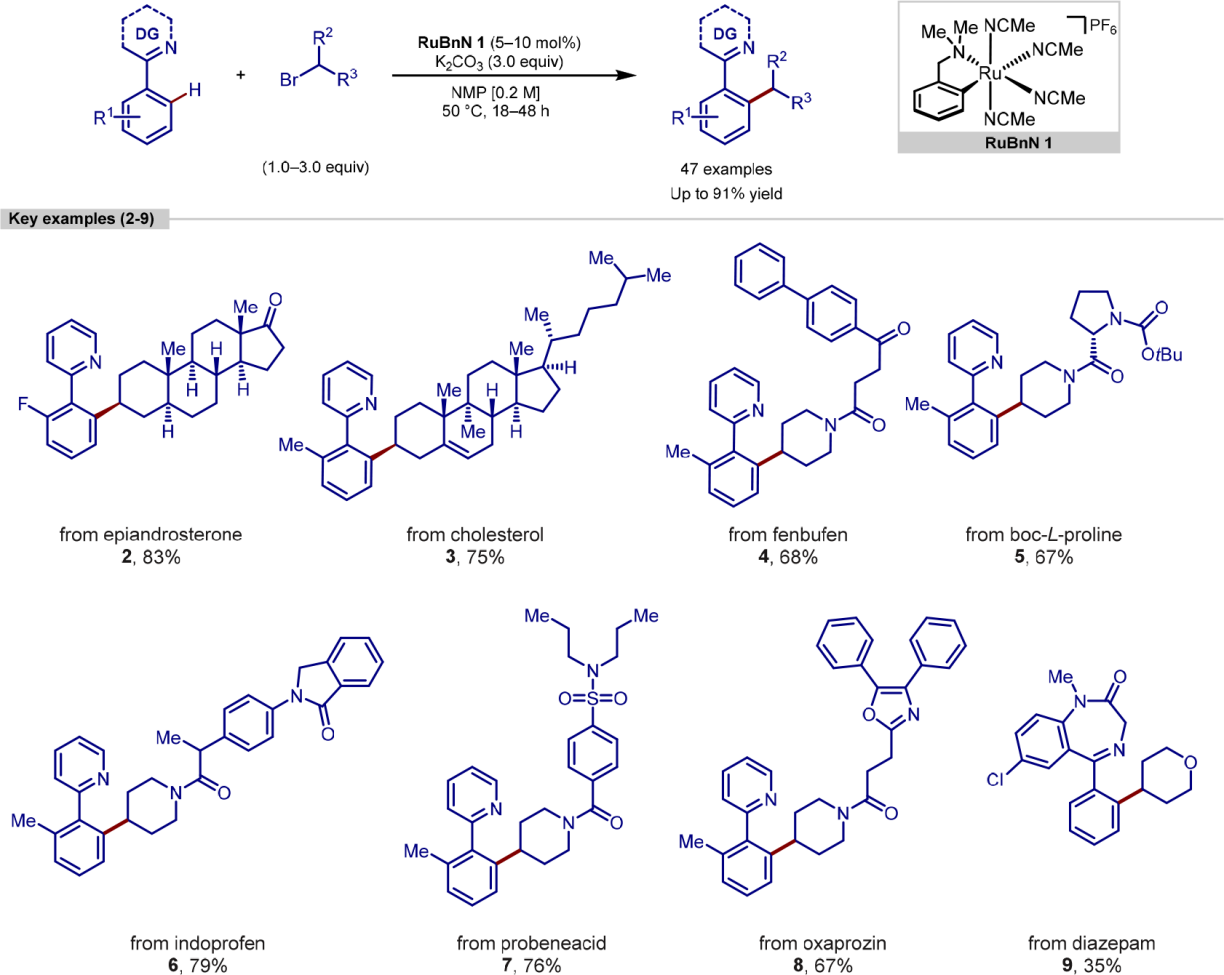
Ruthenium-Catalyzed C(sp^2^)–H Alkylation Using Secondary Alkyl Halides

In 2020, we reported a procedure for the C–H alkylation of directing group arenes with secondary alkyl bromides (Scheme 2).^80^ In contrast to previous ruthenium methodologies using secondary alkyl halides, that generate *meta-*alkylated products,^81^ this procedure delivered a switch in selectivity to give *ortho*-alkylated products, with high selectivity for monoaddition despite the use of an excess of alkyl halide coupling partner. Mechanistic studies were suggestive of the involvement of a key bis-cyclometalated intermediate undergoing oxidative addition with alkyl bromides, avoiding the single electron transfer (SET) step commonly seen with ruthenium that leads to *meta*-alkylated products.

Exploration of the scope of the reaction components showed that a wide range of substituents on 2-phenylpyridine were tolerated, in addition to multiple cyclic and acyclic secondary alkyl halides. Arylketones were also functionalized at the *ortho*-position, *via* an imine directing group strategy. To highlight the robustness of the methodology, a wide range of pharmaceuticals and natural products containing alkenes, amides, ketones, sulfonamides, and nitrogen heterocycles were used as coupling partners after derivatization to a suitable alkyl bromide species. Finally, diazepam, an anxiolytic drug molecule featuring a benzodiazepine core, was alkylated to give **9** in a 35% yield, demonstrating the possible utility of this procedure in drug development.

As with *ortho*-secondary alkylation, numerous reports of primary alkylation with palladium, nickel, cobalt and iron had been reported, although these reactions tended to display narrow directing-group scope, required elevated temperatures or superstoichiometric quantities of Grignard reagents. Ackermann reported the first examples of ruthenium-catalyzed alkylation with primary alkyl bromides;^82−84^ however, these and subsequent reports^85,86^ were limited to simple substrates and functionalities.

**Scheme 3:**
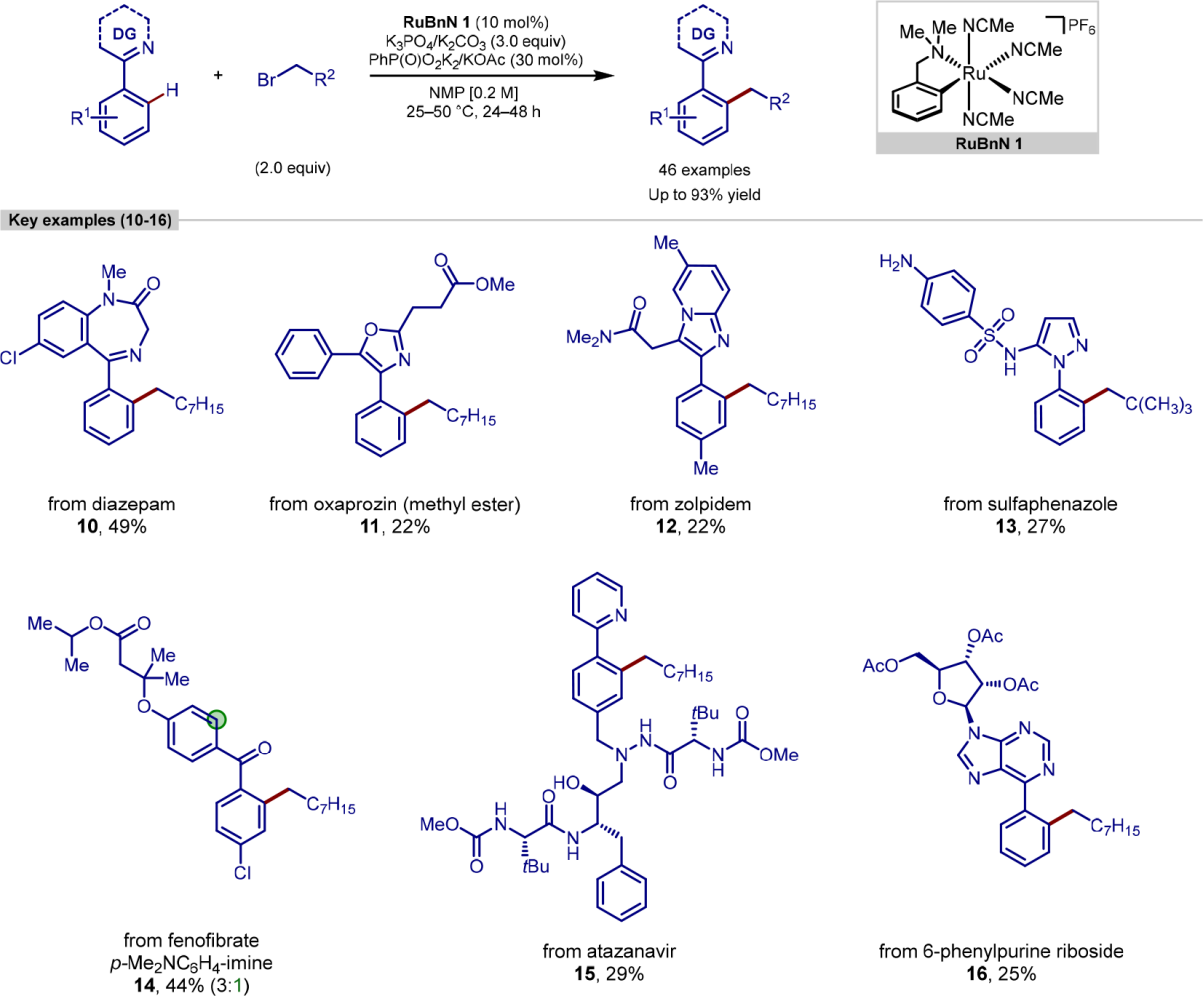
Ruthenium-Catalyzed C(sp^2^)–H Alkylation Using Primary Alkyl Halides

Further investigation by our group into the capabilities of the new class of monocyclometalated ruthenium catalysts resulted in the development of a primary alkylation procedure that proceeded at room-temperature (Scheme 3).^87^ Mechanistic investigations also pointed toward the involvement of a bis-cyclometalated catalytic intermediate, which then enabled oxidative addition of the primary alkyl halide to proceed at room temperature. This methodology displayed high selectivity for the *ortho*-alkylated products, with no *meta*-formation observed in any case. In addition, high selectivity was observed for monoalkylation with most substrates, with the exception of 2-phenylpyridines bearing electron-withdrawing groups, which gave larger quantities of bis-alkylation.

The mild reaction conditions reported allowed the application of this methodology to the late-stage functionalization of some complex molecules. A diverse suite of pharmaceuticals and complex molecules could be successfully alkylated, including: diazepam, sulfaphenazole, oxaprozin methyl ester, zolpidem, atazanavir, and 6-phenylpurine riboside. Fenofibrate, a drug with a benzophenone core, was also able to be alkylated using an imine directing group strategy. These examples highlighted the broad applicability of this method toward C–H alkylation even in the presence of significant molecular complexity and a broad range of functional groups.

**Scheme 4:**
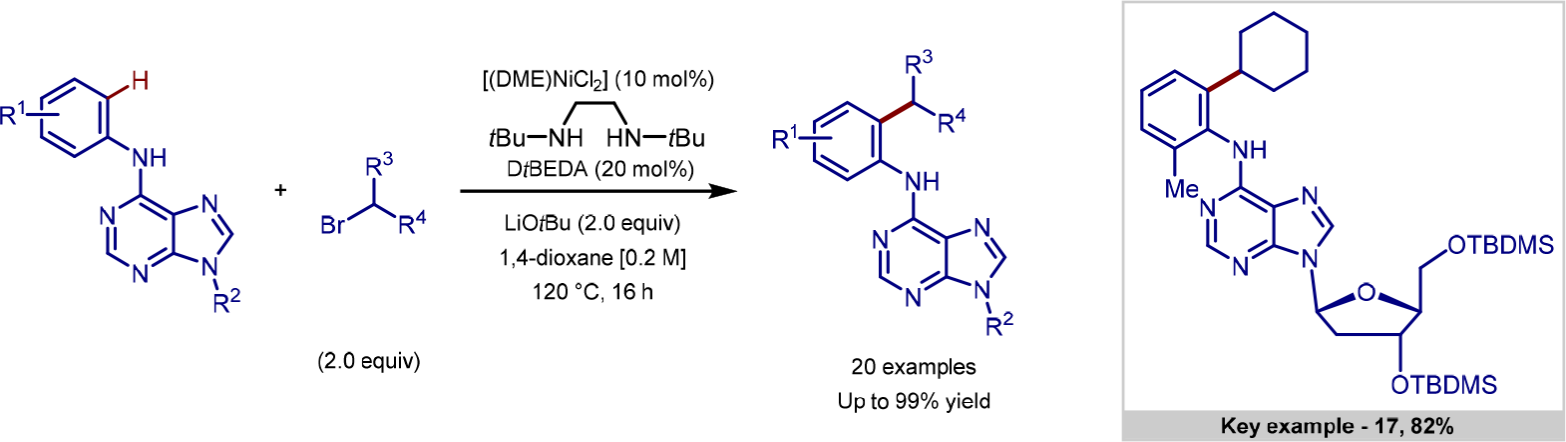
Nickel-Catalyzed C(sp^2^)–H Alkylation of 6-Anilinopurine Derivatives

Ackermann reported a procedure for late-stage primary and secondary alkylation of 6-anilinopurines, using nickel catalysis (Scheme 4). Previous nickel-catalyzed procedures were predominantly limited to benzamide substrates bearing *N*,*N*-bidentate directing groups,^88^ and this was the first report of nickel-catalyzed *ortho*-alkylation of purines.^89^ Both primary and secondary alkylations were reported, including a more complex example of the functionalization of a purine nucleoside to give alkylated product **17** in high yield.

Recently, the alkylation of arenes to install glycosyl groups has gained popularity. These motifs are common in nature, and their presence in a number of bioactive molecules, such as anticancer agent tiazofurin and antidiabetic drug dapagliflozin, demonstrates the need for efficient and stereoselective syntheses. Previous methods for C–H glycosylation operated either through a Friedel–Crafts mechanism, which lacks general regiocontrol of the products, or used bespoke glycosyl donors, organometallic aryl reagents, or transition-metal-catalyzed cross coupling reactions, often in multistep reaction sequences.

**Scheme 5:**
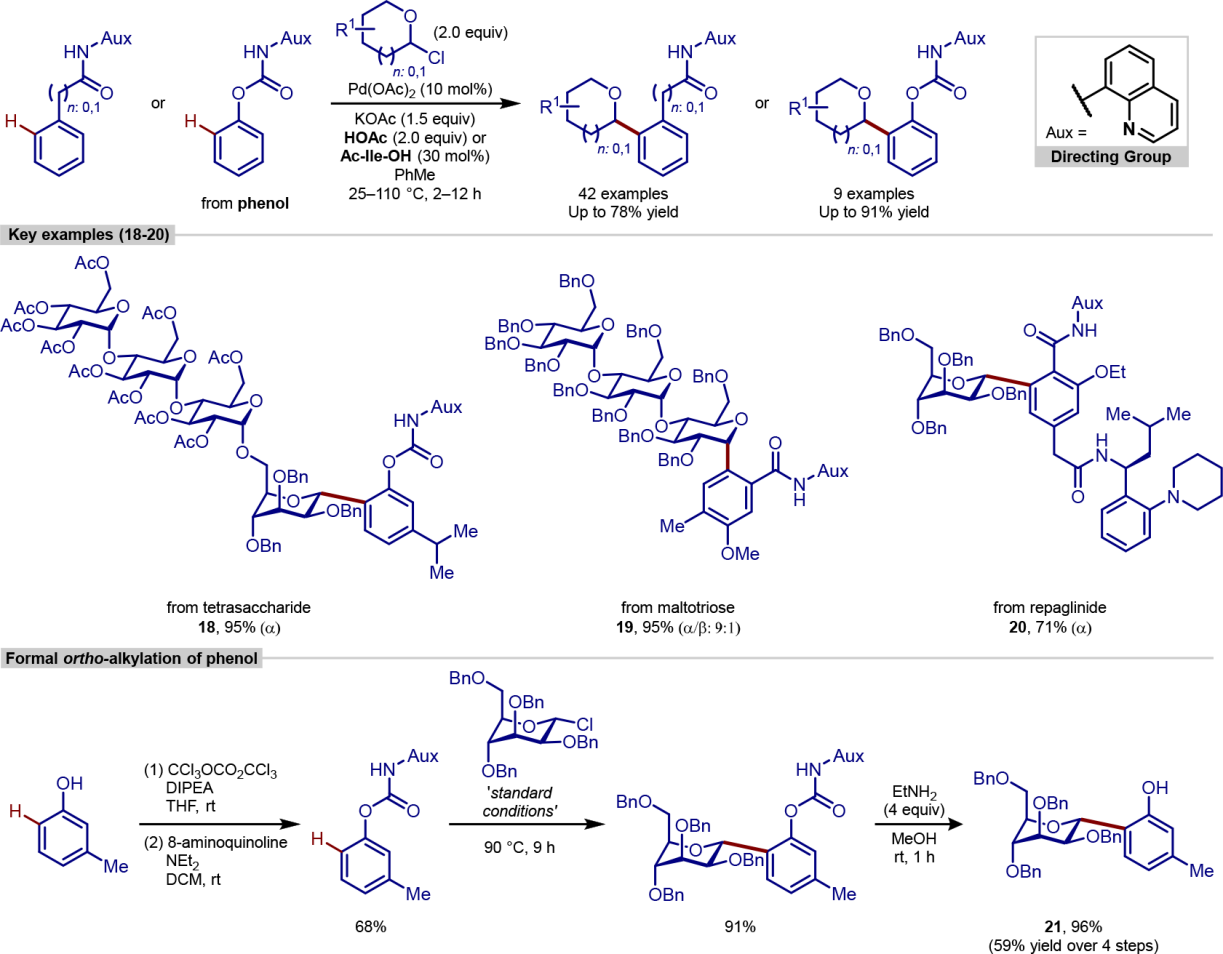
Palladium-Catalyzed *ortho*-C–H Glycosylation Using Glycosyl Chloride Coupling Partners

In 2019, Chen reported a palladium-catalyzed *ortho*-C–H glycosylation procedure for the synthesis of these *C*-aryl glycosides (Scheme 5).^90^ This procedure was able to use easily accessible glycosyl chlorides as coupling partners, which significantly expanded the synthetic utility of the method. Amide directing groups containing an 8-aminoquinoline auxiliary group were used in most cases and proceeded through either a 5- or 6-membered metallacycle intermediate. Different substitution patterns on the arene coupling partner were well tolerated, in addition to heteroarene substrates such as indole, pyrrole, thiophene and furan, generating *C*-heteroaryl glycoside products.

This method was extended to the *ortho*-functionalization of phenol substrates. Transforming the phenol into a carbamate with a linked 8-aminoquinoline auxiliary allowed *ortho*-glycosylation under the same conditions. The synthetic utility of this change was demonstrated by the subsequent removal of the carbamate group under mild conditions (EtNH_2_, 4 equiv, MeOH, r.t, 1 h), to obtain the *ortho*-glycosylated phenol substrates.

Finally, this procedure was extended to more complex substrates without modification of the reaction conditions. Protected tetrasaccharide **18** was installed in the *ortho*-position through a *C*-mannosyl linkage with exclusive α-selectivity. Protected trisaccharide **19** was also installed at the *ortho*-position of a benzamide in high yields and with high α-selectivity of 9:1. An 8-aminoquinoline derivative of repaglinide, an antidiabetic drug, was monoglycosylated in the *ortho*-position, again delivering a good yield and exclusive α-selectivity, showcasing the capability of this methodology to be extended to the functionalization of complex molecules.

Further work has provided alternative methods for C–H glycosylation reactions. A similar nickel-catalyzed variant of this methodology has since been reported for C–H glycosylation of carbamate directing groups containing an 8-aminoquinoline auxiliary group; however, the substrate scope was more limited in comparison.^91^

#### 2.1.2 Directed C–H Alkylation Using Alkenes

In addition to using alkyl groups that contain functional site-specific handles for functionalization, alkenes and alkynes can be used as coupling partners, with the formal addition of a C–H bond across the alkene or alkyne. Their use is particularly attractive due to both the abundance—they are extremely prevalent in many molecules and feedstock chemicals—and the perfect atom economy of these types of reactions, as all atoms from substrates are subsequently found in the products.

Hydroarylation reactions, which involve the addition of aryl C–H bonds across an alkene or alkyne, have been investigated extensively.^92^ Cationic cobalt(III) complexes have been reported previously for these reactions by Kanai, in one of only a limited number of examples that involve well-defined cobalt complexes.^93^

**Scheme 6:**
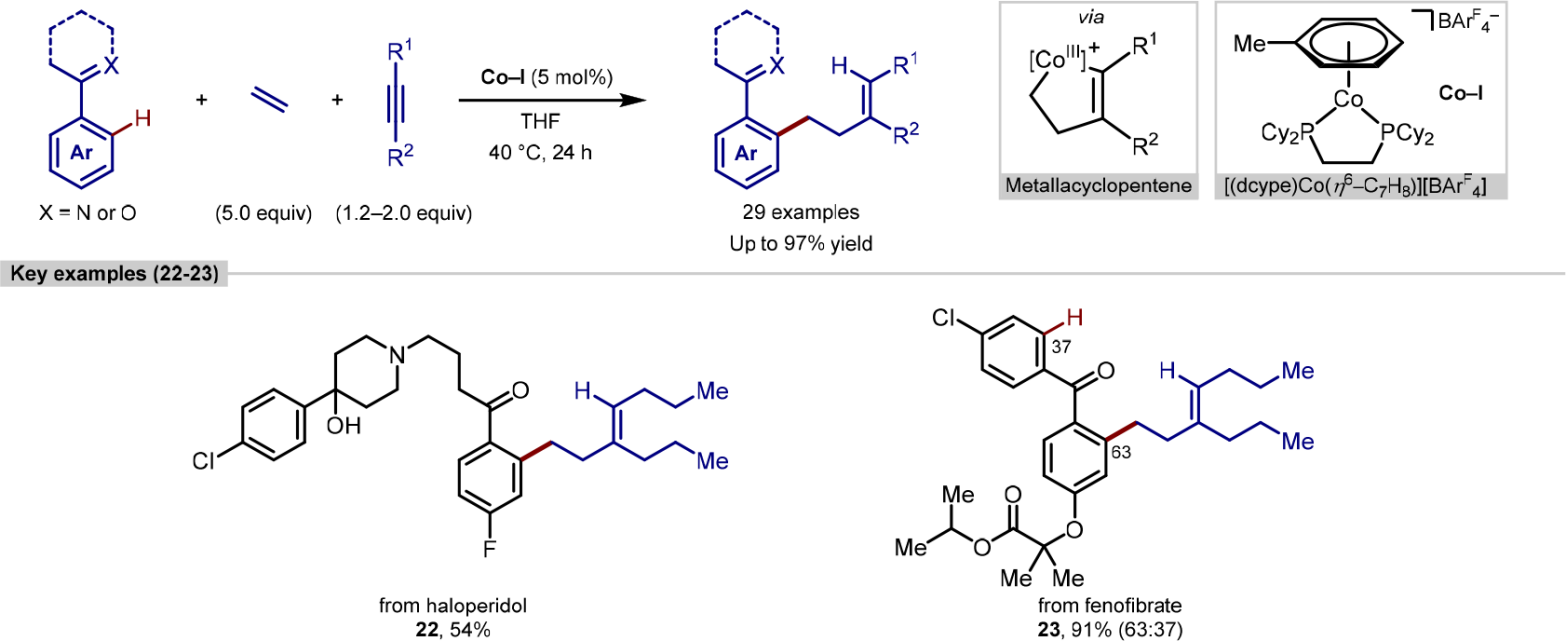
Cobalt-Catalyzed Three-Component Coupling for Synthesis of *ortho-*Alkylated Arenes

Chirik have reported a procedure for the three-component coupling between arenes, ethene and alkynes catalyzed by a cobalt complex, which generated primary alkylated arenes and features examples of late-stage functionalization (Scheme 6).^94^ The mechanism was proposed to proceed *via* a metallocyclopentene intermediate generated from the oxidative cyclization of ethene and an alkyne, followed by C–H functionalization. Similar reports involving a tandem cyclization-hydroarylation process between a 1,6-enyne and an arene were reported by Cheng, and extension to intermolecular systems was considered highly desireable.^95^

The reaction has a broad scope of capable directing groups, arenes, and alkyne components. In all cases, syn-addition across the alkyne generated products with trisubstituted alkene (*Z*)-stereochemistry, and the regioselectivity with unsymmetrical alkynes was strongly influenced by steric effects. The late-stage functionalization of aromatic ketone-containing drug molecules haloperidol and fenofibrate was also demonstrated and gave the corresponding alkylated products in good yields. Two possible sites for functionalization in fenofibrate led to a distribution of products **23**, favoring alkylation at the more election rich arene ring, in a 63:37 ratio.

**Scheme 7:**
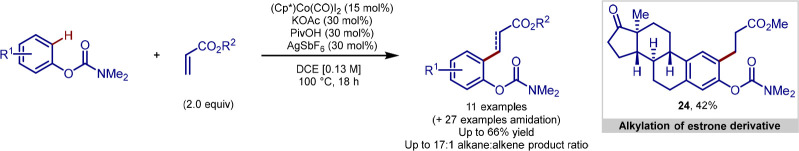
Cobalt-Catalyzed Carbamate-Directed *ortho*-C–H Alkylation and Amidation Using Alkenes

Maji reported further examples of arene C(sp^2^)–H functionalization using cobalt, with the carbamate directed *ortho*-C–H alkylation and amidation using alkenes as coupling partners (Scheme 7).^96^ Functional group interconversion of the phenol of estrone to a carbamate group allowed *ortho*-alkylation to occur under the optimized reaction conditions.

Quinolines and their structurally related analogues are commonly found in pharmaceutical molecules, agrochemicals and natural products. As such, their synthesis and derivatization has been a long-standing target.^97−100^

A common method for the functionalization of quinoline *N*-oxides is by the direct installation of valuable synthetic groups at the C-8 position using transition-metal-catalyzed C–H functionalization. This proceeds *via* formation of a 5-membered metallacycle with metals such as rhodium, iridium or palladium.^97^ Despite this, harsh reaction conditions preclude its use with more-sensitive functional groups, or substrates that are less reactive, reducing the potential scope of the transformation.

**Scheme 8:**
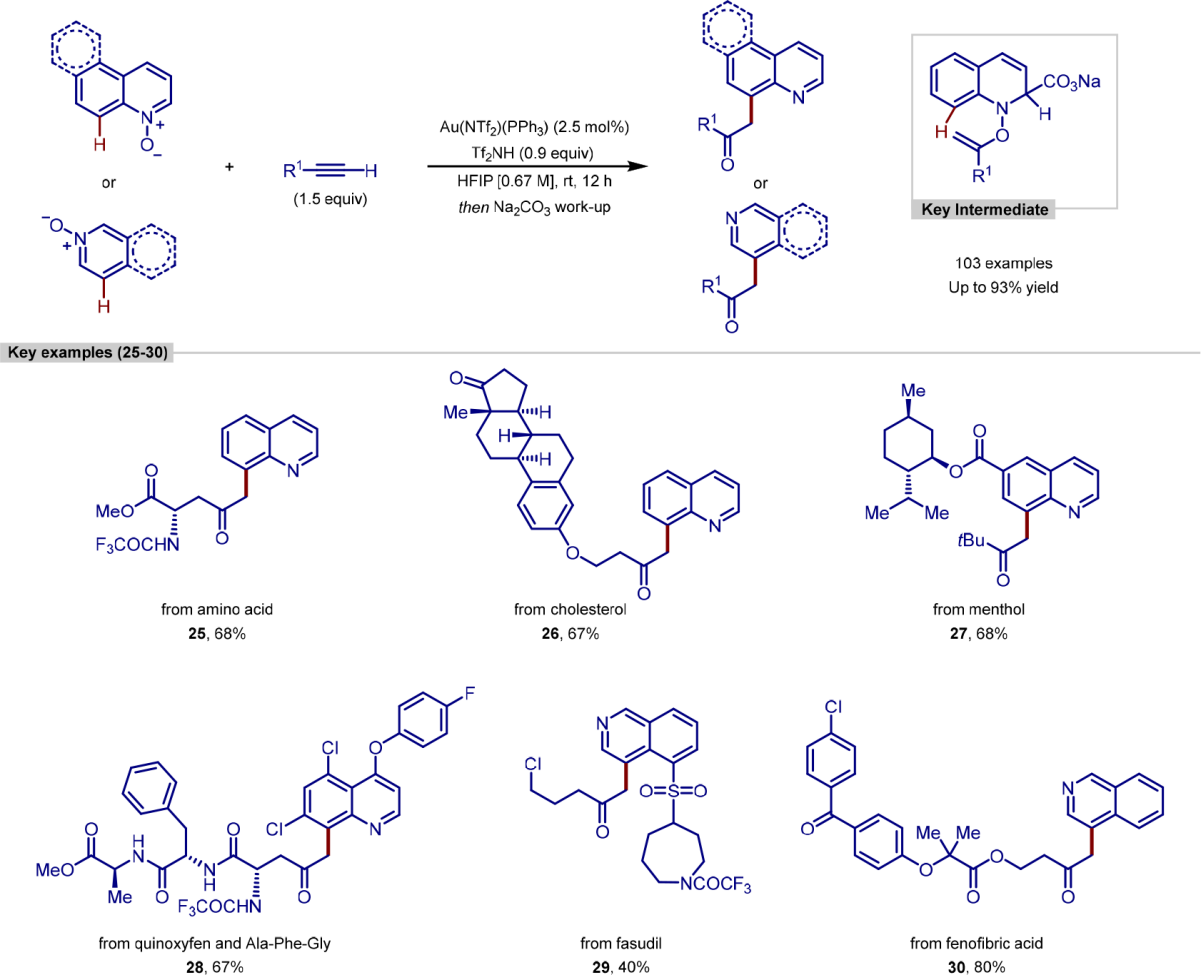
C(sp^2^)–H Functionalization of Heteroarene *N*-Oxides Enabled by a Traceless Nucleophile

In 2020, Hong reported a new procedure for the C–H functionalization of heteroarene *N*-oxides, enabled by a traceless nucleophile (Scheme 8).^100^ The strategy involved the *in situ* formation of *N*-alkenoxyquinolinium salts from quinoline *N*-oxides and unactivated alkynes. *N*-Alkenoxyheteroarenium salts are commonly employed as synthetic equivalents of acylcarbenium cations in umpolung strategies, and as such, efforts have been made to functionalize at the C-8 position of quinolines using this method, through an intramolecular Friedel–Crafts type reaction. Unfortunately, the electrophilic nature of quinolinium salts hinders the Friedel–Crafts step, and hence this strategy remains largely unexplored.

Hong addressed the inherent electron deficiency of quinolinium salts by using a traceless nucleophile. Mechanistic studies indicate that nucleophilic attack at the C-2 position, in this case with a carbonate anion, generates a dearomatized intermediate that is then capable of undergoing a [3,3]-rearrangement to form the C-8 functionalized product. This method allows for mild reaction conditions, proceeding at room-temperature, and displaying a broad scope of quinolines and alkynes, as well as C-8 functionalization of phenanthridine, C-4 functionalization of isoquinolines, and C-3 functionalization of pyridines.

This strategy was also applied to the functionalization of a range of complex molecules. Starting from the *N*-oxide derivatives, quinoxyfen, and analogues of menthol and fasudil were functionalized at the C-8 position. Other quinolines were functionalized using alkyne derivatives of amino acids, small peptides, fenofibrate, and cholesterol, with good yields, site selectivity, and with no observable epimerization of stereocenters.

In comparison with C(sp^2^)–H bond functionalization, the more challenging C(sp^3^)–H bond functionalization is underexplored, likely due to the relative difficulty in metallacycle formation. Despite this, examples of C(sp^3^)–H amidation, arylation, alkenylation, and acylation of 8-alkylquinolines have all been reported with cobalt(III), rhodium(III), and iridium(III) catalytic systems. Complementary ruthenium-catalyzed procedures, particularly alkylations, are relatively underexplored.

Recent reports of C(sp^3^)–H alkylation display a variety of alkylating agents, including α-diazo carbonyls, maleimides, allylic alcohols, and cyclopropanol. In contrast to these substrates, olefin coupling partners are often more abundant, more easily accessible, and would provide more atom-economic methods for alkylation, rendering them attractive coupling partners. In 1993, Murai reported the first example of ruthenium-catalyzed C(sp^2^)–H alkylation using olefin coupling partners.^77^

**Scheme 9:**
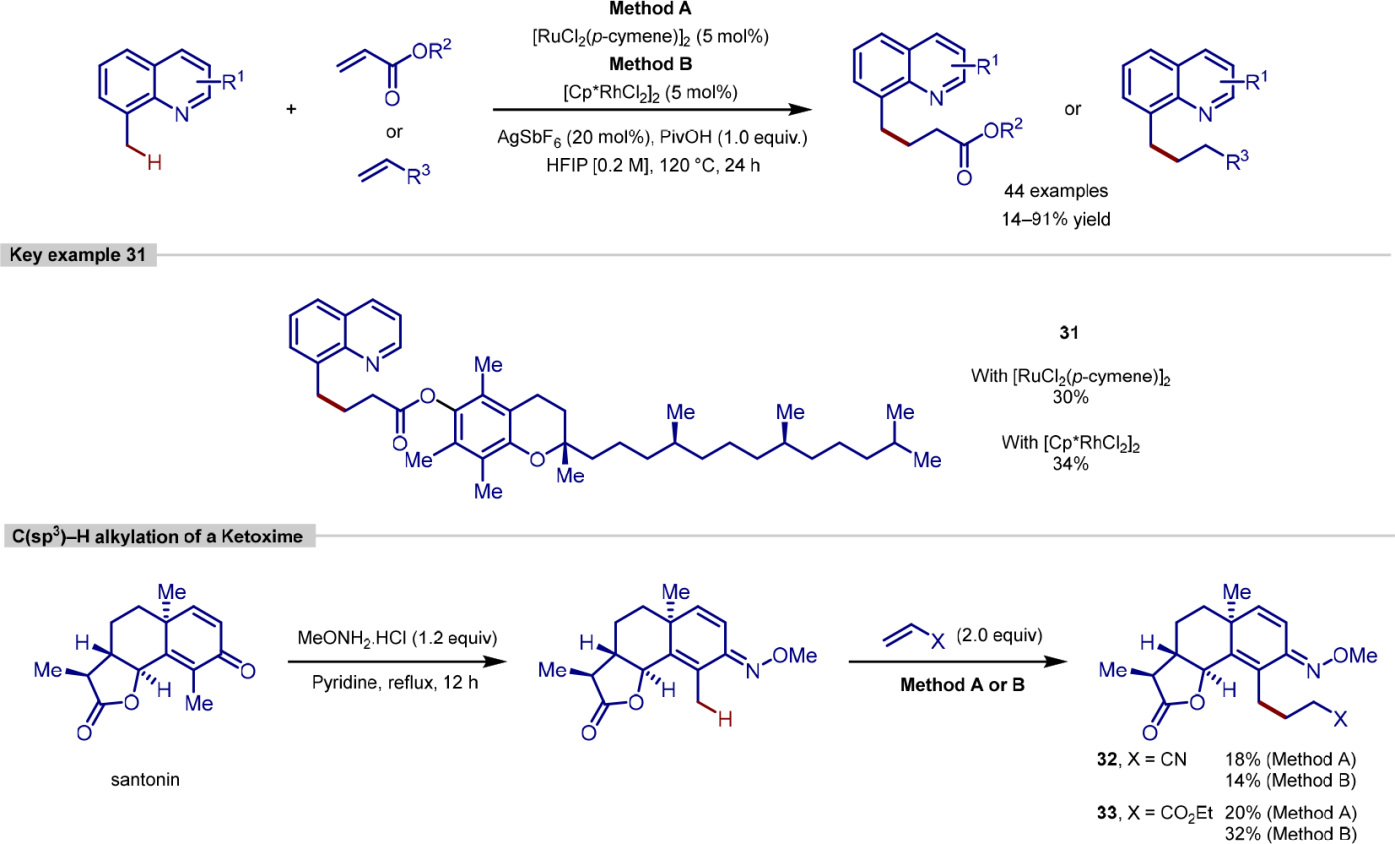
C–H Alkylation of 8-Methylquinolines Using Olefin Coupling Partners

Recently, Sharma have reported the alkylation of 8-methylquinolines using olefins as coupling partners (Scheme 9).^101^ This method was shown to be tolerant to a range of substitution patterns on the quinolone, and a variety of acrylates, styrenes and aliphatic olefins were all coupled successfully, as well as but-3-en-2-one, *N*-methyl maleimide, and an internal alkyne. The robustness of the procedure is demonstrated by its application to the alkylation of (−)-santonin. Derivatization of the ketone functional group to form the ketoxime provided a suitable directing group for *ortho*-functionalization and employing ethyl acrylate or acrylonitrile led to the monoalkylation products.

**Scheme 10:**
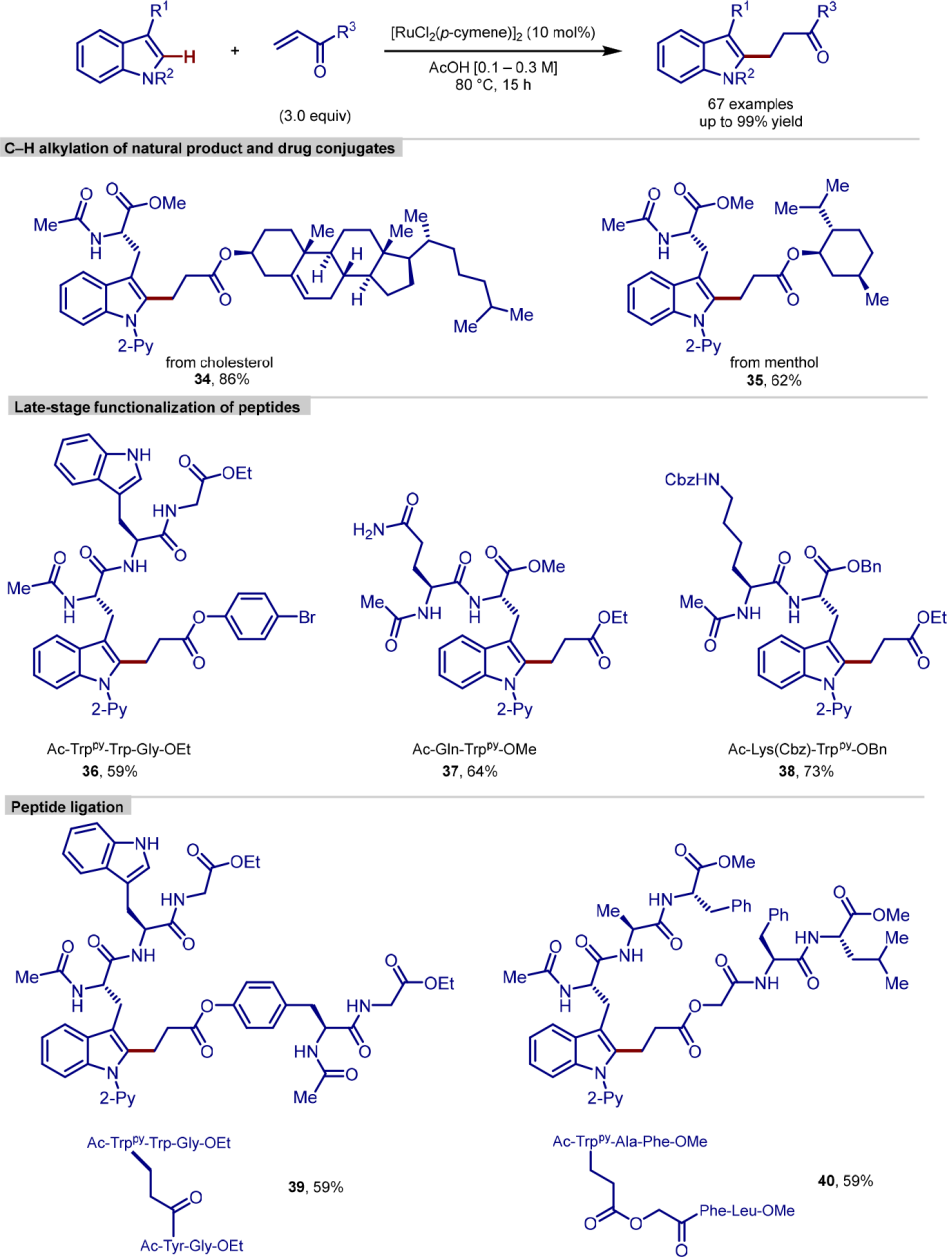
C(sp^2^)–H Alkylation of Tryptophan Residue in Peptides

In 2019, Ackermann reported a peptide C–H alkylation procedure which allows the diversification of several structurally complex peptides (Scheme 10).^102^ Following on from previous work conducted by the group on peptidic C–H arylations, this work provides a method for the chemoselective modification of tryptophan and tryptophan-containing peptides with acrylate coupling partners, via the use of an *N*-substituted directing group strategy. The tolerance of functionality required for subsequent diversification was demonstrated by its application to coupling of drug and natural product conjugates, the ligation of short-chain peptides, and the functionalization of longer chain peptides.

The functionalization proceeded under base-free reaction conditions, which prevented the racemization of amino acids, and the ruthenium catalysis was demonstrated to be both air- and moisture-tolerant, allowing for robust reactivity. A wide range of functional groups, such as bromides, alcohols, alkenes, thioethers, and primary amides, were shown to be tolerated; however, a number of more sensitive functional groups required protection to prevent side reactivity. Interestingly, tryptophan residues unsubstituted at the nitrogen remained unaltered under the reaction conditions, demonstrating the requirement of the directing group strategy to guide selectivity.

In addition to the functionalization of small-chain peptides, the alkylation of nona- and deca-peptides was performed using a solid phase peptide support. This approach demonstrates the advantages of combining on-resin peptide synthesis with this C–H alkylation, allowing for more facile purification of poorly soluble complex peptides.

**Scheme 11:**
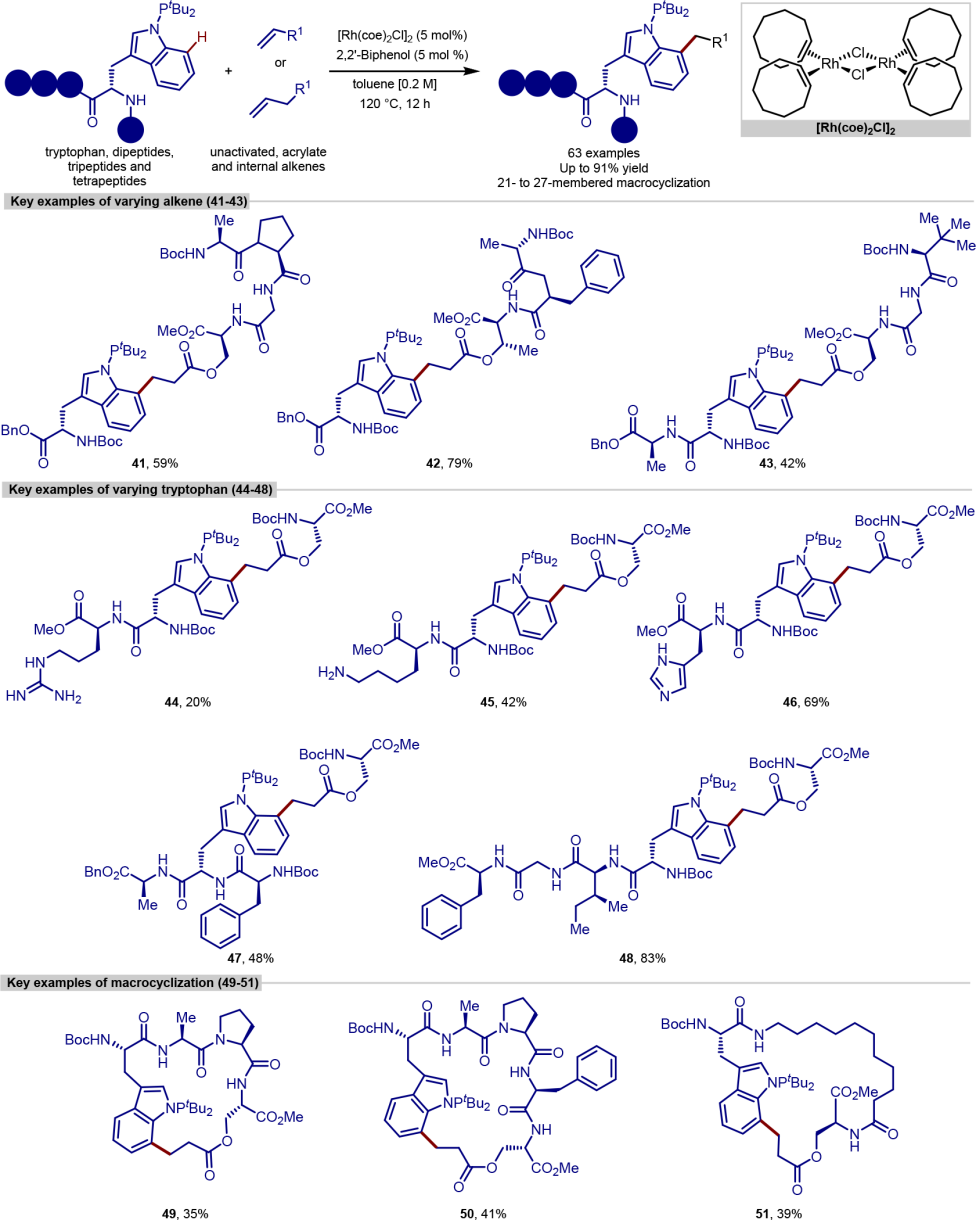
Ligation and Macrocyclization of Amino Acids and Peptides Using a Rhodium Catalyst

More recently, Wang have reported a procedure for the ligation and macrocyclization of tryptophan and tryptophan-containing peptides,^103^ following on from their work on the functionalization of indoles (Scheme 11).^104,105^ In contrast to the procedure developed by Ackermann that allowed functionalization of the more common C-2 position, this method allowed the functionalization at the C-7 position of the indole of tryptophan, utilizing a [Rh(coe)_2_Cl]_2_ complex and an *N*-P(^*t*^Bu)_2_ directing group, which can be cleaved under mild conditions without epimerization.

The scope of coupling partners was very broad for this reaction, and the tryptophan residue in either a terminal or internal position in a range of dipeptides, tripeptides, and tetrapeptides was functionalized selectively and in good yields. Peptides containing protected aspartic acid, cysteine and lysine residues were tolerated, in addition to unprotected tryptophan, methionine, tyrosine and serine residues. Glutamine, arginine, lysine and histidine residues typically pose problems for transition-metal-catalyzed procedures; however, these were found to be tolerated and gave moderate yields with an increased catalyst loading.

Acrylate derivatives of dipeptides and tripeptides were found to function well under the reaction conditions, allowing a rapid buildup of molecular complexity in a single step. In addition to acrylates, several examples of unactivated alkenes as coupling partners are shown to work. Internal alkenes were also found to function as coupling partners, after a previously reported initial regioselective alkene isomerization process to generate the terminal alkene,^106^ with no observed formation of the branched product. Finally, peptide macrocyclization was achieved by the intramolecular alkylation of tryptophan residues, forming 21–27 membered macrocyclic rings.

#### 2.1.3 Dehydrative C–H Alkylation

Early work by Yi demonstrated that a cationic ruthenium hydride complex was an efficient catalyst for the coupling reaction between aryl ketones and alkenes.^107,108^ Preliminary mechanistic studies pointed toward a dehydrative-driven mechanism, which led them to investigate the use of alcohols as coupling partners.

In 2011, Yi went on to report a novel coupling between alkenes and alcohols to form C(sp^2^)–C(sp^3^) bonds.^109^ The reaction proceeded under very efficient catalytic conditions, with only 0.5 mol % catalyst typically employed per reaction, although the authors have also shown that a yield of 51% could be achieved using only 0.0005 mol % of catalyst, equating to an impressive TON of 102,000.

The reaction displayed a broad scope of simple molecules, with cyclic olefins, benzopyran, *N*-methylindole all working well as coupling partners. Under the reaction conditions, isomerization, hydrogenation, or dehydrogenation was observed with some substrates. In addition to varying the olefin coupling partner, both aliphatic and aromatic substituted alcohols functioned well, with secondary aliphatic alcohols reacting more slowly.

**Scheme 12:**
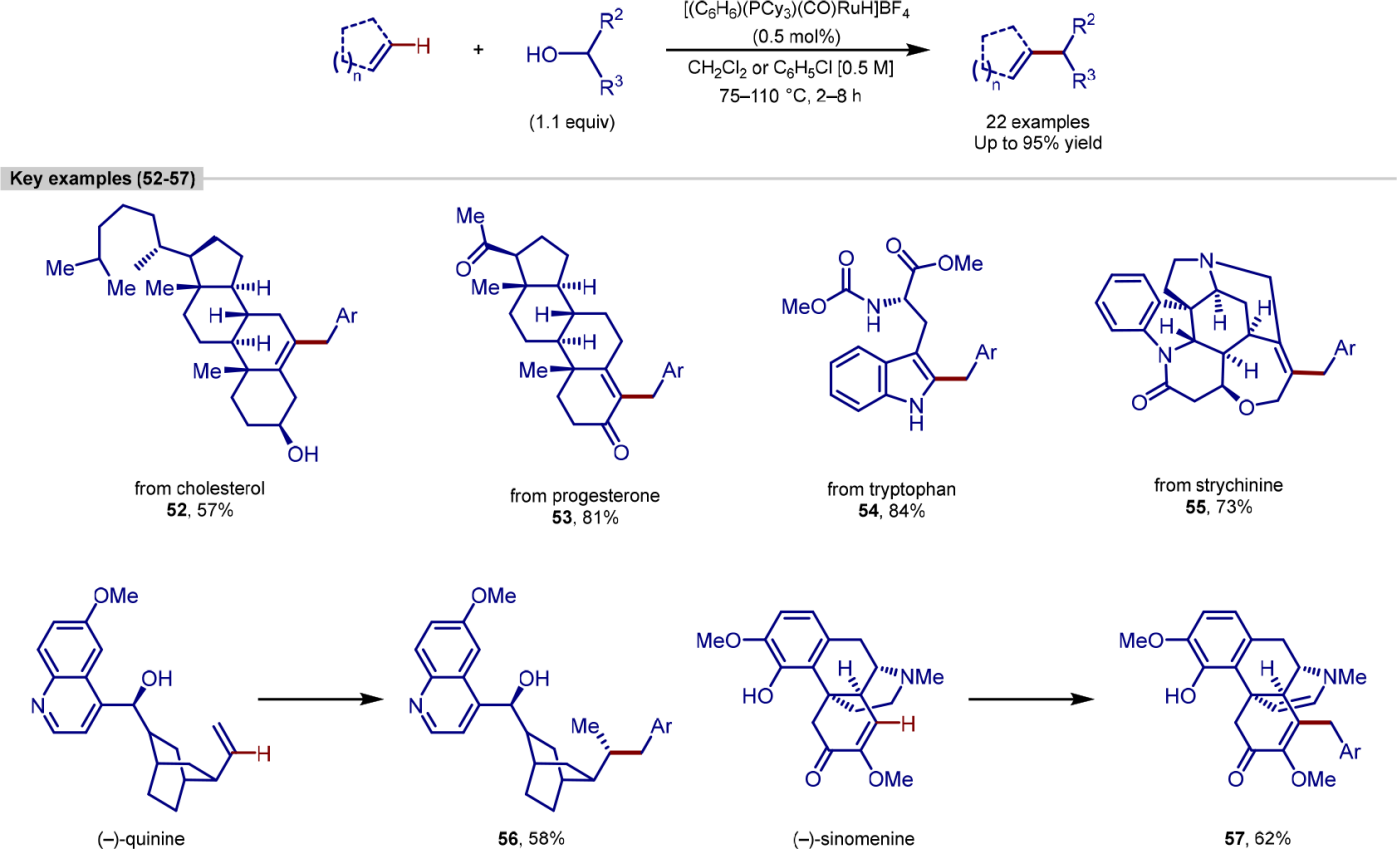
Ruthenium-Catalyzed C(sp^2^)–H Alkylation of Alkenes Using Alcohol Coupling Partners

Interestingly, this procedure was able to be applied to a range of bioactive alkene-containing compounds (Scheme 12). Both cholesterol and progesterone were alkylated successfully using 4-methoxybenzyl alcohol, leaving both the alcohol and carbonyl functional groups intact. Similarly, *N*-methoxycarbonyl-l-tryptophan methyl ester and (−)-strychnine were able to be alkylated without affecting the amino, amide, or ester functional groups. (−)-Quinine underwent regioselective alkylation to form the branched alkylation product, although this was accompanied by a diastereoselective hydrogenation to give product **56**. Conversely, (−)-sinomenine, a morphine analogue, was successfully alkylated using this method, but in this case, this was accompanied by dehydrogenation adjacent to the amino group, resulting in the enamine product **57**. In all cases, these molecules were alkylated without any accompanying racemization.

**Scheme 13:**
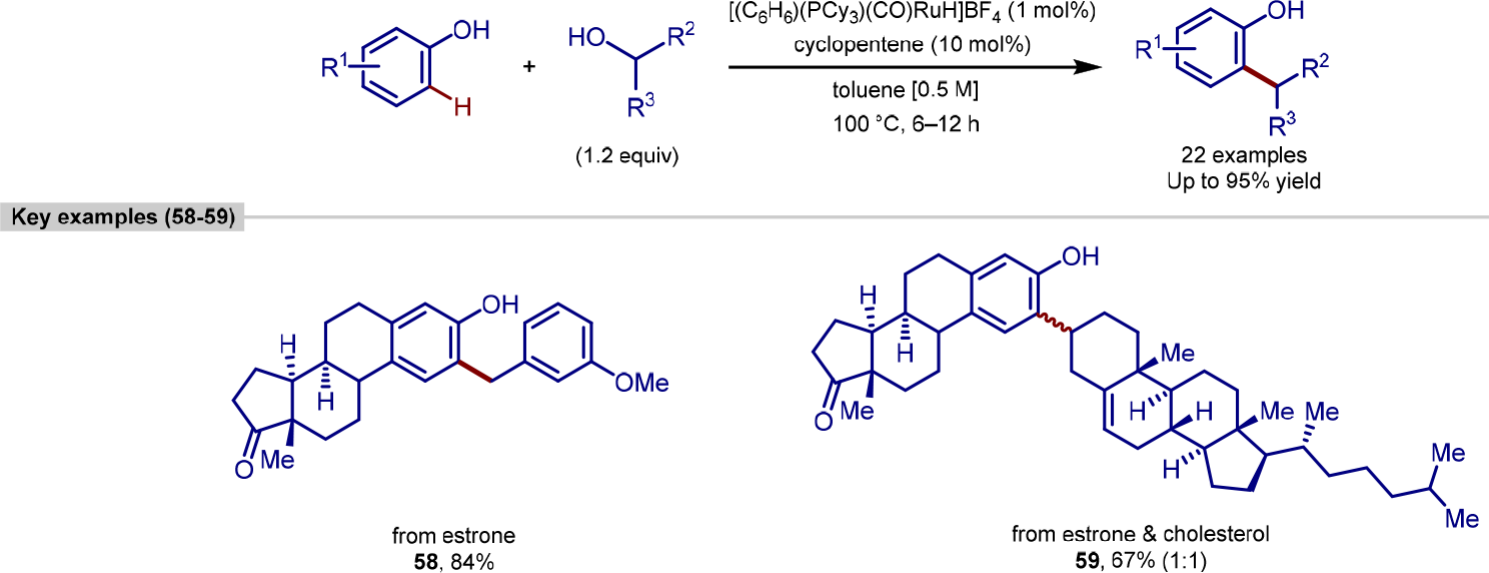
Dehydrative *ortho-C*(sp^2^)–H Alkylation of Phenols Using Alcohols

In addition to this work, in 2012, Yi reported a complementary procedure for the catalytic C–H alkylation and alkenylation of phenols, again using alcohols as coupling partners (Scheme 13). Using the same catalytic ruthenium hydride catalyst [(C_6_H_6_)(PCy_3_)(CO)RuH]BF_4_, phenols were able to be either alkylated or alkenylated at the *ortho*-position, depending on the conditions used.^110^

Primary, secondary, and benzylic alcohols were all capable of functioning as the alkylating agent. A range of simple phenols was functionalized cleanly at the *ortho*-position, with high yields achieved regardless of the substitution pattern. Interestingly, ketone substrates were shown to be suitable coupling partners, generating the alkylated phenol product through a dehydrative mechanism. It was shown that the addition of stoichiometric quantities of sacrificial alkenes led to the alkenylated products, likely through a dehydrogenative pathway and, in addition to the range of alcohols described previously, the authors found that coupling with diols afforded a range of benzofuran products.

This methodology could be applied to the alkylation and alkenylation of a number of biologically relevant phenol and alcohol compounds. Estrone underwent alkylation using either 3-methoxybenzyl alcohol as a coupling partner, or cholesterol, in which case a 1:1 diastereomeric mixture of the corresponding coupling product was generated. Further examples with 1,2-diols led to the annulation products, with estrone, tyrosine, and hydrophenanthrenol forming the corresponding benzofuran derivatives, and a coumarin derivative leading to an α-substituted furanocoumarin compound. In all cases, these reactions proceeded with high functional group tolerance and without detectable racemization.

### 2.2 Remote Alkylation

In contrast to traditional C–H bond functionalization methodologies that utilize Lewis basic groups to direct a metal catalyst into the proximity of a specific C–H bond, some transition metals can enable functionalization of C–H bonds at remote positions.^111−117^ Such remote functionalization is facilitated by the chelation-assisted formation of a classical metallacycle, which then activates distal positions for functionalization.

Frost reported the first example of such remote functionalization in 2011, describing the ruthenium-catalyzed sulfonation of arenes at the position *meta* to the directing group, overriding the traditional *ortho-*selectivity seen with other transition metals.^118^ Since then, the installation of alkyl, halide, nitro, acyl, and even aryl groups have been reported, and these have been covered in recent reviews.^119,120^ The majority of the reports on this topic are methods for *meta*-alkylation, and this section will focus on methods for remote C–H alkylation that contain examples of late-stage functionalization.

**Scheme 14:**
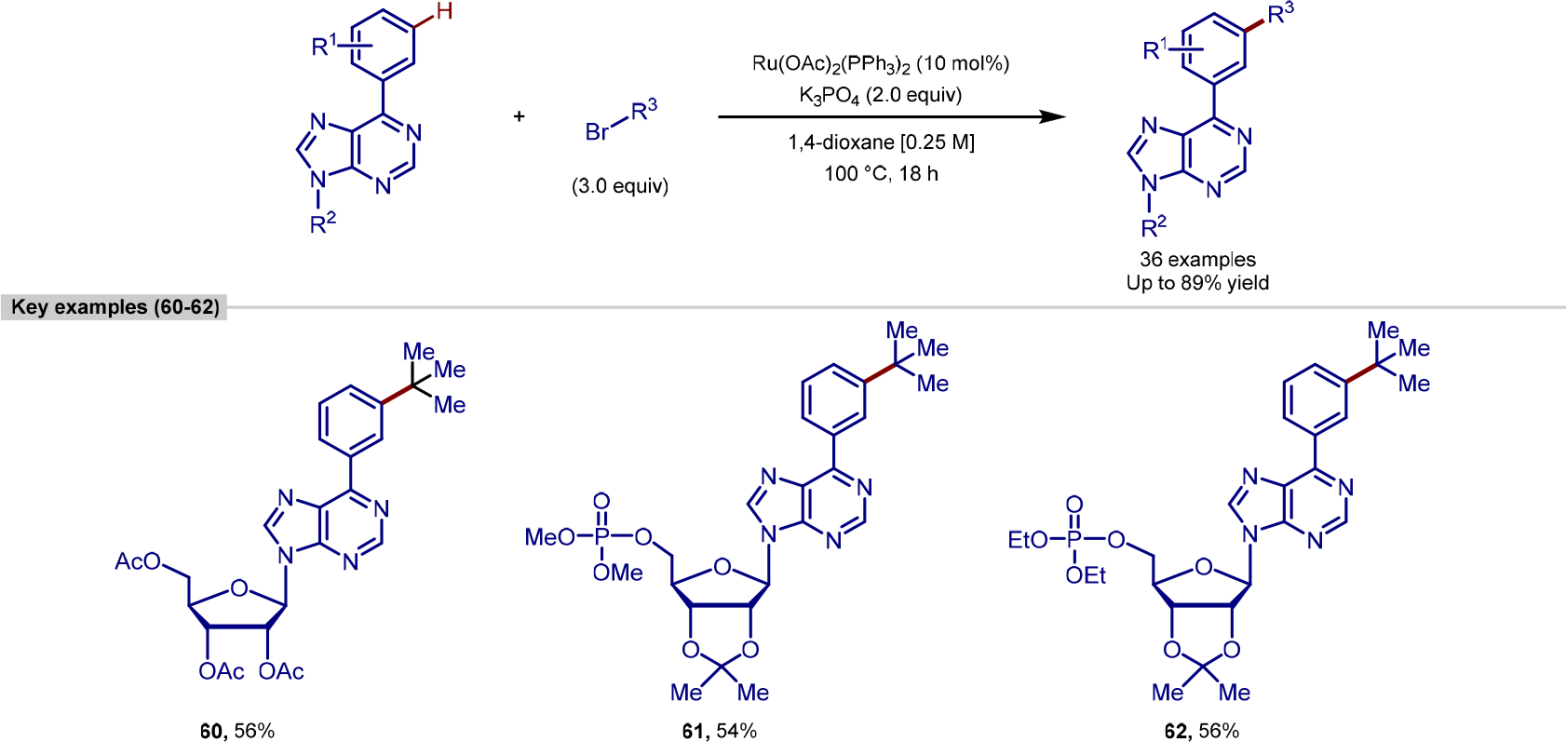
Ruthenium-Catalyzed Remote Alkylation of Arylpurines

The first example of *meta*-alkylation using this σ-activation method was reported by Ackermann in 2013, utilizing *N*-directing group arenes, [RuCl_2_(*p*-cymene)]_2_ as the catalyst, and unactivated secondary alkyl halides (Scheme 14).^121^ Further work by Ackermann reported the remote functionalization of purines, using secondary, tertiary, and activated primary alkyl bromides as substrates.^122^ Despite the numerous C–H bonds that could be functionalized in these molecules, only *meta*-selectivity was observed, generating moderate to high yields with the substrates reported.

In addition to purine as directing group, oxazoline, pyridine, pyrimidine, indazole, and pyrazole directing groups were shown to promote this reactivity. To showcase the utility of this methodology, it was applied to the *meta*-C–H alkylation of a number of sensitive nucleosides. Calculation of the relative radical Fukui indices showed a strong preference for the C–H bond located *para* to the ruthenium in the monocyclometalated species.

**Scheme 15:**
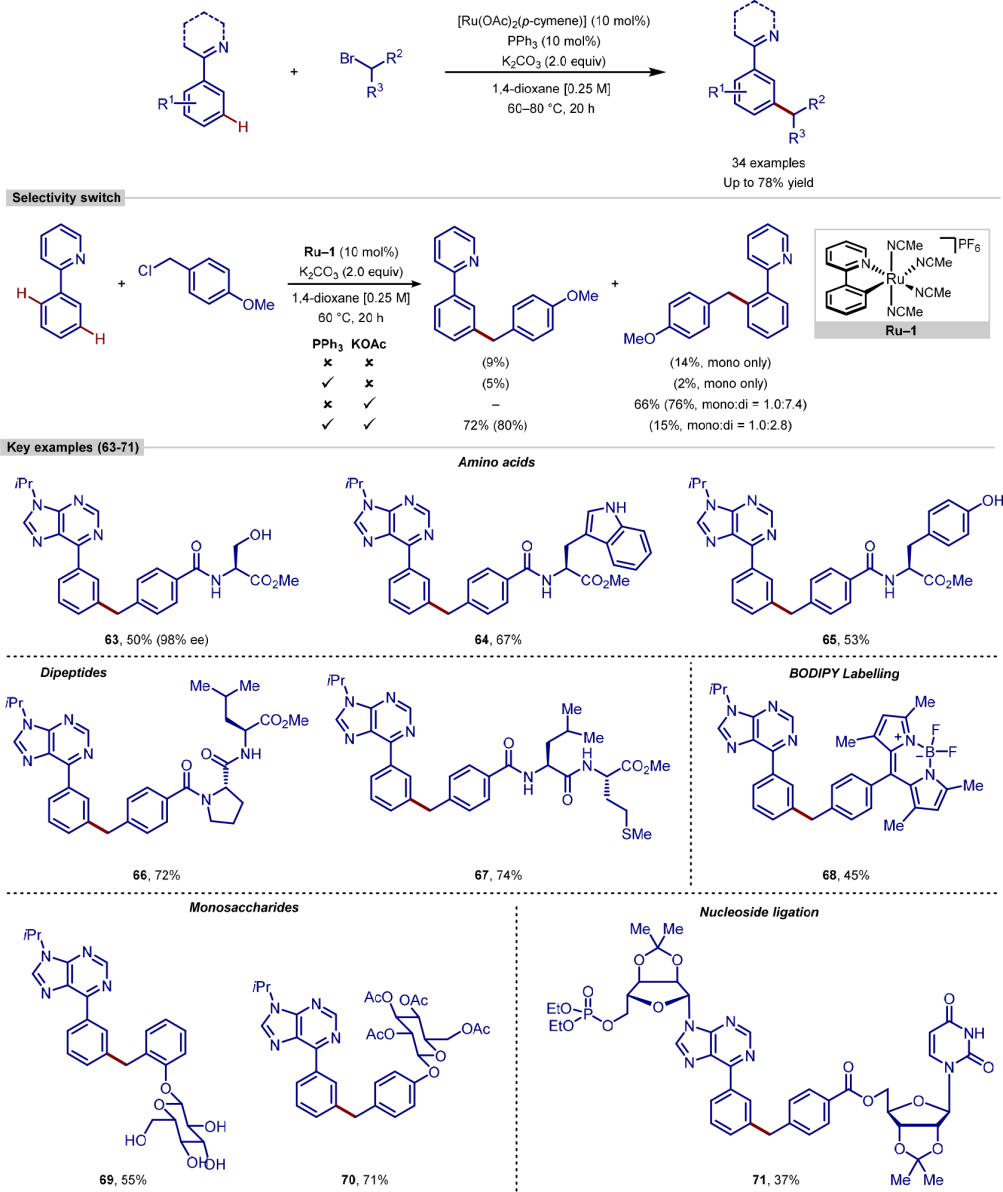
Effect of Additives on Ruthenium-Catalyzed C(sp^2^)–H Alkylation

A subsequent investigation into this type of catalytic system revealed the effect of carboxylate and phosphine additives on the *ortho-/meta-*selectivity (Scheme 15).^123^ Using a monocyclometalated catalyst **Ru-1**, carboxylate additive KOAc was shown to be necessary for reactivity, but predominantly generated the *ortho*-alkylated product. Interestingly, addition of phosphine ligand PPh_3_ with KOAc led to a switch in selectivity, instead favoring the *meta*-alkylated product.

With improved conditions for the *meta*-alkylation, a wider scope of substrates was reported, including the late-stage diversification of a range of complex molecules. *meta*-C–H Alkylation of arene nucleobases with fluorescent BODIPY tags, amino acids and dipeptides, lipids, drugs, protected and unprotected sugars, and nucleosides, were all reported in good yields and with no reported *ortho*-functionalization.

**Scheme 16:**
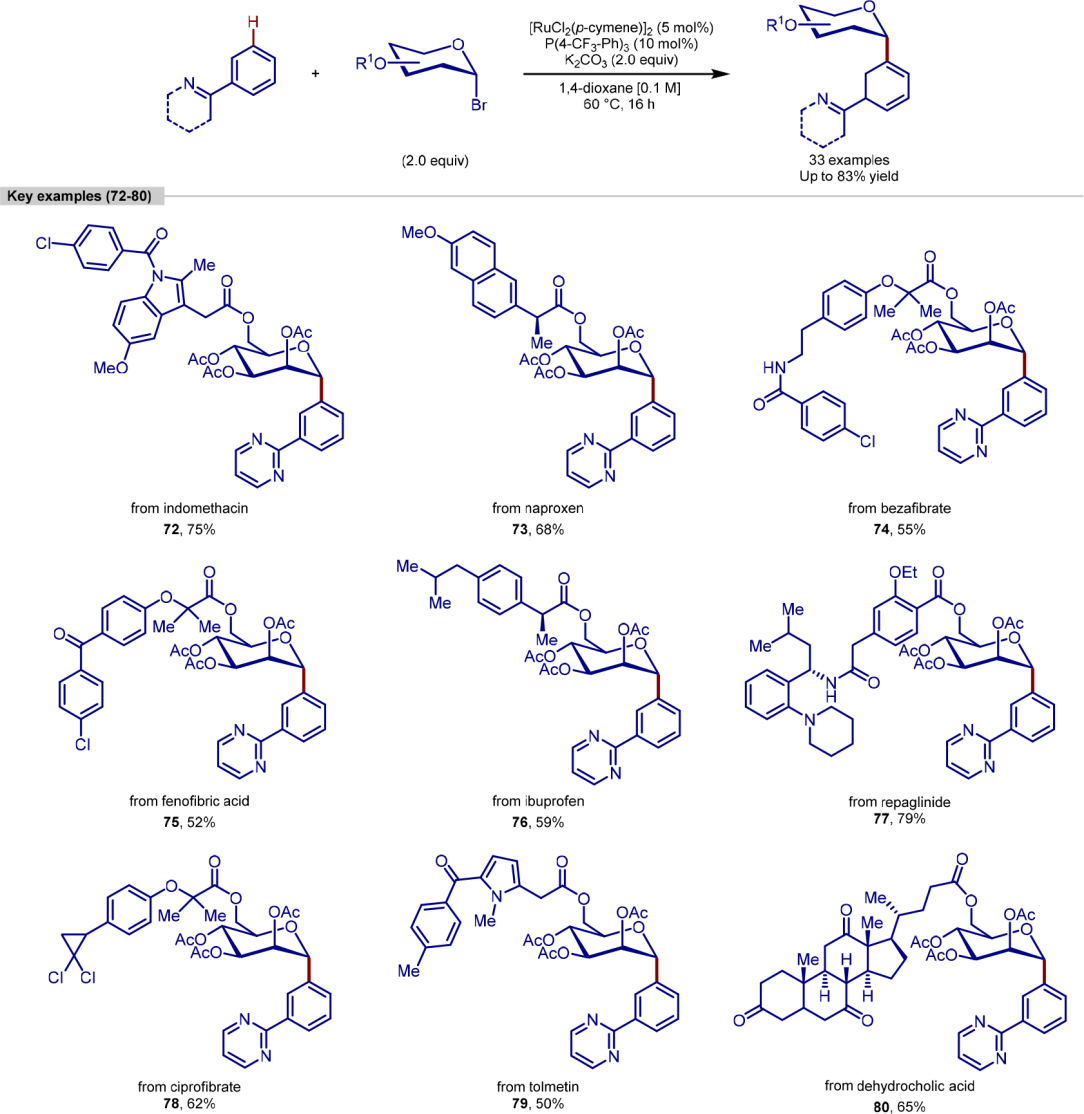
Ruthenium-Catalyzed *meta-*C–H Glycosylation of *N*-Directing Group Arenes

Further work by Ackermann into C–H functionalizations of biorelevant substrates led to the report of a procedure for *meta-*C–H glycosylation of arenes containing *N*-directing groups, using ruthenium catalysis (Scheme 16).^124^ While examples of *ortho*-glycosylation catalyzed by palladium were already known, methods for the synthesis of *meta*-substituted C–H glycosylation products were limited to Catellani-type reactions that generate *meta-*substituted products *via ortho*-glycosylation of aryl halides bearing one *ortho*-substituent, followed by a hydrogenation termination step.^113^

In addition to broad scopes of directing group arenes and glycosyl bromide donors, this method was applied to late-stage *meta*-C–H glycosylation. To achieve this, structurally complex natural products and drugs were derivatized by adding a glycosyl bromide donor through an ester linker, which could then undergo *meta*-addition to a directing group arene. Using this strategy, derivatives of indomethacin, bezafibrate, naproxen, fenofibric acid, dehydrocholic acid, ibuprofen, repaglinide, ciprofibrate, and tolmetin were all appended successfully in the *meta*-position in good yields, despite a wide range of sensitive functional groups and stereocenters being present in these complex molecules.

**Scheme 17:**
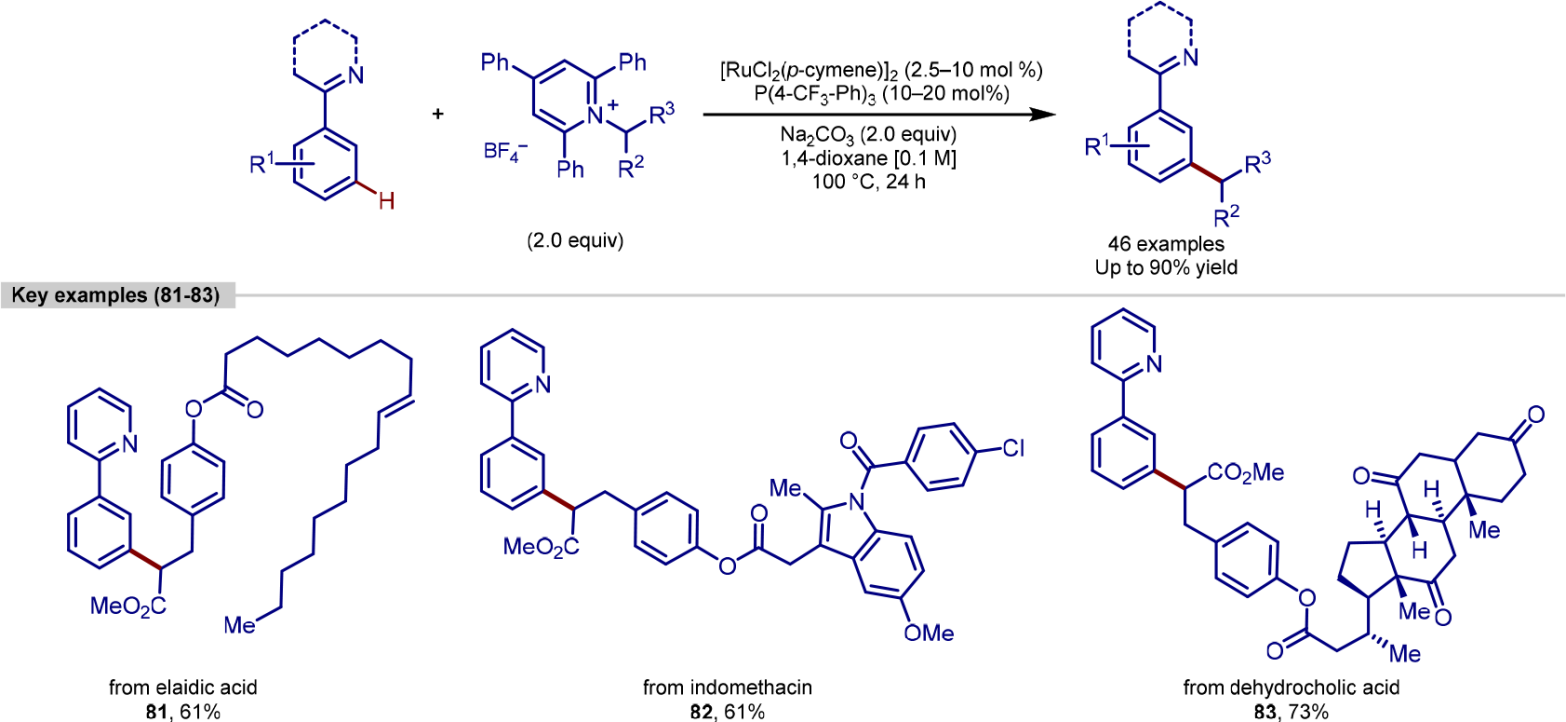
Ruthenium-Catalyzed Deaminative *meta*-C–H Alkylation Strategy

Alternative methods for *meta*-functionalization with ruthenium were also reported by Ackermann (Scheme 17). One such method utilized pyridinium salts in place of alkyl halides as alkylating agents, in a deaminative strategy.^125^ These salts are known to be alkyl radical precursors and can be formed by a reaction between the corresponding primary amines and pyrillium salts, effectively allowing amines to act as coupling partners.^126−129^*N*-Benzylpyridinium salts were shown to be suitable coupling partners, and the pyridinium derivatives of amino acids also functioned well. Like previous reports, the use of a linker-strategy allowed the incorporation of bioactive molecule derivatives of indomethacin, dehydrocholic acid, and elaidic acid at the *meta*-position.

**Scheme 18:**
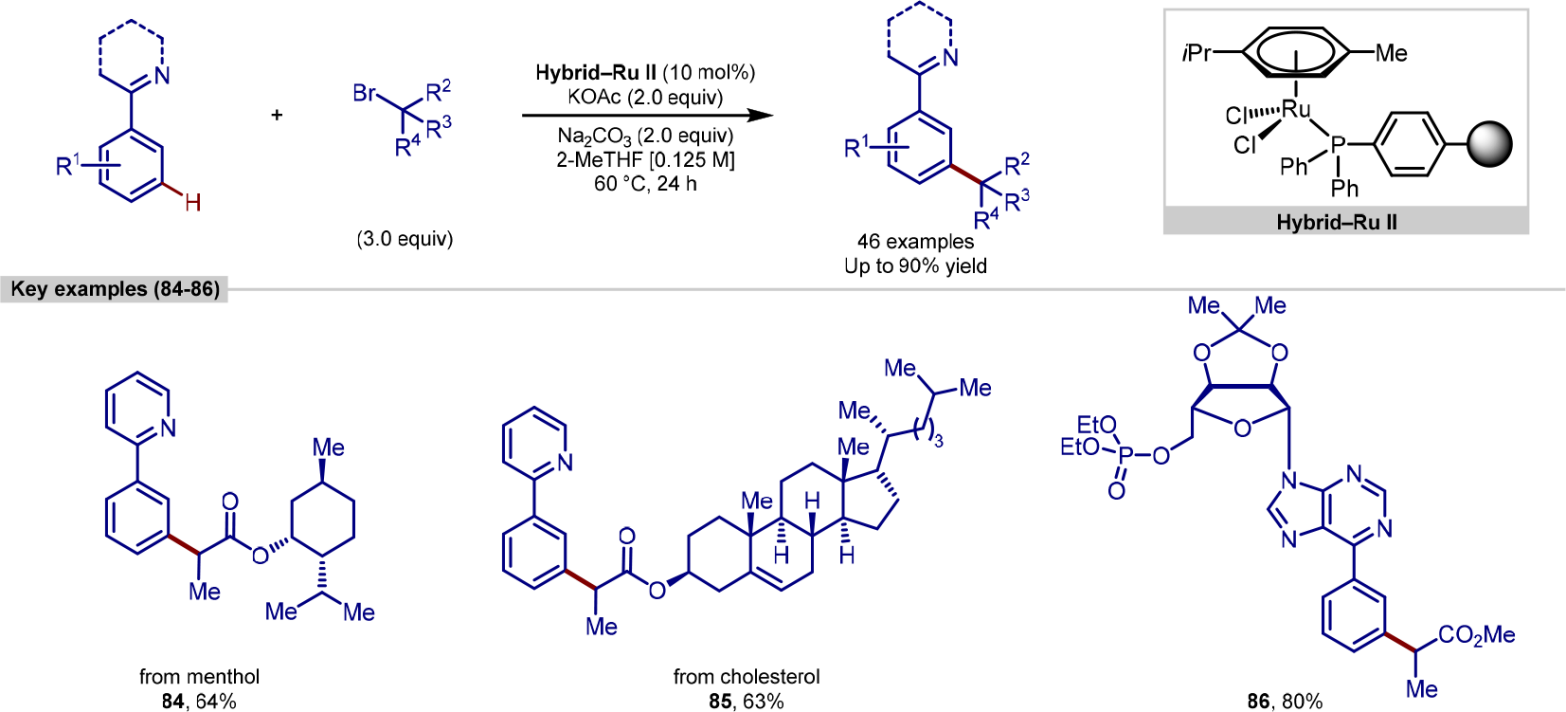
Recyclable Ruthenium Catalyst for *meta-*C–H Alkylation

Another advance in *meta*-C–H activation with ruthenium allowed the use of a recyclable ruthenium catalyst (**Hybrid-Ru II**) to facilitate the reaction (Scheme 18).^130^ Previously reported methodologies were only shown to work with homogeneous catalysts, which significantly restricted the ability of catalyst separation and reuse after the reaction and gave rise to the possibility of the presence of trace metal impurities in target compounds. In this report, a ruthenium complex was immobilized onto a solid support using an organic linker containing a phosphine donor ligand in a similar strategy that had been previously used by the groups of Davies, Jones, Sawamura, and Ackermann.^131−135^ A scope of reaction substrates showed tolerance of a range of functional groups and directing groups, with examples of functionalization of menthol and cholesterol derivatives, along with the *meta*-alkylation of some complex nucleosides.

**Scheme 19:**
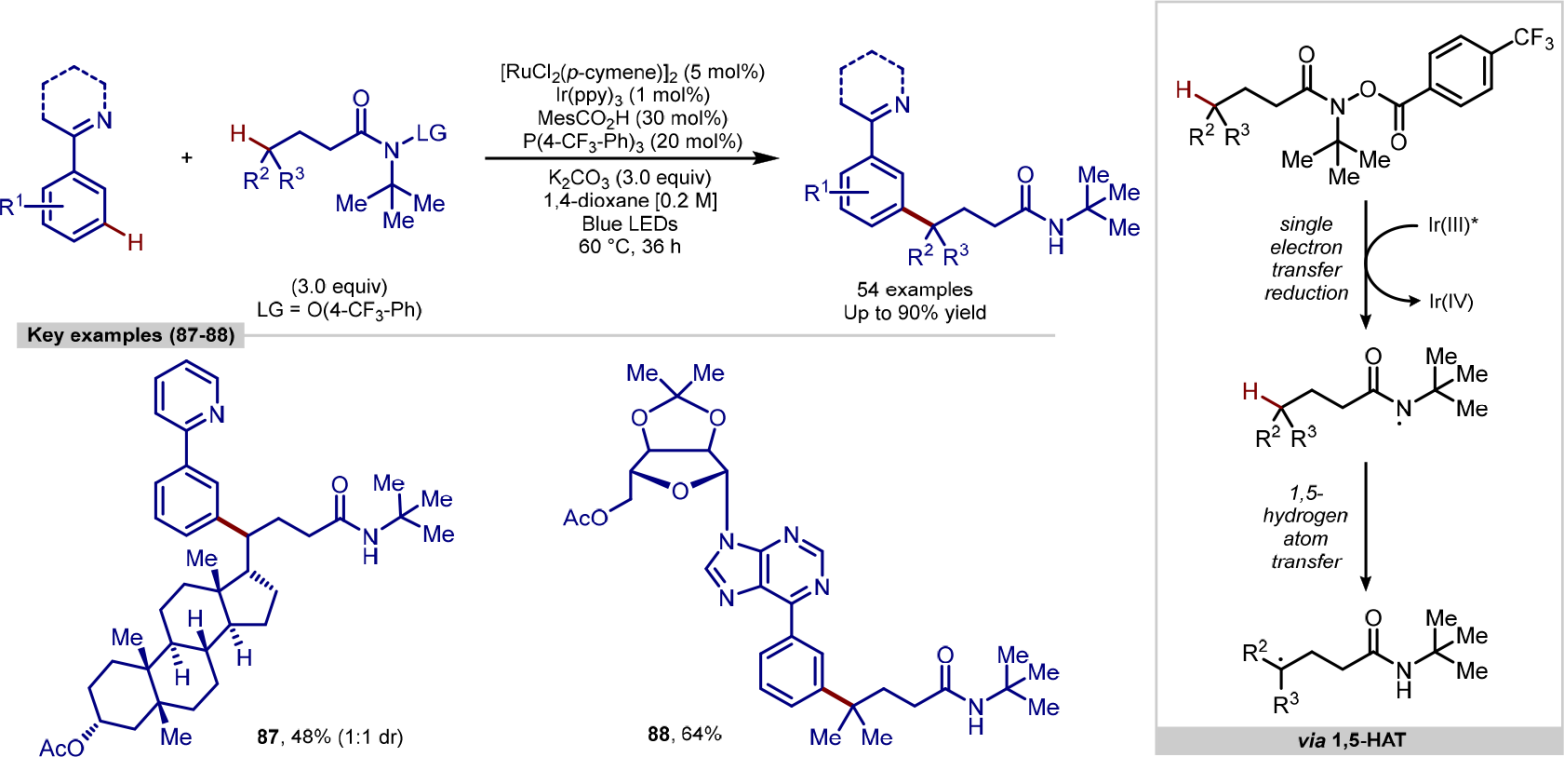
Ruthenium/Iridium Dual-Catalysis for *meta-*C–H Alkylation *via* 1,5-HAT

The majority of ruthenium-catalyzed *meta*-functionalization procedures utilize a single ruthenium catalyst that is proposed to participate in multiple steps of the catalyst cycle, including C–H activation for metallacycle formation, and SET for alkyl radical generation. Recently, Liang developed a ruthenium/iridium dual catalytic process for the directed *meta*-alkylation of arenes (Scheme 19).^136^ Activated esters are used as starting materials, in which a SET from the excited iridium photocatalyst results in homolytic N–O bond cleavage to generate a nitrogen-based radical. This is then capable of undergoing a 1,5-hydrogen atom transfer (1,5-HAT) to form a carbon-based radical, which undergoes addition at the C–H bond *para* to the ruthenium on metallacycle arene. The final product was a *meta*-functionalized arene, formed through two separate and distinct distal C–H activation procedures. Using this method, an amide derivative of the steroidal molecule lithocholic acid was successfully coupled at the *meta*-position of a 2-phenylpyridine molecule, in addition to a range of simpler substrates.

**Scheme 20:**
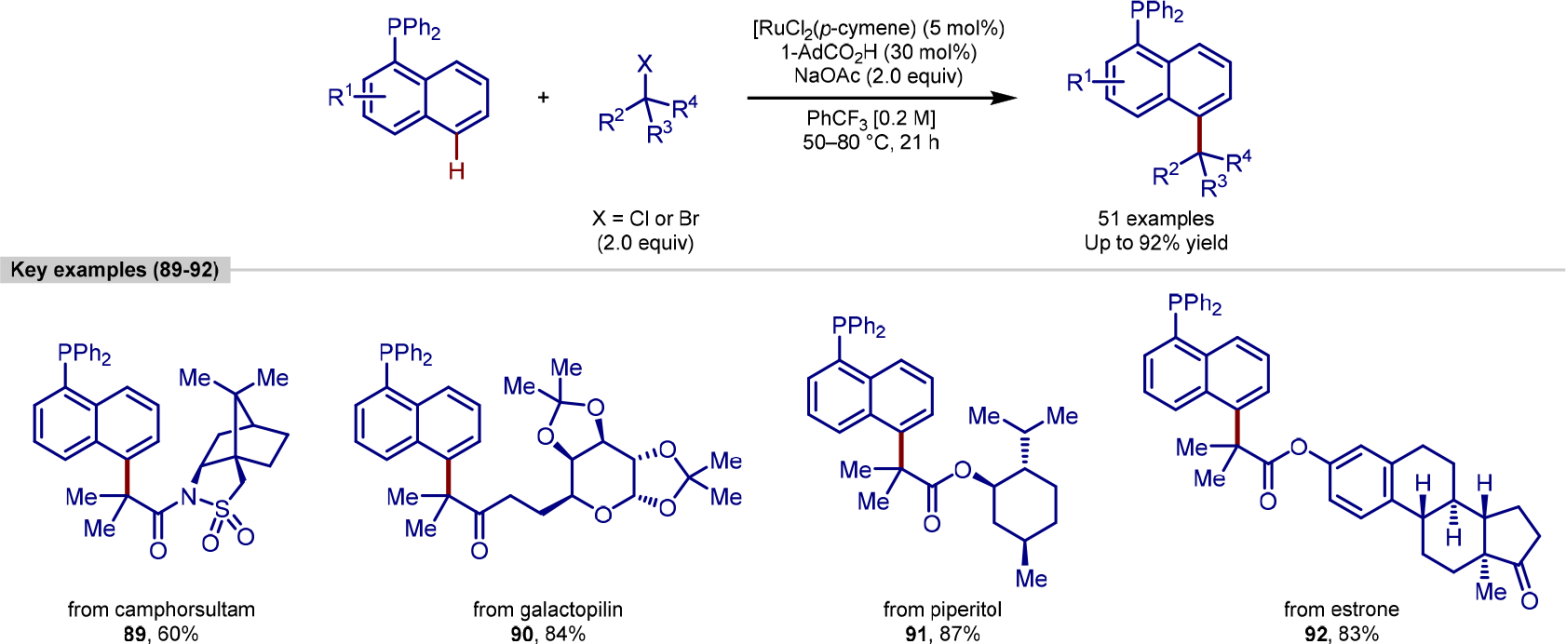
Phosphine-Directed Remote C-5 Alkylation of Naphthalenes

Remote functionalization using the σ-activation method is not only limited to phenyl rings, and many groups have worked on the functionalization of naphthalene compounds. Naphthalene compounds bearing a directing group in the C-1 position have shown reactivity at the proximal C-2 and C-8 positions, in addition to the distal C-4, C-6, and C-7 sites, but had not been functionalized at the furthest C-5 position.^137−139^ In an analogous system to those described above, a recent reaction reported utilizes a C-1 substituted naphthalene with secondary and tertiary α-carbonyl alkyl bromides as alkylating agents, which are proposed to undergo SET reduction with the ruthenium catalyst to generate the carbon-based radical, which then undergoes addition at the C-5 position *para* to the ruthenium–carbon bond (Scheme 20).^139^

The phosphine-directing group was shown to be crucial for the formation of the initial metallacycle, and for activation of the C-5 position through its inductive effect. This method was also capable of the modification of complex molecules using an amide or ester linkage. Through this route, C-5 functionalization of naphthalene was achieved in high yields, with the additions of derivatives of tocopherol, camphorsultam, galactolipin, piperitol, cholesterol and estrone. Examples of ruthenium-catalyzed direct alkylation reactions have generally used nitrogen-based directing groups and thus this example represents an important and valuable strategy that can allow the direct formation of phosphine ligand libraries from unfunctionalized precursors.

**Scheme 21:**
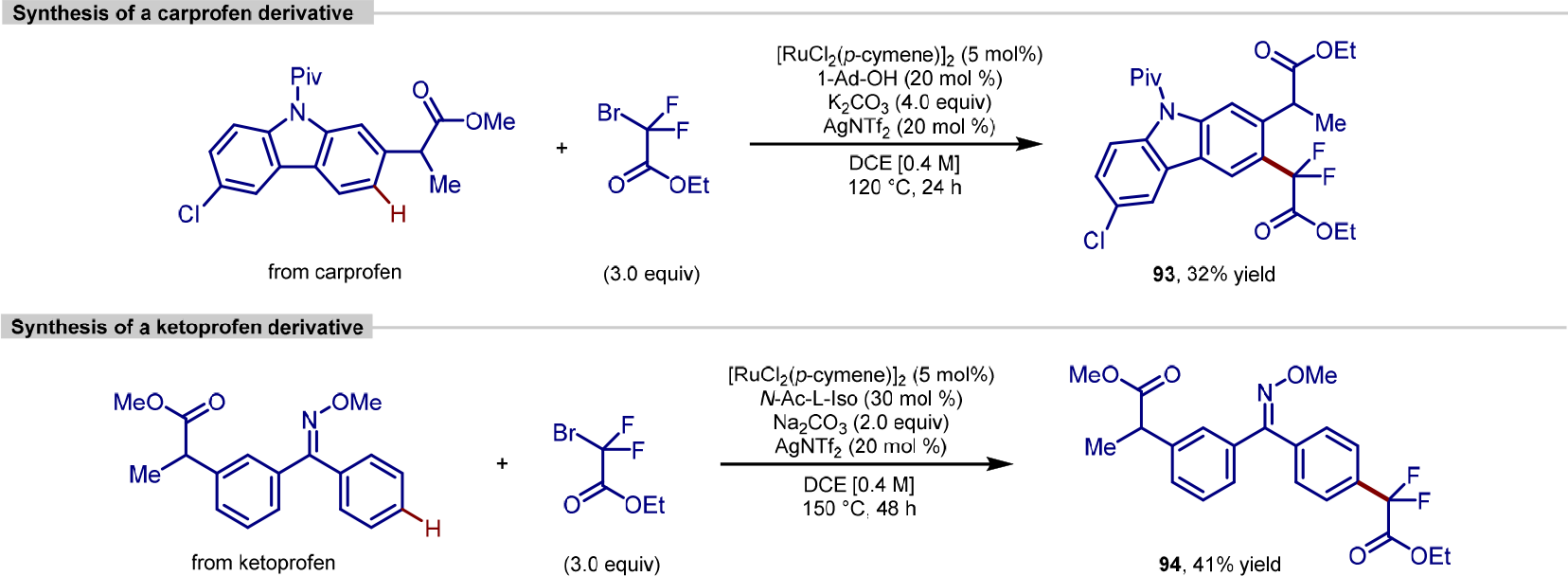
Ruthenium-Catalyzed *para*-C–H Difluoroalkylation of Carprofen and Ketoprofen Derivatives

In addition to *meta*-functionalization, some substrates are capable of remote alkylation in the *para*-position through the same σ-activation type pathway (Scheme 21). In 2018, Zhao reported the ruthenium-catalyzed *para*-C–H difluoromethylation of anilides, in a mechanism that is proposed to be similar to that of the *meta*-functionalization reactions.^140^ The generation of these difluoroalkylated products has grown in interest in previous years with applications growing within the pharmaceutical and agrochemical industries and the importance of the difluoromethyl group has been highlighted in a recent perspective by Gouverneur and co-workers.^141^ For Zhao’s work in this area, electronic effects appeared to play a strong role on the overall selectivity obtained, and hence directing group choice was extremely important. Here, anilide directing groups give rise to the *para*-fluoroalkylated product in good yields, and further work by Zhao utilized ketoximes to give the same selectivity.^142^ In both cases, this methodology was applied to the synthesis of a drug derivative, furnishing carprofen and ketoprofen derivatives in moderate yields. For both procedures, the conditions were not compatible with free carboxylic acids and thus the starting materials had to be converted to the analogous methyl esters before transformation could occur.

**Scheme 22:**
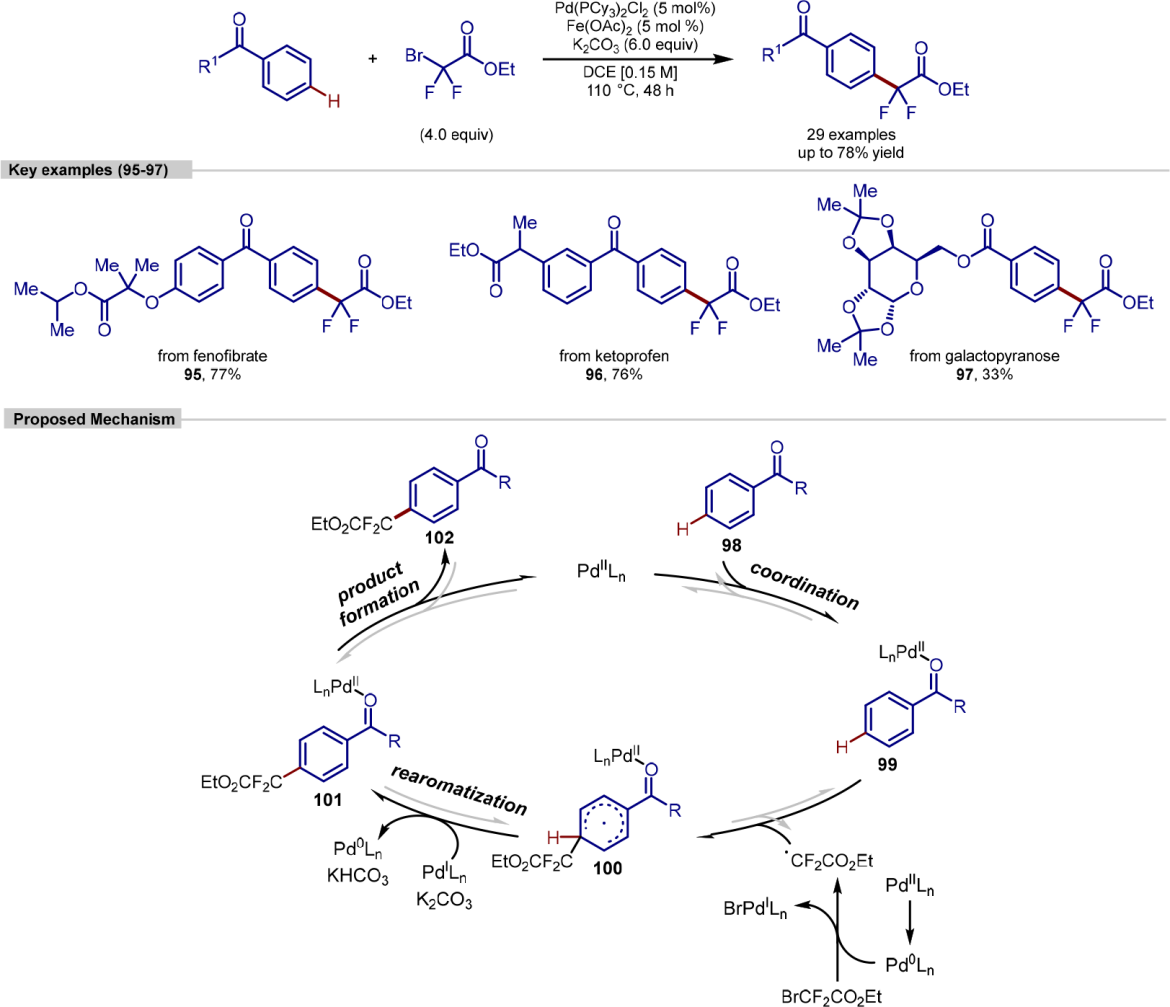
Palladium-Catalyzed *para*-C–H Difluoroalkylation

Difluoromethylations were also demonstrated by Xu to be possible using aromatic carbonyls and palladium catalysis (Scheme 22).^143^ This strategy was demonstrated to be useful in the late-stage functionalization of some natural products and drug molecules, appending difluoroalkyl groups to ketoprofen, fenofibrate, octabenzone, 1-isochromanone, and galactopyranose. Transformations occurred using a large excess of the difluoromethylating reagent (4 equiv) and base (6 equiv) at 110 °C. Similar to previous examples, the reaction could not be performed with unprotected carboxylic acids and these had to be protected as the corresponding methyl esters prior to reaction. The authors further demonstrated the utility of this methodology by further transforming the attached difluoromethyl handle to several different functional groups including a CF_3_ group.

The authors proposed a reaction mechanism based on several varied mechanistic studies (Scheme 22, lower). It was proposed that the palladium catalyst activates the carbonyl via coordination, rather than cyclopalladation, forming intermediate **99**. A radical formed via initiation of the fluoroalkyl halide then reacts with this activated species adding at the *para*-position, which likely occurs under steric control. These form intermediate **100** which can then undergo deprotonation and rearomatization giving the palladium-coordinated intermediate **101**, which then liberates the final product. Cyclopalladation *via ortho-*C–H activation was ruled out as a possibility as no H/D scrambling was observed at this position when *d*_5_-acetophenone (fully C(sp^2^)-D) was used as a substrate in the presence of H_2_O. Further evidence for this was the successful transformation of 2,6-difluoro acetophenone, where the both sites *ortho* to the directing group were substituted, in 45% yield.

**Scheme 23:**
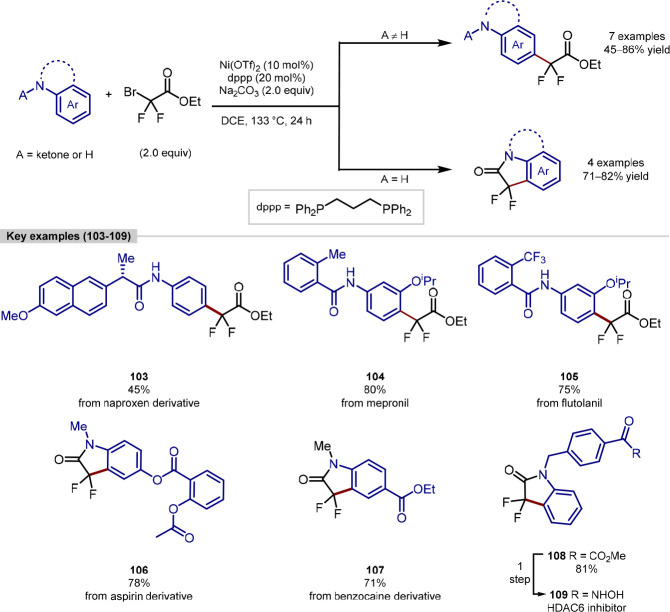
Gou’s Nickel-Catalyzed Difluoromethylation of Aromatic Amines Using Ethyl Bromodifluoroacetate

More recently, in 2022 Gou presented the divergent regioselective difluoromethylation of aromatic amines *via* nickel catalysis (Scheme 23).^144^ A *para*-selective difluoromethylation could be achieved using a coordinating group tethered to the amine such as an amide carbonate and was shown to be compatible with seven different biologically relevant structures in moderate to good yields of 45–86%. The reaction showed exclusive regioselectivity and was able to be performed on a gram scale for simple and less functionally diverse substrates with only minimal effect on reaction efficiency. It was found that the bidentate phosphine ligand was essential for the reaction to occur after significant effort with monodentate phosphine ligands failed to give the desired product. The authors also showed that the reaction conditions could be used to construct 3,3-difluoro-2-oxindole rings, such as products **106**–**108**, which has been demonstrated to be an important structure within the synthetic and medicinal community.^145,146^ This scaffold could be prepared with several molecules containing drug fragments under the conditions with four given examples of this affording product in good yields of 71–82%. They further demonstrated the utility of this through the synthesis of a HDAC6 inhibitor **109** in just 2 steps from commercially available materials.

### 2.3 Rhodium C–H Insertion Strategies

A directing-group strategy can be a reliable method for C–H bond functionalization reactions; however, often these groups require postfunctionalization and so extra synthetic steps are necessary for their removal, in addition to their installation. Consequently, the development of C–H activation methodologies that can achieve predictable reactivity and selectivity through alternative methods such as catalyst and/or reagent control are highly desirable.^147^ One method for this is through the rhodium-catalyzed reactions of donor/acceptor carbenes, in which site selectivity is governed by a balance of steric and electronic effects and typically favors functionalization at secondary and tertiary alkyl C–H bonds.

**Scheme 24:**
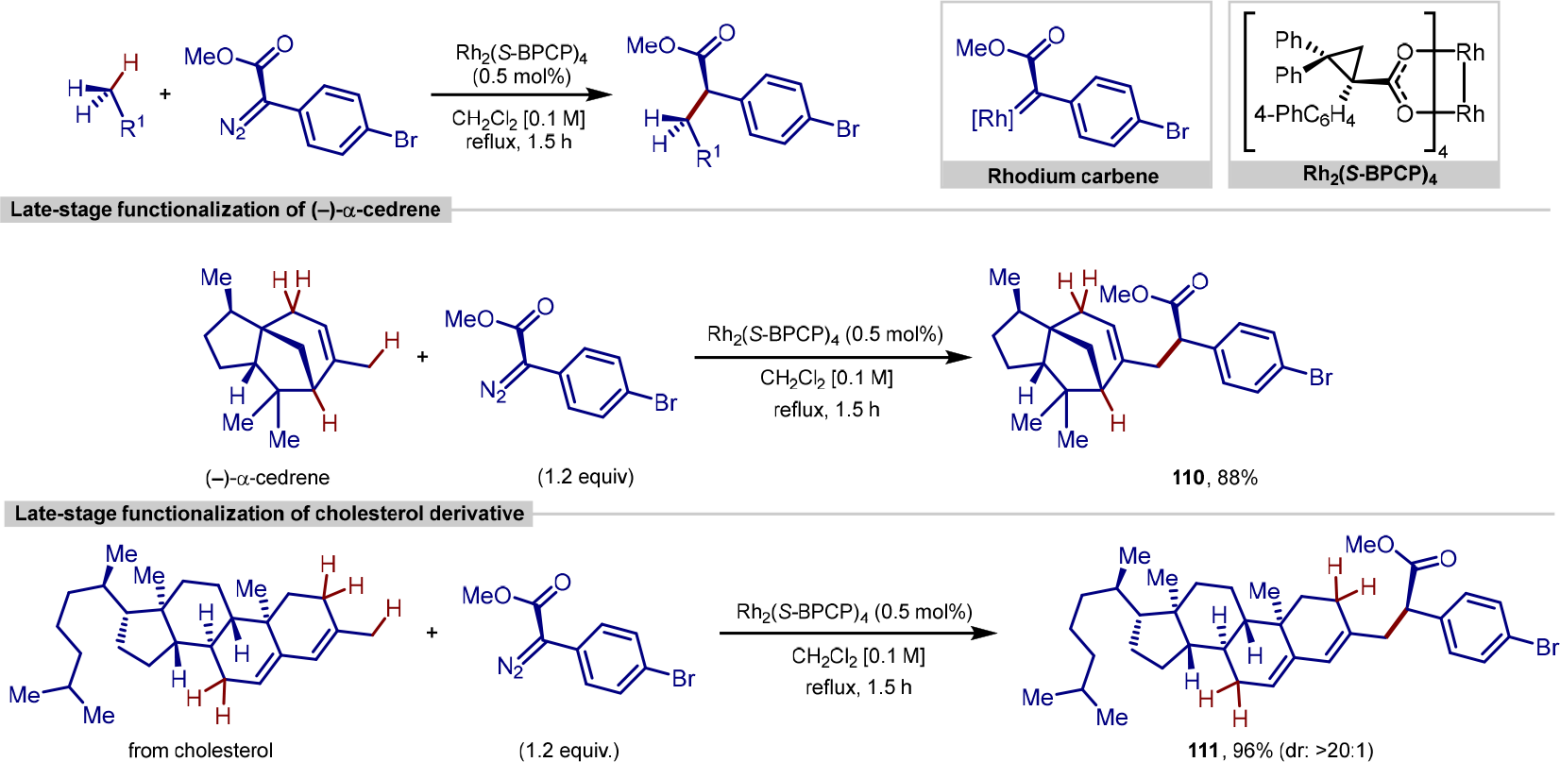
Site-Selective C(sp^3^)–H Bond Functionalization Using Rhodium Donor/Acceptor Carbenes

In 2014, Davies reported a procedure for site selective C–H bond functionalization with these donor/acceptor carbenes, in which the very bulky dirhodium catalysts Rh_2_(*R*-BPCP)_4_ and Rh_2_(*S*-BPCP)_4_ were responsible for a switch in site selectivity, favoring formal alkylation at activated primary C–H bonds, in contrast to results obtained when using Rh_2_(*R*-DOSP)_4_ as a catalyst (Scheme 24).^148^ Intramolecular competition experiments between primary and secondary or tertiary benzylic C–H bonds in benzylic, allylic, and ethereal systems all showed a strong preference for functionalization at the primary C–H bond. With the exception of C–H functionalization adjacent to oxygen atoms, the enantioselectivity of the procedure was also very high.

To further showcase the predictable site selectivity of this procedure, the late-stage functionalization of two molecules containing multiple possible C–H bonds for functionalization was carried out (Scheme 24). When (−)-α-cedrene, a molecule that contains primary, secondary, and tertiary allylic C–H bonds, was subjected to the reaction conditions, functionalization occurred exclusively at the primary C–H bond, generating a single diastereomer in an 88% yield. In addition, a steroid containing three allylic sites showed exclusive reactivity at the primary C–H bond when using both Rh_2_(DOSP)_4_ and Rh_2_(BPCP)_4_ as catalysts. Despite this, the nature of the catalyst still influences the reaction, with a 16:1 mixture of diastereomers achieved with Rh_2_(*S*-DOSP)_4_, which increases to >20:1 with Rh_2_(*S*-BPCP)_4_.

Methods for the C–H functionalization of complex alkaloids are typically hampered by the presence of basic amines, which commonly inhibit catalytic processes, as well as other reactive functional groups. Limited examples of C–H oxidation and amidation of alkaloids are known,^149,150^ although with amidation, the formation of aza-ylide products often dominates. The derivatization of alkaloids using metallocarbenoids has been widely reported in the literature, also proceeding through the formation of aza-ylide species and followed by ring expansion.^151−154^ A metal free carbene approach for the derivatization of brucine **112** has also been reported.^155^

**Scheme 25:**
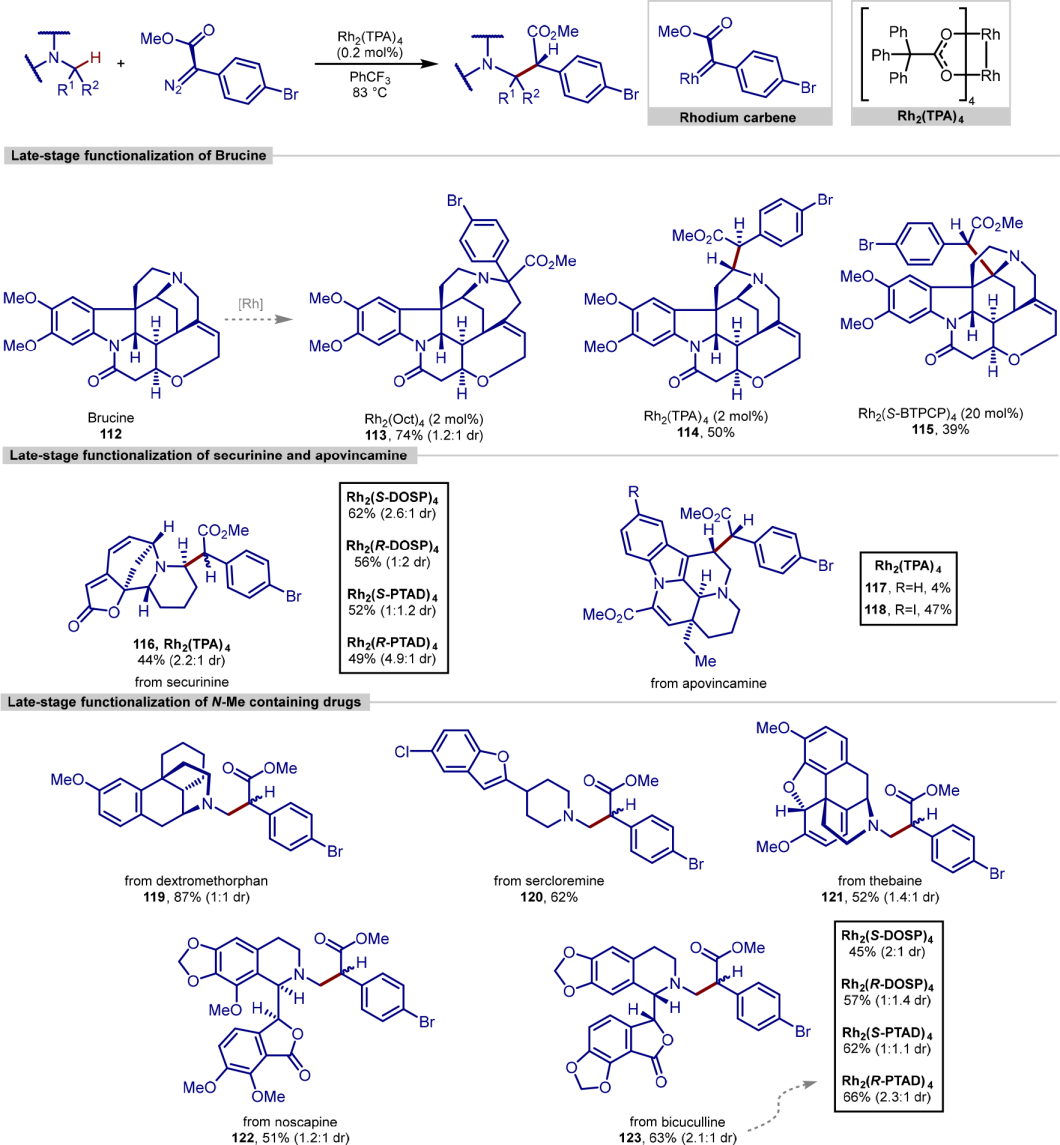
Late-Stage and Site-Selective C(sp^3^)–H Bond Functionalization Using Rhodium Donor/Acceptor Carbenes

Davies in collaboration with Beckwith (Novartis) utilized rhodium-carbenoid chemistry to perform the late-stage functionalization of a number of complex alkaloid natural products and drug molecules.^156^ Starting with brucine **112**, the authors showed that the selection of an appropriate catalyst and reaction conditions was able to influence the preference of the system to effect C–H insertion as opposed to aza-ylide formation (Scheme 25). Testing of a selection of dirhodium catalysts showed that the bulky Rh_2_(TPA)_4_ dirhodium catalyst performed best, promoting formation of C–H insertion product of brucine **112** in a 50% yield as a single diastereomer. Using 20 mol % of very bulky Rh_2_(*S*-BTPCP)_4_ catalyst unexpectedly led to selective functionalization at the tertiary C–H bond adjacent to the amine, in contrast to the usual tendency of bulky catalysts to favor C–H bonds with less steric bulk.

Further application of this method was demonstrated by subjecting securinine, a GABA_A_ antagonist possessing two olefin functional groups in conjugation with a lactone, in addition to a tertiary amine, to the reaction conditions, leading to selective formation of a single C–H insertion product **116**, despite containing four C–H bonds adjacent to the amine. Functionalization of apovincamine, a vasodilator containing an electron-rich indole ring, led to predominantly bis-cyclopropanation and gave product **117**. However, iodine-substituted analogue successfully led to the C–H insertion product **118**, with site-selective functionalization despite the presence of four adjacent methylene and one methine bonds. Finally, a range of *N*-Me containing natural products and drug molecules dextromethorphan, sercloremine, thebaine, noscapine, and bicuculline were successfully functionalized at the *N*-methyl C–H bond to give products **119**, **120**, **121**, **122**, and **123**, despite the presence of multiple alternative activated C–H bonds. As donor–acceptor rhodium carbenoids are typically initiated by a hydride transfer event, the electronic preference would typically be for methine and methylene positions, which are more capable of stabilizing positive charge build-up at the carbon. Despite this, the bulky nature of donor–acceptor carbenoids renders the majority of the most electronically favored sites inaccessible and favors functionalization at the sterically most accessible sites.

### 2.4 C(sp^2^)–H Bond Methylation

**Scheme 26:**
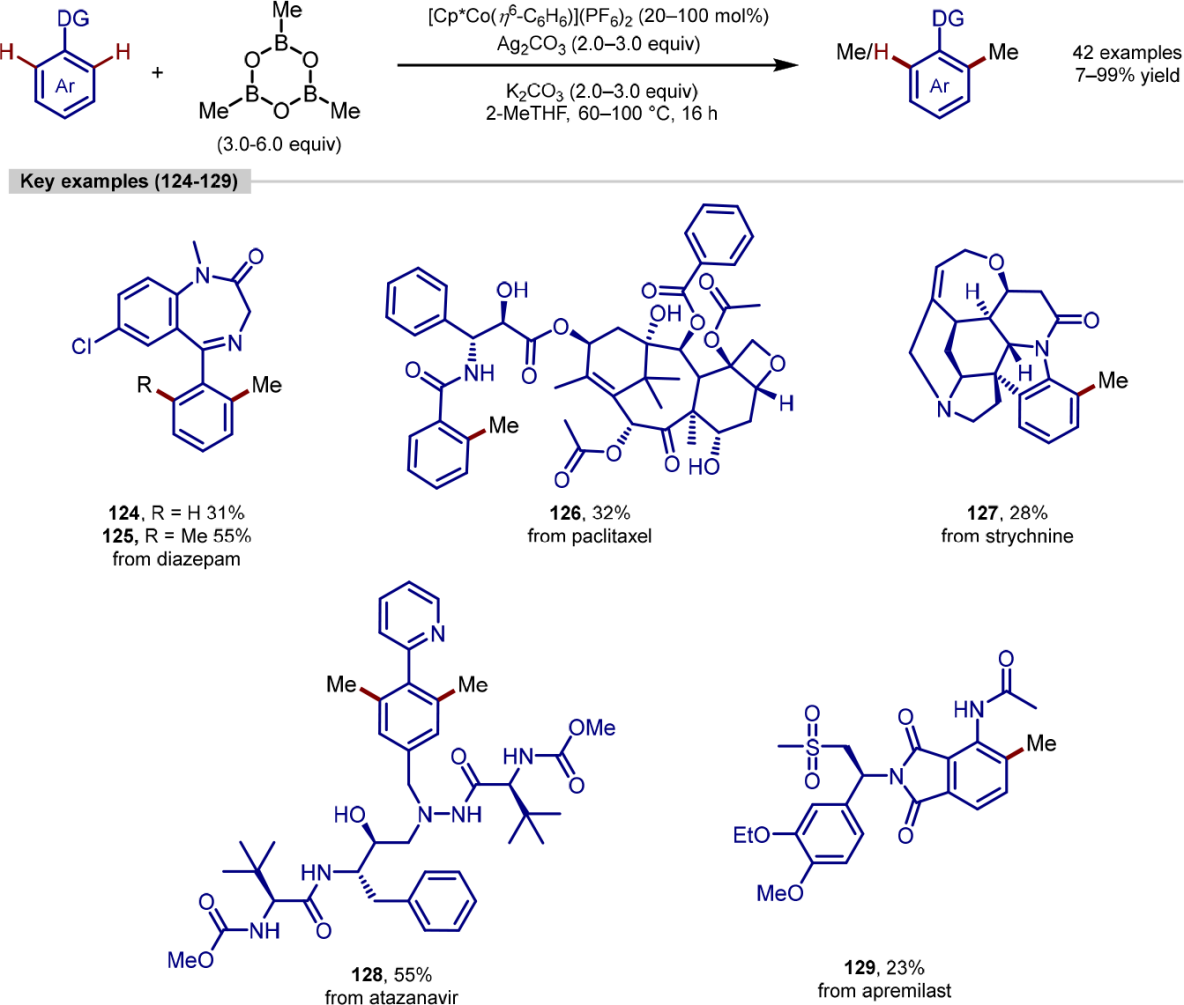
Ackermann’s C(sp^2^)–H Methylation of Biologically Active Molecules Using Cobalt Catalyst [Cp*Co(η^6^-C_6_H_6_)](PF_6_)_2_ and Methylboroxine as Methylating Agent

Ackermann reported the directed C–H methylation of arenes with the Earth-abundant cobalt catalyst [Cp*Co(η^6^-C_6_H_6_)](PF_6_)_2_ and commercially available trimethylboroxine (Scheme 26).^157^ Transformations were achieved at elevated temperatures (60–100 °C) with superstoichiometric quantities of both a potassium carbonate base and a silver carbonate oxidant. The methodology was applied to several simple arenes with generally good yields (24–99% yield, 26 examples) as well as a variety of natural products and biologically active molecules (7–55%, 16 examples). The method proved to be broadly applicable with the transformation successful in the presence of various functional groups including amine, alcohol, amide and ketone groups. A wide range of directing-groups was demonstrated, including several examples of weak directing-groups that are less common such as ketones and aldehydes. Pyridine, amide, ketone, and diazole directing-groups gave the desired products in high yields (up to 99%) while thiazole, pyridazine, oxazolines, and aldehyde directing groups gave products in modest yields of 43–66%. No prefunctionalization or postreaction deprotection was required for the transformation to occur. It is also worth noting that the mass balance of these reactions was high in all cases which is a particularly important factor in LSF due to the typically high value of the starting materials. The utility of the transformation is further highlighted *via* comparison with the longer *de novo* syntheses of the products with late-stage products formed in just one step rather than 3–12 steps. One limitation of the methodology was that for several examples, stoichiometric amounts of the catalyst were required. In addition to this, while predictions could be made regarding levels of mono/bis-methylated product, the methodology gives the synthetic practitioner limited control over the ratio of these with mixtures of mono- and bis-product frequently observed, often requiring separation by preparative HPLC. The authors also investigated the effect that the added methyl group had on the physiochemical properties of the prepared analogues. Interestingly, while the effect of adding a methyl group to simple molecules is generally predictable, it was found that this was not the case for more complex systems. For example, for simple molecules the lipophilicity of the compound generally increases with the addition of a methyl group. However, for the methylation of these more complex drug-like molecules in several cases a decrease in lipophilicity was observed compared with the parent compound.

**Scheme 27:**
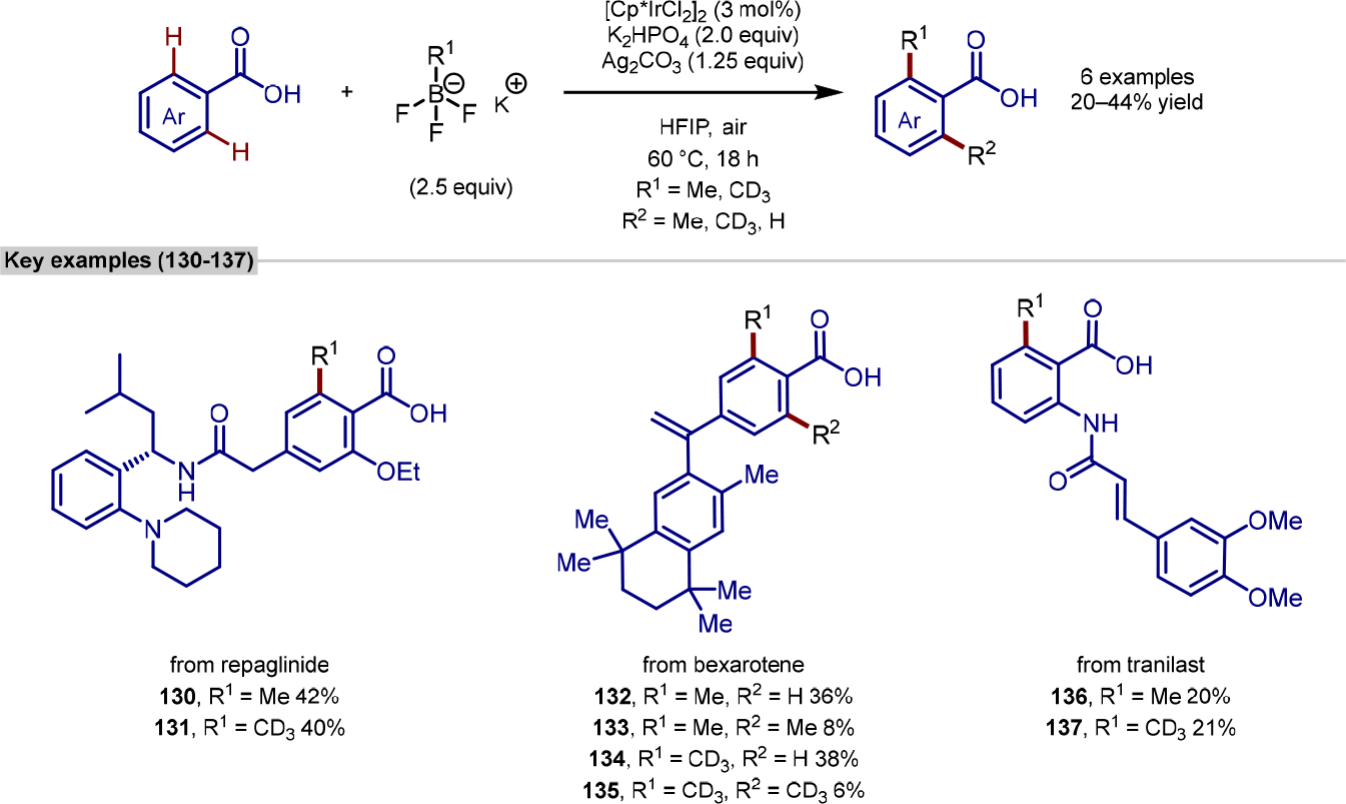
Johansson and Martín-Matute’s Late-Stage C(sp^2^)–H Iridium-Catalyzed Methylation Using Potassium Methyltrifluoroborate as a Methylation Reagent

Following this report, Johansson and Martín-Matute reported the *ortho*-selective C–H methylation of benzoic acids using commercially available reagents and iridium precatalyst [Cp*IrCl_2_]_2_ in 2021 (Scheme 27).^158^ The synthesis of *d*_3_-methylated products was also reported and was the first procedure to do so that demonstrated compatibility with late-stage functionalization. The authors show the methylation of repaglinide giving compound **130** in 42%, which would otherwise require a 16-step *de novo* synthesis, once again demonstrating the utility of late-stage methylation procedures. The authors also show that this methylated analogue *d*_*3*_**-131** has increased metabolic stability highlighting the positive impact of the transformation. Three medicinally relevant compounds were transformed into their methylated and *d*_3_-methylated analogues with moderate yields (20–44%). Electronics appeared to have little effect on the reaction outcome with both electron-donating groups and electron-withdrawing groups well tolerated on the benzoic acid. However, sterically bulky substituents *ortho-* to the benzoic acid were not tolerated, limiting the scope of the methodology. The reaction also benefits from being air- and moisture-tolerant and thus could be performed under ambient conditions, further highlighting potential use cases in high-throughput experimentation. The authors also demonstrate that the HFIP used as a solvent can be distilled and reused in the reaction with minimal loss of reaction efficiency. The reaction also proceeded with complete regioselectivity for the *ortho*-position. One limitation of the reaction is that the reaction formed a mixture of mono- and bis-products where two C–H bonds were available *ortho*- to the directing group which can be difficult to separate due to their similarity.

**Scheme 28:**
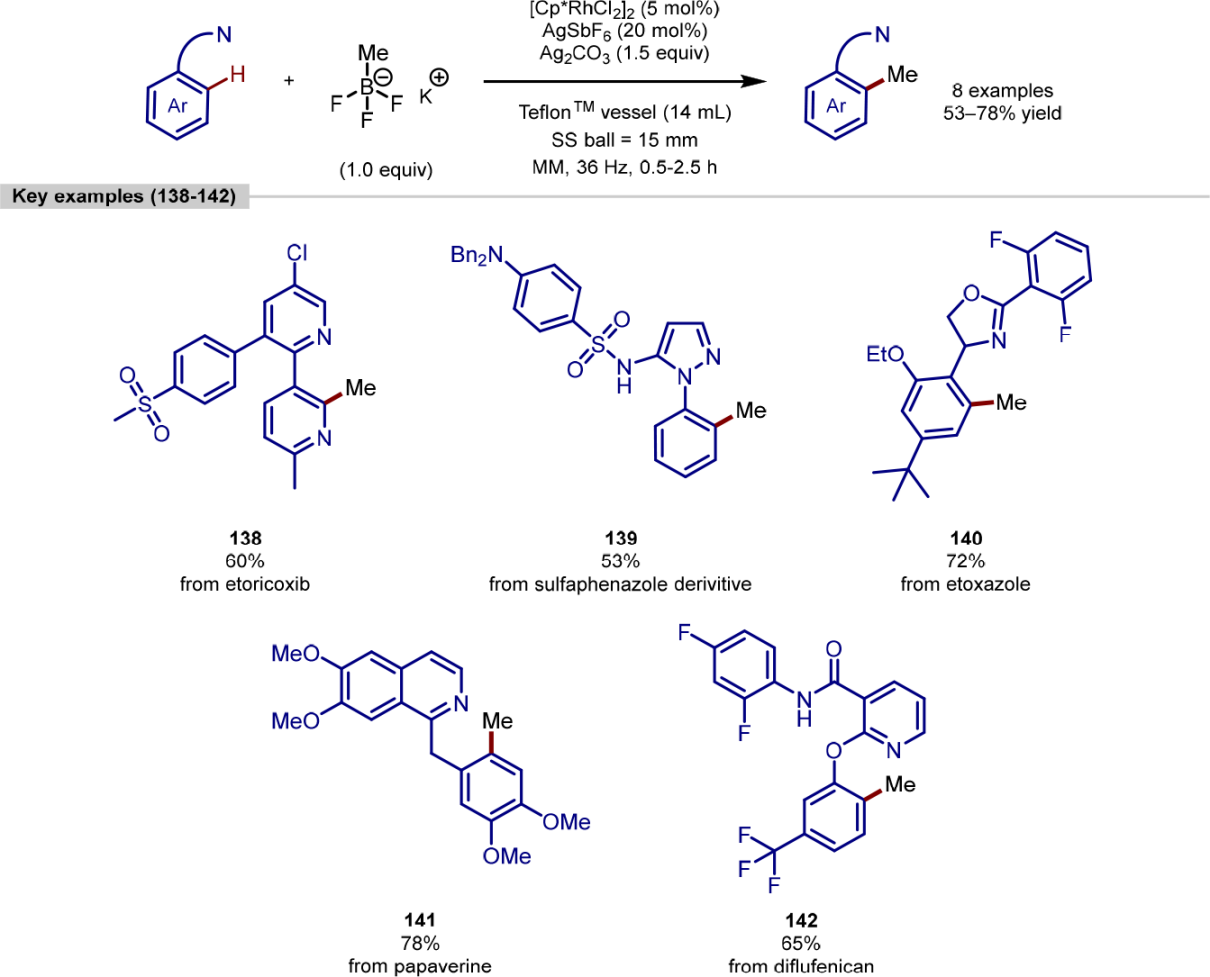
Mechanochemical C(sp^2^)–H Methylation of Directing-Group Containing Arenes Using a Rhodium Catalyst [Cp*RhCl_2_]_2_ and Potassium Methyltrifluoroborate as a Methyl Source

Pilarski demonstrated the first mechanochemical late-stage methylation of several biologically relevant compounds in 2021 using rhodium catalysis (Scheme 28).^159^ The use of mechanochemistry enabled the reaction to be carried out without the use of solvents, which is estimated to contribute to 85% of pharmaceutical waste every year, making this an appealing advantage for late-stage functionalization.^160^ In addition to this, much shorter reaction times were required compared to other methods with only 0.5–2.5 h needed to give the late-stage products. In addition, significantly less of the undesired bis-methylated product was observed (up to 32:1 mono:bis). Simple molecules were successfully converted in up to quantitative yield and the reaction conditions were shown to be compatible with eight biologically active substrates in moderate to good yields of 53–78%. The reaction conditions proved to be compatible with the formation of both 5- and 6-membered postulated rhodacycle intermediates. The formation of these 6-membered intermediates is less thermodynamically favored, and products formed via six-membered metallocycles in C–H functionalization are often given in modest yields.^161−163^ However, the use of mechanochemistry for these examples gave the complex products **138**–**142** in 53–78%. Interestingly, when the AgSbF_6_ was removed from the reaction, products formed via a 6-membered rhodacycle were given in significantly reduced yields. It was proposed that the AgSbF_6_ facilitates transmetalation or reductive elimination when these 6-membered intermediates are involved in the reaction pathway.

**Scheme 29:**
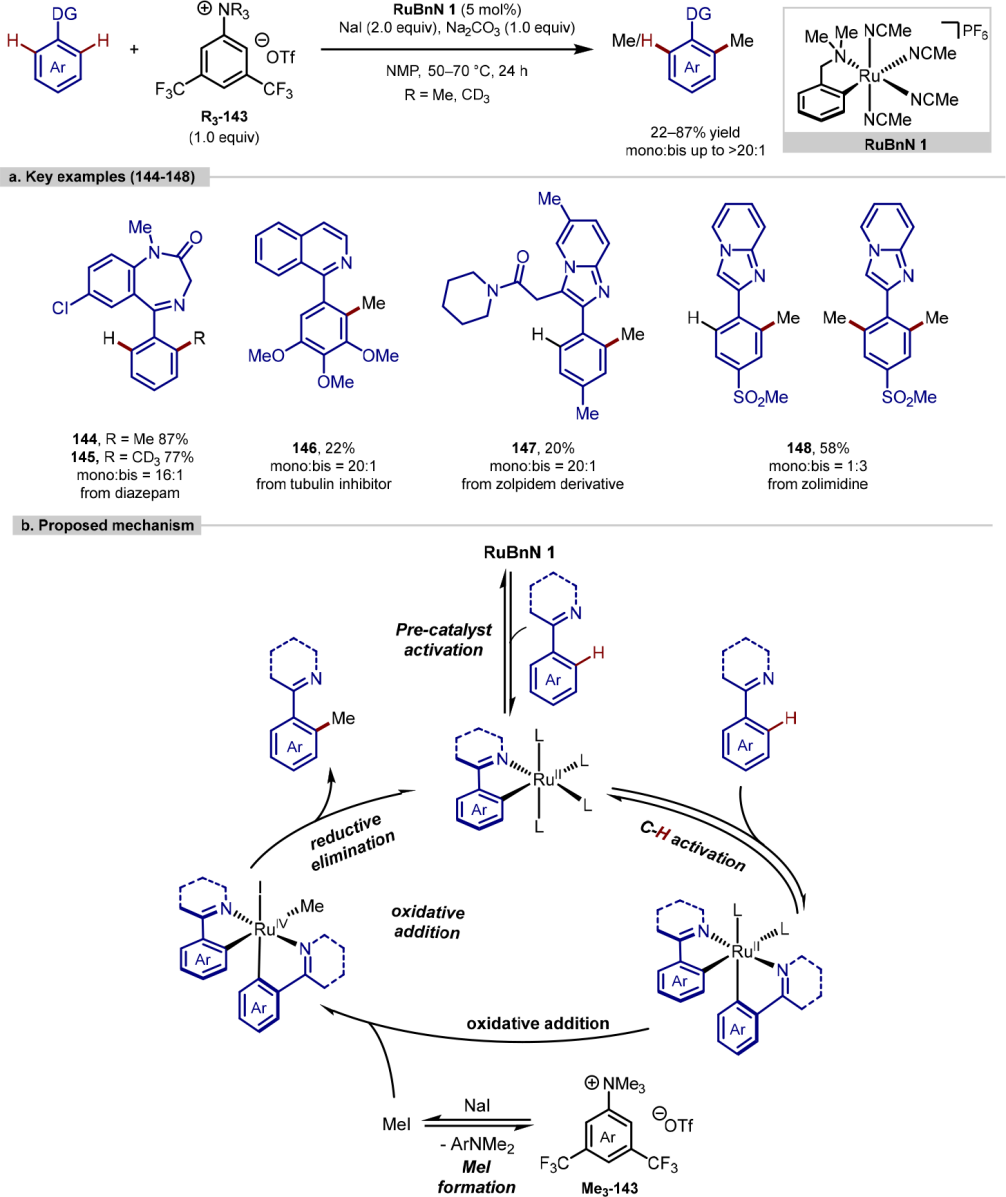
Larrosa’s Methylation of Arenes with Cyclometalated Ruthenium Catalyst and Anilinium Salt as a Methyl Source

Larrosa recently described the ruthenium-catalyzed monoselective C–H methylation and *d*_3_-methylation of arenes, using *N*,*N*,*N*-trimethyl anilinium salts as an easy to handle and stable electrophilic methyl source.^164^ Using 5 mol % of **RuBnN** catalyst **1**, one equiv of Na_2_CO_3_ as a base, two equiv of NaI as an additive and one equivalent of the methylating ammonium salt in NMP at 50–70 °C, high levels of monoselectivity were achieved with 24 examples of methylation and 6 examples of deuteromethylation. This protocol was demonstrated on eight late-stage examples using ammonium salt **Me**_**3**_**-143** bearing two trifluoromethyl groups. Using this more electrophilic salt, the C–H methylation reaction could be performed at lower temperatures allowing the methylation and deuteromethylation of imines, along with the late-stage functionalization of different pharmaceuticals, biologically active molecules and their derivatives (Scheme 29a). Mechanistic studies showed that the slow formation of MeI from the ammonium salt and NaI is the rate-determining step of the reaction (Scheme 29b). This slow formation of MeI *in situ* led to increased monoselectivity as well as the absence of *N*-methylation, which had been observed with the direct use of MeI in the reaction.

### 2.5 C(sp^2^)–H Bond Arylation

**Scheme 30:**
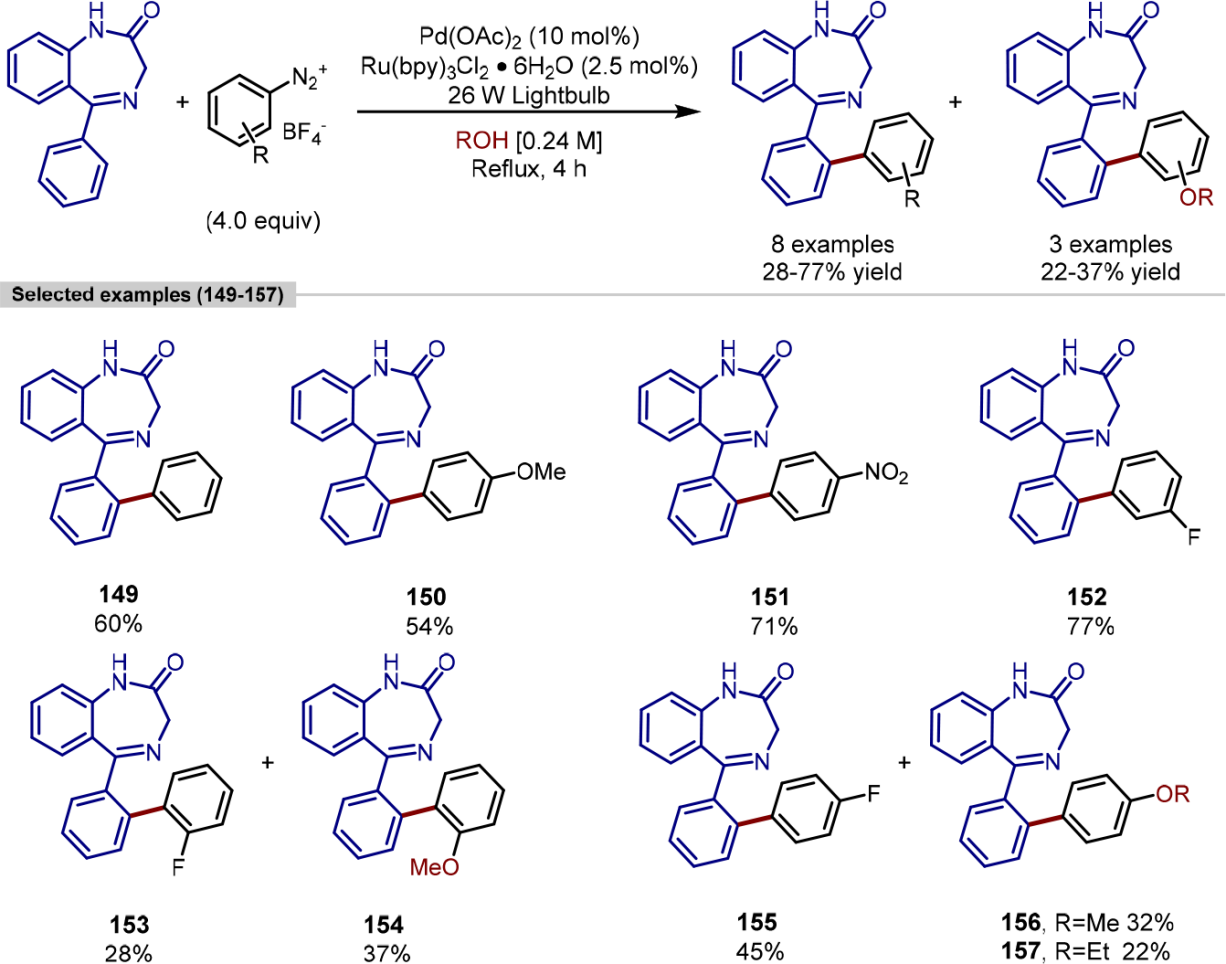
*Ortho*-C(sp^2^)–H Arylation of Benzodiazepines with Aryldiazonium Salts Enabled by a Pd–Ru Dual Catalytic System

Spencer reported a dual catalytic system (palladium catalysis and photoredox catalysis with a ruthenium photosensitizer) that enabled the *ortho*-C(sp^2^)–H arylation of benzodiazepines with a range of aryldiazonium salts (Scheme 30).^165^ The use of 2- and 4-fluorophenyl diazonium salts led to a mixture of arylated products arising from the diazonium salt starting material undergoing nucleophilic aromatic substitution with the solvent (MeOH or EtOH) prior to engagement in catalysis. The γ-aminobutyric acid (GABA) receptor binding ability of the arylated benzodiazepine analogues generated via this synthetic approach was evaluated to determine any changes in biological activity conferred by the introduction of substituted phenyl rings. However, the new benzodiazepine analogues did not display superior binding affinities in the biological assay compared to the controls, nordazepam and diazepam. The best analogue, **149**, was 6-fold less efficient at binding the GABA receptor.

**Scheme 31:**
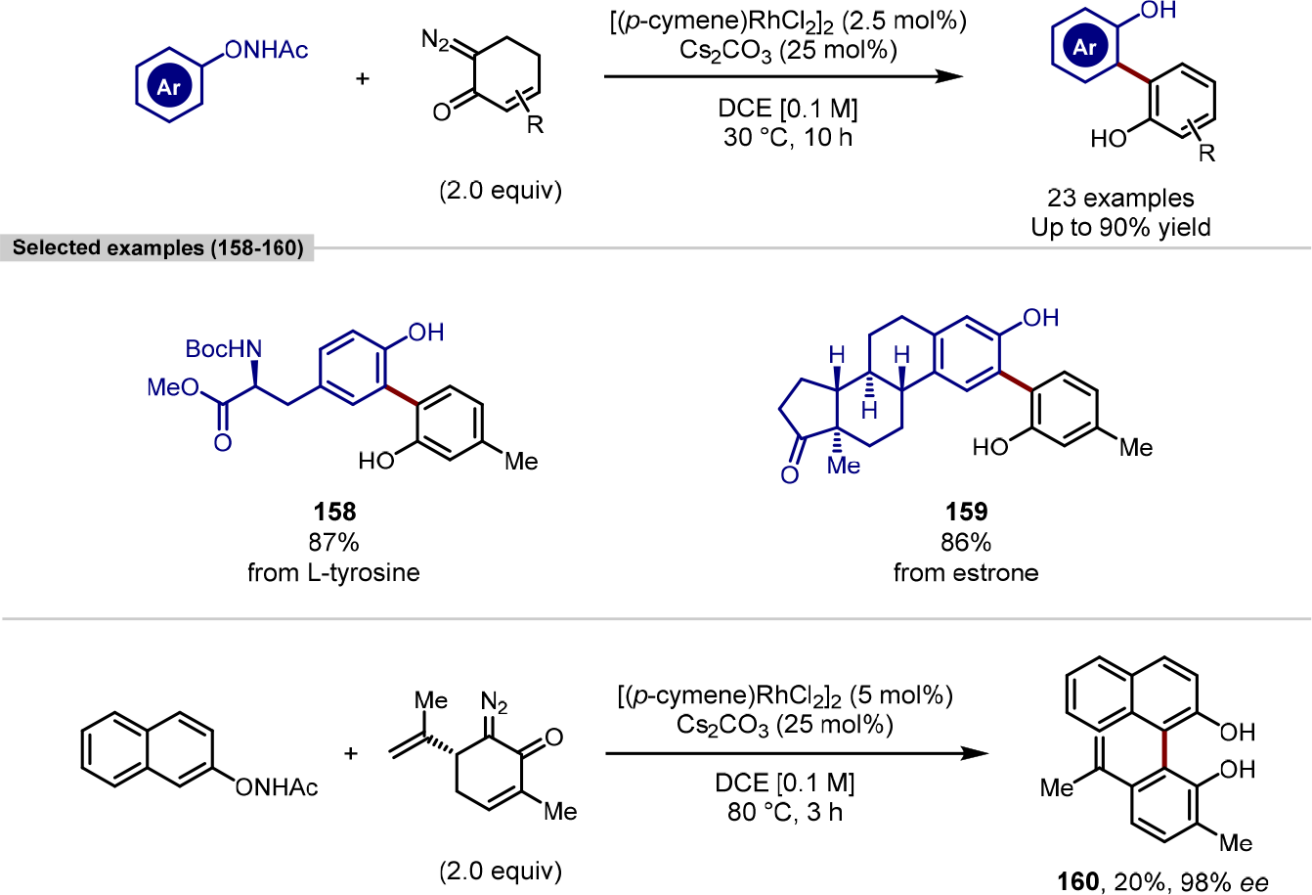
A Redox-Neutral Rh-Catalyzed *ortho*-C(sp^2^)–H Arylation between *N*-Aryloxyacetamides with 6-Diazo-2-Cyclohexenones for the Synthesis of 2,2′-Biphenols

The 2,2′-biphenol motif is commonly found in natural products that exhibit atropisomerism, with this biaryl axis often being the source of axial chirality.^166^ While the most efficient route to accessing these compounds is through the dehydrogenative cross-coupling of phenols, this is challenging when discrete phenol coupling partners are used, since the level of homodimerization versus cross-coupling needs to be controlled. C(sp^2^)–H functionalization approaches in which one phenol partner, or precursor, is prefunctionalized to avoid homodimerization are therefore valuable in the synthesis of these biologically relevant biaryl fragments, albeit with reduced atom economy. In this context, Liu and Hu disclosed a redox-neutral *ortho*-C(sp^2^)–H arylation of *N*-aryloxyacetamides using 6-diazo-2-cyclohexenones as coupling partners, which were oxidized to a phenol unit during catalysis (Scheme 31).^167^ Key to achieving a redox-neutral manifold was the use of the *N*-aryloxyacetamide functional group that directs cyclo-rhodation to the *ortho*-C(sp^2^)–H bond where it is subsequently oxidized, regenerating rhodium(III), thereby avoiding the use of stoichiometric external oxidants.^168^ Complementing a broad substrate scope was the derivatization of l-tyrosine and estrone. A highly atroposelective variant of the reaction was achieved using *N*-(naphthalen-2-yloxy)acetamide and an α-diazo derivative of (*R*)-carvone. The C(sp^2^)–H arylation was rendered atroposelective through a center-to-axial chirality transfer mechanism facilitated by the latter coupling partner, however the absolute stereochemistry of the biaryl axis was not assigned. Future studies could investigate the atroposelective *ortho*-2,2′-biphenol synthesis in a late-stage manner, with the alkene serving as a functional handle for further synthetic transformations.

**Scheme 32:**
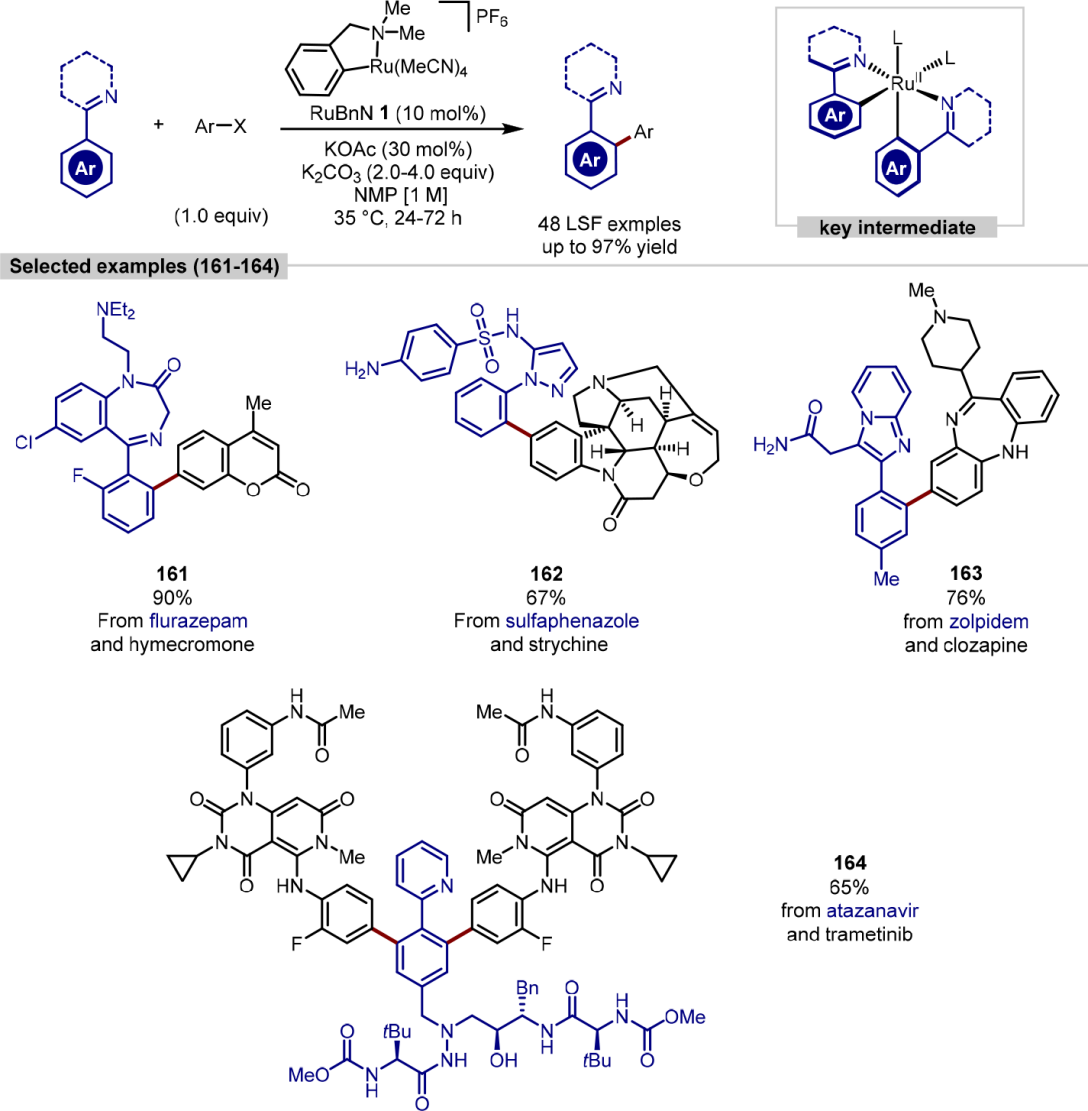
RuBnN **1**, a Novel Ru^II^ Precatalyst, Enables the Room-Temperature Arylation of Pharmaceuticals and “Drug–Drug” Coupling

Larrosa has demonstrated how mechanistic studies, in which the kinetics of the *N*-directed ruthenium-catalyzed *ortho*-C(sp^2^)–H arylation were investigated, can assist with the design of a new precatalyst able to tolerate various Lewis basic functionalities in substrates, thereby enabling the late-stage functionalization of complex molecules.^79^ The kinetics studies uncovered that the *p*-cymene ligand, present in the commonly used precatalyst [Ru(*p*-cymene)Cl_2_]_2_, plays an inhibitory role in catalysis as dissociation is required before catalytically active species can be accessed, with a previously unknown active intermediate not able to form until this step occurs. Elevated reaction temperatures are typically required for this dissociation to occur, yet the remainder of the cycle proceeds under mild conditions. These key mechanistic insights allowed for the design and use of an η^6^-arene-free precatalyst, RuBnN **1**, that enabled the efficient *ortho*-C(sp^2^)–H arylation of arenes containing *N*-directing groups at close to room temperature (Scheme 32). The tolerance of this new precatalyst toward unprotected Lewis basic functional groups was demonstrated through its broad substrate scope which consisted of the functionalization of *ortho*-tolylpyridine with 34 halide and pseudohalide-containing pharmaceuticals as well as ten examples of pharmaceutical late stage arylation. The robust catalytic procedure was also applied to the coupling of two complex pharmaceuticals to give an overall “drug–drug” coupling, thus highlighting the high utility and tolerance of the method.

**Scheme 33:**
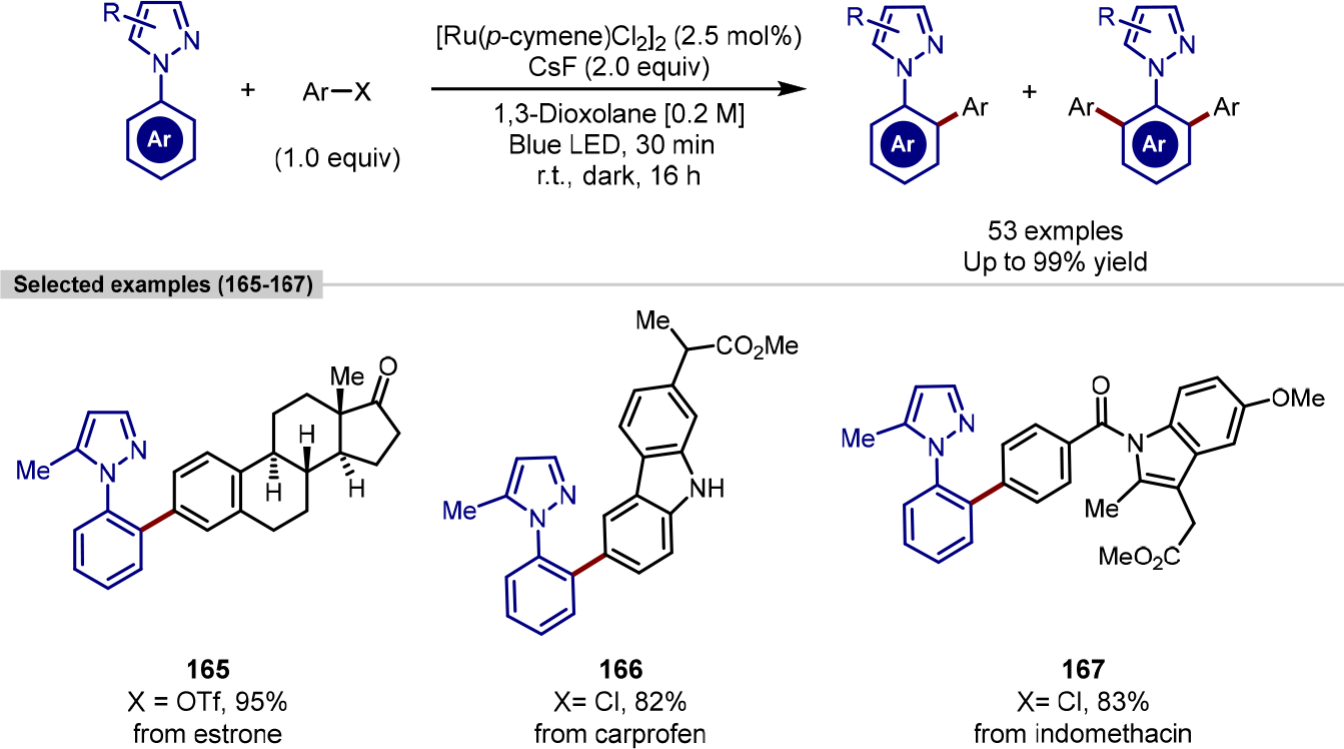
Directed *ortho*-C(sp^2^)–H Arylation under Visible-Light Irradiation Allows for the Functionalization of Biologically Active Molecules Using a Commercially Available Ru^II^ Precatalyst under Mild Conditions

Since Larrosa proposed a new mechanism for the ruthenium-catalyzed C(sp^2^)–H arylation of *N*-directing group arenes, in which a biscyclometalated Ru^II^ complex was found as a key intermediate, the photoinduced dissociation of *p*-cymene *in situ* has enabled a number of room temperature C(sp^2^)–H arylations using the commercially available [Ru(*p*-cymene)Cl_2_]_2_.^169,170^ Zhang demonstrated that an initial 30 min period of irradiation with 455 nm LEDs could generate a sufficient quantity of arene-free ruthenium(II) in the reaction mixture to affect a room temperature C(sp^2^)–H arylation with [Ru(*p*-cymene)Cl_2_]_2_.^171^ (Pseudo)Halide derivatives of natural products and pharmaceuticals were coupling partners used with a 5-methyl-1-phenylpyrazole substrate to demonstrate applicability of the methodology, with all proceeding in excellent yield (Scheme 33).

**Scheme 34:**
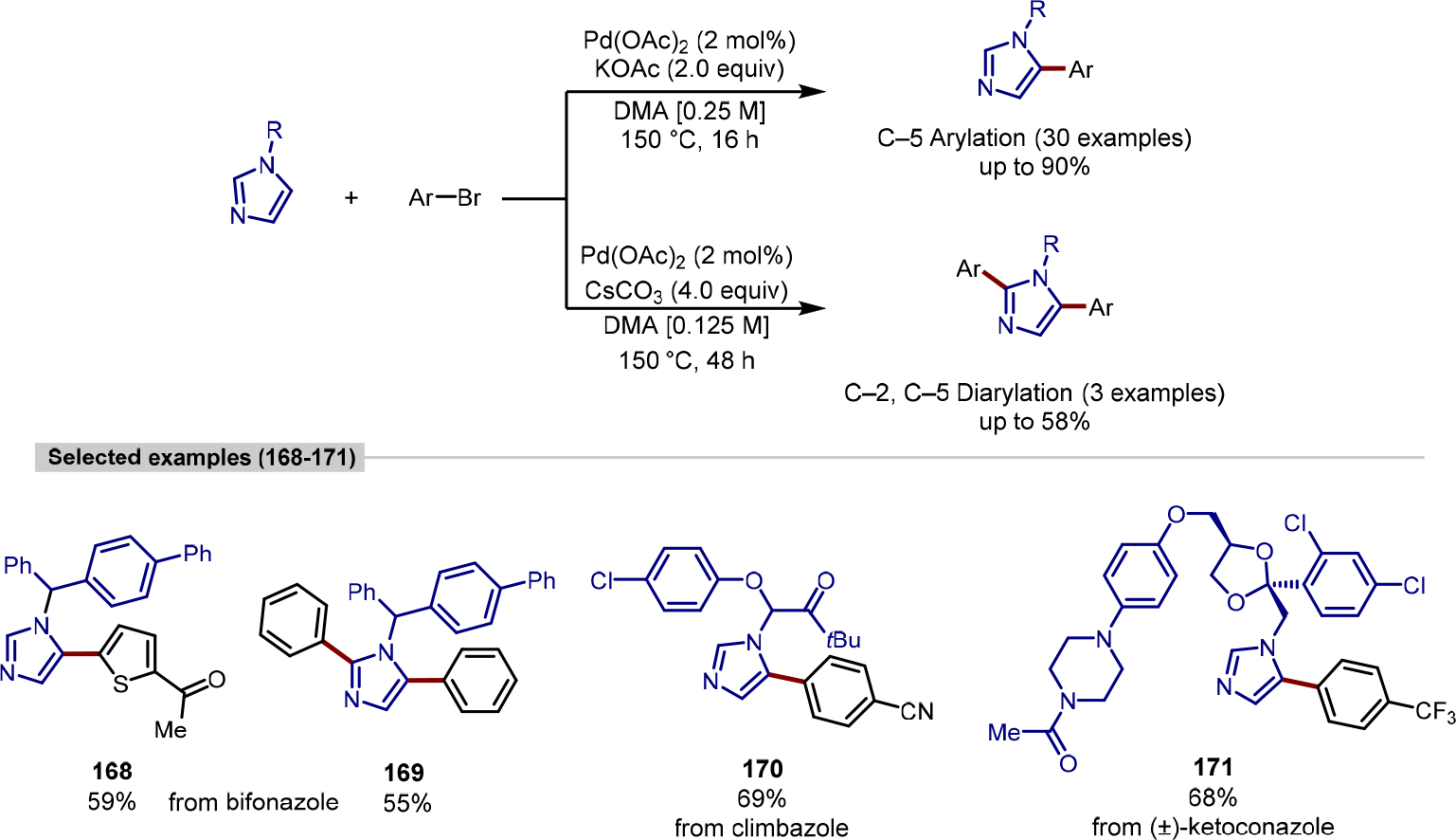
Regioselective C-5 Arylation of Bifonazole, Climbazole, and Ketoconazole with Aryl Bromides

The imidazole heterocycle is within the top 10 most frequently used ring systems in pharmaceuticals^172^ and can undergo C–H activation in the absence of a directing group, although C-2 versus C-5 functionalization needs to be controlled owing to the greater acidity of the C–H bond at C-2.^173,174^ Doucet and Soule reported a strategy for the palladium-catalyzed C(sp^2^)–H arylation of imidazole-containing pharmaceuticals with (hetero)aryl bromide coupling partners, with complete selectivity for arylation at C-5 observed.^175^ Bifonazole, climbazole, and ketoconazole were the pharmaceuticals used to demonstrate applicability of the transformation within a complex and functionally diverse environment (Scheme 34). For bifonazole, it was demonstrated that diarylation (C-2 in addition to C-5) could be achieved when the reaction was run for an additional 32 h in the presence of excess aryl bromide (3 equiv) and Cs_2_CO_3_ in-place of KOAc. C-5 Arylation of the imidazole units within climbazole and ketoconazole proceeded in low to moderate yields, with both substrates containing aryl chloride functionalities that were untouched under the reaction conditions.

**Scheme 35:**
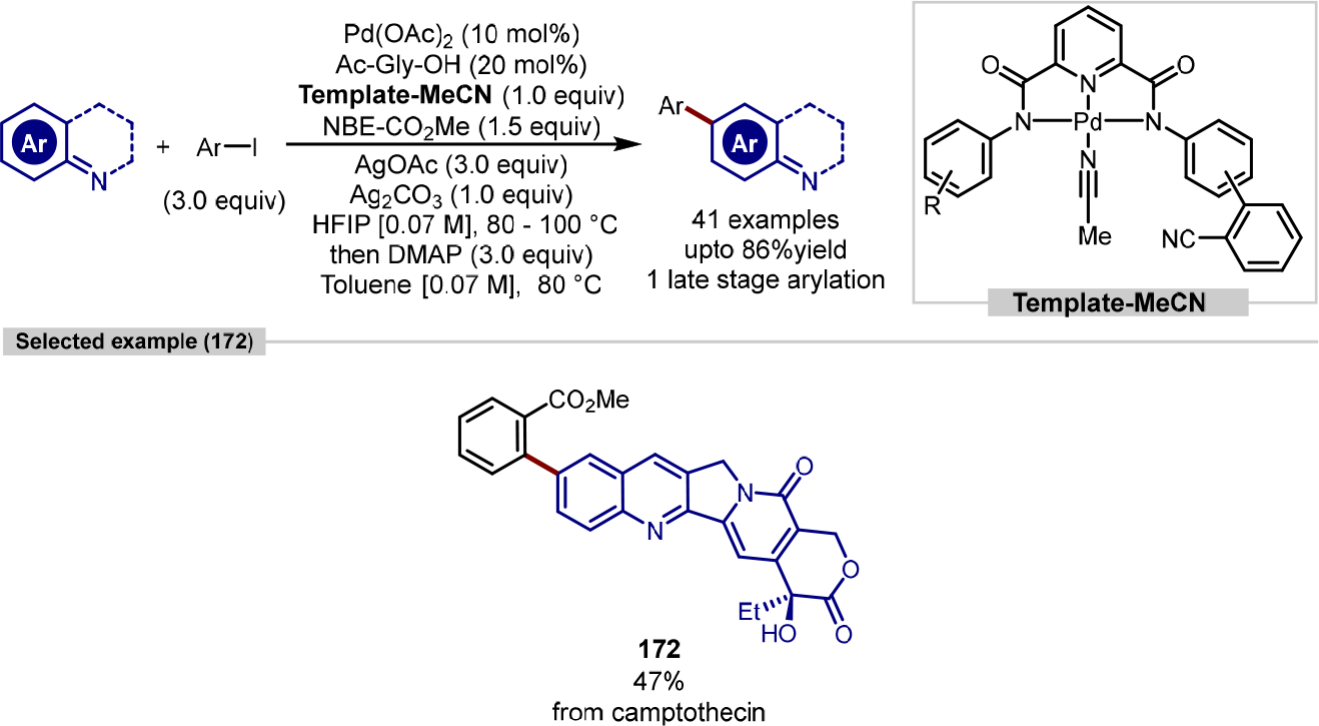
Remote C-6 Arylation of Camptothecin Enabled by a Template-Norbornene Approach

The potential for C–H functionalization to be an enabling technology in expediting the discovery of pharmaceuticals, agrochemicals, and functional materials lies in the ability to efficiently explore chemical space around a lead compound. However, achieving selectivity for C–H bond activation when there is little electronic bias between C–H bonds in a molecule is challenging. While the directing-group approach is a reliable strategy for achieving site-selectivity in C–H activation, remote C–H bond activation remains challenging, particularly for electronically similar C–H bonds.^176^ To achieve the selective C-6 arylation of (iso)quinolines, Yu built on their previous work for the C-5 selective olefination of quinolines,^177^ in which a template-based approach enabled the aforementioned site-selectivity for C–H activation and ensuing olefination. In order to activate the C–H bond at C-6, the template approach was augmented with a norbornene relay. Template-directed C–H activation occurs at C-5 with norbornene acting as a transient mediator migrating the palladium(II) complex over to C-6, thereby activating this C–H bond.^178^ While both the template and norbornene derivative were required in stoichiometric loadings, the exclusive C–6 selective arylation of quinoline and isoquinoline substrates was demonstrated on a broad range of quinoline and isoquinoline systems. With respect to the former, camptothecin served as a late-stage example of quinoline C-6 arylation; the functionalization furnished a camptothecin analogue **172** in moderate yield in the presence of a free hydroxyl group that could potentially compete for binding the palladium center of the template with the quinoline nitrogen atom (Scheme 35).

**Scheme 36:**
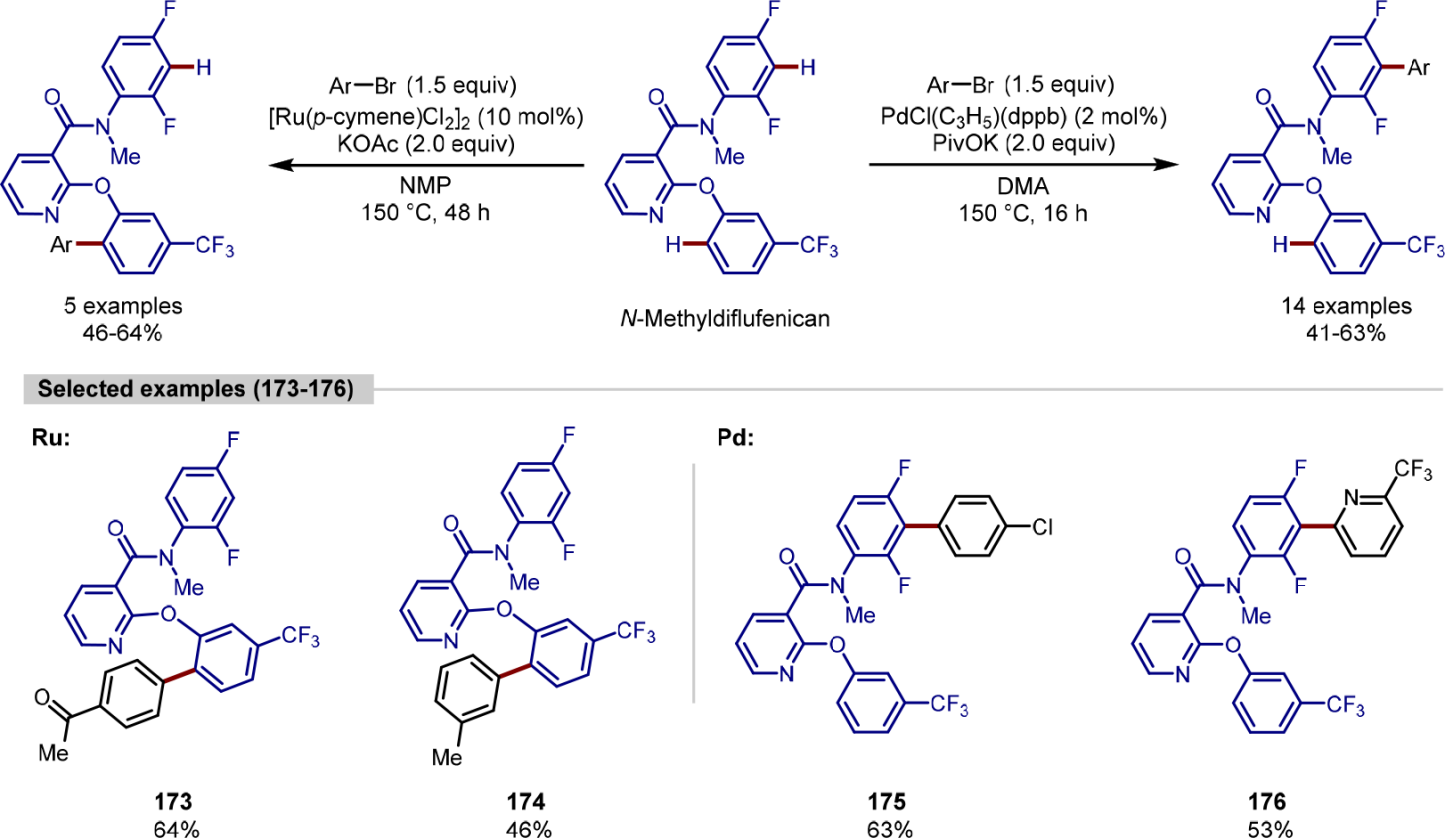
Regiodivergent C(sp^2^)–H Arylation of *N*-Methyldiflufenican with Aryl Bromides and Ru or Pd Precatalysts

Doucet demonstrated how, by choosing the right catalyst, different C–H bonds in *N*-methyldiflufenican can be selectively arylated, thereby generating analogues covering a greater area of chemical space (Scheme 36).^179^ The 2-phenoxypyridine motif within diflufenican enabled LSF using a directed arylation with aryl-bromide electrophiles in combination with a [Ru(*p*-cymene)Cl_2_]_2_ precatalyst (Scheme 36, left). Instead, Pd(OAc)_2_ led to the direct arylation on the most acidic bond in the 1,3-difluorobenzene ring (Scheme 36, right). In both strategies, moderate yields were obtained and a variety of (hetero)aryl bromides were tolerated as coupling partners.

**Scheme 37:**
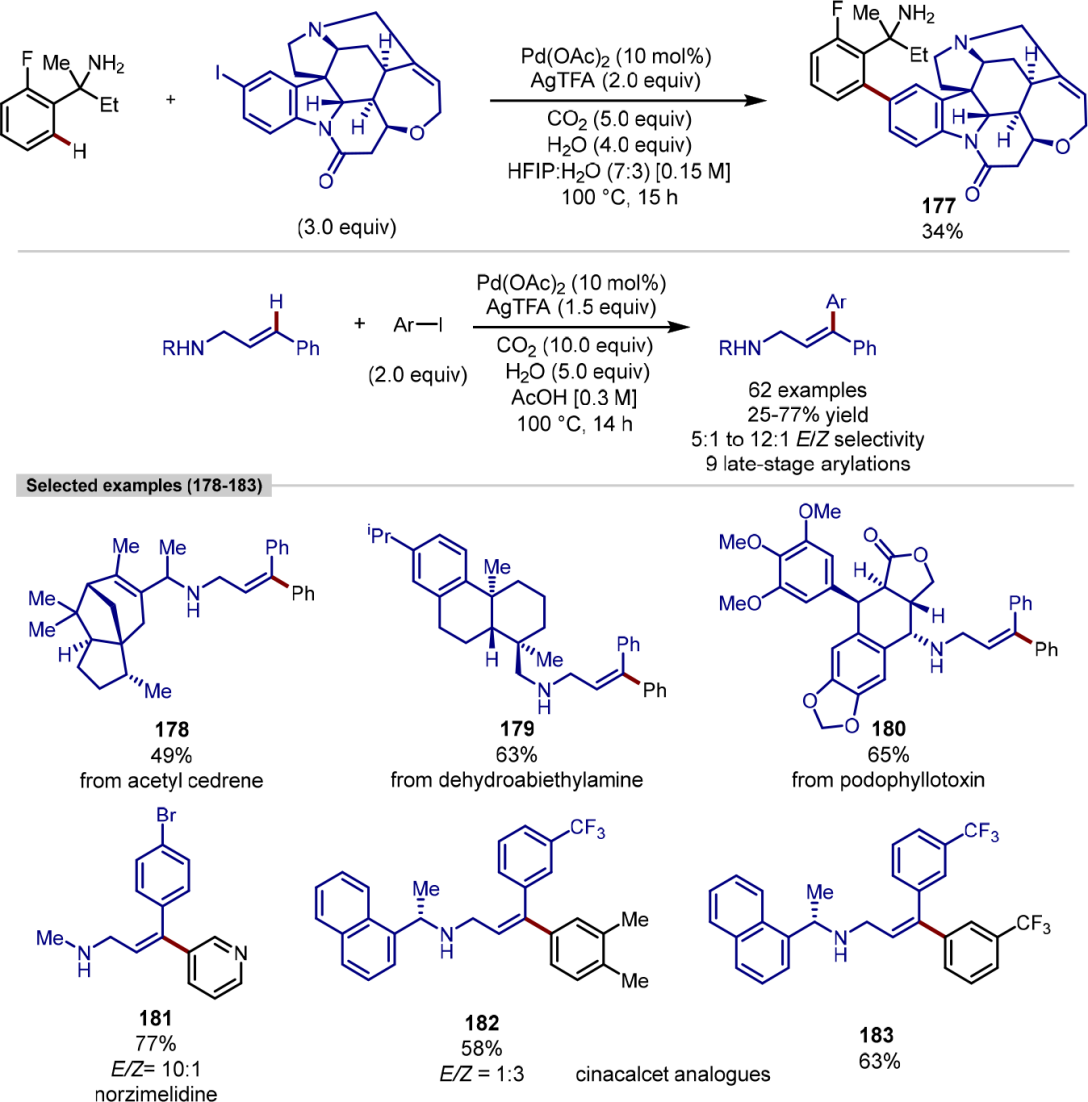
γ-C(sp^2^)–H Arylation of Benzylamines and Allylamines Using an *In Situ* Directing Group Approach with CO_2_

Despite being prevalent motifs in pharmaceuticals, free amines are unfavorable directing groups. This is due to the highly coordinating nature of the nitrogen atom toward the transition-metal catalysts, which can lead to catalytically inactive Bisamine complexes. Amine oxidation can also be detrimental and potentially lead to catalyst inhibition. A solution to these problems is to attenuate the Lewis basicity of the nitrogen atom by installing protecting groups; however, atom and step economies suffer as a result. Young has investigated the use of CO_2_ to generate a carbamate *in situ* that can both reduce amine-Lewis basicity as well as generate a transient directing group that can be used for the Pd-catalyzed γ-C(sp^2^)–H arylation of benzylamines (both primary and secondary)^180^ and γ-C(sp^2^)–H arylation of allylamines.^181^ They demonstrated the utility of these arylation methodologies through the functionalization of several natural products. In both transformations CO_2_ was added as dry ice. For the γ-C(sp^2^)–H arylation of benzylamines, an aryl iodide derived from strychnine was used as the electrophilic coupling partner, furnishing **177** in moderate yield (Scheme 37). A broader scope of late-stage functionalization was reported for the γ-C(sp^2^)–H arylation of allylamines, with cinnamylamine derivatives of various natural products undergoing γ-C(sp^2^)–H arylation in moderate to high yield. For norzimelidine and cinacalet analogues, introduction of a discrete (hetero)aryl ring led to 3,3-diarene products gave with *E*-stereoisomer forming as the major isomer as confirmed by NOESY NMR spectroscopy.

The palladium-catalyzed C-2 arylation of the indole unit within the amino acid tryptophan with aryl iodides has been well-established.^182^ While the approach to amino acid functionalization has been extended to linear peptides by Ackermann in which diaryliodonium salts are used as arylating agents,^183,184^ such compounds suffer from poor metabolic stability, and reduced target affinity owing to the conformational flexibility. Conversely, macrocyclic peptides are of interest due to the conformationally constrained nature of these ring systems, particularly structures in which amino acid side chains are linked, leaving the *N*- and/or *C*-termini untouched since these motifs can comprise key interactions to the target.^185^

**Scheme 38:**
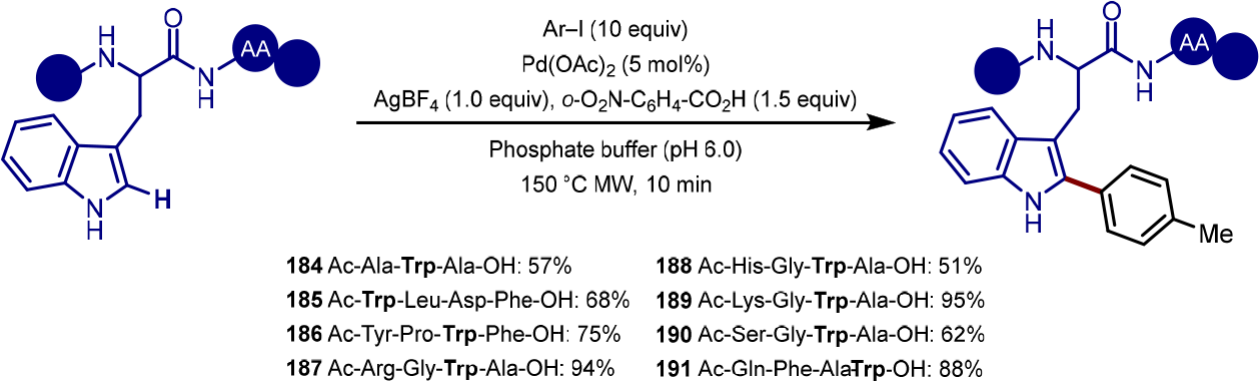
Palladium-Catalyzed C(sp^2^)–H Arylation of the Indole Units of Tryptophan Using Aryl Iodides

The direct C-2 arylation of indoles with aryl iodides under mild conditions was first reported by Larrosa in 2008, with mechanistic studies uncovering the inhibitory role of tertiary phosphines in these transformations.^186^ A subsequent study from the same group demonstrated the reaction to be possible “on water”, making this C–H arylation methodology applicable to water-soluble substrates such as peptides, in addition to avoiding the use of DMF.^187^ Albericio and Lavilla built on this procedure to achieve the chemoselective C-2 arylation of tryptophan residues within tetrapeptide substrates, with a phosphate buffer replacing DMF as the solvent system (Scheme 38).^188^ While only tetrapeptide substrates were investigated, a broad range of amino acid residues were tolerated; notably arginine, tyrosine, histidine and lysine. Furthermore, the carboxylic acid at the *C*-terminus could be left unprotected, generating the opportunity for further peptide couplings following late-stage arylation.

**Scheme 39:**
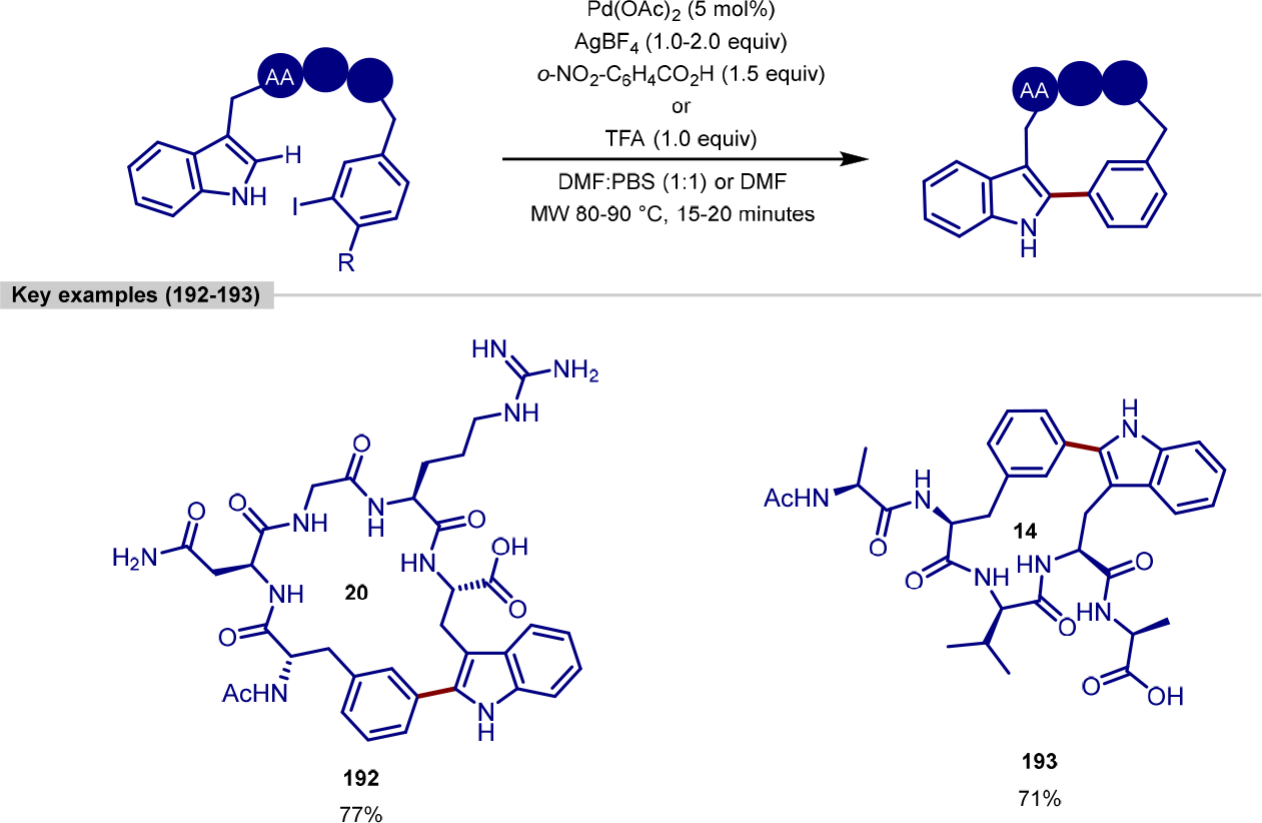
Palladium-Catalyzed C(sp^2^)–H Arylation of the Indole Units Reported by Albericio and Lavilla

Building on their initial report, Albericio and Lavilla then reported a strategy for peptide macrocyclization, achieved *via* the stapling of tryptophan with either *meta*-iodo-phenylalanine or tyrosine residues (Scheme 39).^189^ The scope included a variety of ring sizes (from 14- to 24-membered macrocycles), double C(sp^2^)–H arylation products and cyclodimerized products. Subsequent work from these authors examined the effect of spacer length between tryptophan and the (*ortho*-, *meta*-, or *para*-)iodo-phenylalanine residues on the propensity for the substrate to undergo macrocyclization or cyclodimerization.^190^ Similarly, to the intermolecular C-2 arylation of indole motifs within tetrapeptides, amino acid residues bearing polar chain could be tolerated. One drawback of this protocol for the late-stage stapling of peptides lies in the method of purification. This was achieved using semipreparative RP-HPLC, affording the products in poor isolated yields, even when high conversions were observed.

**Scheme 40:**
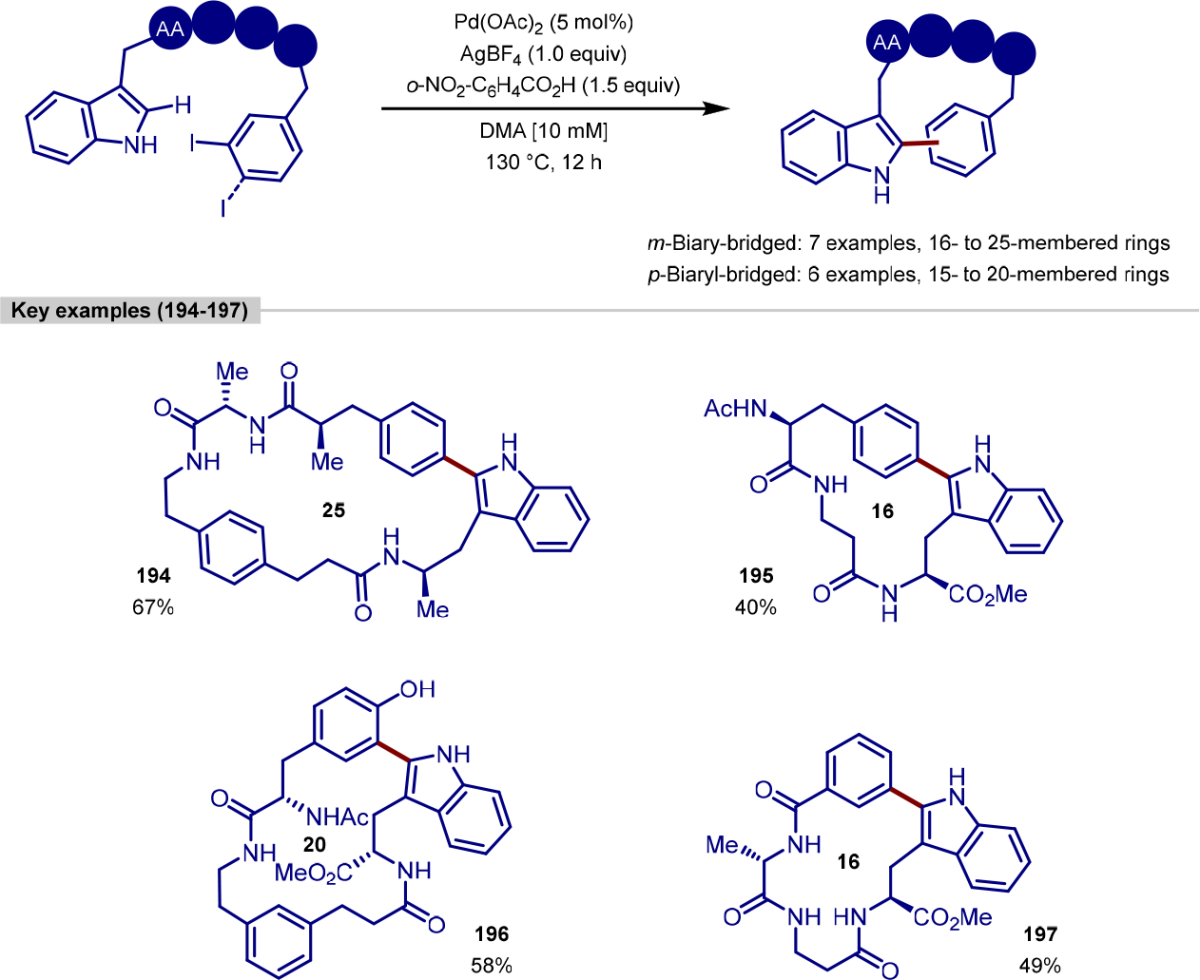
Peptide Macrocyclization by Palladium-Catalyzed C(sp^2^)–H Intramolecular Arylation

James also used a variation on the conditions by Larrosa on the C-2 arylation of indoles to realize peptide stapling between a *C-*terminus tryptophan and a *para*- or *meta*-iodo-phenylalanine residue located at the *N*-terminus (Scheme 40).^191^ Compared to the original conditions from Larrosa, elevated temperatures and greater dilutions (10 mM) were required for the intramolecular arylation, with the latter likely necessary to avoid cyclodimerization. Either *para*- or *meta*-biaryl-bridged macrocyclic peptides could be synthesized, with macrocyclic ring sizes up to 25-membered or 20-membered, respectively. Despite a broad range of ring sizes being demonstrated, the scope featured no examples of amino acid residues with unprotected polar side chains. Further to this, the *C*- and *N*-termini were protected as a methyl ester and acetamide, respectively.

## 3.0 C(sp^3^)–H Bond Alkylation

**Scheme 41:**
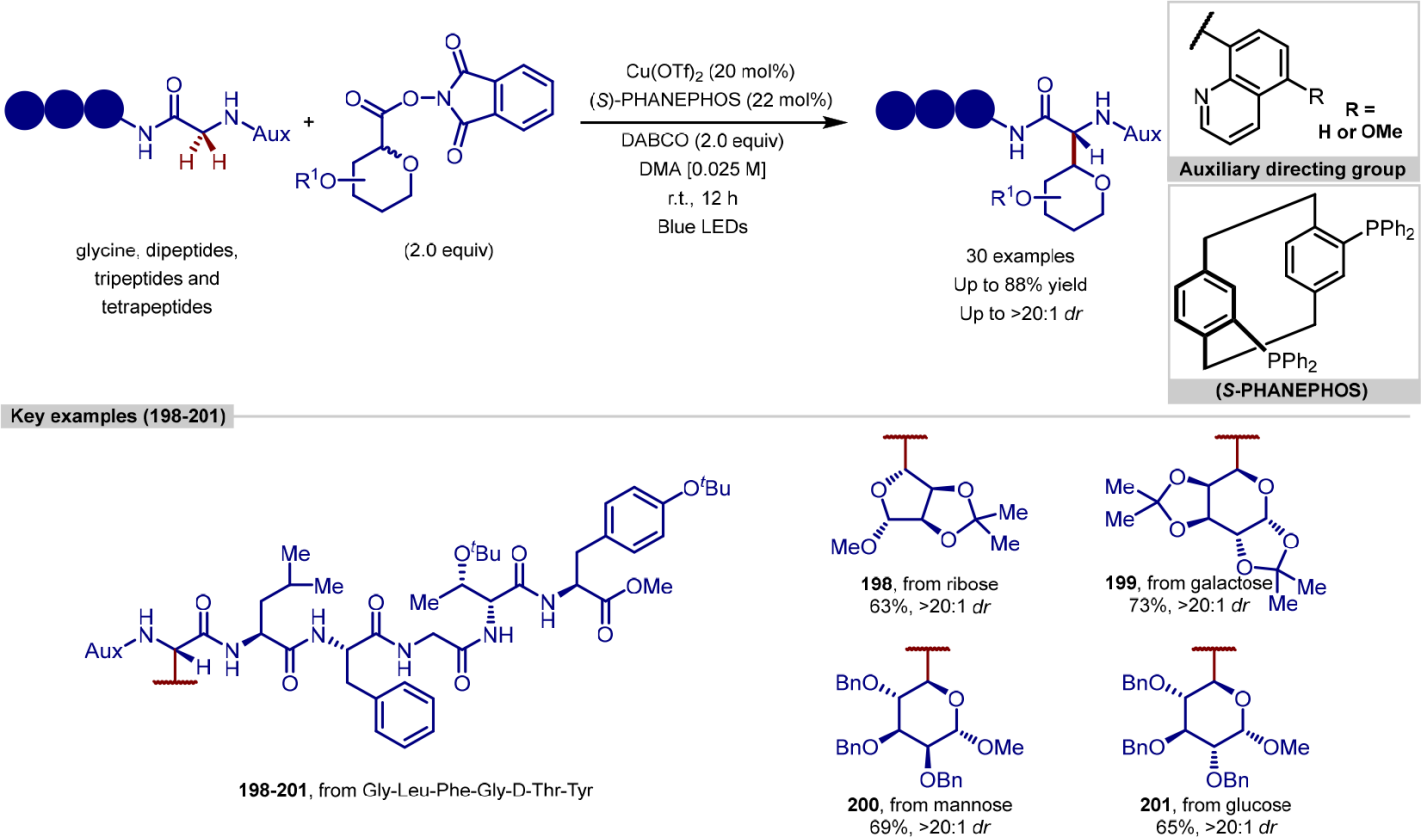
Copper-Catalyzed C(sp^3^)–H Glycosylation of Quinolinyl-8-glycinate Derivatives

In 2022, Xu reported a method for the C(sp^3^)–H glycosylation of quinolinyl-8-glycinate derivatives using visible light promotion and copper catalysis (Scheme 41).^192^ Previous methods for the *C*-glycosylation of peptides were restricted to those with tryptophan residues, or prefunctionalized peptides. While a palladium-catalyzed example was known, this proceeded under harsh reaction conditions and was not compatible with more complex peptides and oligosaccharide substrates.

Glycosyl NHP-esters derived from various monosaccharides including ribose, mannose, xylose, galactose, glucose, and fructose all participated in the reaction well, producing products in high yields and >20:1 d.r. in all examples. Tolerance of molecular complexity was demonstrated by the inclusion of di-, tri-, and even penta- and hexapeptides, without loss of regio- or stereoselectivity.

In 2007, in an effort to direct C–H functionalization with groups that can more easily be functionalized post-transformation, Yu reported the first palladium-catalyzed procedure for the coupling of both *ortho*-C–H bonds in benzoic acids and β-C–H bonds in aliphatic carboxylic acids with organoboron reagents.^193^ Unfortunately, this procedure necessitated the use of either aryl boronic esters or methyl boronic acids, and other C(sp^3^) boronic acids were not tolerated. This procedure also required the use of a stoichiometric silver oxidant, thus limiting sustainability of the process.

**Scheme 42:**
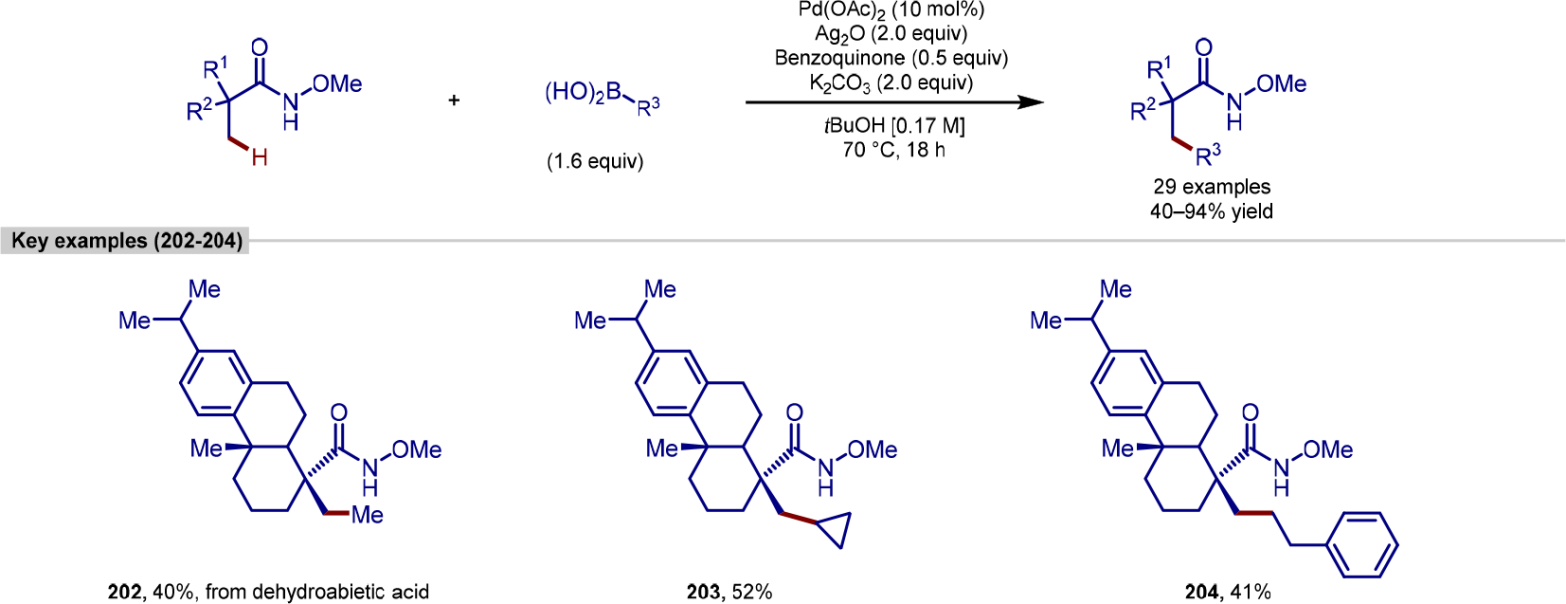
Methyl Hydroxamic Acids as Directing Groups for β-C(sp^3^)–H Alkylation

In further work in 2008, Yu reported the use of methyl hydroxamic acids as substrates to overcome the limitations associated with their previous procedure (Scheme 42).^194^ It was thought that the inability to use phenyl boronic acids, or other alkyl boronic esters, was due to undesired homocoupling or β-hydride elimination from the alkyl fragments of the C(sp^3^) boronic acids. Consequently, it was believed that derivatization of carboxylic acids into the structurally analogous and stronger binding *O*-methyl hydroxamic acids would prevent these unwanted processes from occurring.

Under the new conditions, a range of boronic acids were shown to function as coupling partners. Substituted and unsubstituted aryl boronic acids led to β-arylated products, with substitution on the aryl ring giving generally lower yields. Alkyl boronic acids were also suitable coupling partners, requiring the use of 2,2,5,5-tetramethyltetrahydrofuran as a solvent, which was proposed to act as a sterically bulky ligand that prevents homocoupling and β-hydride elimination. It was also shown that Ag(I) salts could be replaced by using air as an oxidant.

In addition to the application of this methodology to simple substrates, the utility of this reaction was demonstrated through the derivatization of dehydroabietic acid, a natural product identified as an efficient BK channel opener. Converting the carboxylic acid functional group to the *O*-methyl hydroxamic acid allowed for the diversification of an otherwise difficult-to-functionalize complex molecule. Through this method, the installation of methyl, cyclopropyl, and propylphenyl groups to the methyl were performed, generating three unique dehydroabietic acid derivatives.

**Scheme 43:**
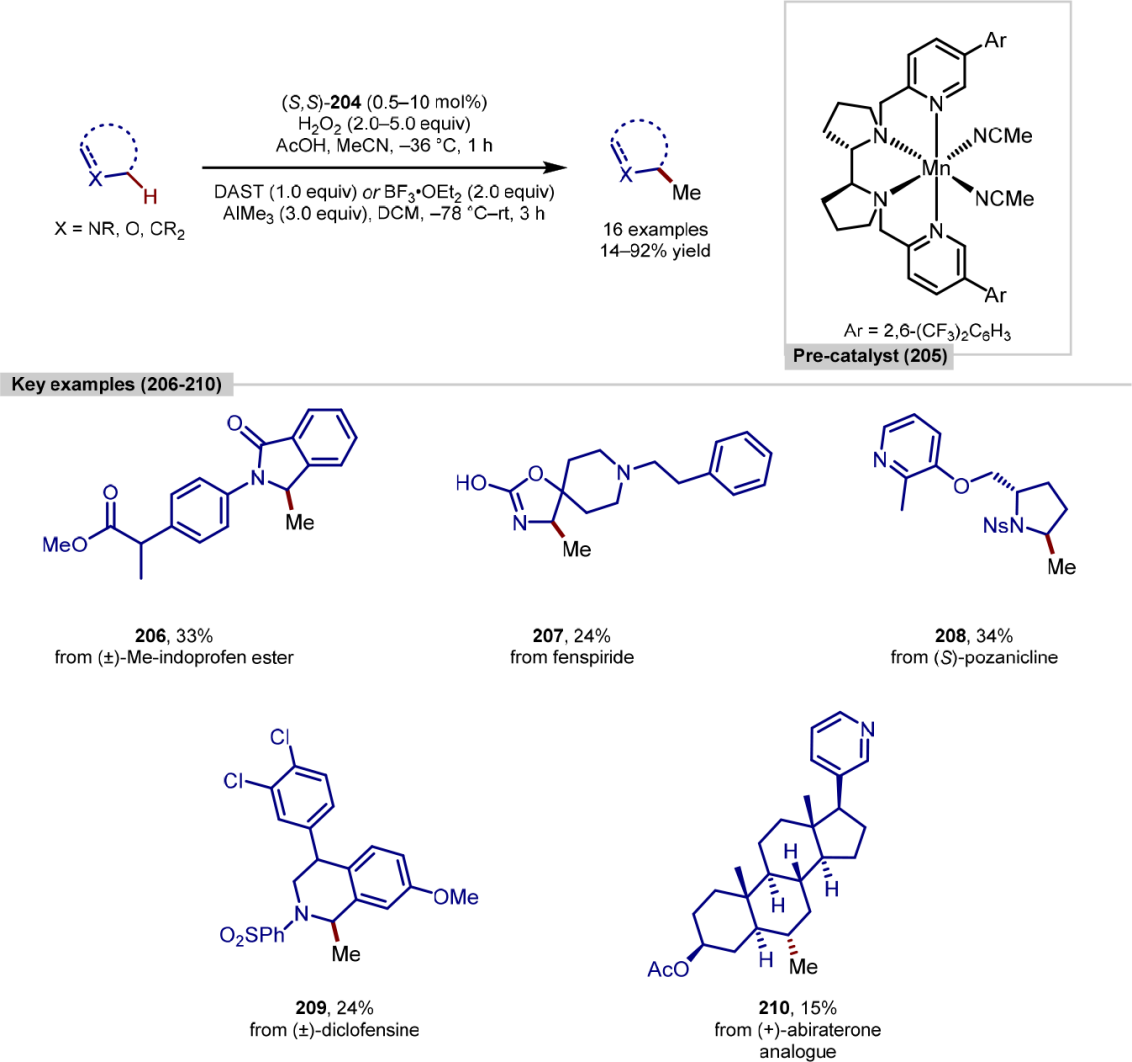
White’s Oxidative C(sp^3^)–H Methylation of Pharmaceuticals Procedure Using a Manganese Catalyst

In 2020, White presented the first generally applicable late-stage methylation (Scheme 43).^195^ This methodology used a of manganese catalyst (*S*,*S*)**-205** to enable the site-selective oxidative C–H hydroxylation of electron-rich and electron-neutral heterocycles with catalyst loadings as low as 0.5 mol %. A fluorine source or Lewis acid was then used to form a reactive iminium/oxonium intermediate with subsequent addition of the commercially available AlMe_3_ methylating reagent affording the desired products. The methodology was applied to 16 different medicinally relevant cores with 14–92% overall yields of products including **206**–**210** and was successfully performed on a gram-scale with no loss of efficacy. Separation of starting materials and products was difficult for direct methylation procedures due to the structural similarity of the materials. However, using this procedure this challenge could be overcome by separating the hydroxylated intermediate prior to methylation allowing pure products to be easily obtained. Substrates bearing enantioenriched stereocenters were also well tolerated under the reaction conditions with simple examples showing 100% enantiospecificity. While most examples result in activation of heterocyclic C–H bonds α-to nitrogen, higher loadings of the manganese catalyst were also shown to hydroxylate methylene C–H bonds, albeit in lower yields. This was demonstrated using an abiraterone analogue giving the product **210** in 15% yield and documents the first remote C–H methylation of an unactivated C(sp^3^)–H bond.

## 4.0 C(sp^3^)–H Bond Arylation

**Scheme 44:**
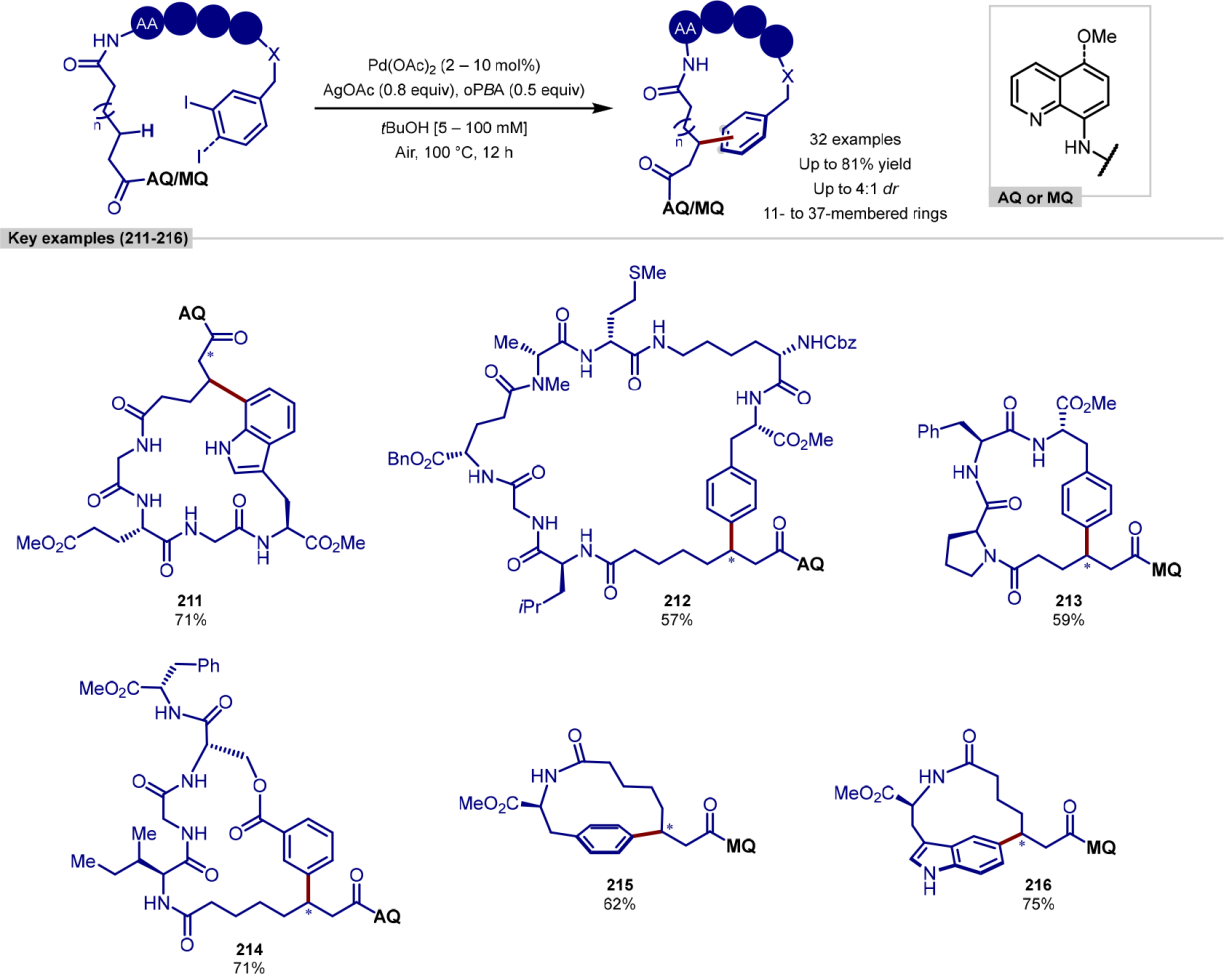
Intramolecular β-C(sp^3^)–H Arylation, Directed by an 8-Aminoquinoline (AQ) or 5-Methoxy-8-aminoqunioline (MQ) Auxiliary, Facilitates the Macrocyclization of Linear Peptides with (Hetero)aryl Iodide Amino Acid Derivatives at the *C*-Terminus

Macrocyclic peptides are an important and growing class of therapeutics used to treat a variety of different diseases and disorders.^196^ These scaffolds possess a large surface area and many functional groups akin to those found on protein surfaces, making them effective for targeting protein–protein interactions that are typically challenging druggable targets.^197^ This, in addition to the cyclic nature conferring physicochemical advantages over their linear congeners, makes efficient macrocyclization methodologies highly valuable. Chen have reported a palladium-catalyzed intramolecular β-C(sp^3^)–H arylation protocol that can provide entry to cyclophane-braced macrocyclic peptide scaffolds. Site-selectivity for C–H macrocyclization was achieved using the bidentate 8-aminoquinoline (AQ) or 5-methoxy-8-aminoquinoline (MQ) directing groups (Scheme 44).^198^ Cyclophane-type linkers have been found in cyclic peptide natural products, such as Vancomycin A and Celogentin C, with constrained conformations generated as a result of the transannular strain (in 1,4-systems) and rigidity of the aromatic plane. This transformation was made possible from previous palladium-catalyzed C–H functionalization methodologies and total syntheses reported by these authors.^199^ Within this work, aliphatic dicarboxylic acids of various chain lengths were appended at the *N*-terminus of peptides, with the other carboxylic acid site used to install the AQ or MQ directing groups, necessary to guide C–H arylation onto this aliphatic chain. High dilutions, typically 5 to 25 mM, were required to avoid dimerization and to encourage intramolecular reactivity (less than 5% dimerization reported for all examples), although concentrations as high as 100 mM were exemplified. The substrate scope demonstrated tolerance toward a range of ring sizes, with 11- to 37-membered macrocycles reported. The highly strained nature of smaller macrocycles was illustrated with an X-ray crystal structure for **215** which showed the phenyl ring to be bent out of the plane by 6.5°. Attempts to synthesize **215** under macrolactamization conditions (HATU/DIPEA) yielded less than 5% of the macrocyclic product. Different aromatic motifs could be employed for the cyclophane linkage, including 1,3- and 1,4-disubstituted (**214** and **215**) phenyl rings and indoles (**211** and **216**), enabled by a *C*-terminus tryptophan bearing an iodide at C-5. While a stereogenic center is formed in the C(sp^3^)–H arylation event, a maximum diastereoselectivity of 4:1 was achieved. Only removal of the MQ directing group was achieved using cerium ammonium sulfate to furnish, the corresponding free amide of **214** in 71% yield from the **214**-MQ analogue. Macrocyclic peptides synthesized using this protocol were screened against various cancer cell lines, assessing proliferation inhibition for macrocyclic peptides against their linear congeners. Peptide **214** showed the highest level of inhibition, reaching almost 1 μM potency against P4926 Tet off, a Myc-dependent cell line. A 20-fold difference in potency was reported versus the linear analogue of **214** and interestingly the analogue of **214** in which the AQ directing group was removed. This thereby demonstrated the potential for this methodology to provide efficient access to strained macrocyclic peptides that possess biological activity against a given Myc-dependent and independent cell lines.

**Scheme 45:**
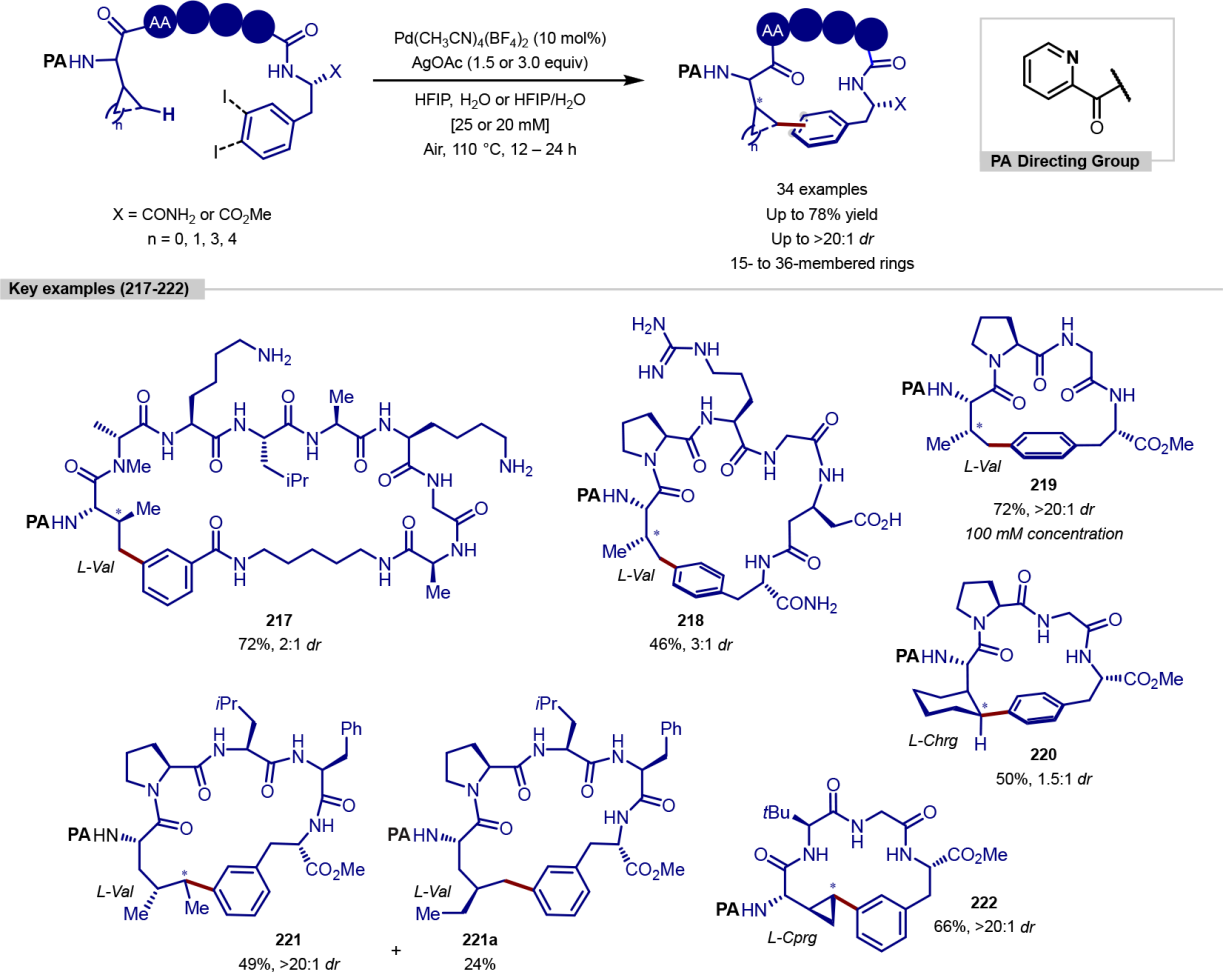
Intramolecular γ-C(sp^3^)–H Arylation, Directed by a Picolinamide (PA) Auxiliary Located at the *N*-Terminus, Facilitates the Macrocyclization of Linear Peptides with Aryl Iodide Amino Acid Derivatives at the *C*-Terminus

Chen has also employed an *N*-terminal picolinamide (PA) auxiliary to synthesize macrocyclic peptides through directed γ-C(sp^3^)–H arylation (Scheme 45).^200^ Using this auxiliary at the *N*-terminus permitted functionalization of amino acid residues directly, since the macrocyclization occurs onto the alkyl chain of the *N*-terminus residue. Furthermore, employing the PA auxiliary allowed for easier deprotection using Zn and aqueous HCl at room temperature. The γ-C(sp^3^)–H arylation transformation was optimized for valine, in which a primary C–H bond located on one of the geminal methyl groups was the site of C–H activation and subsequently macrocyclization. Compared to the previously described methodology, a cationic palladium(II) catalyst, Pd(CH_3_CN)_4_(BF_4_)_2_, was used and HFIP, H_2_O, or H_2_O:HFIP (9:1) was employed as the solvents of choice. Since this protocol allowed intramolecular arylation of terminal C–H bonds, the substrate scope was not restricted to cyclization onto aliphatic dicarboxylic acid fragments. Instead, a range of *N*-terminus amino acids possessing γ-C–H bonds were exemplified. These included l-valine (primary C–H bond, **217**, **218**, and **219**), l-isoleucine (competition between the secondary and primary C–H bonds, **221** and **221a**, respectively), L-*tert*-leucine, and d-allo-isoleucine. Unnatural amino acids containing constrained secondary C–H bonds could undergo macrocyclization, evidenced by **220** and **222**, with the substrates featuring a terminal cyclohexylglycine and cyclopropylglycine respectively. Introduction of both cyclophane-type linkers and saturated carbocycles into cyclic peptides could yield control over molecular conformation, generating rigid scaffolds that may confer advantages against a biological target. Unlike in the β-C(sp^3^)–H macrocyclization strategy, good chemoselectivity was achieved: polar functionalities such as amides, amines (Lys), guanidine, carboxylic acids, and alcohols (Ser) did not require protection. However, it was noted that thiol (Cys), imidazole (His), and indole (Trp) functionalities were incompatible. Future opportunities for this late-stage functionalization macrocyclization strategy may lie in the augmentation of automated SPPS with high-throughput experimentation to generate libraries of cyclic peptides containing a cyclophane-type linker and testing the biological activity against a given target.

**Scheme 46:**
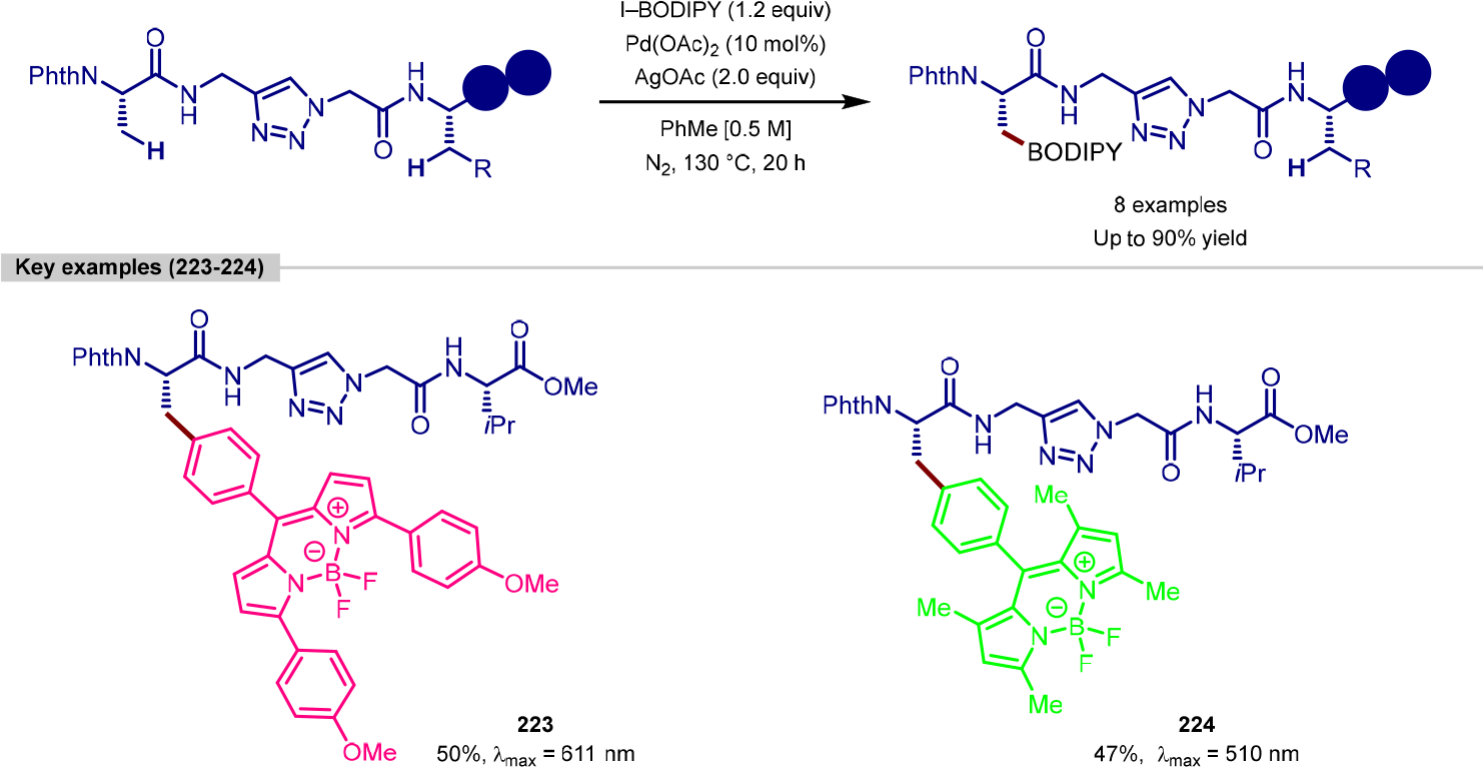
Palladium-Catalyzed β-C(sp^3^)–H Arylation of *N*-Terminus Ala Using 1,2,3-Triazoles, a Peptidomimetic, as a Directing Group with BODIPY Aryl Iodide Coupling Partners

The directing group strategy for C–H activation and subsequent functionalization has been used extensively, since it can enable highly site-selective transformations. However, in some cases the functional group used to direct C–H activation is redundant after the C–H functionalization event and requires removal with subsequent steps, generating additional waste when accessing target molecules. 1,2,3-Triazoles are heterocyclic motifs that can be synthesized in a highly efficient manner using Click chemistry approaches and permit the conjugation of biologically relevant molecules to another or to a fluorescent label. Ackermann has reported the β-C(sp^3^)–H arylation of *N*-terminus alanine residues in peptides, in which triazole is used as a directing group to achieve site-selectivity (Scheme 46).^201^ Within the context of peptides, the triazole motif can act as a peptidomimetic.^202^ The substrate scope featured only dipeptide substrates, joined by the triazole, with no polar functionalities present. However, it was demonstrated that various BODIPY analogues, a fluorescent dye used in biological labeling studies, could be installed in moderate yield. The authors also measured the maximum emission wavelengths of the products–the colors corresponding to the fluorescence of the molecule have been highlighted on the BODIPY label. While the C(sp^3^)–H arylation of phenylalanine was demonstrated on the single amino acid, achieving high diastereoselectivity, >20:1, this residue was not exemplified for the dipeptide or extended peptide substrates. This advance highlights the possibility of using the triazole linker to create peptide–drug conjugates and demonstrates the aptitude of the reported protocol for creating BODIPY-labeled analogues of these medicinally relevant conjugates. The authors have also reported the use of this triazole-directed method for the arylation of tripeptides with amino acid-based aryl iodides, including examples of aryl iodide coupling partners derived from tyrosine.^203^

**Scheme 47:**
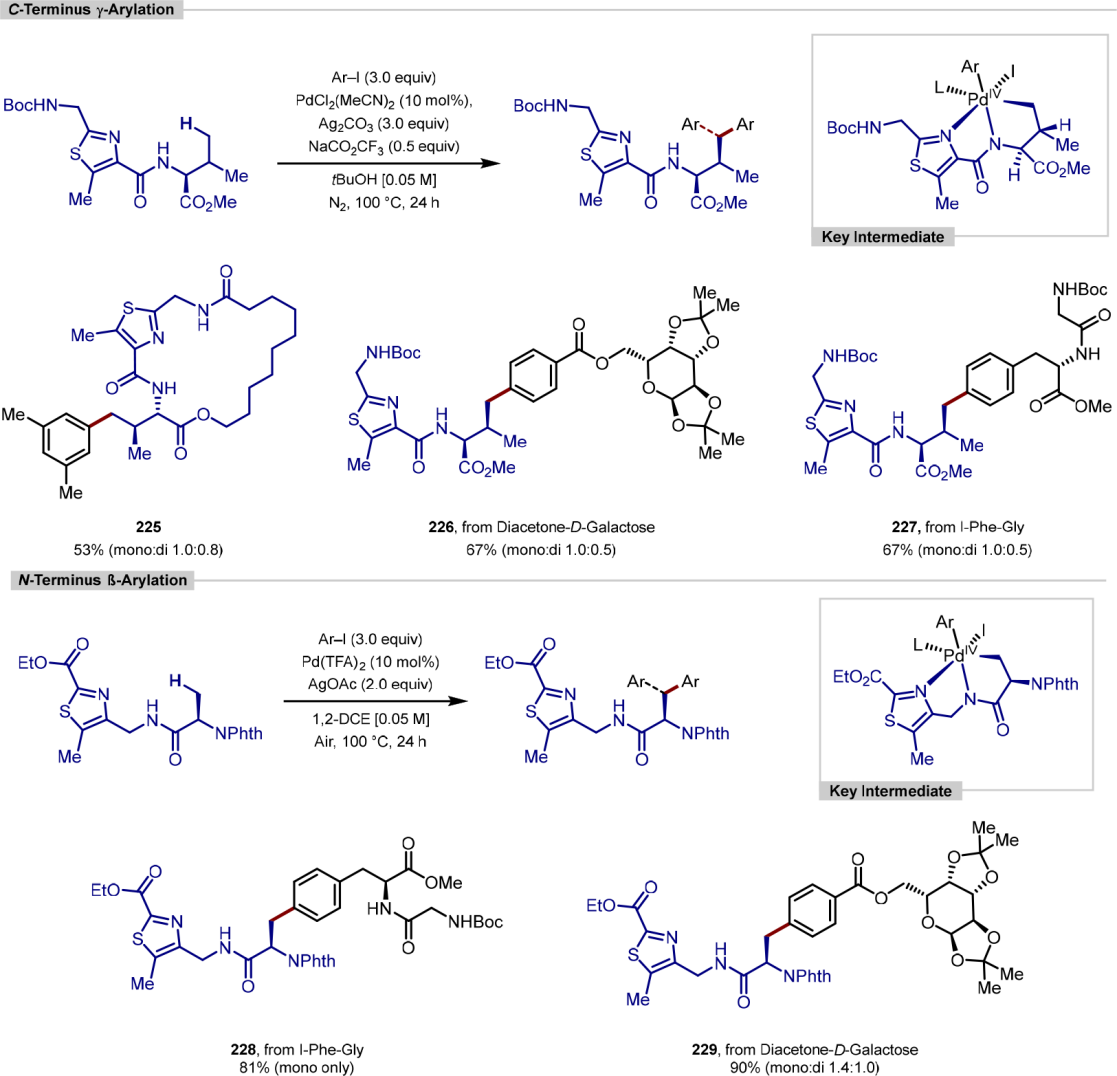
*C*-Terminus γ-C(sp^3^)–H Arylation and *N*-Terminus β-C(sp^3^)–H Arylation of Peptidic Substrates at Alanine Using Thiazole as a Directing Group

In a similar approach to *N*-terminal palladium-catalyzed C(sp^3^)–H arylation, Liu and Wang have utilized thiazole to serve as a directing group in peptidic substrates (Scheme 47).^204^ A range of different marketed peptidic drugs that display anticancer and antiviral properties contain thiazole units. In a similar manner to the triazole motif, thiazoles can act as amide bond surrogates, and owing to the conformationally constrained nature of the five-membered ring, can impart interesting topological effects in macrocyclic peptide systems.^205^ The authors demonstrated the ability of this directing group to activate a γ-C(sp^3^)–H bond in *C*-terminal valine residues (**225** to **227**) or a β-C(sp^3^)–H bond in *N*-terminal alanine residues (**228** to **229**) and enable the installation of various aryl groups. In many cases, a mixture of mono- and diarylation was observed. In both reaction mechanisms, the key intermediate was proposed to be a 5,5-fused bicyclic palladacycle, with a *N*,*N*-bidentate binding mode to the palladium center between the nitrogen atoms of the thiazole and adjoined amide, assisting direction toward the β- or γ-C(sp^3^)–H bond in a similar way to the 8-AQ system. Using either C(sp^3^)–H functionalization protocol, the requisite *C*- or *N*-terminal residue could be arylated with a protected derivative of d-galactose or dipeptide, with the latter coupling partner forming a noncanonical bond in a peptide system, not accessible through typical peptide synthesis approaches. The γ-C(sp^3^)–H arylation methodology was applied to a macrocyclic peptide substrate, affording **225** in moderate yield as a near 1:1 mixture of mono- and diarylation. This example demonstrated the opportunity to diversify biologically active macrocyclic peptides bearing the thiazole motif in a late-stage manner, accessing analogues that would require *de novo* synthesis of the macrocycle. A limitation of this late-stage functionalization methodology was that all polar (Lewis basic) functional groups required protection, thus application to a densely functionalized macrocyclic peptide may require reoptimization.

**Scheme 48:**
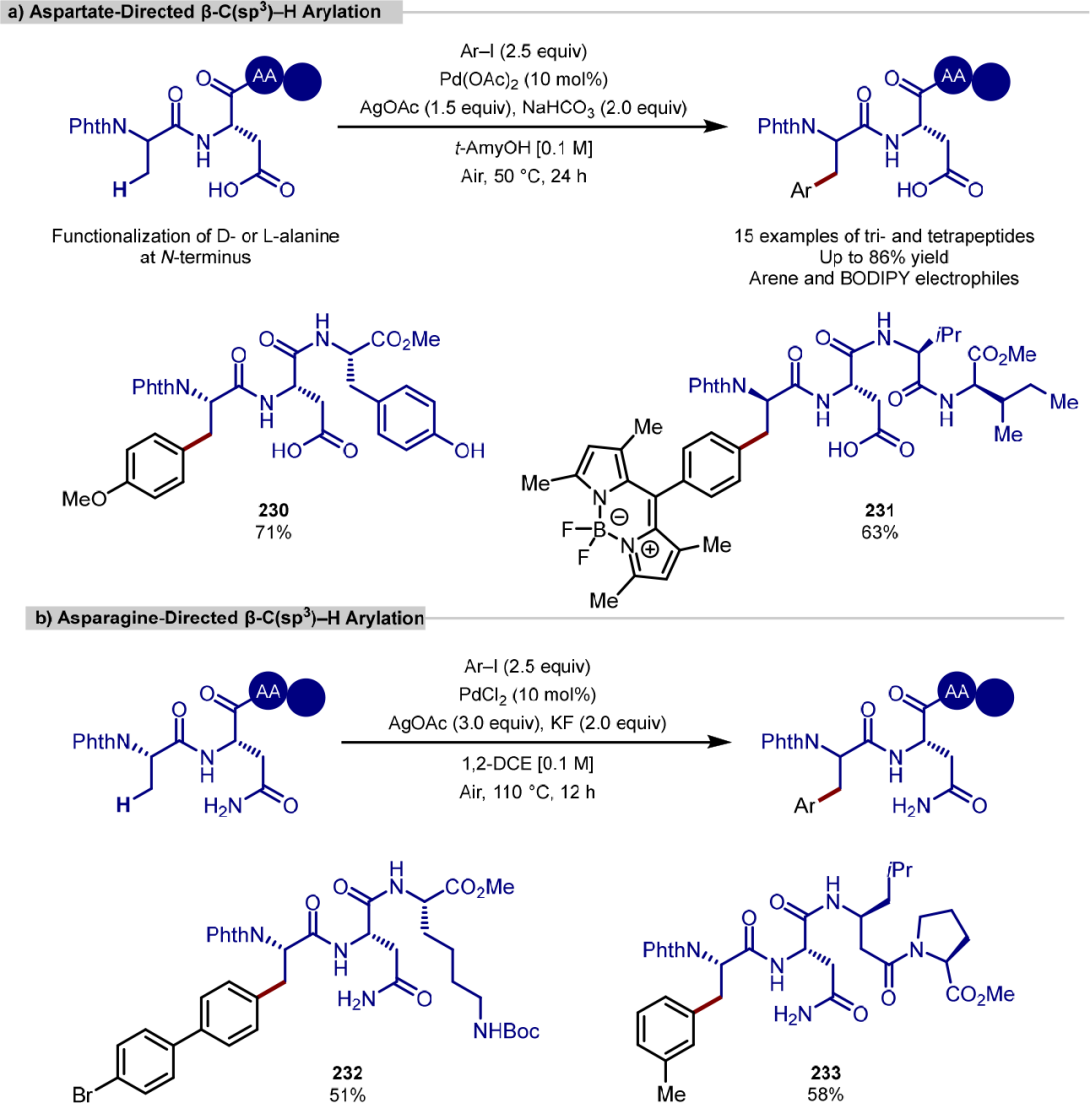
Carboxylate and Amide Side Chains of Aspartate and Asparagine Respectively Enable a Directed β-C(sp^3^)–H Arylation of Tri- and Tetrapeptides at Alanine with Aryl Iodides under Palladium Catalysis

In the examples of peptide C(sp^3^)–H arylation highlighted thus far, exogenous directing groups require installation to achieve the targeted site-selectivity. Considering the privileged nature of monoprotected amino acids as ligands in palladium-catalyzed C–H activation,^206^ using the endogenous functional groups of peptides to direct C–H activation has been challenging. A seminal report by Yu disclosed the C–H activation of peptides directed by native directing groups: either a *C*-terminus carboxylate in dipeptides or *C*-terminus amide in tri- and tetrapeptides.^207^ Building on this, Ackermann and Weng have shown a palladium-catalyzed β-C(sp^3^)–H arylation of *N*-terminal alanine residues in peptides that can be achieved by recruiting the side-chain carboxylate or amide of aspartate^208^ (Scheme 48a) or asparagine^209^ (Scheme 48b), respectively, as an endogenous directing group. In both instances, a 5,6-fused bicyclic palladacycle intermediate was proposed to form following the C–H activation step under a *N*,*N*- or *N*,*O*-bidentate binding mode with an internal amide and requisite peptide side-chain. Different palladium(II) precatalyst, additive and solvent were required for each directing group. While the reactions using asparagine operated under much harsher conditions compared to aspartate, racemization under either conditions was not reported. Various aryl iodide coupling partners showed success, generating unnatural α-amino acids at the *N*-terminus in good yields. BODIPY coupling partners could also be appended at the *N*-terminus, creating fluorescent labeled peptides. Further development of these methodologies would be investigating if longer chain or macrocyclic peptides were conducive to arylation and importantly the inclusion of amino acids with polar functional group-containing side-chains, e.g., Ser, Cys, and Lys.

**Scheme 49:**
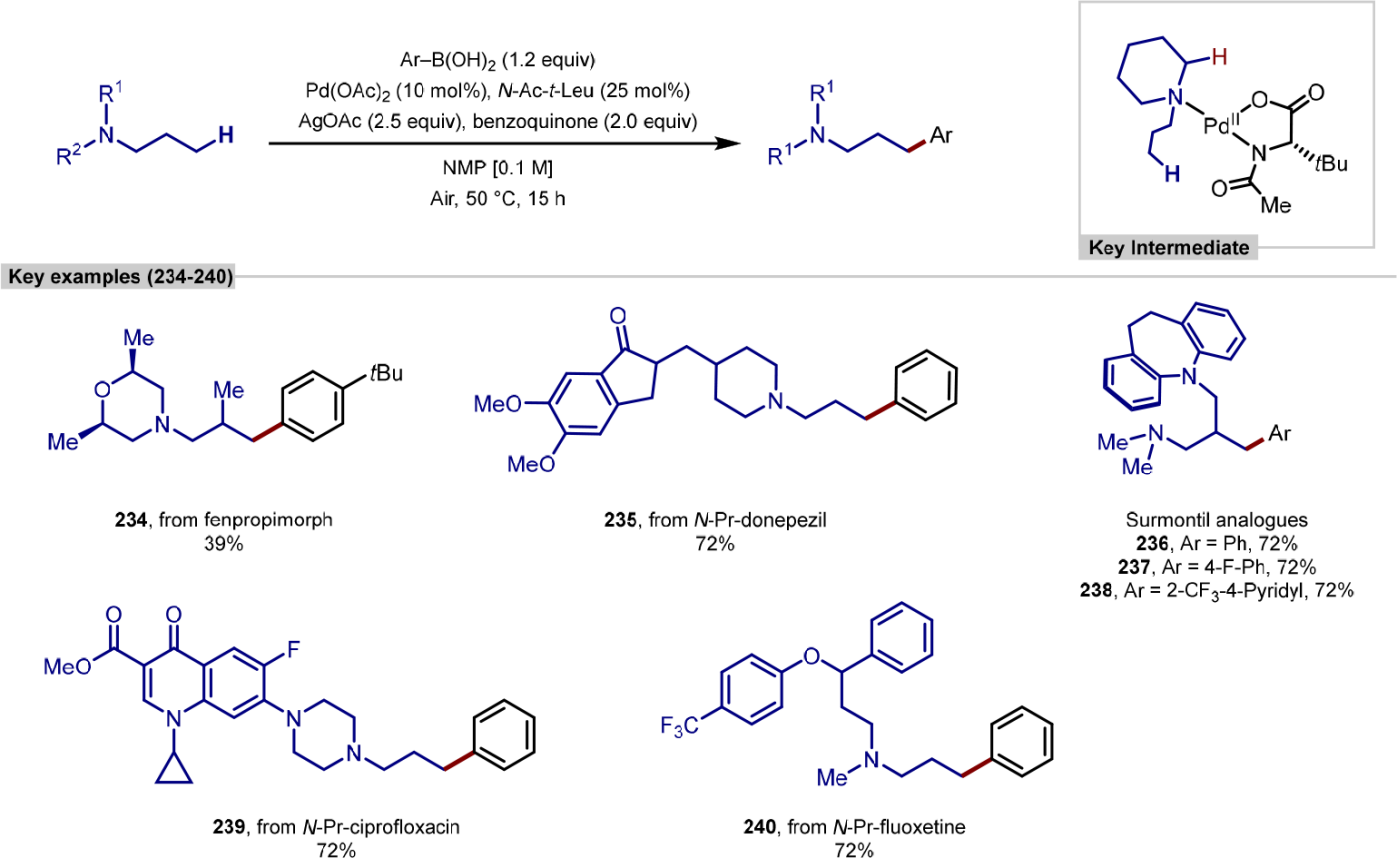
Palladium-Catalyzed γ-C(sp^3^)–H Arylation of *N*-Pr Derivatized Biologically Active Molecules with (Hetero)aryl Boronic Acids

Employing tertiary alkylamines as directing groups for C(sp^3^)–H functionalization is desirable, since this motif is recurrent in both pharmaceuticals and agrochemicals, thus expediting the generation of analogues of a lead compound through C–H functionalization approaches. The strongly directing nature, owing to a high Lewis basicity, of tertiary amines permits facile coordination to transition metal centers. However, once datively bound at the metal, tertiary alkylamines are susceptible to decomposition pathways such β-hydride elimination and amine oxidation under the reaction conditions typically employed in C–H activation protocols. Gaunt has shown that a monoprotected amino acid ligand can be used to overcome the innate and deleterious reactivity of tertiary alkylamines as directing groups and achieve γ-C(sp^3^)–H arylation.^210^ Here, the bisanionic, bidentate *N*-acetyl-*tert*-leucine ligand distorts the coplanar geometry required for β-hydride elimination (proton highlighted in red), thus energetically favoring a C–H activation event by a calculated 3.2 kcal mol^–1^, with the basic acetamide assisting C–H bond cleavage. Under optimized conditions, which were mild and tolerant of air, (hetero)aryl groups could be introduced at the γ-position in various drugs to which a propyl chain was appended to the nitrogen atom of secondary alkylamines (**235**, **239**, **240**, Scheme 49). Fenpropimorph and surmontil are an agrochemical and pharmaceutical respectively that contain a tertiary alkylamine with a γ-C(sp^3^)–H bond ready to undergo functionalization. Three analogues of the latter were generated in moderate to excellent yield (**236** to **238**) and a single analogue of the former synthesized in good yield (**234**).

**Scheme 50:**
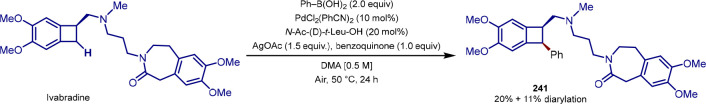
Palladium-Catalyzed γ-C(sp^3^)–H Arylation of Ivabradine with Phenylboronic Acid

Gaunt has extended the platform for tertiary alkylamine directed γ-C(sp^3^)–H arylation to functionalize aminomethylcyclopropane (AMCP) and aminomethylcyclobutane (AMCB) rings, prevalent motifs in pharmaceuticals, in a stereoselective manner under similar conditions to those previously reported.^211^ Introducing strained carbocycles such as these to drug candidates can lead to greater metabolic stability, owing to strong C–H bonds conferred by an enhanced π-character as a consequence of shorter C–C bonds.^212^ The reaction scope featured a broad range of tertiary alkylamine directing groups and aryl boronic acid coupling partners for both AMCP and AMCB systems. The enantioselective C(sp^3^)–H arylation protocol optimized for AMCB substrates was exemplified in the late-stage functionalization of ivabradine, with arylation proceeding in 20% yield (with 11% diarylated product) to generate the ivabradine analogue **241** as a single diastereomer (Scheme 50).

**Scheme 51:**
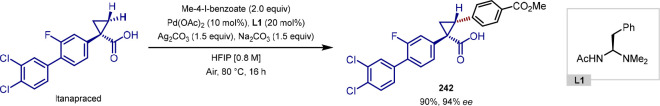
Palladium-Catalyzed β-C(sp^3^)–H Arylation of Itanapraced, Directed by a Native Carboxylic Acid

Another example of palladium-catalyzed enantioselective C(sp^3^)–H functionalization of cyclopropane rings that uses native directing groups has been reported by Yu, in which weakly coordinating carboxylic acids direct β*-*C(sp^3^)–H activation and affect a stereoselective arylation.^213^ This was the first example in which carboxylic acids could be used as directing groups in this class of transformation without preinstallation of an exogenous functionality at the carboxylic acid site, which would necessitate removal following C–H functionalization. Key to achieving reactivity and enantioselectivity was a monoprotected aminoethyl amine chiral ligand (**L1**) which could be accessed in four simple steps. Itanapraced, a γ-secretase modulator that has potential for treatment of neurological disorders such as Alzheimer’s, was used as a substrate to demonstrate the applicability of the methodology to late-stage functionalization that proceeded with both excellent yield and enantioselectivity to give **242** (Scheme 51).

**Scheme 52:**
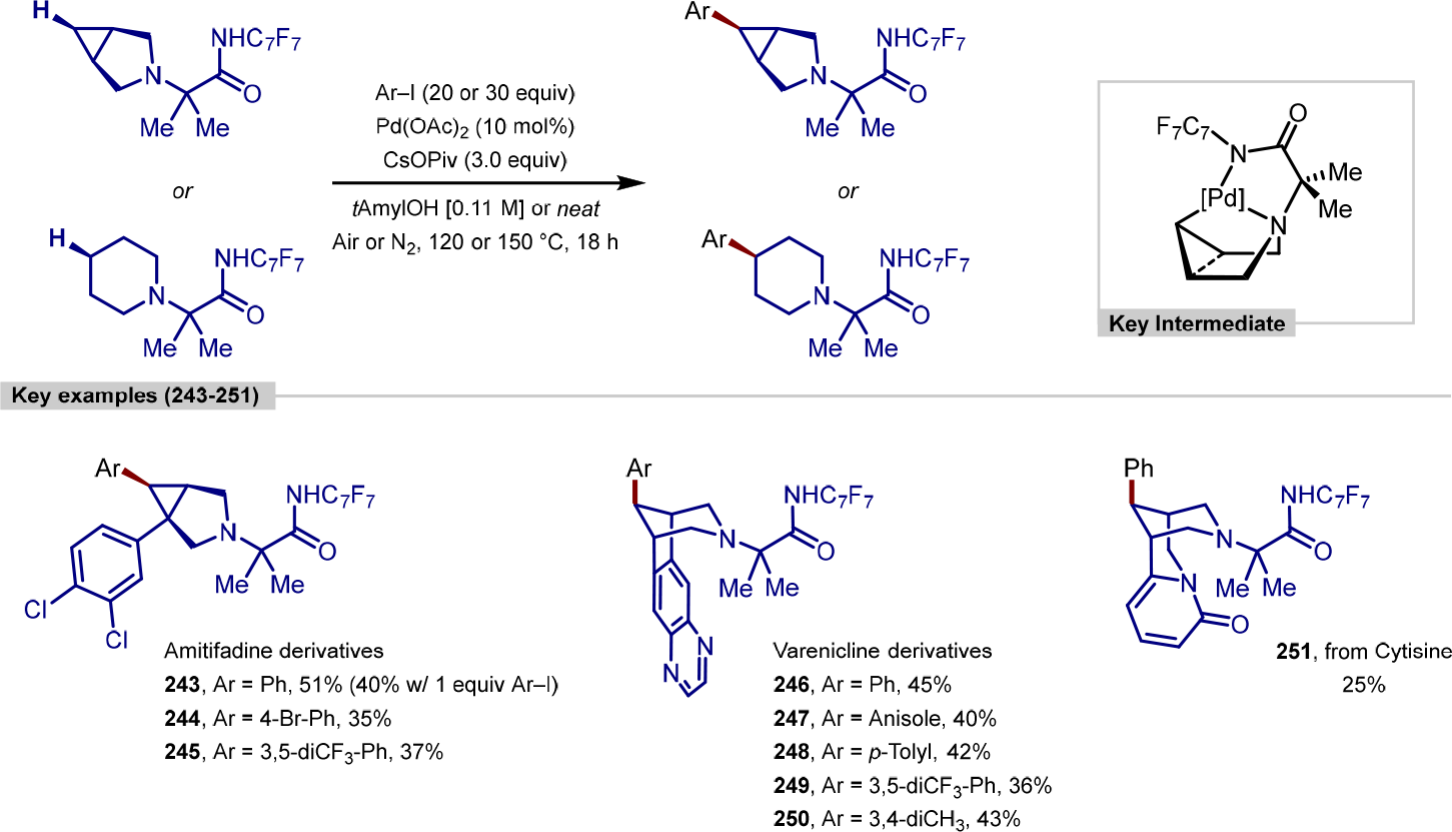
Palladium-Catalyzed Remote, Transannular C(sp^3^)–H Arylation of Pharmaceuticals and Biologically Active Molecules Containing 3-Azabicyclo[3.1.0]hexane or Piperidine Motifs

Alicyclic amines are prevalent motifs in pharmaceutical agents and agrochemicals. As of 2014, such ring systems comprised three of the top ten most common rings found in drugs that included aromatic systems.^176^ Recent drug discovery approaches have sought to increase the fraction of sp^3^ carbon atoms within lead compounds,^214^ with high values of this molecular descriptor being linked to decreased attrition rates in clinical trials; the prominence of alicyclic amines is therefore likely to increase.^215^ Using transition-metal-catalyzed C–H functionalization to build libraries of ring-substituted analogues is therefore of great interest. Seeking to broaden the scope of alicyclic amine C–H functionalization beyond functionalization at the α-position or at C–H bonds *exo* to the alicyclic amine, Sanford has reported the palladium-catalyzed remote, transannular C(sp^3^)–H arylation of 3-azabicyclo[3.1.0]hexane and piperidines (Scheme 52).^216^ To access the lower equilibrium populated boat conformation of such systems with greater ease, 3-azabicyclo[3.1.0]hexane was chosen as the test substrate to achieve optimal conditions. Achieving reactivity in this substrate was easier since the requirement for boat conformation is fulfilled by the innate conformation of the bicyclic ring system. Key to reactivity was a fluorinated anilide directing group appended to the alicyclic nitrogen atom. This directing group which was proposed to assist C–H activation, affording a bicyclic palladacycle intermediate. This auxiliary could be removed following the arylation procedure using SmI_2_, with this being demonstrated on the test substrate, achieving 52% yield over three steps (DG installation, transannular C(sp^3^)–H arylation then DG removal). An interesting feature of the optimized reaction conditions was cesium pivalate replacing the stoichiometric silver salt used in similar processes to perform iodide abstraction. Amitifadine is a pharmaceutical, used in the treatment of depression, possesses the 3-azabicyclo[3.1.0]hexane ring system and so is a prime candidate for illustrating the applicability of the transannular arylation to a complex substrate following appendage of the anilide directing group. For this substrate, three different aryl iodide coupling partners were demonstrated in good yield.

The transannular arylation of the piperidine ring system is more difficult, owing to the lower equilibrium population of the boat conformation that is key to achieving the C–H activation at a remote C–H bond. The cyclopropyl C–H bond is also weakened relative to the C–H bonds in piperidine due to a more sp^2^-like character. These factors contributed to an estimated 6 kcal mol^–1^ increase in activation energy barrier for the piperidine substrate. The optimized reaction conditions reflected this, with the reactions being run neat and at a higher temperature. Complementing a broad substrate scope for this class of alicyclic amines, which included piperidines and various bicyclic amines, the authors applied the conditions to generate arylated analogues of Varenicline and cytisine, all in moderate yield. While a large excess of aryl iodide was required, this methodology permits access to arylated analogues of relevant drug molecules that would be time-consuming to synthesize *de novo*.

**Scheme 53:**
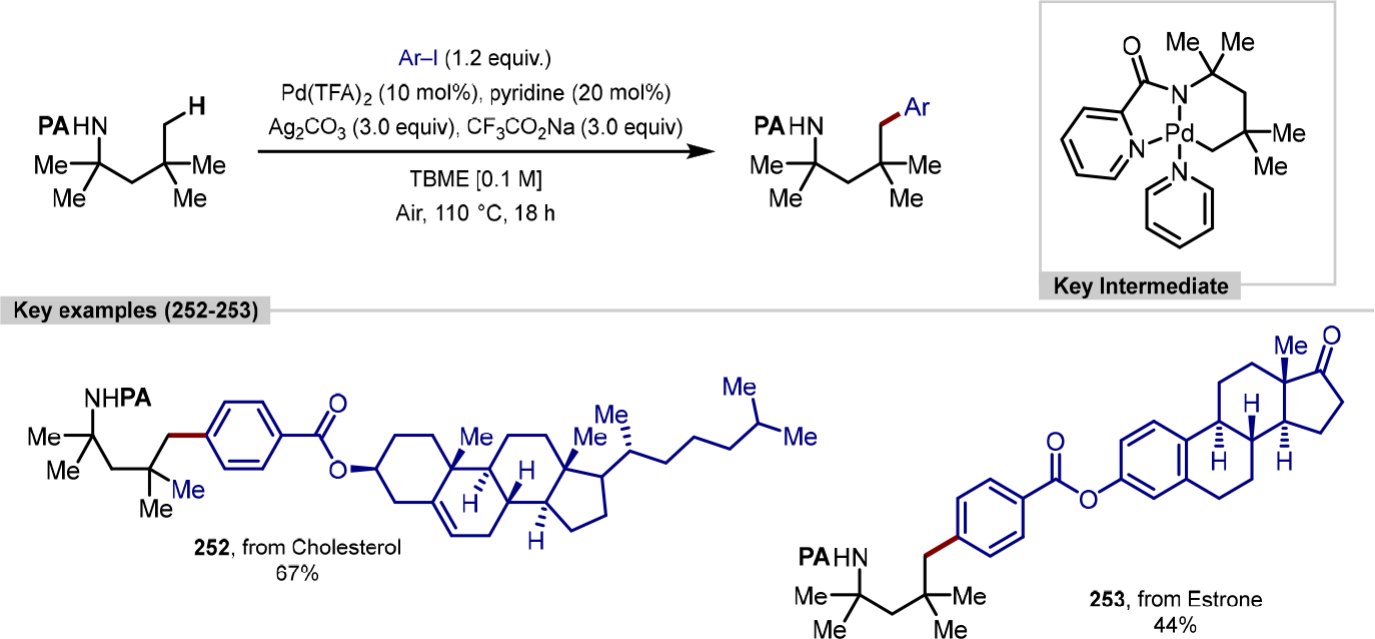
Picolinamide-Directed δ-C(sp^3^)–H Arylation of Amines with Aryl Iodide Derivatives of Complex Molecules

The arylation of remote δ-C(sp^3^)–H bonds in aliphatic and alicyclic amine systems catalyzed by palladium has been achieved by Maiti, through the use of a PA directing group (Scheme 53).^217^ Optimized conditions were applied to a range of different natural product derivatives, including cholesterol and estrone, with the aryl iodide component installed *via* esterification with 4-iodobenzoic acid. The role of the simple pyridine ligand was elucidated using both experimental and computational methods. Early in the catalytic cycle, the pyridine was proposed to dissociate a trinuclear palladium-paddlewheel complex formed by bridging acetate ligands, with the formation of mononuclear Pd(TFA)_2_(Py)_2_ both accessing the mononuclear pathway required for catalysis and being enthalpically favored. Thermodynamically, the pyridine conferred a 10.9 kcal mol^–1^ lower energy barrier, increasing the rate of reaction under the conditions. A 5,6-fused bicyclic palladacycle with a coordinated pyridine was synthesized, an X-ray crystal structure obtained and demonstrated to be competent as a well-defined catalyst for arylation of the PA-appended substrate. It was also proposed to prevent additional arylation events prior to protodemetalation by coordination being thermodynamically favorable over another oxidative addition of aryl iodide.

**Scheme 54:**
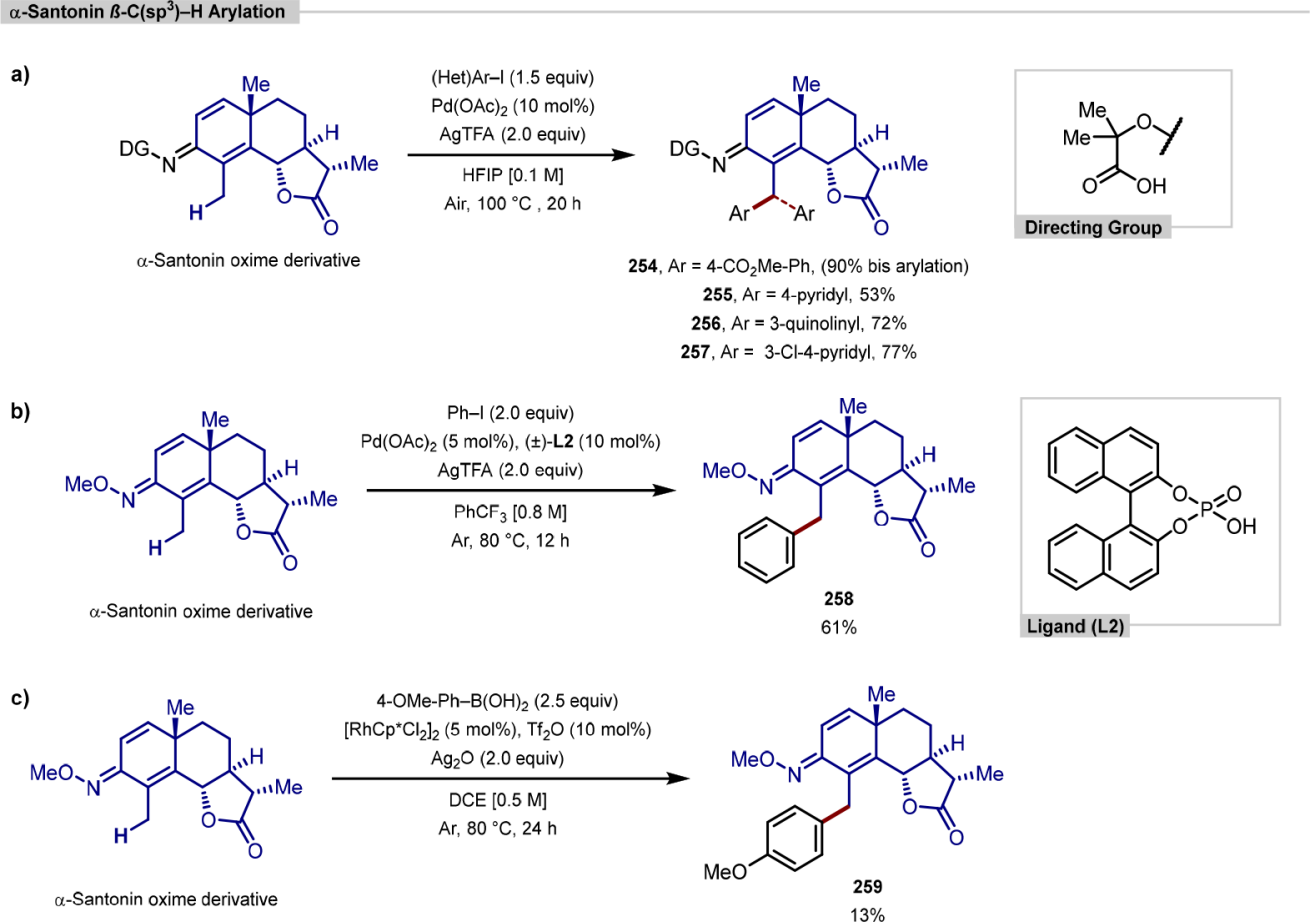
Approaches to the β-C(sp^3^)–H Arylation of α-Santonin Using Different Oxime Auxiliaries to Achieve Site-Selective C–H Activation

α-Santonin is a sesquiterpene lactone that has historical use as an anthelmintic; however, hepatic and renal toxicities and mental defects have seen other treatments replace it for this means. It has been shown to possess antioxidant, anti-inflammatory, and immunosuppressive properties.^218^ Therefore, accessing analogues of α-santonin to develop lead compounds for the aforementioned treatments is of interest. α-Santonin has been exemplified as a substrate in two palladium-catalyzed C(sp^3^)–H arylation procedures and an approach that used rhodium catalysis, with all targeting the same β-C(sp^3^)–H bond, directed by an oxime-based auxiliary. In the example from Yu,^219^ the dimethylaminooxyacetic acid auxiliary was designed to ensure facile C–H palladation by serving as an L,X-type bidentate ligand that generates a palladium(II) species with a coordinated acetate to enable the C–H activation reaction to occur (Scheme 54a).^220^ The weakly binding carboxylate on the directing group was proposed to assist in subsequent reactivity versus traditional L,L-type bidentate directing groups. The protocol was exemplified with aryl and three heteroaryl iodide coupling partners in good to excellent yield. The approach to the β-C(sp^3^)–H arylation of α-santonin reported by Shi operates under similar conditions but employs a BINOL-derived phosphoric acid, with the role of this additive in catalysis was not investigated (Scheme 54b).^221^ Only a single example using simple phenyl iodide was reported, with a moderate yield achieved. Sharma reported a rhodium-catalyzed reductive β-C(sp^3^)–H arylation approach to α-santonin derivatization (Scheme 54c).^222^ Only a single turnover was achieved in this transformation, with this inefficiency linked to the reaction conditions that had been optimized for the C(sp^3^)–H arylation of 8-methylquinoline substrates.

**Scheme 55:**
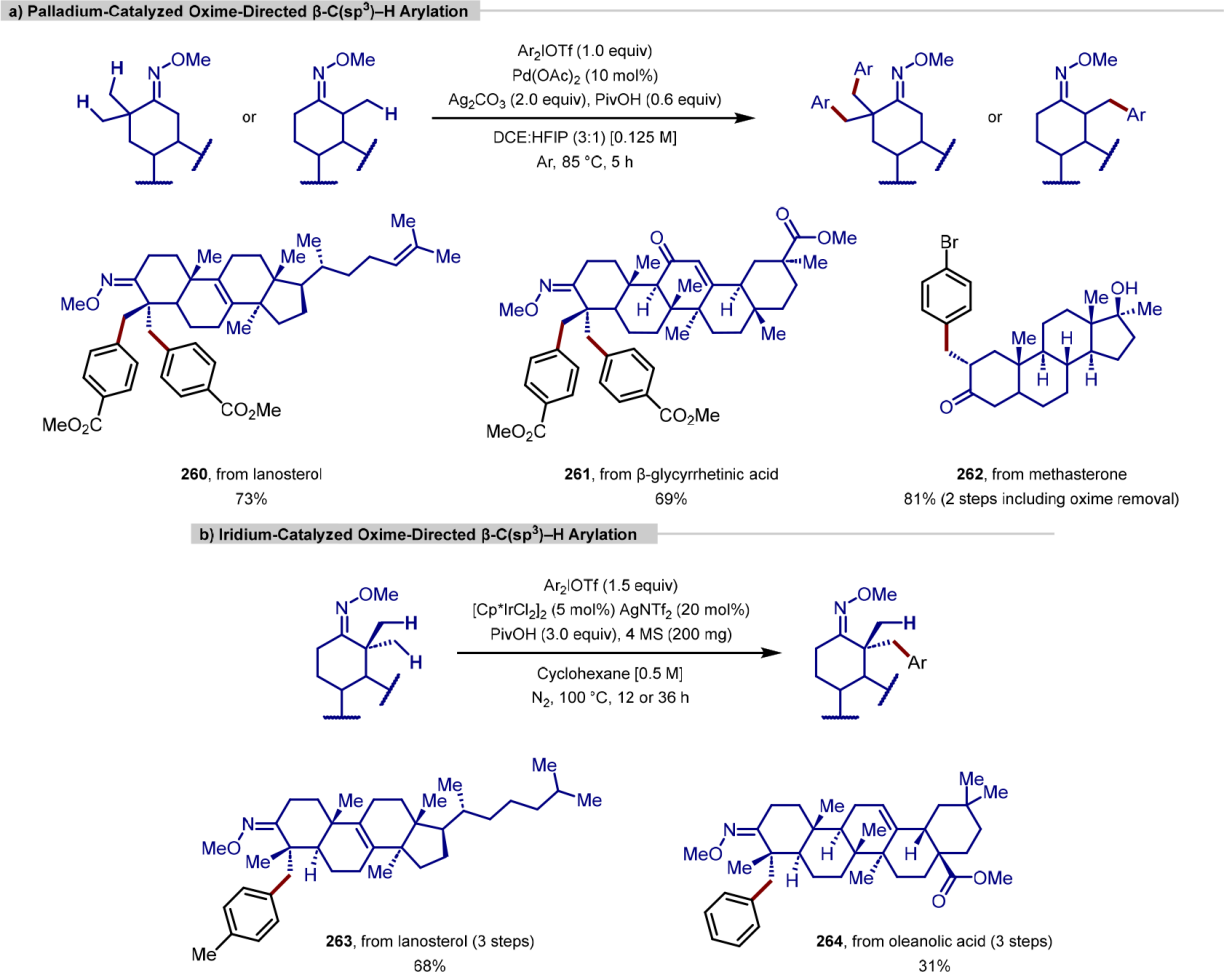
Oxime-Directed β-C(sp^3^)–H Arylation of Natural Product Derivatives

The β-C(sp^3^)–H arylation directed by oxime functionalities has also been applied to other steroidal natural products, using diaryliodonium salts as the aryl source under either iridium^223^ or palladium^224^ catalysis with moderate to excellent yields reported. In the palladium-catalyzed example by Chen (Scheme 55, **a**), an 81% isolated yield was attained across the C(sp^3^)–H arylation and oxime removal steps. Bis-arylation of lanosterol and β-glycyrrhetinic acid derivatives under palladium catalysis could be achieved using two equivalents of the diaryliodonium salt. Site-selectivity for the monoarylation in the iridium-catalyzed arylation reported by Xia and Shi was determined by either X-ray crystallography or NOESY NMR experiments (Scheme 55, **b**). All examples of functionalization across both reports required extensive substrate manipulation prior to subjection to the requisite directed C(sp^3^)–H arylation conditions. Manipulation included esterification of carboxylic acids, oxidation of secondary alcohols to generate a ketone for oxime installation and in the case of lanosterol, hydrogenation of the terminal tertiary alkene.

## 5.0 C–H Bond Alkenylation Strategies

Metal-catalyzed C–H alkenylation protocols have been extensively investigated over the last 20 years. Several transition metals along with the use of directing groups have been used to give important and useful results in terms of reactivity and selectivity. In addition, other methodologies have been described without the use of a DG and have obtained high levels of selectivity. These methods have used specific ligands that accelerate the C–H activation step and helps in the control of the selectivity or make use of the electronic nature of the arene that was targeted for functionalization. Several research groups have described different protocols for the alkenylation of simple molecules and once optimal reaction conditions were established, these methods were extended to the late-stage functionalization of molecules with higher structural complexity. In this section we describe the extension of these alkenylations to the functionalization of pharmaceuticals, biologically active molecules and their analogues. Alkenes are versatile building blocks, giving the chemist the opportunity to incorporate different functionalities at four different positions. The importance of the alkene moiety in pharmaceutical and bioactive compounds relies on the planarity and rigidity of the double bond, fixing the four functional groups attached to the olefin within a rigid conformation, allowing the synthesis of a large variety of derivatives to interact with different targets.^225,226^

### 5.1 *ortho*-Alkenylation

**Scheme 56:**
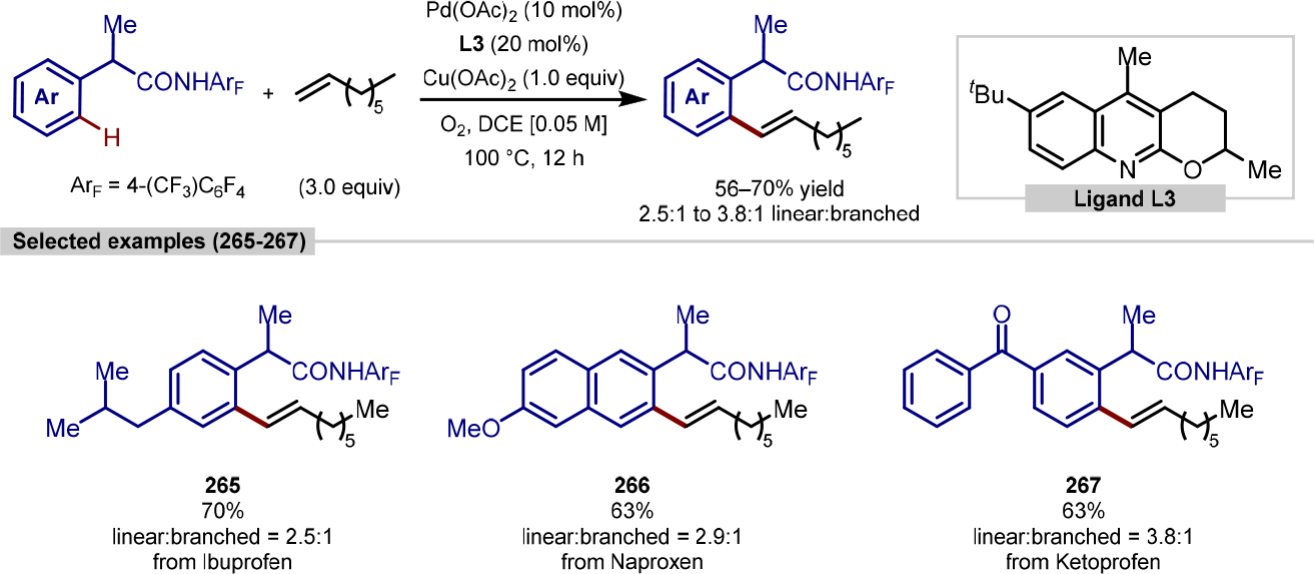
Palladium-Catalyzed *ortho*-Selective Alkenylation of Phenylacetic Amides with Unactivated Alkenes

Yu described the palladium-catalyzed C–H alkenylation of phenylacetic amides with challenging unactivated alkenes, thus constituting the activation of two C(sp^2^)–H bonds (Scheme 56).^227^ The use of substoichiometric quantities of pyridine and quinoline ligands was crucial to obtain good reactivity, and **L3** performed the best in terms of reactivity and selectivity with respect to the linear versus branched olefin products. The reaction proceeded at 100 °C, using oxygen as a terminal oxidant, with one equivalent of Cu(OAc)_2_ acting as co-oxidant in DCE as solvent. The methodology was applied to the olefination of ibuprofen, naproxen, and ketoprofen analogues were achieved using this methodology, with good yields and moderate linear-to-branched olefin selectivity. It is important to highlight that the C–H olefination reaction using unactivated alkenes are still a challenge in organic synthesis methodology.

**Scheme 57:**
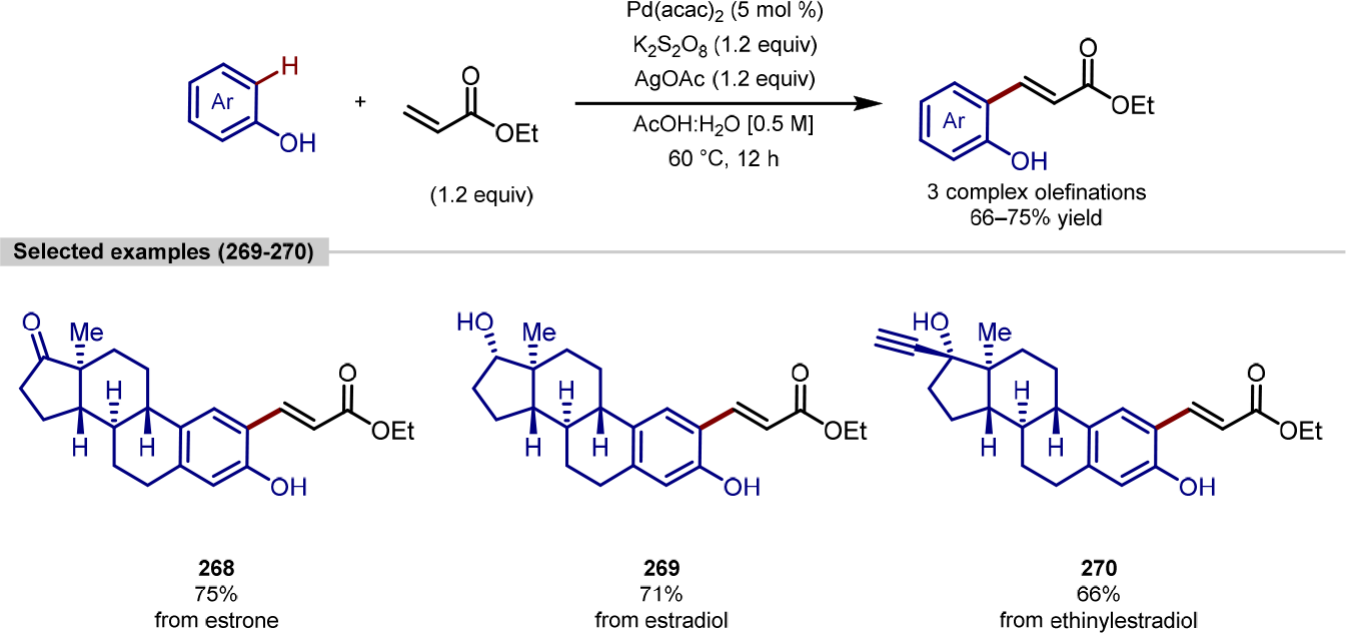
Palladium-Catalyzed Late-Stage *ortho*-Selective Alkenylation of Biological Active Phenols

Similarly, Zhu reported the palladium-catalyzed late-stage C–H *ortho*-selective alkenylation of phenols (Scheme 57).^228^ Exclusive levels of *ortho*-selectivity were achieved with the phenolic hydroxyl-group acting as a directing group, with this protocol being the first regioselective olefination of unprotected phenols. The use of potassium persulfate as an oxidant, along with silver acetate, and green solvents (water and acetic acid) afforded the *ortho*-alkenylated products using only mild temperature (60 °C). The late-stage alkenylation products of estrone **268**, estradiol **269**, and ethynylestradiol **270** were obtained with good yields (66–75%), and these derivatives showed enhanced inhibitory activities toward MCF-7 and PC-3 cancer cell lines, compared with their nonolefinated analogues.

**Scheme 58:**
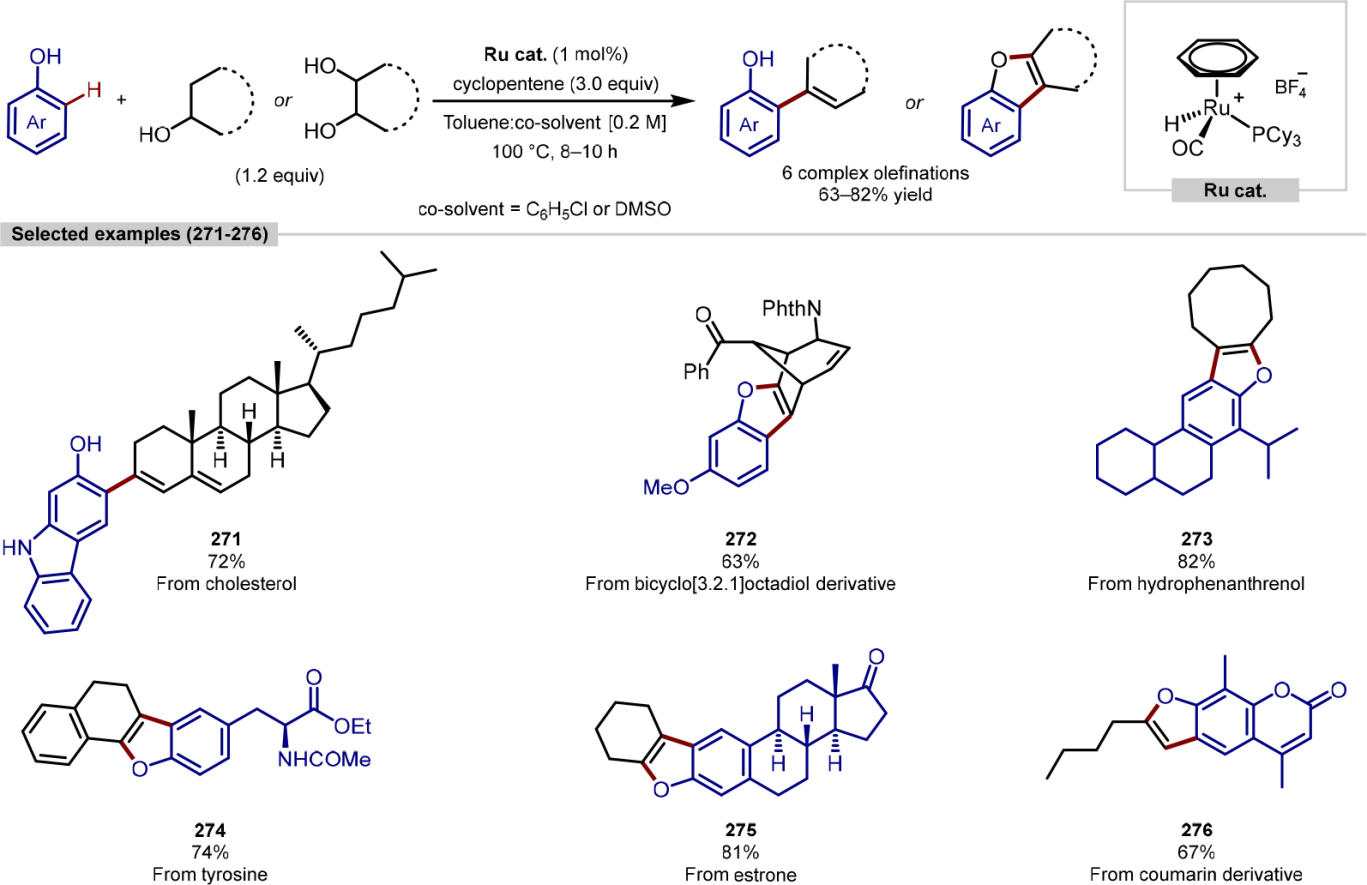
Ruthenium-Catalyzed *ortho*-Alkenylation of Phenols Using Alcohols and 1,2-Diols as the Source of Alkene Coupling Partner

An alternative strategy to obtain *ortho-*alkenylated phenols was described by Yi in 2012 (Scheme 58).^113^ In this example, using ruthenium catalysis, instead of directly using alkenes as reagents, alcohols and diols were used in a dehydrative mechanism. The authors developed first the alkylation of phenols with alcohols, and after the optimization of this reaction, they extended the method to obtain styrenes and benzofuran derivatives in a tandem alkylation/dehydrogenation reaction. A key feature for both reactions was the use of cyclopentene as a sacrificial hydrogen acceptor in the C–H activation step. The use of alcohols led to olefinated products, and the use of 1,2-diols led to benzofuran derivatives. The developed alkenylation reaction was successful for six different substrates using both approaches: the alkenylation of the drug-like molecule and using the drug molecule as the alkenylation partner.

**Scheme 59:**
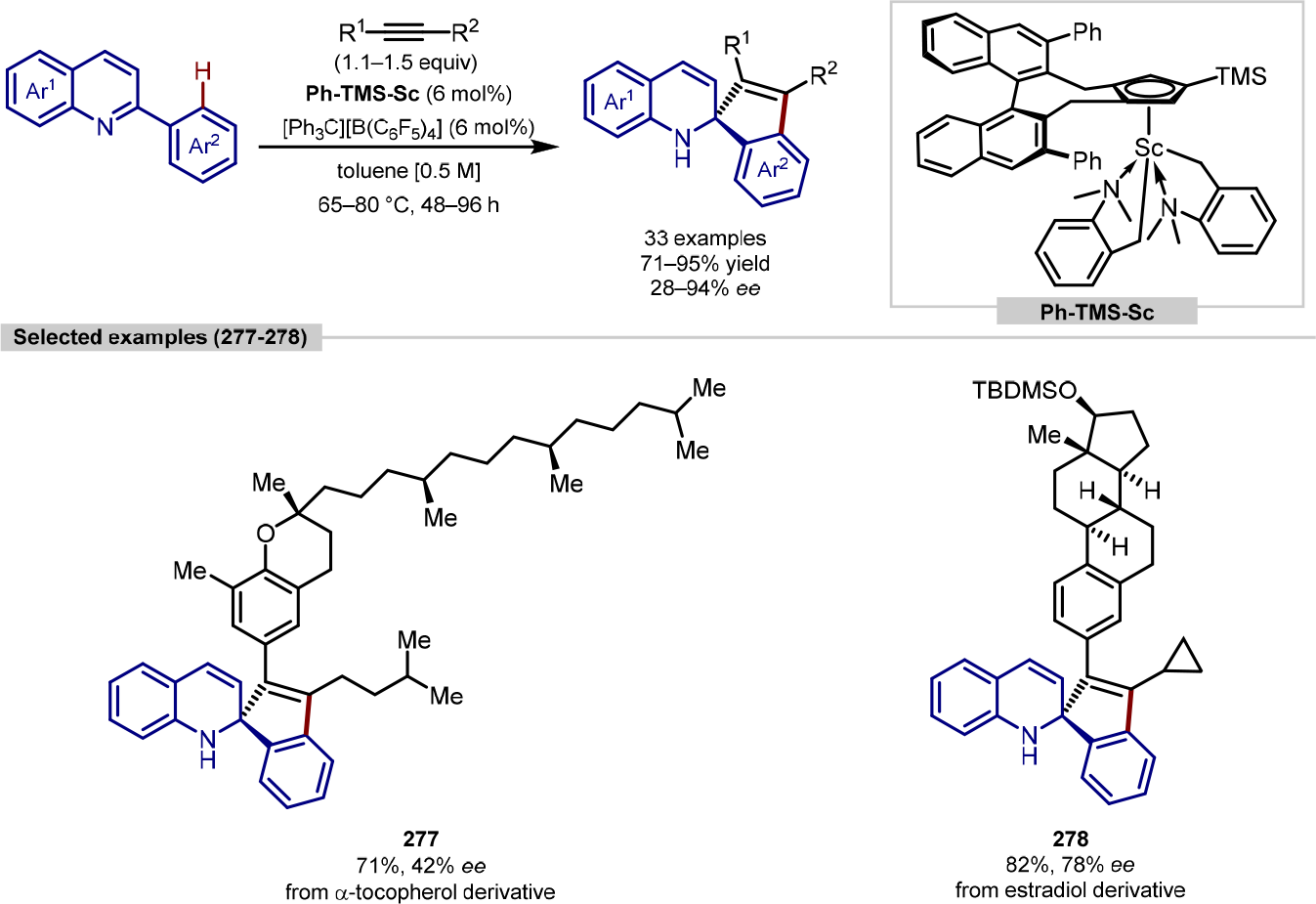
Scandium-Catalyzed *ortho*-Alkenylation and spiro-Annulation of 2-Arylquinolines Using Alkyne-Derived Bioactive Molecules

Recently, Hou developed the scandium-catalyzed dearomative spiro-annulation of quinolines with alkynes (Scheme 59).^229^ The quinoline group of the 2-arylquinolines starting materials acted as a directing group to perform the *ortho*-selective C–H activation with an enantiopure cyclopentadienyl scandium catalyst following by the alkenylation *via* alkyne insertion. The intermediate would emerge by intramolecular 1,2-addition of scandium-alkenyl bond to the C–N double bond of the quinoline ring, affording the dearomatized enantioenriched products **277** and **278**. Excellent yields and enantioselectivities were obtained using simple starting materials, and good yields and modest to good enantioselectivities were obtained for more complex molecules, with two examples of using alkyne-derived bioactive molecules.

**Scheme 60:**
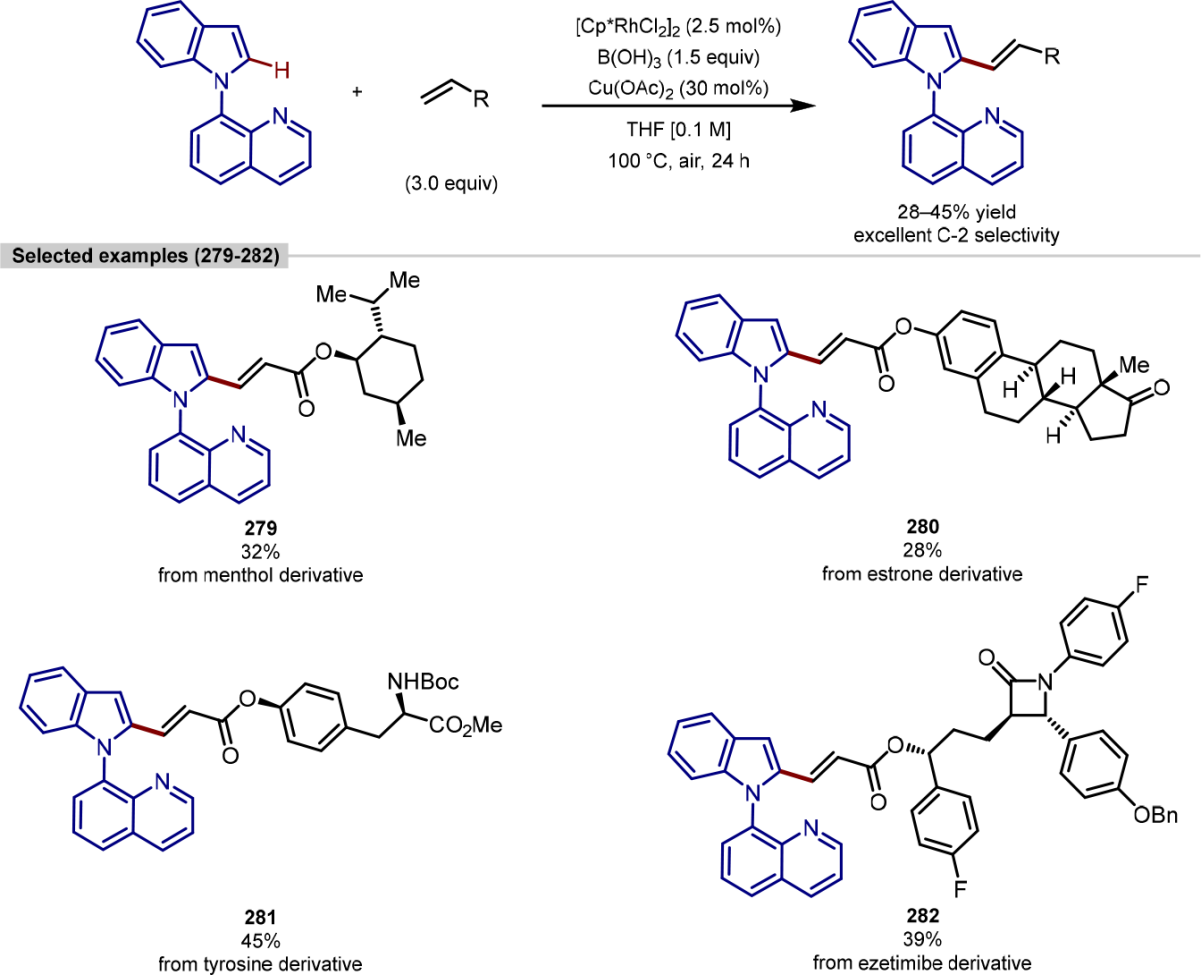
Rhodium-Catalyzed C-2 Alkenylation of *N*-(8-Quinolyl)indoles Using Bioactive and Drug-like Olefins

Zhang described the rhodium-catalyzed C-2 selective C–H alkenylation of indoles, using 8-quinoline as a directing group installed onto the nitrogen of the indole starting material (Scheme 60).^230^ The reaction was successful with different olefins such as acrylates, phenyl vinyl sulfone, diethyl vinyl phosphonate, *N*,*N*-dimethylacrylamide or styrene. However, when they used enones and slightly modified reaction conditions, they obtained the C-2 alkylated products instead. The reaction with alkenes derived from menthol, estrone, tyrosine and β-lactone-based drug afforded the corresponding alkenylated products **279**–**282** with low and moderate yields (28–45%) and total C-2 selectivity.

### 5.2 *meta*-Alkenylation

**Scheme 61:**
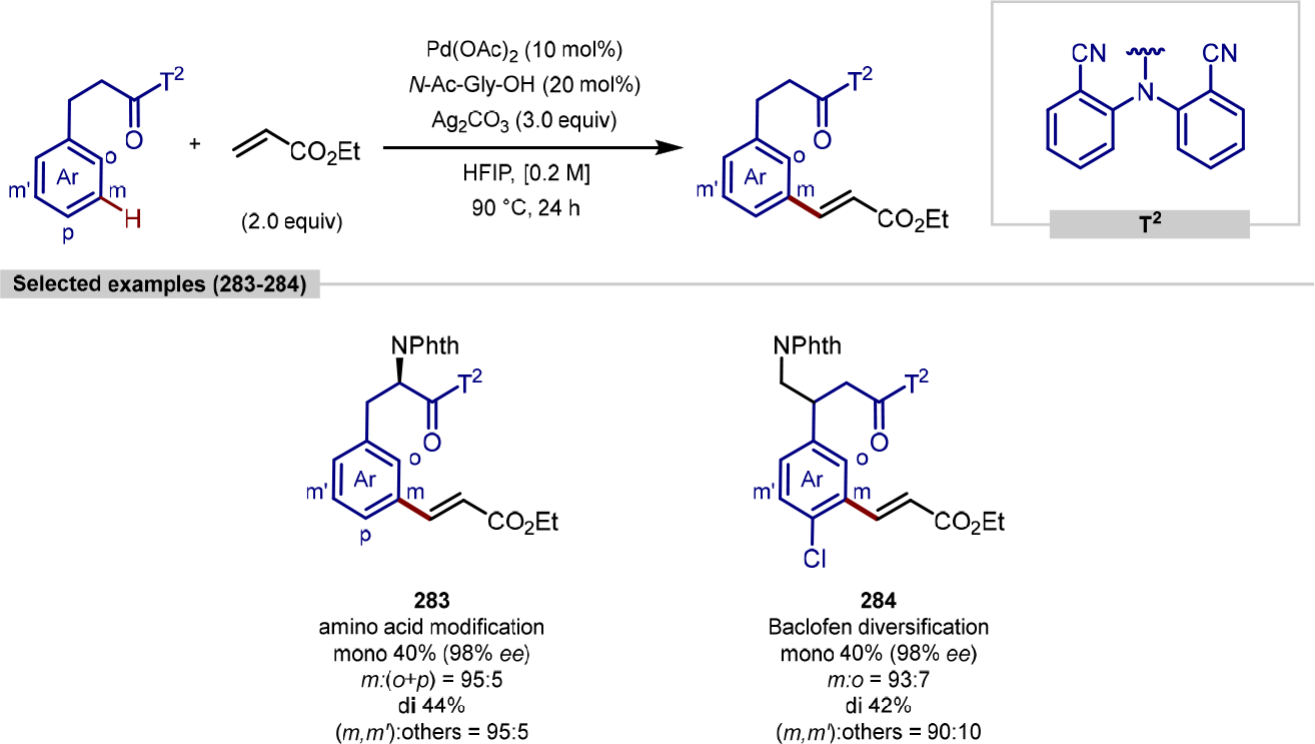
Palladium-Catalyzed *meta*-Selective Alkenylation of Arenes Using End-On Nitrile Directing Groups

Yu described in 2012 the first protocol for the palladium-catalyzed *meta*-selective alkenylation of arenes (Scheme 61).^231^ The remote functionalization was achieved with the use of end-on nitrile-based directing groups. Good results were obtained with toluene derivatives (with the directing group linked to the methyl group) with different substitution in *ortho-*, *meta-*, and *para-*positions of the arene, using mono- and disubstituted alkenes. Specifically, for the more complex functionalizations, *N*,*N*-bis(2-cyanophenyl)amides were used as directing groups. The use of two equivalents of ethyl acrylate, 10 mol % of Pd(OAc)_2_ as a catalyst, 20 mol % of *N*-acetyl glycine as a ligand, and three equivalents of silver carbonate as an oxidant, in HFIP at 90 °C, after 24 h gave the alkenylated products of an unnatural amino acid **283** and baclofen **284** with excellent regioselectivity in the *meta*-position but with only moderate monoselectivity.

**Scheme 62:**
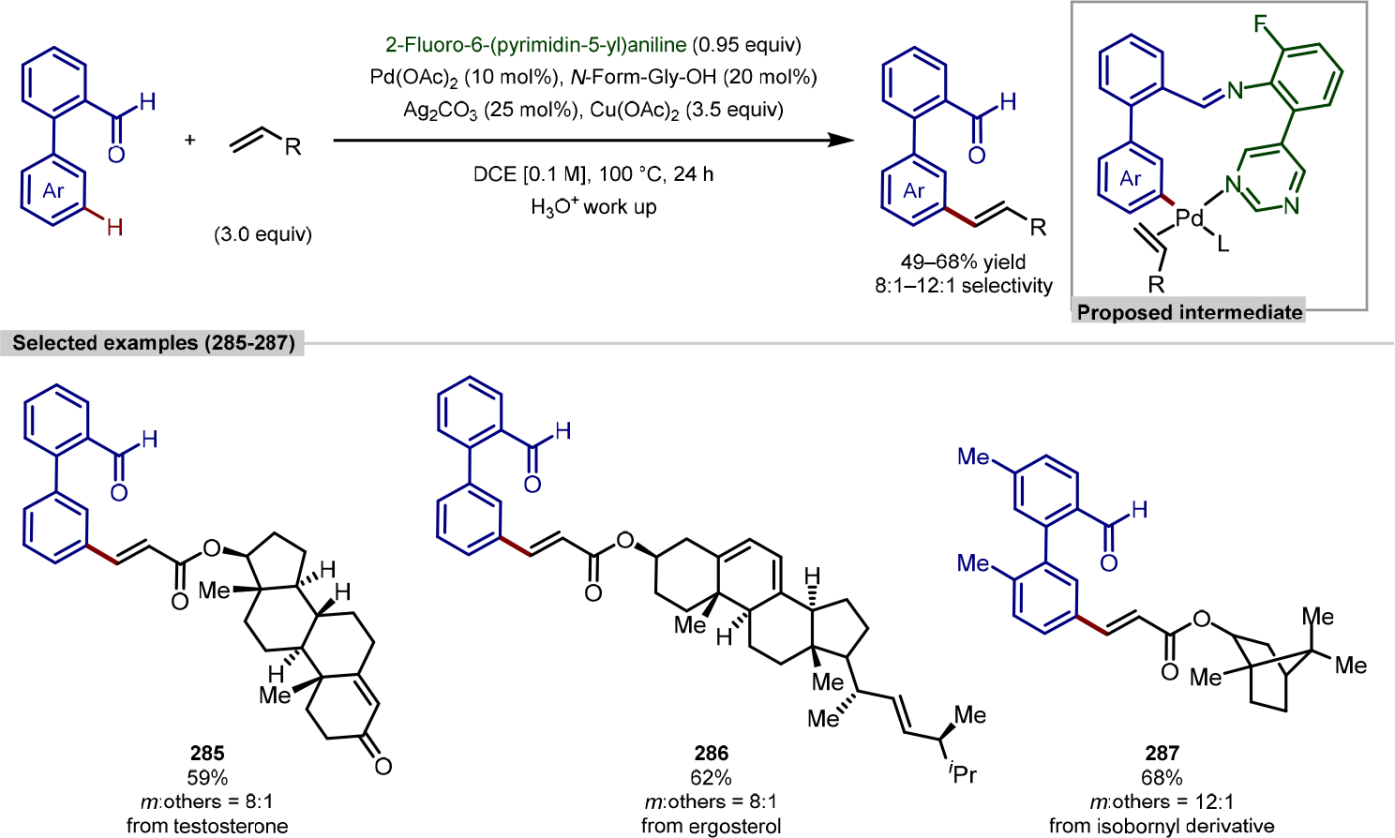
Palladium-Catalyzed *meta*-Selective C–H Alkenylation of 2-Arylbenzaldehydes Using an Imine Temporary Directing Group

For *meta*-selective alkenylations, Maiti described in 2021 the use of imines as temporary directing groups for the palladium-catalyzed *meta-*selective alkenylation of 2-aryl benzaldehydes (Scheme 62).^232^ After an exhaustive screening of amines they found that the best candidate for the reaction was 2-fluoro-6-(pyrimidin-5-yl)aniline. The fluorine substituent in the aniline ring was observed to be critical to avoid undesired *ortho-*olefination products, and the use of pyrimidine coordinating group enabled strong coordination with the palladium catalyst, which aided the C–H activation. In this case, unlike Yu’s *meta*-selective alkenylation, the high complexity coupling partner came from the alkene. The use of three equivalents of testosterone, ergosterol and isobornyl alcohol-derived alkenes in the olefination of 2-arylbenzaldehyde, along with 10 mol % of Pd(OAc)_2_ as a catalyst, 20 mol % of *N*-formyl glycine and 25 mol % of silver carbonate as additives, 3.5 equiv of Cu(OAc)_2_ as an oxidant, in DCE, at 100 °C, afforded the desired products **285**–**287** in good yields (59–68%).

**Scheme 63:**
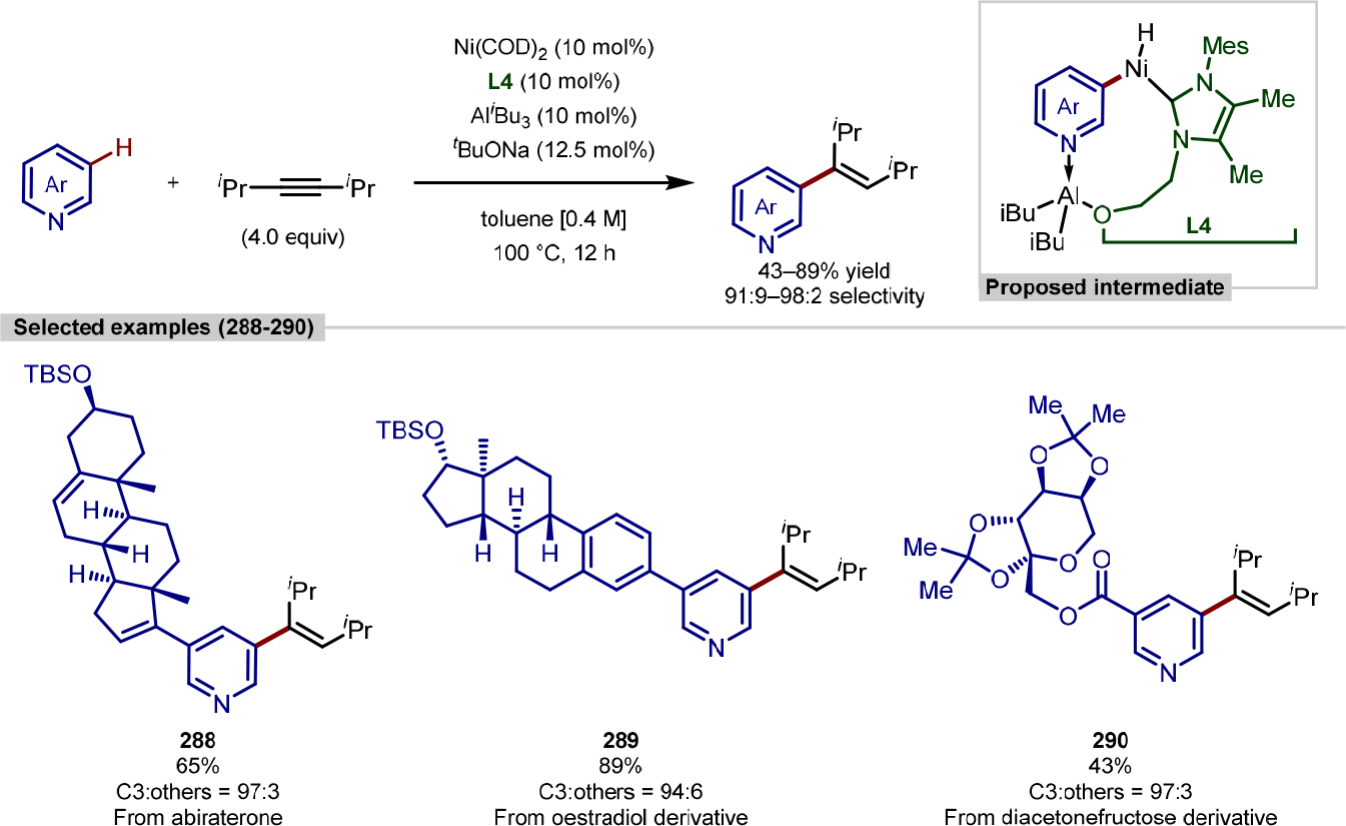
Nickel-Catalyzed C-3 Selective C(sp^2^)–H Alkenylation of Pyridine-Based Bioactive Molecules and Drugs

Yu additionally described the nickel-catalyzed C-3 alkenylation of pyridines using a bifunctional NHC ligand to overcome C-2 and C-4 selective alkenylation (Scheme 63).^233^ Using this strategy, the NHC ligand bearing a hydroxyl-group in its structure was proposed to coordinate with a Lewis acid (diisobutylalkoxyaluminum) that is also coordinated with the nitrogen of the pyridine starting material. At the same time, the NHC ligand is coordinated with the nickel catalyst favoring the activation in C-3 of the pyridine ring. C-2 Functionalization was blocked *via* repulsion between the ligand and the Lewis acid and C-4 activation was avoided by modifying the ligand linker length. Following this protocol, nine different high-complexity functionalizations were achieved with moderate to excellent yields and excellent C-3 selectivity.

### 5.3 *para*-Alkenylation

**Scheme 64:**
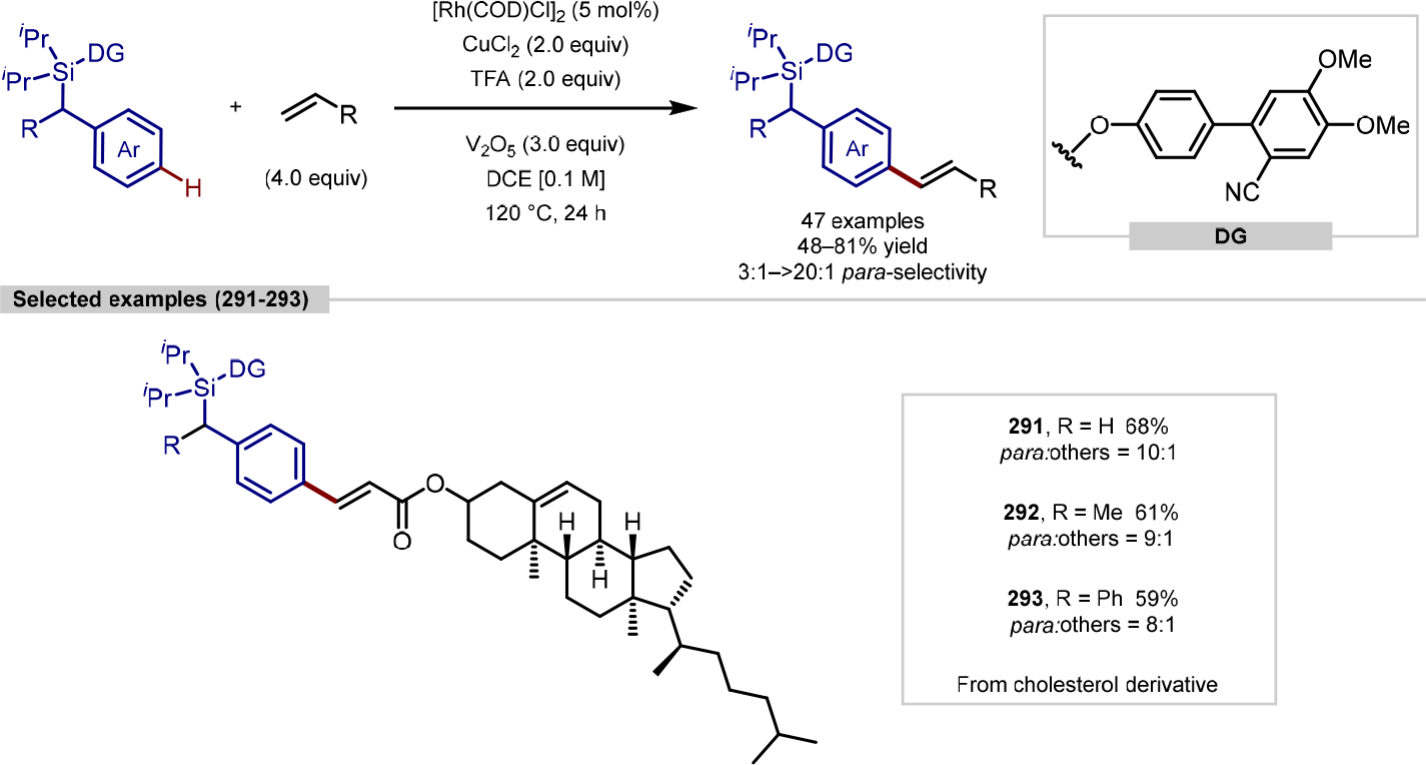
Rhodium-Catalyzed *para*-Selective C–H Alkenylation of Cholesterol Derivatives

The first rhodium-catalyzed *para*-selective alkenylation of arenes was described by Maiti in 2019 (Scheme 64).^234^ To achieve this challenging *para*-selectivity, a silicon-linked electron-rich cyano-biphenyl traceless directing group was used in toluene derivatives. The best conditions were obtained using 5 mol % of Rh(COD)Cl dimer as a precatalyst, the combination of two equivalents of CuCl_2_ and TFA as an oxidant (forming Cu(TFA)_2_*in situ*), three equivalents of V_2_O_5_ as co-oxidant, and four equivalents of the olefin, in DCE at 120 °C. A broad selection of substrates was applicable (47 examples) with modest to good yields and overall good selectivity. The late-stage alkenylation of three cholesterol-based molecules were additionally described with good yields (59–61%) and good *para*-selectivity (8:1–10:1).

### 5.4 Heteroarene-Alkenylation

More recently, other interesting approaches in the alkenylation of arenes and heteroarenes have emerged without the use of directing groups but obtaining high levels of regioselectivity. The key feature for these protocols was the use of specific ligands that coordinate the metal catalyst, accelerate the C–H activation reaction and increase the selectivity. Another key feature to note is the role of electronic control for selectivity in the reaction.

**Scheme 65:**
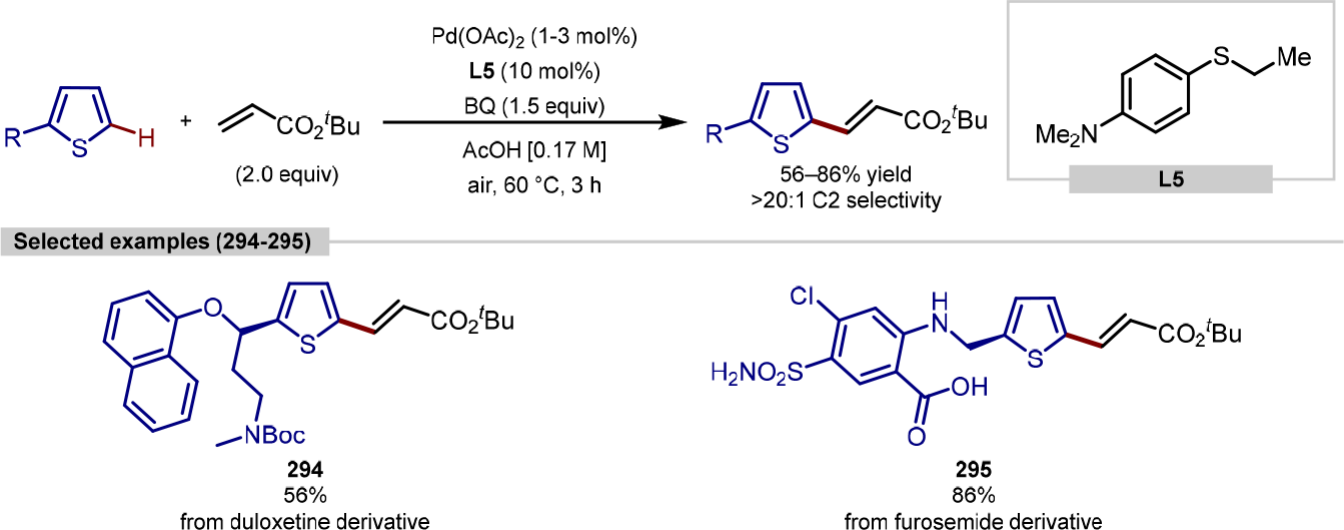
Pd-Catalyzed C-2 Selective C–H Alkenylation of Thiophenes

**Scheme 66:**
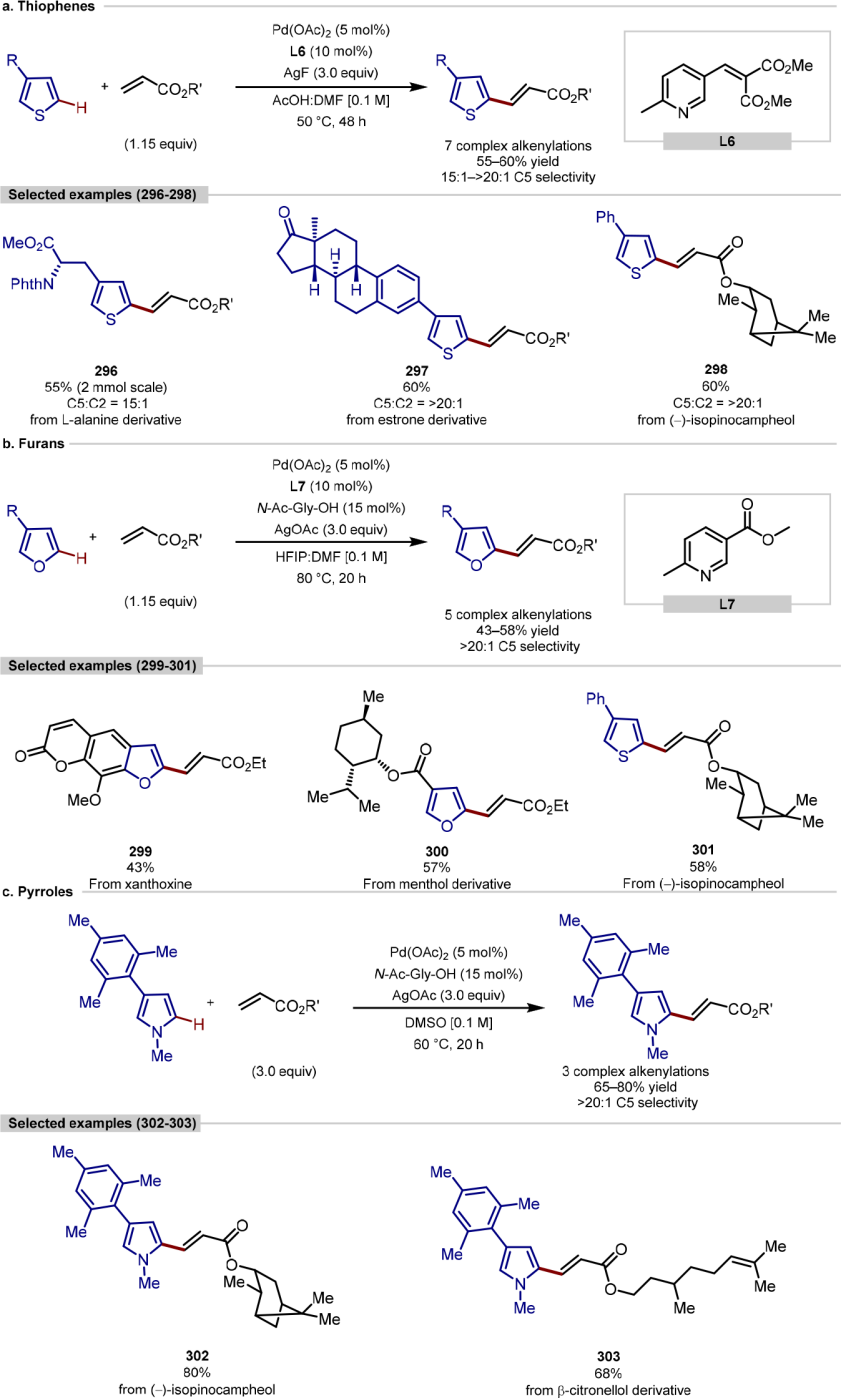
Pd-Catalyzed C-2-Selective C–H Alkenylation of Thiophenes, Furans, and Pyrroles

In 2017, Carrow used this approach in the palladium-catalyzed C-2 selective alkenylation of heteroarenes (Scheme 65).^235^ The use of thioether ligands accelerated this reaction when compared the ligand-free system and other commonly used ligands for C–H olefinations like pyridine, amino acids or triphenylphosphine. In a detailed kinetic study, the authors observed the same yield with the thioether ligand **L5** after 8 min than with the other ligands and ligand-free reaction in three hours. The rate-enhancement was attributed to a change from a neutral to a cationic pathway because of the thioether coordination to the palladium center, with C–H bond cleavage being the rate-determining step for this reaction. In addition, the formation of a cationic, low-coordinate catalytic intermediate was determined to be responsible for the observed electronic controlled site selectivity. In terms of the alkenylation of complex substrates, the reaction of duloxetine and furosemide derivatives with two equivalents of *tert*-butyl acrylate, 1–3 mol % of Pd(OAc)_2_ as a catalyst, 1.5 equiv of benzoquinone as an oxidant, and 10 mol % of 4-(ethylthio)-*N*,*N*-dimethylaniline as a key ligand, in acetic acid under air at 60 °C, afforded the desired olefination products **294** and **295** in good to excellent yields.

van Gemmeren later used a similar strategy for the palladium-catalyzed C-5 selective alkenylation of 3-substituted 5-membered heteroarenes. Using the heterocycle coupling partner as the limiting reagent, the development of alkenylation reactions of valuable heterocyclic bioactive compounds could be achieved (Scheme 66).^236^ In this case, 6-methyl-3-substituted pyridines were used as ligands to obtain good results in terms of selectivity and reactivity. This selectivity may be due to steric factors relative to electronic effects or by suppressing a weak directing effect by the electron-poor α,β-unsaturated ester.^237^ For the alkenylation of thiophenes, 5 mol % of palladium acetate was used as catalyst, 10 mol % of 3-malonate-6-methylpyridine derivative (**L6**) was used as a ligand, three equivalents of silver fluoride as an oxidant, in AcOH:DMF mixture. For the alkenylation of high complexity substrates, several examples were described (7 examples, 55–60% yield), with the bioactive molecule present both in the thiophene and alkene coupling partners (Scheme 66a). Contrastingly, in the reaction with electron-poor thiophenes and furans, 5 mol % of palladium acetate was used as a catalyst, 10 mol % of methyl 6-methylnicotinate ligand (**L7**) was used, along with three equivalents of silver acetate as an oxidant, in a mixture of HFIP and DMF. In this case, 15 mol % of *N*-acetyl glycine was also used in the reaction, showing that a dual ligand system was necessary to obtain the desired products. A single electron-poor thiophene and six furan derivatives were obtained in the late-stage alkenylation, with total selectivity toward C-5 functionalization (Scheme 66b). Finally, for the alkenylation of pyrroles, the reaction proceeded with high levels of C-5 selectivity using only *N*-acetyl glycine as a ligand, 5 mol % of palladium acetate as a catalyst, and three equivalents of silver acetate as an oxidant in DMF. 3-Mesityl-*N*-methyl pyrrole was used in the late-stage olefination with four different alkenes bearing a bioactive component (Scheme 66c).

### 5.5 Undirected-Alkenylation

**Scheme 67:**
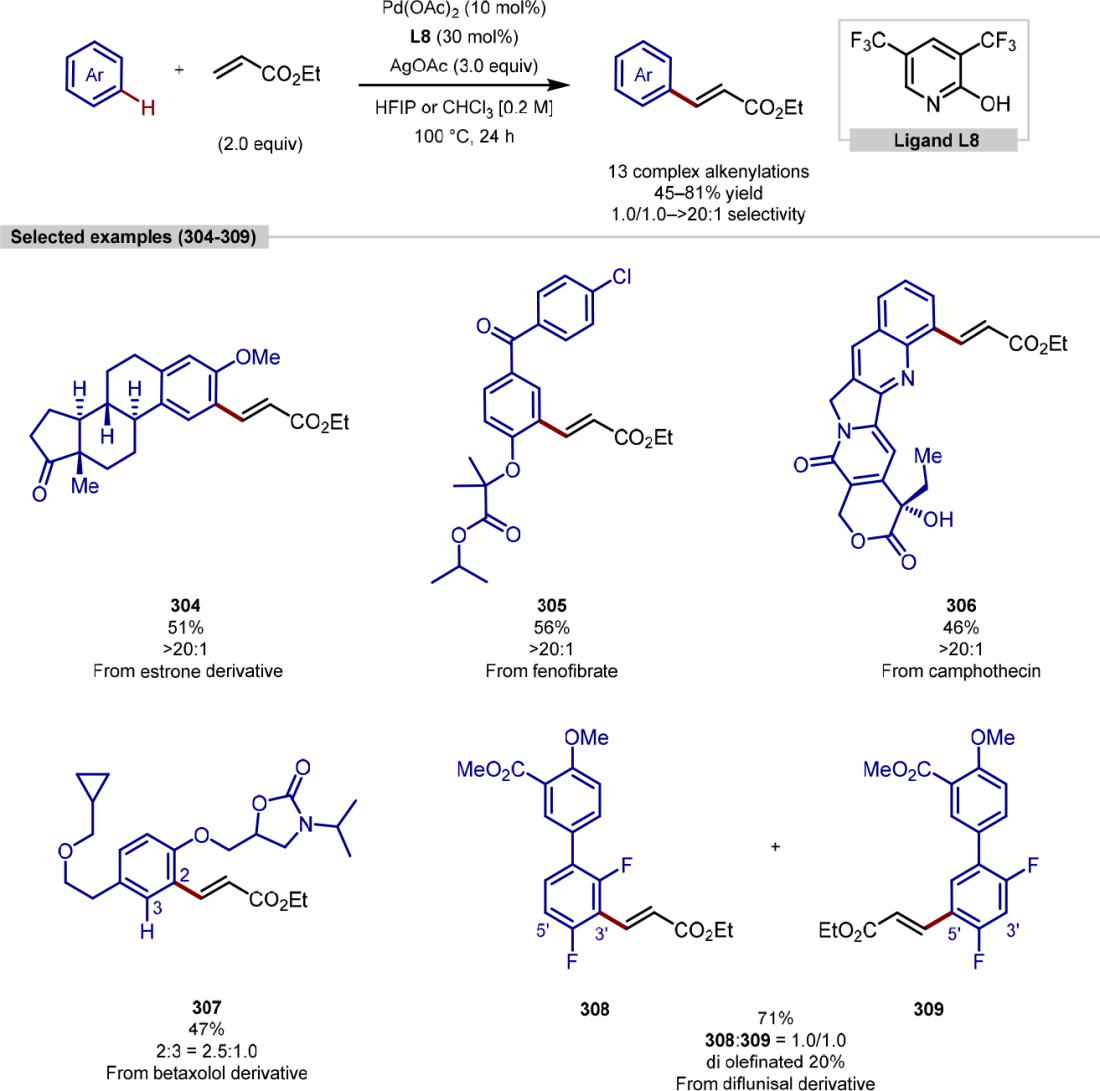
Undirected Pd-Catalyzed Late-Stage Alkenylation of Arenes

In 2017, Yu developed the undirected C–H alkenylation of arenes using 2-hydroxy-3,5-bis(trifluoromethyl)pyridine **L8** as a key ligand for this transformation (Scheme 67).^238^ This reaction proceeded at elevated temperatures (100 °C) and required an excess of AgOAc as an oxidant for catalyst turnover. In an extensive testing of reaction conditions using unique arenes as starting materials, 2-hydroxypyridine ligands bearing electron-withdrawing groups gave the best results in terms of reactivity and selectivity, with **L8** being the best candidate. To illustrate the crucial role of **L8** in the reaction, when *o-*xylene was used as starting material, the yield increased from 12% to 83% using **L8**, and the reaction selectivity increased from 4.4/1 to complete regioselectivity. A broad selection of simple arenes and heteroarenes (53 examples) were successfully alkenylated with ethyl acrylate using this protocol, with good to excellent yields (32–88%) and selectivities from 1.0:1.0 to >20:1. Halides, aldehydes, ketones, and esters were tolerated in the reaction; however, unprotected amines or alcohols were not tolerated under the reaction conditions. Using *o-*xylene as a starting material, a wide variety of alkenes could be used in the reaction, including vinyl sulfones, acrylates acrylamides, or vinyl phosphonates. This method was extended to the functionalization of 13 natural product and drug-type targets with moderate yields (45–81%) and with variable selectivity depending on the substitution and electronic nature of the functionalized arene. This undirected C–H alkenylation provides a synthetic procedure without the need of directing groups or prefunctionalization of the starting materials as halides or pseudo halides, allowing the alkenylation of complex and potentially interesting molecules.

**Scheme 68:**
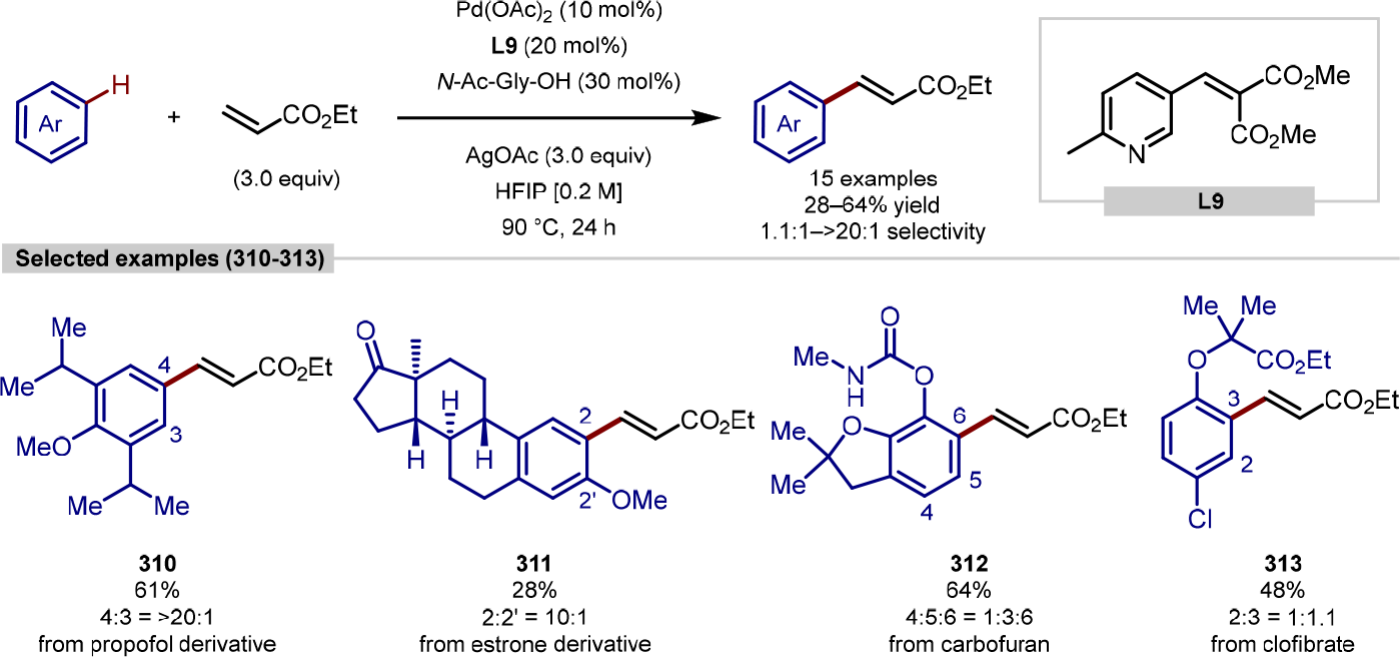
Undirected Pd-Catalyzed C-2 Selective C–H Alkenylation of Arenes

In 2021, van Gemmeren described the palladium-catalyzed undirected alkenylation of arenes using a dual ligand system (Scheme 68).^239^ This work was an extension of the method developed by the same research group in 2018 for simple arenes, in which the combination of 6-methyl-3-(dimethylmalonate)pyridine (**L9**) and *N*-acetyl glycine as ligands could facilitate the concerted metalation-deprotonation step, without the use of directing groups. In addition, it was proposed that this dual ligand–Pd system could be capable of overriding weak coordinating effects previously reported in direct arene C–H activation protocols, obtaining alkenylated products with complementary selectivity. Akin to the work from Yu (Scheme 67), three equivalents of silver salt were used as an oxidant in this reaction. In this work, 15 complex alkenylations with ethyl acrylate were described, with moderate to good yields and moderate to excellent regioselectivities.

**Scheme 69:**
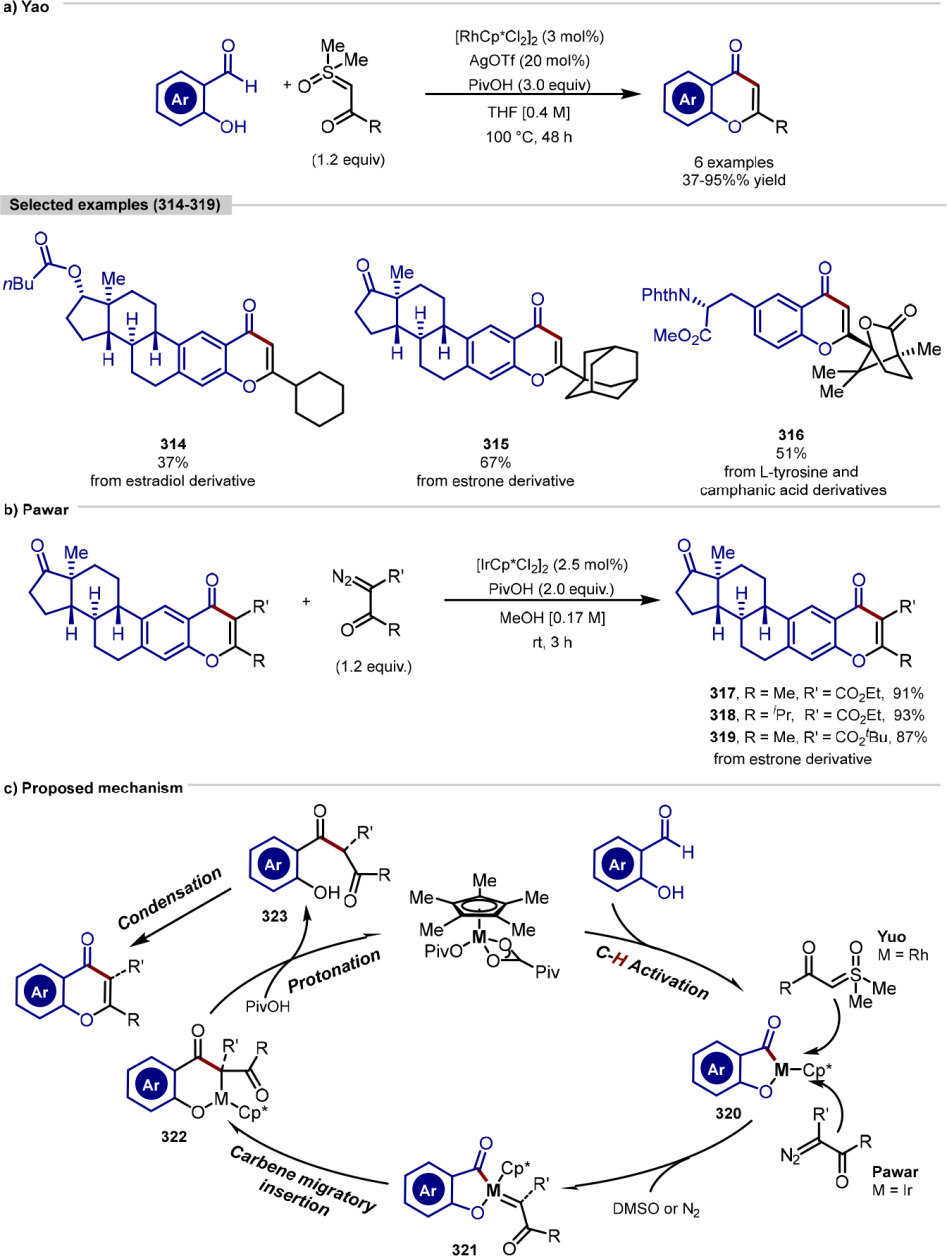
Yao and Pawar’s C–H Alkenylation of Salicylaldehydes in the (sp^2^)C(O)–H Bond

All the precedents in this section so far have described the C(sp^2^)–H alkenylation of arenes and heteroarenes. In 2019, Yao and Pawar described the Rh(III)^240^ and Ir(III)^241^ C–H activation/annulation of salicylaldehydes to obtain chromones, activating the aldehyde C–H bond (Scheme 69). These methods constitute a formal alkenylation reaction of the starting materials, but the alkenylated product was formed via C–H alkylation followed by an intramolecular condensation. Sulfoxonium ylides were used by Yao for the alkylation reaction (Scheme 69a). Mechanistically, the reaction was proposed to proceed by the attack of the nucleophiles to the metal center of the rhodacycle **320** formed after the C–H activation step (Scheme 69c). This intermediate evolved *via* elimination of DMSO to afford a carbene species **321**. Subsequent migratory insertion of the Rh–C bond into the carbene and protonation gave the alkylated product which formed the final chromone in an intramolecular dehydrative condensation. In the case of Pawar’s work, the same type of mechanism was described, but using α-diazocarbonyl compounds as alkylating reagents (Scheme 69b). In this case, after the coordination of the diazo compound to the metallacycle intermediate **320** and loss of N_2_, the same carbene intermediate **321** was formed, evolving to the final product in the same reaction mechanism. The most complex functionalization in this work was limited to an estrone derivative with three different diazo-compounds in excellent yields (87–93%).

### 5.6 C–H Alkenylation in Peptides

A research area that has received more interest recently is the directed C–H alkenylation of peptides. In these works, two main approaches have been established: the use of protein backbones as an internal directing group, and the incorporation of additional functionalities that act as directing groups to obtain the desired alkenylation products.

**Scheme 70:**
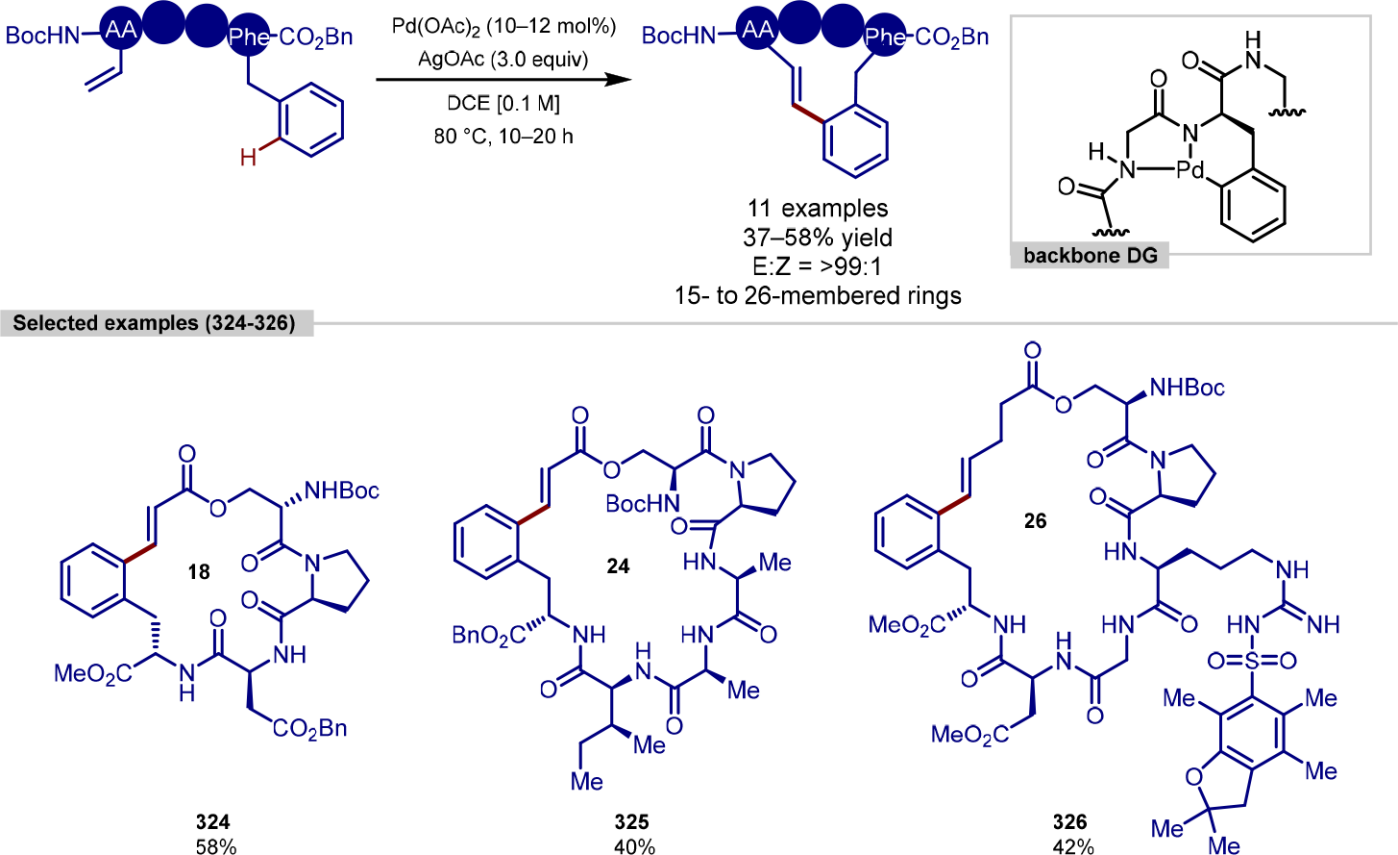
Pd-Catalyzed Directed C–H Macrocyclization of Peptides Using Protein Backbone as DG

Wang has developed a well-established research line in the late-stage alkenylation of peptides, including their macrocyclization. Similarly, in 2018 Wang described the Pd-catalyzed directed C–H alkenylation and macrocyclization of peptides (Scheme 70).^242^ This powerful strategy used peptide backbone amides terminal to a phenylalanine residue as directing groups to the *ortho-*selective C–H alkenylation in the aromatic ring of this amino acid. Using the established optimal conditions, three equivalents of silver acetate were used as an oxidant, in DCE at 80 °C. For the late-stage macrocyclization, the formation of nine macrocycles was described using acrylate-derived peptides in good yields forming 15-, 18-, 21-, and 24-membered macrocycles. In addition, two examples were described using challenging unactivated alkene-containing peptides, in good yields and forming 17- and 26-memebered macrocycles.

**Scheme 71:**
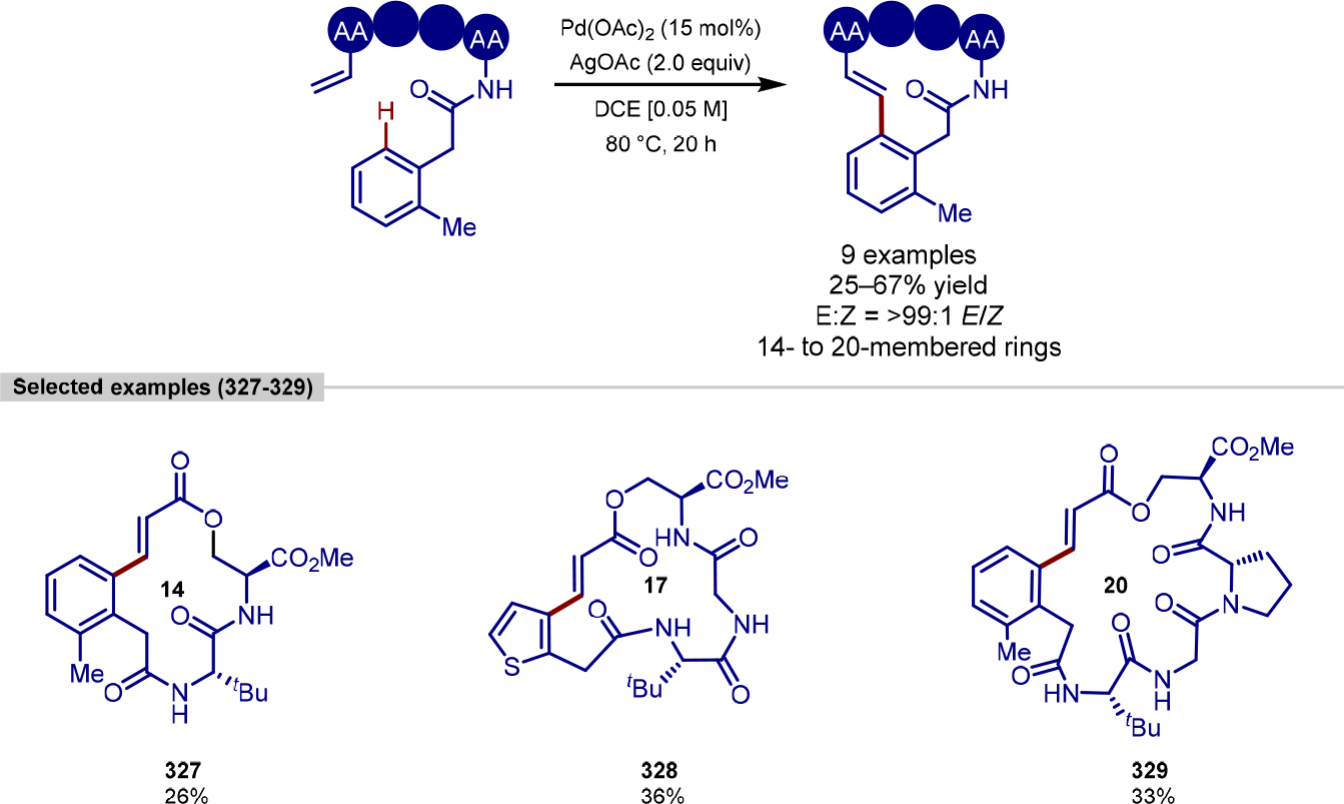
Pd-Catalyzed Directed C–H Macrocyclization of Peptidoarylacetamides

Using the same catalytic system, and backbone amides as internal directing groups, the authors described the palladium-catalyzed directed C–H alkenylation and macrocyclization of peptidoarylacetamides (Scheme 71).^243^ In this case, the aryl group used had one of the *ortho*-positions blocked to the acetamido- directing group, avoiding undesired bis-olefinated products. Nine macrocycles were obtained in the self-assembled alkenylation, with ring sizes between 14- and 20-atoms and yields between 26% and 67%, with one example in the alkenylation of the thiophene-based peptide in C-3.

**Scheme 72:**
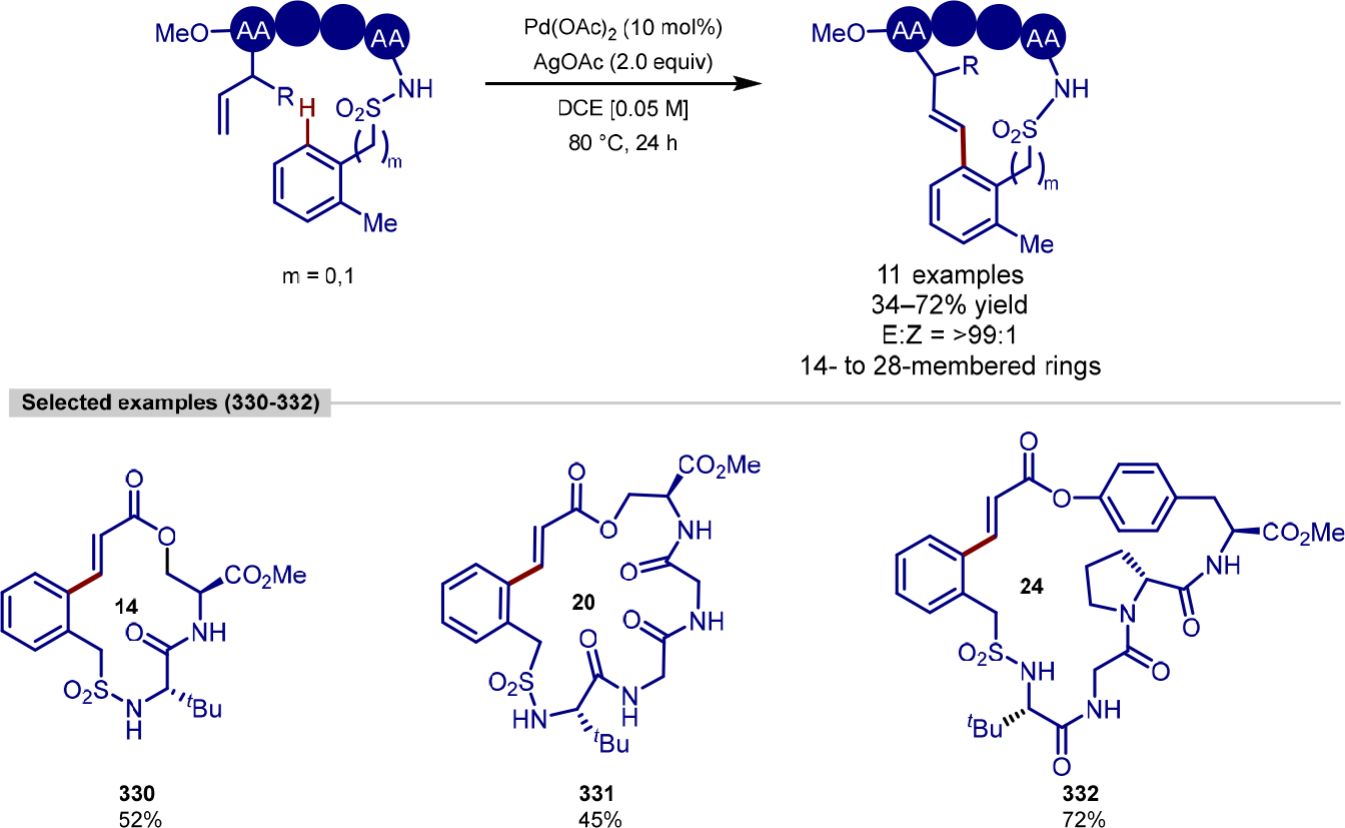
Pd-Catalyzed Directed C–H Alkenylation and Macrocyclization of Sulfonamido Peptides

Wang additionally reported the Pd-catalyzed C–H alkenylation and macrocyclization of peptides using, in this case, sulfonamides as directing groups (Scheme 72).^244^ This sulfonamide moiety is present in the peptide structure, acting as internal directing group. Before the development of the late-stage macrocyclization, the C–H alkenylation with an external alkene was studied, obtaining two types of products: 25 examples of the alkenylated product in a variable mono/bis- ratio, and 28 benzosultam derivatives. The authors proposed a second C–H activation in the olefinic C(sp^2^)–H bond to obtain the benzosultam products. The reaction tolerated EWG and EDG in *ortho-*, *meta-*, and *para-*positions of the aromatic ring and different olefins for both protocols. For the benzosultam formation, the reaction was successful with a large range of acrylates, dimethyl acrylamide, and ethyl vinyl ketone, but not with unactivated olefins. For the self-assembled macrocyclization, 11 macrocycles of 14- to 28-memebered ring sizes were obtained with good yields, including a 28-membered macrocycle with fluorescein isothiocyanate conjugated to a lysine residue. This macrocycle also contained an arginine, glycine, and aspartate (RGD) sequence that, in an appropriate cyclic structure, is reported to selectively bind to integrins. The incubation of U87MG cells (glioblastoma cell line overexpressing the αvβ3 integrin) with this cyclic peptide showed strong fluorescence staining, which corresponded to a binding affinity to the target integrin, demonstrating the applicability of this method to generate bioactive peptides.

**Scheme 73:**
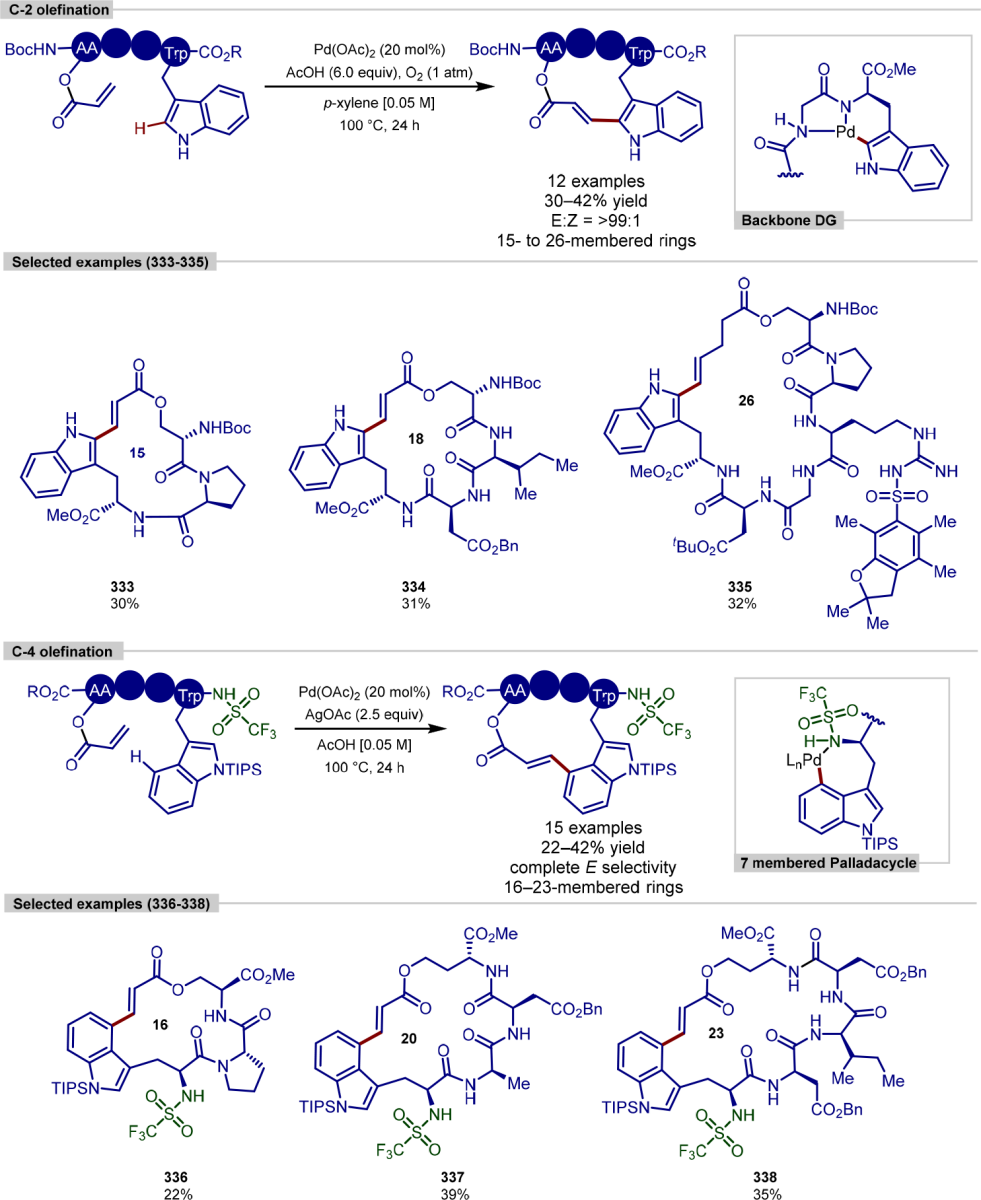
Pd-Catalyzed C-2 and C-4 Directed C–H Macrocyclization of Tryptophan-Derived Peptides

In 2020, the same research group described the macrocyclization of tryptophan-derived peptides in C-2 or C-4 positions *via* palladium-catalyzed C–H alkenylation (Scheme 73).^245^ To obtain the C-2 selective alkenylation products, the authors used their well-established protocol of using the protein backbone as directing groups. Additionally, they described the first example of peptide macrocyclization by palladium-catalyzed C–H alkenylation at the C-4 position using *N*-terminal trifluorosulfonamide as directing group. Significantly, this substitution pattern is challenging to obtain by classical lactonization protocols. The alkenylation in the C-2 position of different peptides afforded 12 macrocycles with ring sizes between 15- and 26-members with good yields for this type of cyclization (between 30% and 42%). In 11 of these examples, acrylate-based peptides were used and in one of them an unactivated olefin afforded the desired cyclic product in 32% yield. After the development of the intermolecular C-4 selective C–H alkenylation, including the homoligation of tryptophan using a bifunctional alkene, they extended the protocol to the macrocyclization of peptides. Fifteen examples of 15- to 23-memebered macrocycles were obtained in 20–42% yield, with complete C-4 selectivity.

**Scheme 74:**
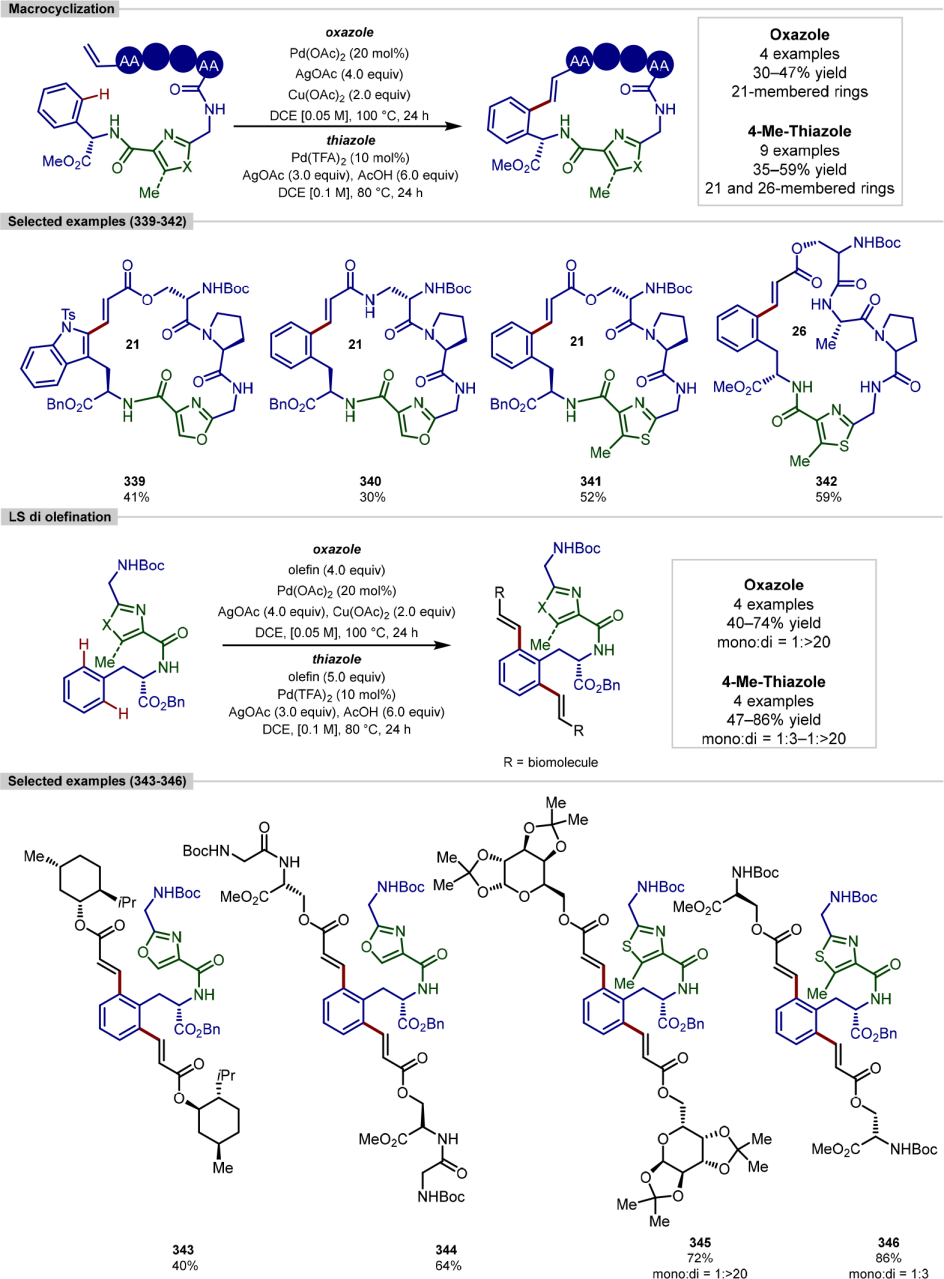
Pd-Catalyzed Directed C(sp^2^)–H Alkenylation and Macrocyclization of Oxazole and Thiazole-Derived Peptides

In 2021 and 2022, Wang developed the macrocyclization of peptides *via* palladium-catalyzed C–H alkenylation, bearing oxazole^246^ and thiazole^247^ in their structures (Scheme 74). These heterocycles served as directing groups, overcoming the backbone direction observed without the presence of these scaffolds. In the intermolecular reaction between oxazole-based peptides and an excess of acrylates, the dialkenylation reaction was developed, including four biomolecule-derived alkenes, using four equivalents of silver acetate and two equivalents of copper acetate as an additive, in DCE at 100 °C. Four structurally similar 21-memebered macrocycles were obtained with 30–47% yield, using the same catalytic system. These cyclic peptides showed strong cytotoxicity against the U87 cell line.

In the reaction with thiazole derivatives, after the development of the intermolecular version with variable mono/diselectivity (including the coupling with biomolecule-based acrylates), nine 21- to 25-memebered macrocycles were obtained in 35–59% yields. The authors used a similar catalytic system to the one used in the reaction with oxazoles: silver acetate as an oxidant, acetic acid as an additive and in DCE at 80 °C. Two of these macrocycles showed good bioactivity toward the U87 cell line.

**Scheme 75:**
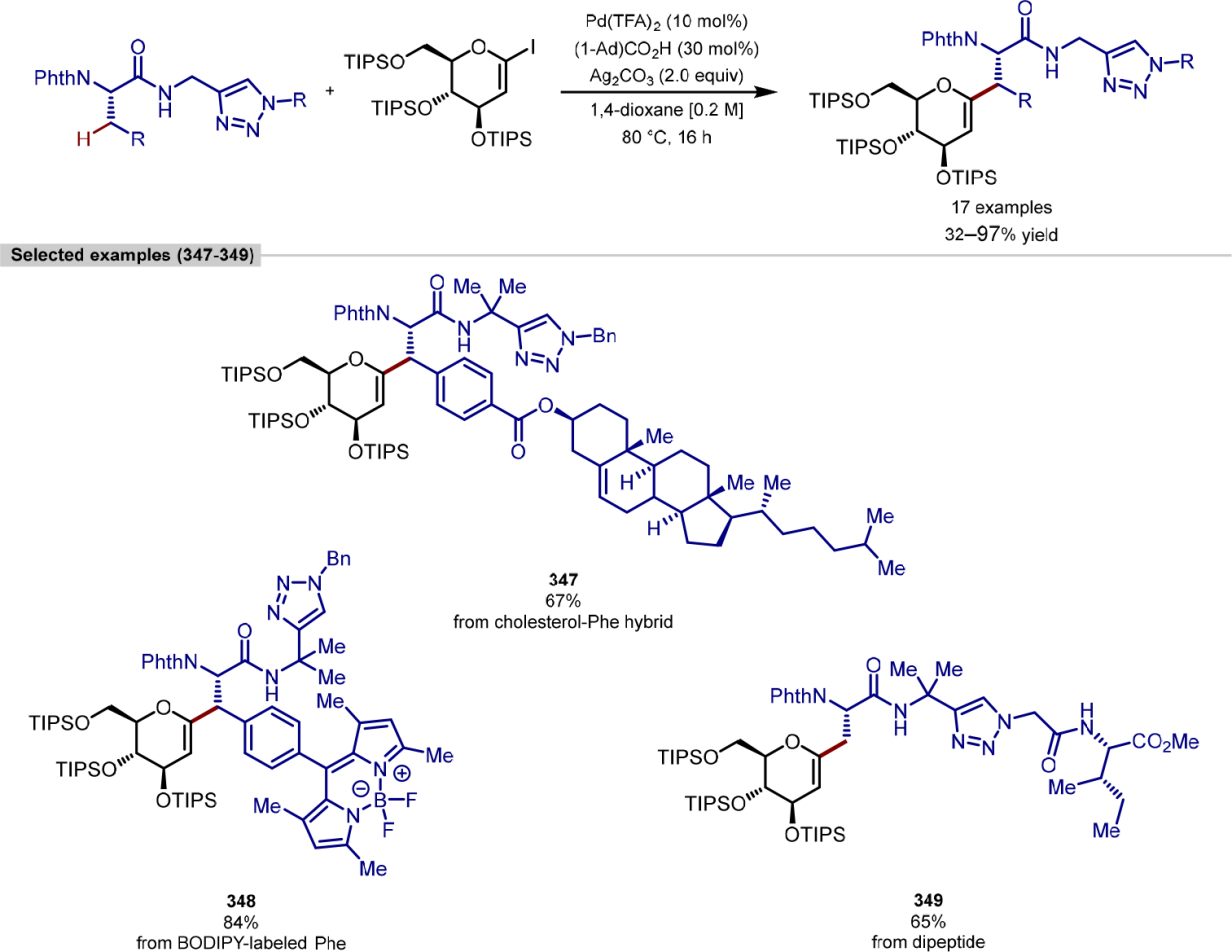
Pd-Catalyzed Directed C–H Glycosylation of TAM-Derived Peptides

Ackermann has recently developed several examples in the field of C–H alkenylation of peptides. In 2020, the Pd-catalyzed C(sp^3^)–H glycosylation of amino acid derivatives and peptides was described (Scheme 75).^248^ In this work, triazolydimethylmethyl amide (TAM) and 8-aminoquinoline were used as directing groups for simple phenylalanine derivatives, obtaining excellent results in reactivity and diastereoselectivity. Palladium trifluoroacetate was used as catalyst in this protocol, along with 30 mol % of 1-adamantyl carboxylic acid as an additive and two equivalents of silver carbonate as an oxidant and base, in 1,4-dioxane, at 80 °C. Ackermann reported that TAM could be used as a directing group in the terminal position for the glycosylation of terminal peptides and hybrids with biologically active molecules (such as cholesterol or menthol) in moderate to good yields. When the TAM directing groups were present in the amino acid chain structure, the unprecedented internal glycosylated peptides were obtained in 50–66% yield. In addition, considering the broad importance of BODIPYs as biocompatible fluorescence probes, BODIPY-labeled amino acids were successfully glycosylated, obtaining 5 different molecules in 57–97% yield.

**Scheme 76:**
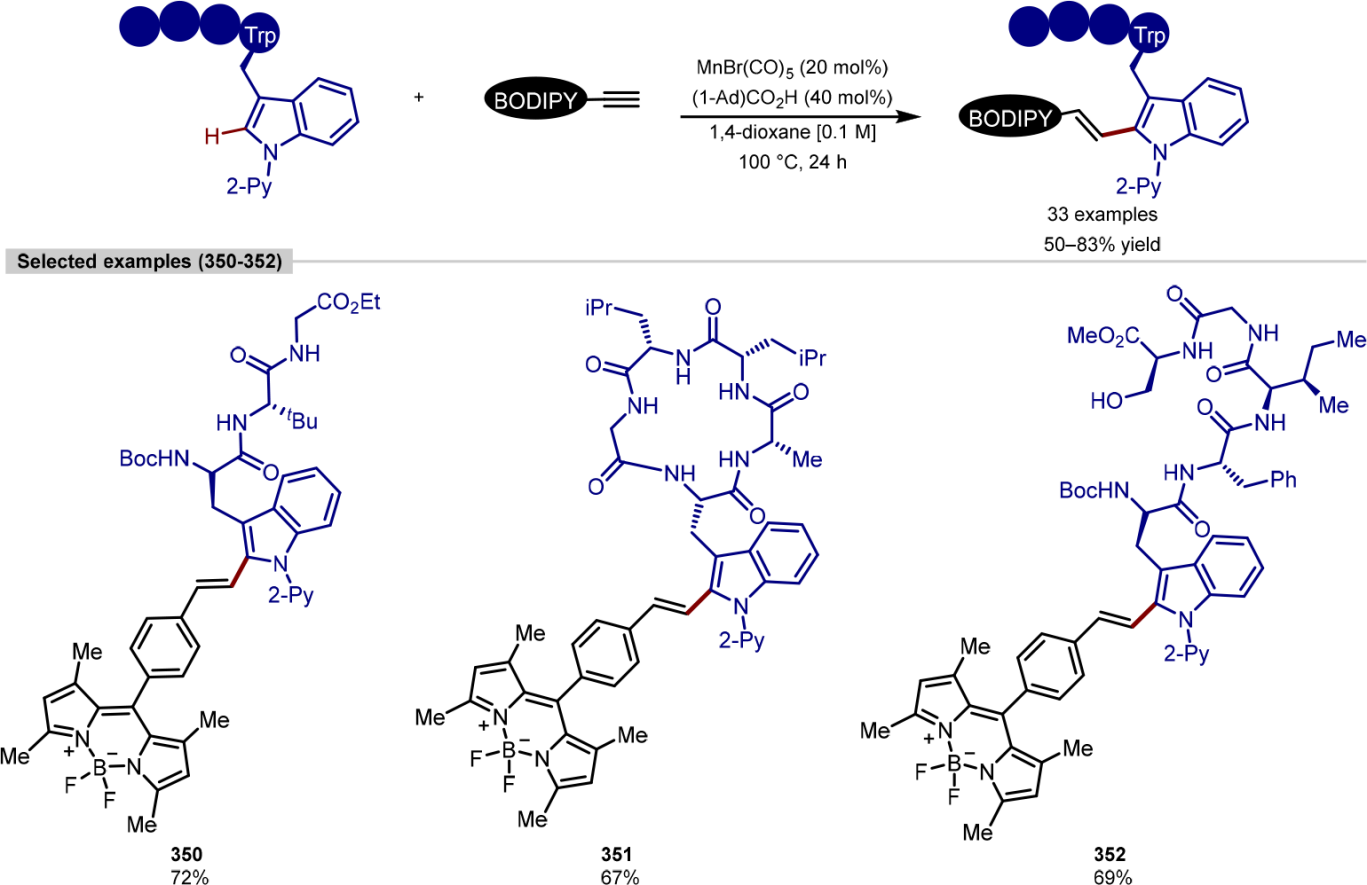
Mn-Catalyzed Directed C(sp^2^)–H Hydroarylation of BODIPY-Derived Alkynes

Ackermann additionally reported the manganese-catalyzed C–H alkenylation of peptides selectively in the C-2 position of tryptophan amino acid (Scheme 76).^249^ These are important examples of formal alkenylation *via* hydroarylation strategies, which allow for the use of alkynes as coupling partners. Using a Mn(I) precatalyst, the reaction between *N*-(2-pyridine) tryptophan peptides and BODIPY-labeled alkynes was discovered and developed, and the application for their use in fluorescence imaging probes was demonstrated. The best conditions for this reaction were the use of 20 mol % of MnBr(CO)_5_ as catalyst and 40 mol % of 1-adamantyl carboxylic acid as additive, in 1,4-dioxane, to obtain the alkenylated products. In the alkenylation scope, good-to-excellent results were obtained both with simple amino acids and in the late-stage functionalization: tri-, tetra-, penta-, and cyclic peptides are tolerated showing a high functional group tolerance, including disulfide scaffold.

**Scheme 77:**
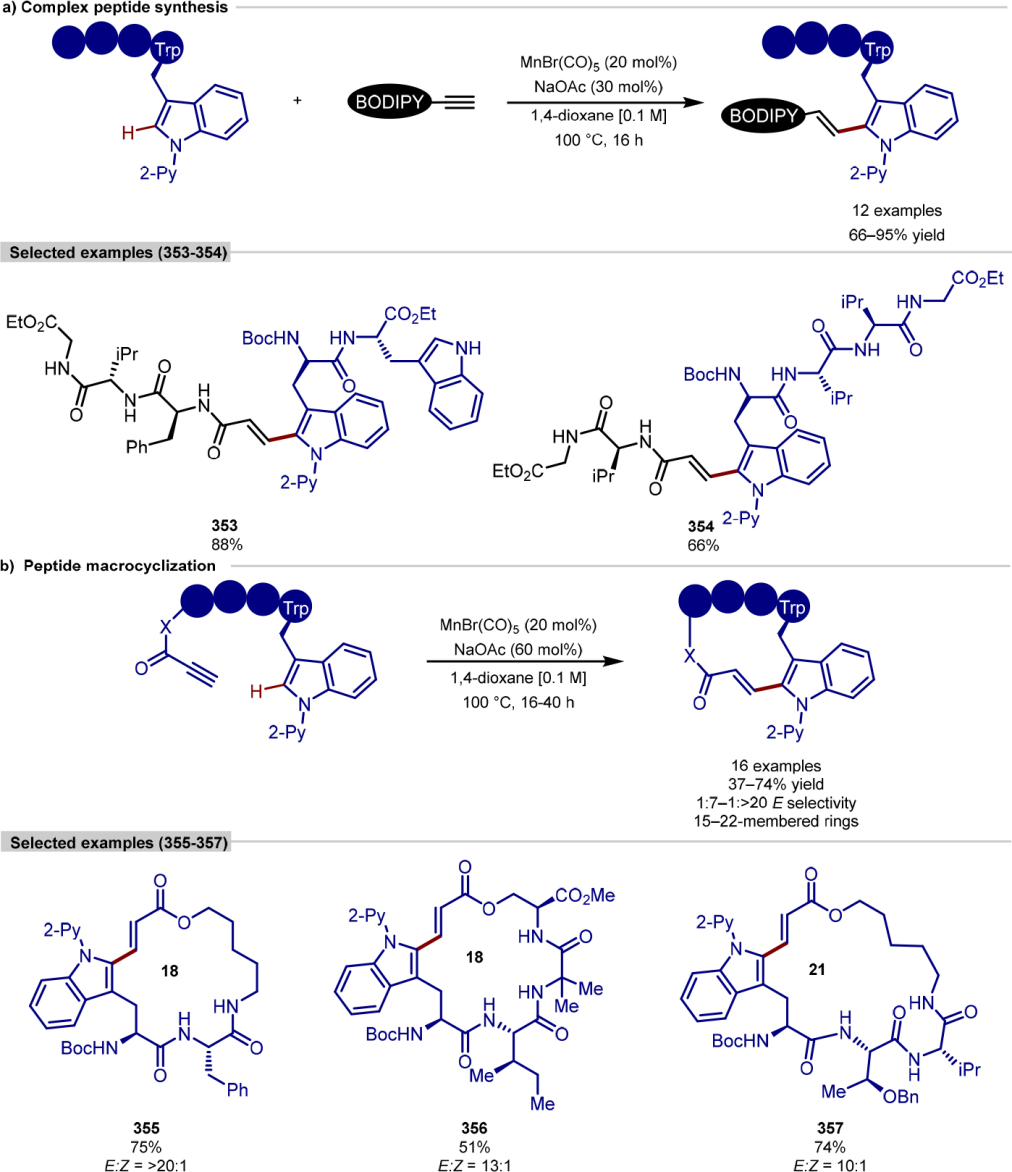
Mn-Catalyzed C-2 Directed C(sp^2^)–H Alkenylation of Tryptophan Derivatives Using Bioactive Molecule-Derived Alkynes (a) and Macrocyclization Reaction (b)

Using the same manganese catalyst and changing the additive to sodium acetate, Ackermann described the Mn(I)-catalyzed hydroarylation of alkynes using *N*-(2-pyridine)tryptophan derivatives (Scheme 77).^250^ Complex peptides containing a tryptophan coupling partner were applied giving the product with yields of 65–88%, including peptides with another tryptophan unit that regioselectively afforded products through the use of *N*-(2-pyridine) as a directing group. The reaction was also tolerant of biomolecule-derived alkynes, obtaining five mixed tryptophan derivatives in 72–95% yield. In addition, the self-assembled macrocyclization was described for the formation of 16 cyclic peptides that contained 15–22-member ring sizes in 37–74% yield. Finally, two of the cyclic peptides were studied in an anticancer experiment against HCT116 cells, showing considerable anticancer activities.

**Scheme 78:**
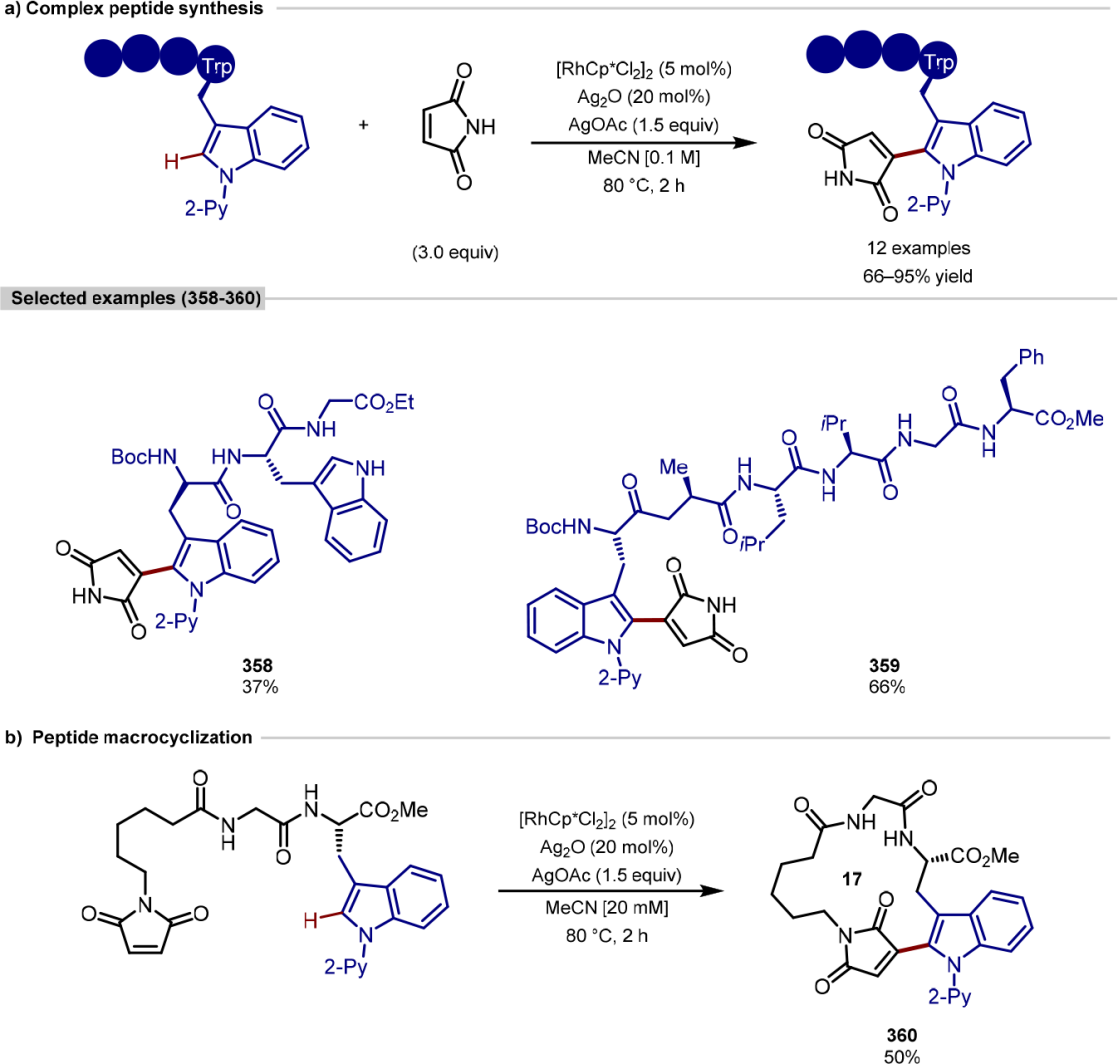
Rh-Catalyzed C-2 Directed C(sp^2^)–H Olefination of Tryptophan Derivatives (a) and Macrocyclization Reaction (b)

Using the same approach with 2-pyridine as a directing group for the C-2 alkenylation of tryptophan, Liu described in 2020 the rhodium-catalyzed alkenylation of this amino acid derivative with maleimides (Scheme 78).^251^ [RhCp*Cl]_2_ (5 mol %) was used as a catalyst, along with three equivalents of maleimides as olefin partners, 20 mol % of silver oxide as an additive, 1.5 equiv of silver acetate as an oxidant, in acetonitrile (0.1–0.2 M) at 80 °C. Terminal and internal *N*-(2-pyridine)tryptophan showed reactivity in the alkenylation of tri- to hexapeptides, with good results even with another indolic NH unprotected tryptophan in the structure. In addition to the alkenylation of complex peptides, the macrocyclization using maleimide-derived peptides was described, obtaining 18- and 20-membered ring cyclopeptides in 50% and 55% yield, respectively, and a dimer macrocyclic peptide in 32% yield. For the macrocyclization reaction, it was found to be necessary to decrease the concentration 10-fold to obtain good results.

**Scheme 79:**
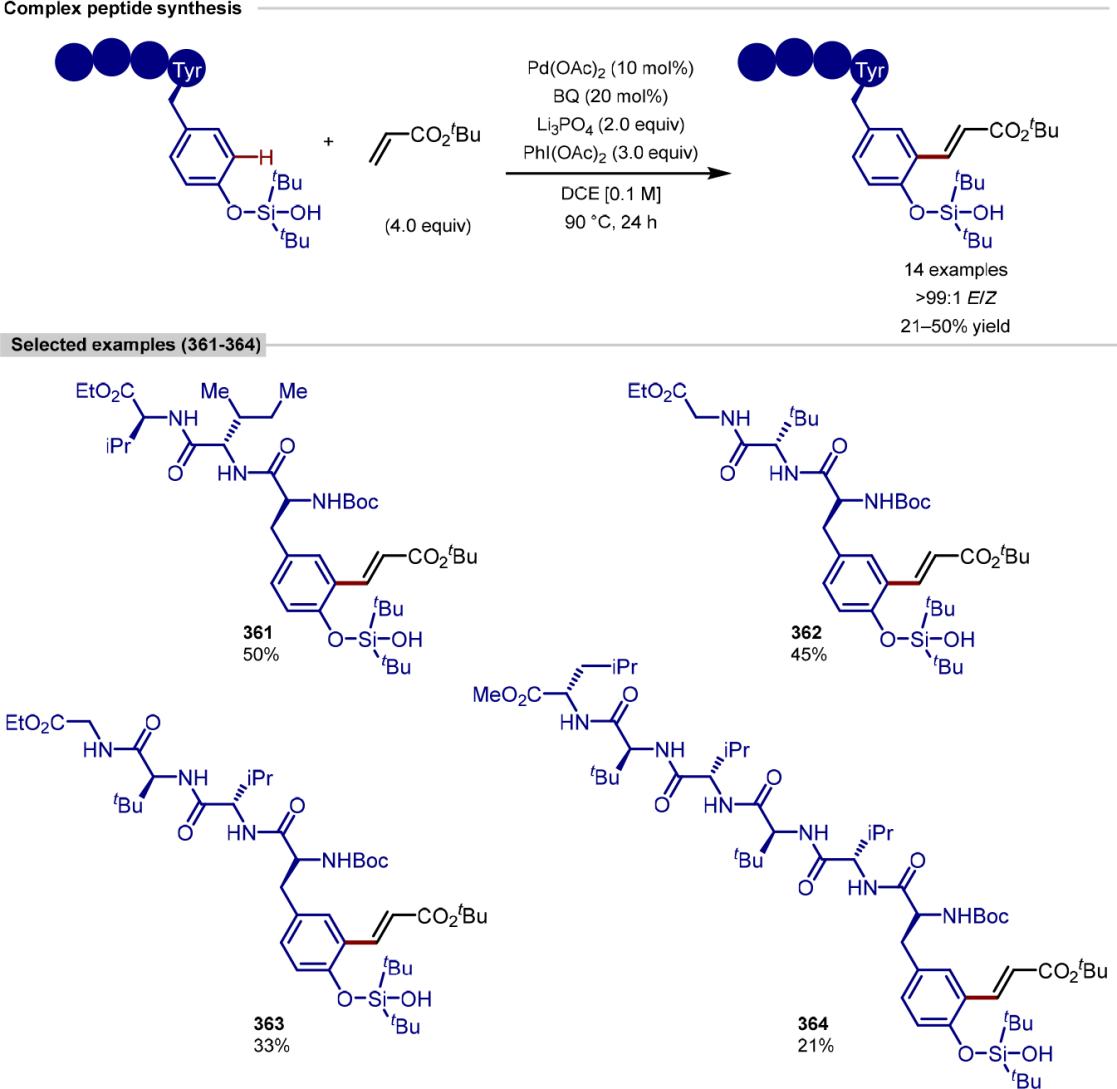
Pd-Catalyzed *ortho*-C(sp^2^)–H Alkenylation of Tyrosine Derivatives Using Silanol as Protecting and Directing Group

Using di-*tert*-butyl silanol as a protecting and directing group, Xiong described in 2020 the palladium-catalyzed *ortho*-alkenylation of tyrosine derivatives and tyrosine containing di- and complex peptides (Scheme 79).^252^ Considering the backbone direct alkenylation described for phenylalanine derivatives (Scheme 70), this protocol allowed for a complementary substitution pattern to these methodologies in the *meta-*site to the peptide chain. The best conditions for this reaction were obtained with the use of 10 mol % of Pd(OAc)_2_ as a catalyst, three equivalents of (diacetoxyiodo)benzene as an oxidant, two equivalents of lithium phosphate as a base, and 20 mol % of benzoquinone as an additive, in DCE (0.1 M) at 90 °C. The authors suggest that the use of benzoquinone could suppress the formation of palladium black, increasing the yield of the reaction by 18% during the optimization process. In addition, total monoselectivity was observed in all cases. This observation may rely on the sterically large *tert*-butyl groups on silicon that hinder the rotation of the silanol directing group, preventing the second alkenylation from occurring. Fourteen tetra- to hexapeptides underwent successful alkenylation with *tert*-butyl acrylate in 21–50% yield, showing complete *ortho*-selectivity to the silanol directing group and obtaining only the monoalkenylated products.

## 6.0 C(sp^2^)–H Bond Alkynylation

**Scheme 80:**
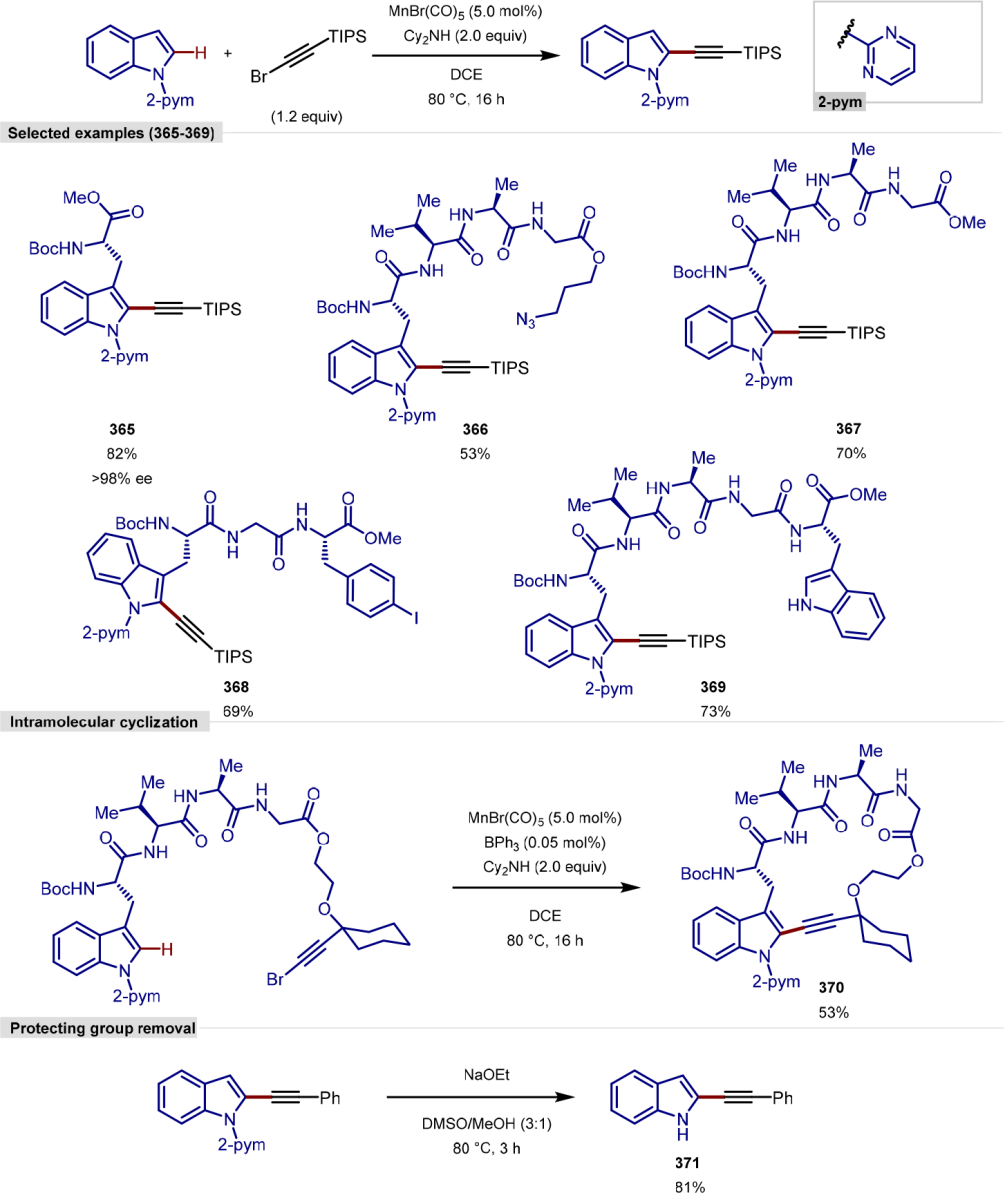
Manganese(I)-Catalyzed C(sp^2^)–H Alkynylation, Showing Selected Examples and an *N*-Deprotection

**Scheme 81:**
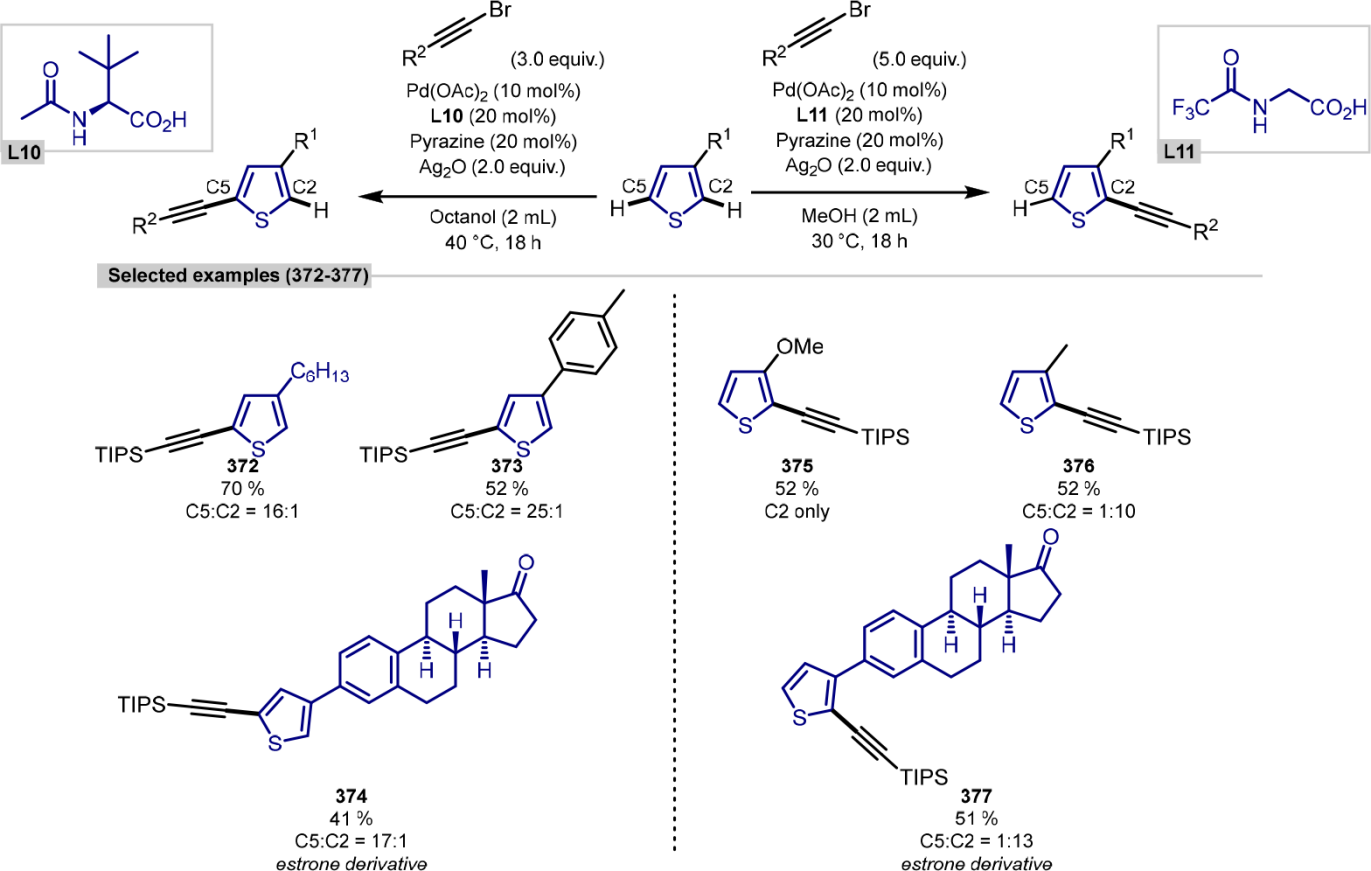
Palladium(II)-Catalyzed C(sp^2^)–H Alkynylation of Thiophenes, Showing Ligands Used to Achieve C-2 or C-5 Selectivity

**Scheme 82:**
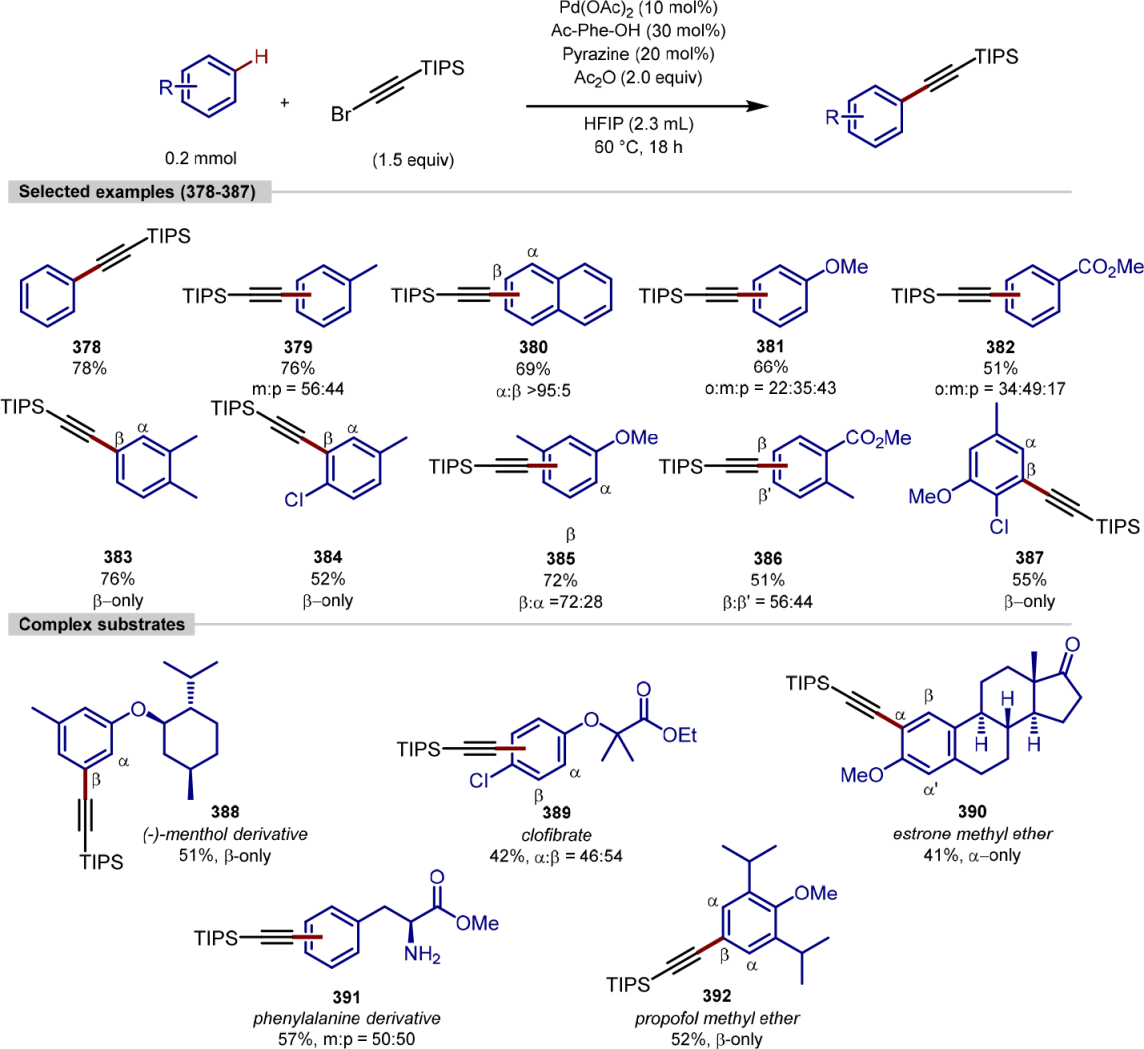
Nondirected Palladium(II)-Catalyzed Alkynylation of Arenes

In 2017, Ackermann reported the directing group-assisted C(sp^2^)–H alkynylation of tryptophan and its derivatives using [MnBr(CO)_5_] (Scheme 80).^253^ The method was initially developed using silyl-substituted haloalkynes (e.g., 1-bromo-2-(triisopropylsilyl)acetylene) with *N*-pyrimidyl-substituted indoles and pyrroles. Subsequently, it was discovered that the addition of cocatalytic BPh_3_ enabled the use of nonsilylated alkyl, alkenyl and aryl alkynes in high yields by accelerating β-elimination of the bromide. As part of mechanistic investigations, the use of D_2_O as a cosolvent led to hydrogen isotope exchange at the indole C-2 position. However, there was no change in the reaction rate when an isotopically enriched substrate was used (KIE = 1.0), suggestive of facile C–H activation. Kinetic studies showed the reaction was first-order in both starting materials and the catalyst. The method was applied to eight complex peptides and gave the alkynylated products **365**–**369** in moderate to good yields (53–82%) and importantly without any observed epimerization of stereocenters. A further example of macrocyclization was demonstrated by the construction of a 21-membered macrocycle **370** by intramolecular C–H alkynylation. It is also notable that the pyrimidyl-directing group necessary for this transformation could be removed in a traceless fashion using NaOEt in DMSO/MeOH (3:1).

van Gemmeren and Mondal disclosed a palladium-catalyzed regiodivergent C–H alkynylation of C-3 substituted thiophenes (Scheme 81).^254^ The aim was to enable regioselectivity for C-2 or C-5 functionalization regardless of the electronic properties of the C-3 substituent, which was achieved using careful choice of amino acid-derived ligands. Selectivity at the C-5 position was enabled by increasing steric demand of the α-substituent of the amino acid ligand, with the optimal *tert-*butoxyl group giving a C-5:C-2 ratio of 94:6 (compared to 47:53 in the absence of the ligand) during optimization. To achieve C-2 selectivity, *N*-substituted electron-withdrawing groups in the ligand were preferred, with the best results obtained using a COCF_3_ substituent, suggesting a stronger influence by electronic rather than steric effects for this selectivity. The C-5 scope included 15 thiophene examples giving 41–70% yields with C-5:C-2 ratios between 3:1 and 25:1, while the C-2 scope contained 14 thiophene examples giving 41–61% yields with C-5:C-2 ratios of 1:4 to 100% C-2 selectivity. In each case, the method was demonstrated using an estrone derivative, achieving 41% and 51% yields of C-5 and C-2 products, respectively.

van Gemmeren reported a directing group-free palladium-catalyzed C–H alkynylation of aromatics (Scheme 82).^255^ While poor to moderate regioselectivity was observed with monosubstituted aromatics, disubstituted and trisubstituted substrates showed significant regioselectivity for the least sterically hindered positions, giving complete regioselectivity in many cases. The conditions were applied to several examples of complex molecule C–H alkynylation, of which four were fully regioselective and all were obtained in respectable yields (40–57%) while the other examples gave mixed ratios of isomers. On probing the mechanism, a kinetic isotope effect was observed, indicating that the C–H activation was rate-determining, and the addition of cocatalytic pyrazine was deemed essential for improvements to both reaction rate and product yield. Based on experiments monitoring initial rates with and without pyrazine, it was proposed that this additive enables the formation of a more catalytically active species. The mild reaction temperature (60 °C) and conditions make this a versatile method for late-stage nondirected C–H alkynylation.

**Scheme 83:**
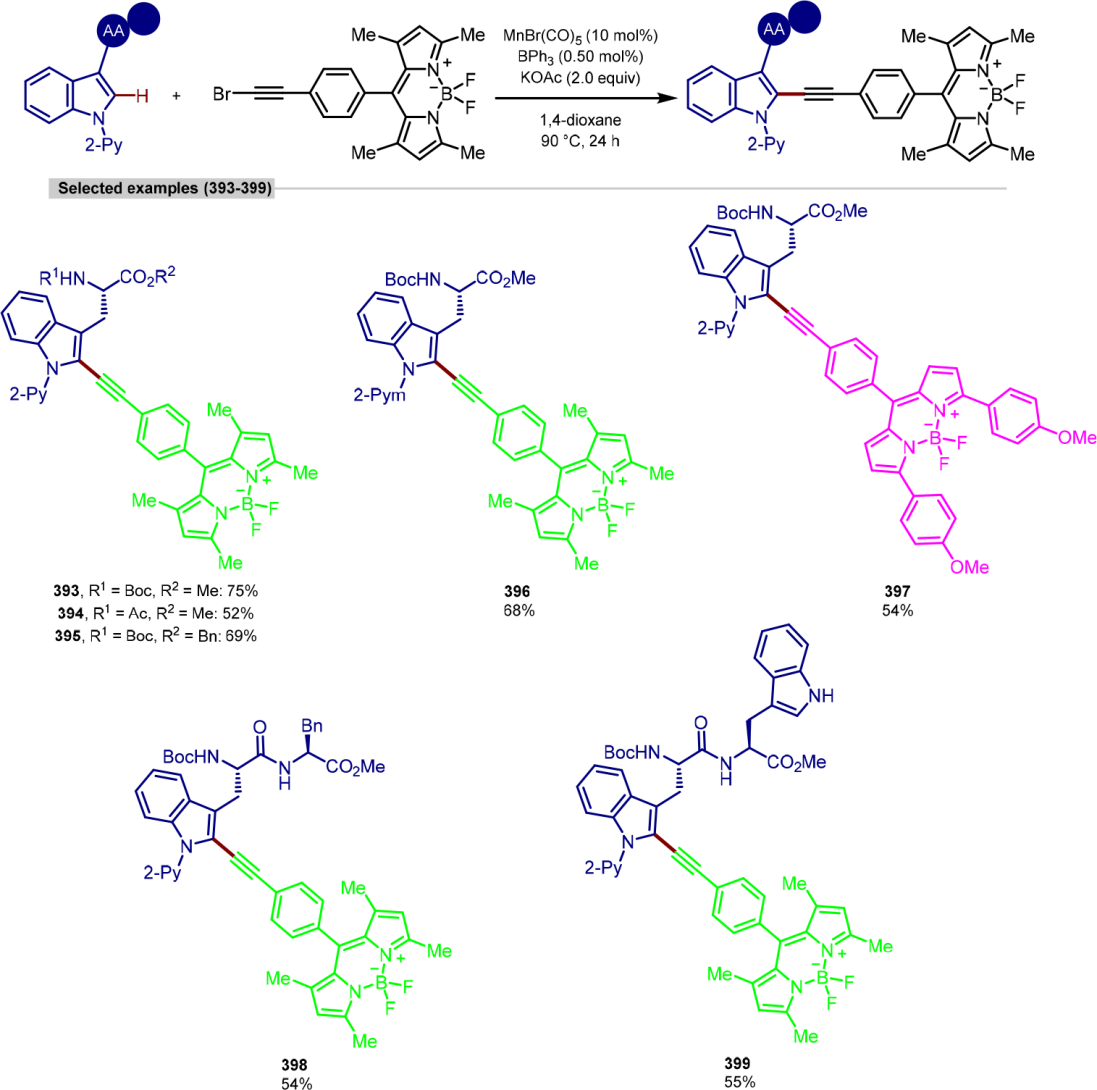
Fluorescent Labelling of Peptides by Manganese(I)-Catalyzed Alkynylation

Ackermann and Vendrell reported the fluorescent labeling of complex peptides via C(sp^2^)–H bond alkynylation using manganese(I) catalysis (Scheme 83),^249^ addressing the excessive typical dependence on more scarce transition-metal catalysts. The fluorophore coupling partner was a BODIPY derivative bearing a bromoalkyne, and a scope of 14 dipeptides carrying a tryptophan residue, modified with a 2-pyridyl directing group were alkynylated at the indole C-2 position to install the BODIPY moiety in moderate to good yields (50–75%). The addition of BPh_3_ was found essential for reactivity, which was proposed to be due to its Lewis acidity enabling β-bromide elimination. By modification of the BODIPY-alkyne, substituting the methyl groups at the pyrrole α-position for aryl groups, the emission wavelength could be extended.

### 6.1 C(sp^3^)–H Bond Alkynylation

**Scheme 84:**
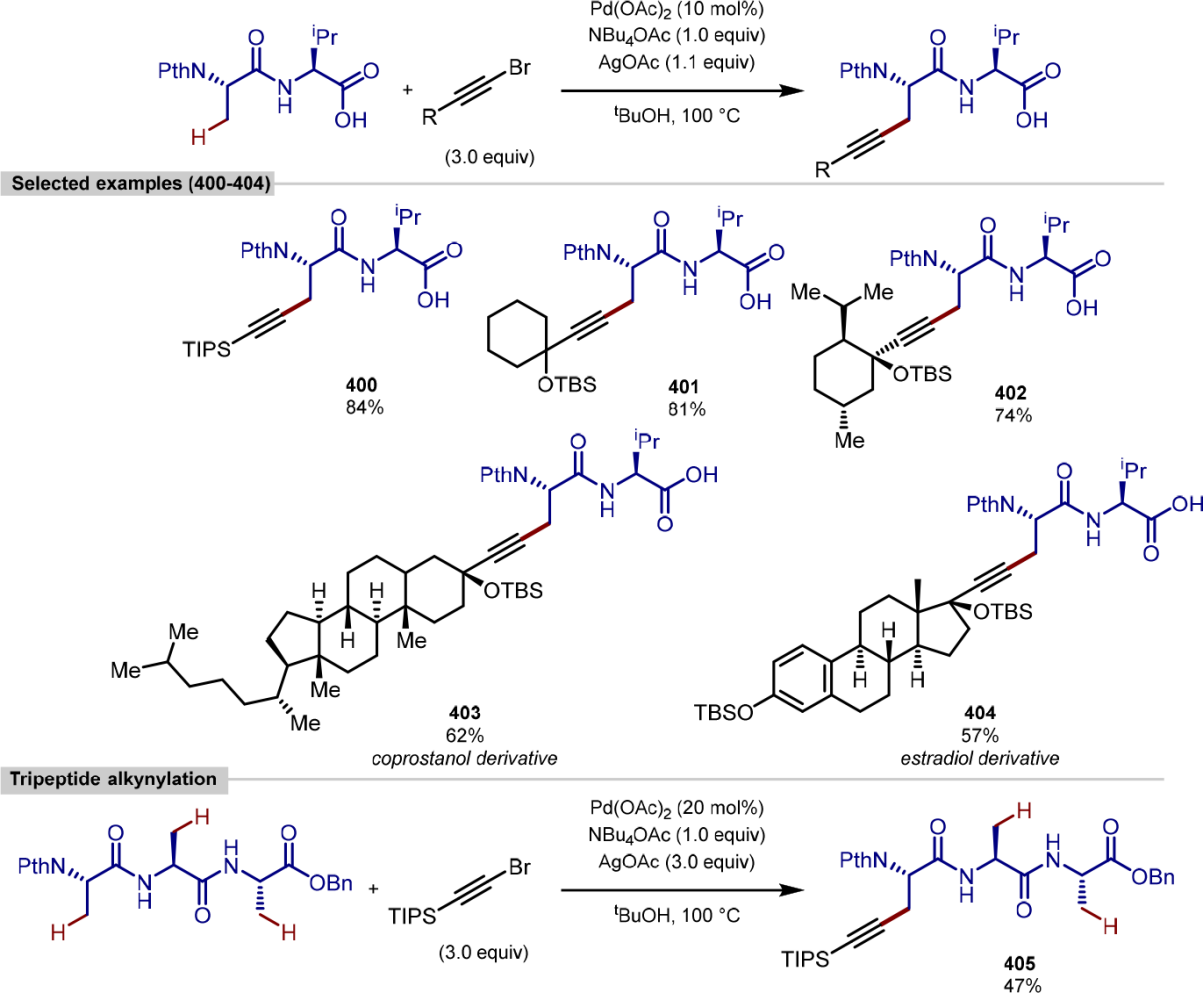
Palladium(II)-Catalyzed C(sp^3^)–H Alkynylation of Dipeptides and a Tripeptide

The first example of a C(sp^3^)–H alkynylation was described by Yu *via* a palladium-catalyzed reaction, which enabled a linchpin approach to the derivatization of the unactivated side chains of oligopeptides with alkyne coupling partners in good to excellent isolated yields (Scheme 84).^256^ A wide range of alkyne coupling partners were prepared from the corresponding ketones, including derivatives of coprostanol (which binds to overexpressed androgen receptors in prostate tumor cells)^257^ and estradiol (binds to overexpressed estrogen receptors in breast tumor cells).^258^ A scope of 16 alternative dipeptides also showed high yields (51–84%), including five *N-*methylated variants, all with tolerance for the free carboxylic acid at the *C*-terminus. The reaction of a tripeptide also gave 47% of alkynylated product **405** with regioselectivity for the *N*-terminus. While this reactivity is versatile, preparation of the bromoalkyne coupling partner requires four steps, which is potentially costly in time and resources.

**Scheme 85:**
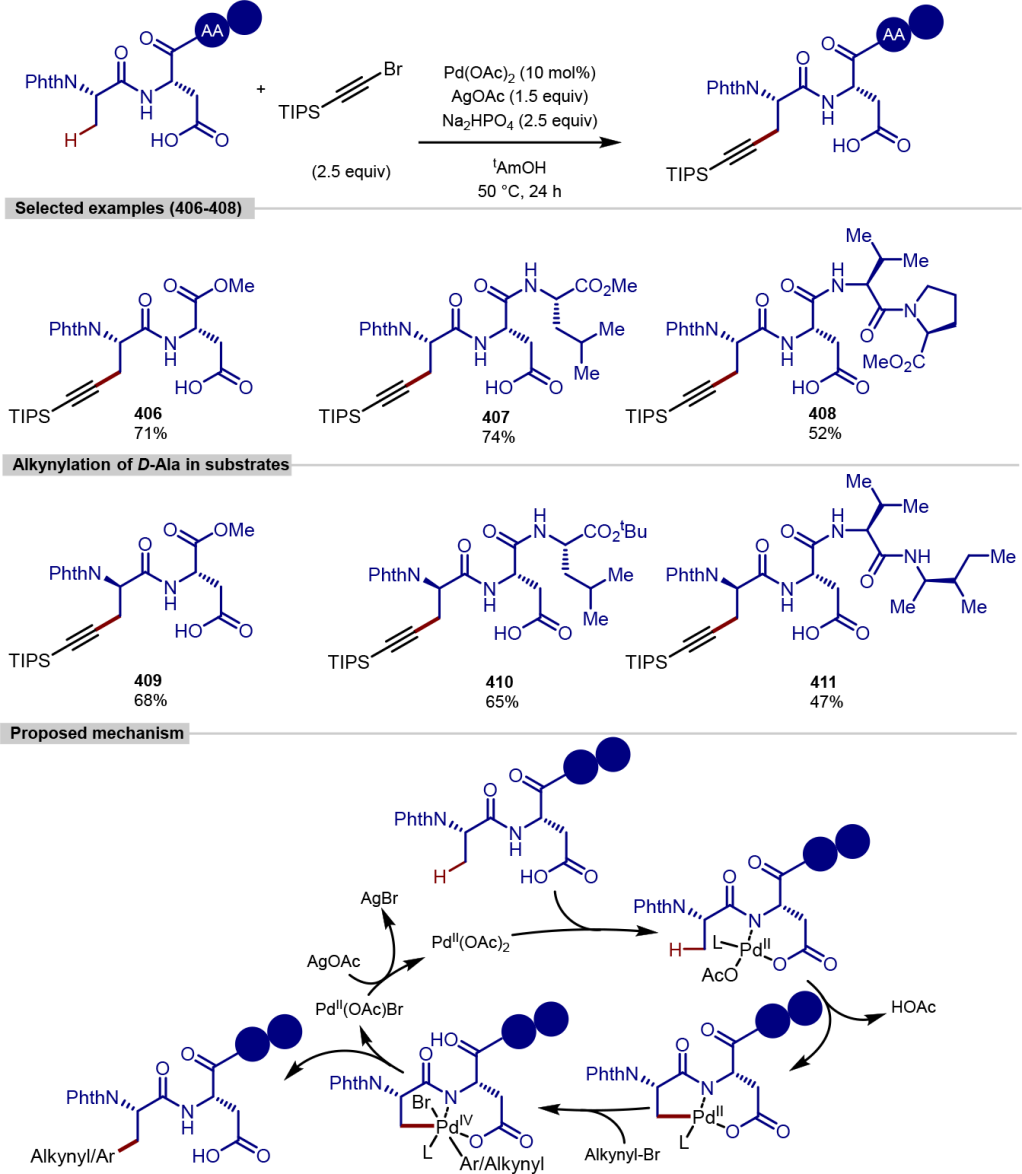
C(sp^3^)–H Alkynylation of Alanine Using Palladium-Catalysis and Alkynylbromides

Weng reported a palladium-catalyzed C(sp^3^)-H alkynylation of alanine (Ala) residues in peptides, taking advantage of an aspartic acid (Asp) as an endogenous directing group (Scheme 85).^259^ The reaction manifold was shown to be tolerant of di/tri/tetrapeptides giving yields up to 86% under mild conditions (50 °C) being equally compatible with both the *L*- and *D*-Ala epimers. Probing the reaction mechanism, a control experiment using the methyl ester of the Asp residue showed no successful reactivity, indicating the key role as directing group of the free carboxylic acid. The proposed mechanism proceeds *via* base-assisted C–H activation enabled by chelation from the Asp carboxyl group and backbone nitrogen atom. Oxidative addition of the coupling partner followed by reductive elimination gave the product and a palladium bromide species. Silver acetate was then proposed to facilitate ligand substitution by abstracting the bromide ligand, thus closing the catalytic cycle.

## 7.0 C–H Bond Carbonylation, Carboxylation, and Cyanation

Carboxylation and carbonylation reactions are potent methods for C–C bond formation and can facilitate atom economical functionalization of substrates by capture of CO_2_ and CO. Industrial applications include the carbonylation of methanol with CO to form acetic acid by the Monsanto and Cativa processes (utilized on a global scale), and the integration of transition-metal catalysis has enabled the incorporation of carbonylation reactions into various natural product syntheses.^260,261^ Similarly, CO_2_ is an abundant, nontoxic and low-cost C-1 reagent. These qualities make it valuable in chemical synthesis for the atom-economical formation of valuable structural motifs, including carboxylic acids, lactones, lactams, and carbonates, which are all commonly found in valuable natural products and drug molecules.^262,263^

**Scheme 86:**
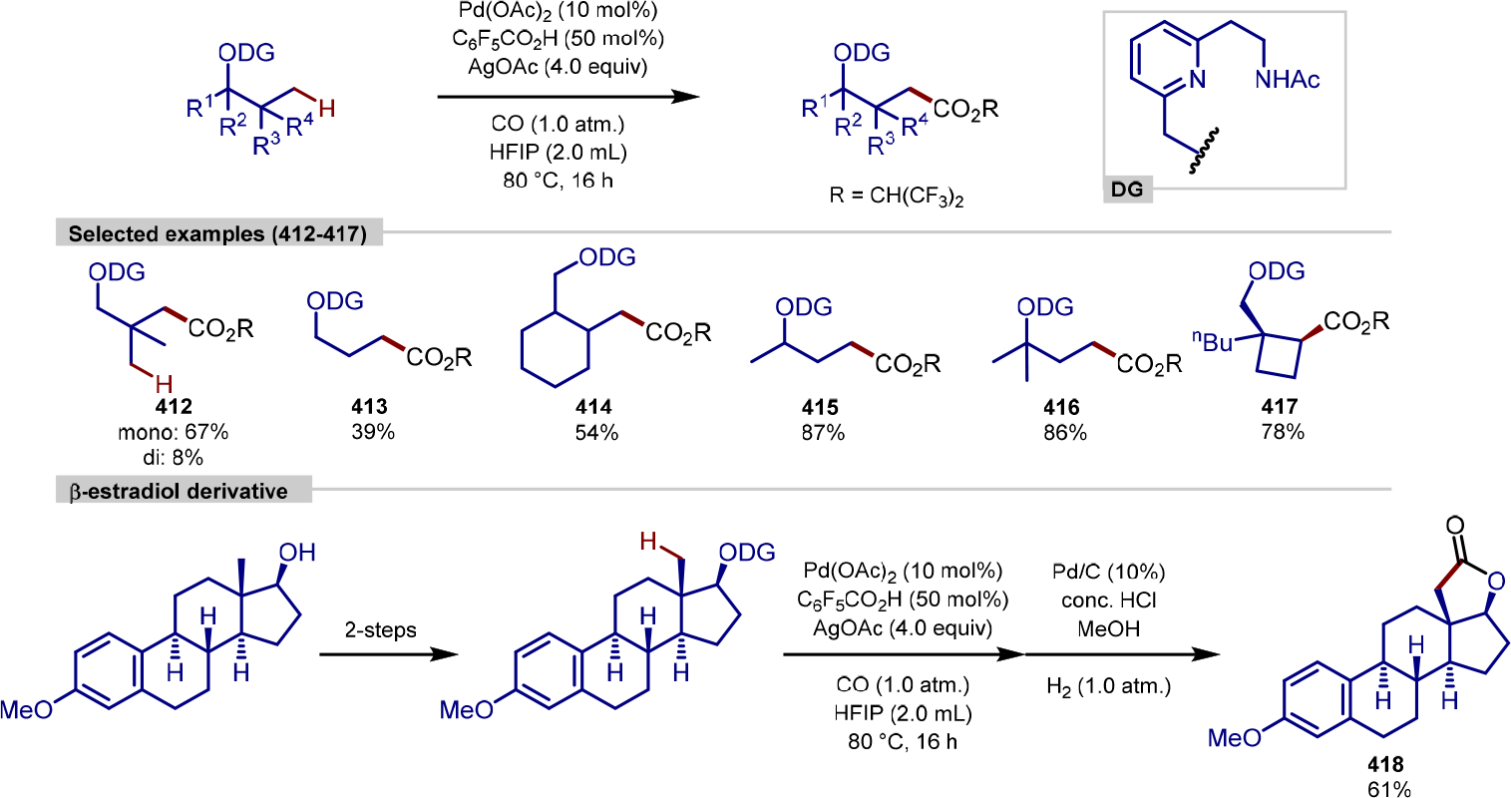
C(sp^3^)–H Carbonylation by Palladium(II)-Catalysis Using Ethereal Oxygen as an Intramolecular Directing Group

Yu detailed a route to achieving γ-selective C(sp^3^)–H carbonylation and olefination of labile directing-group bearing alcohols using Pd(OAc)_2_ as a catalyst in combination with perfluorobenzoic acid (Scheme 86).^263^ It was found that the reaction was enabled due to the weakly coordinating nature of the ethereal oxygen allowing binding of other reaction components (carbon monoxide and solvent), and the pyridine-containing directing group chosen could be readily removed. The scope of 23 examples included primary, secondary and tertiary alcohol derivatives that were obtained in moderate to good-isolated yields (39–87%) of the desired carbonylation product. The established reactivity was exemplified through use of a derivative of γ*-*estradiol with the directing group installed, undergoing γ-carbonylation and intramolecular cyclization upon subsequent removal of the directing group to give **418** in good yield. The mechanism proposed proceeds via directing-group assisted C–H activation followed by binding of carbon monoxide (displacing the ethereal oxygen). Migratory insertion with binding of a molecule of solvent (HFIP) then forms the new C–C bond, before reductive elimination forms the product and palladium(0), which is reoxidized by silver(I) to close the cycle forming palladium(II). Overall, this process represents a novel method for γ-selective C(sp^3^)–H activation with applicability to complex molecules functionalization due to the facile directing group removal by hydrogenation.

**Scheme 87:**
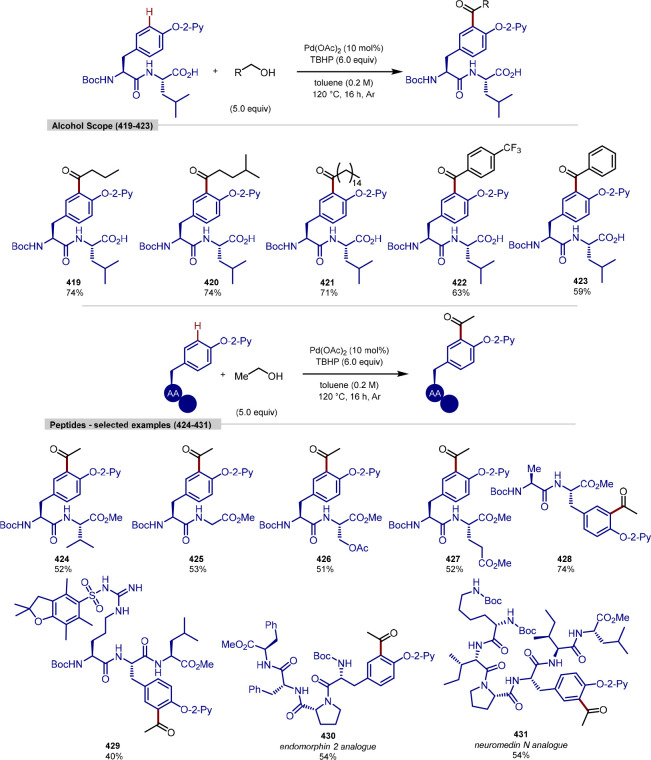
Palladium-(II)-Catalyzed *ortho*-C–H Carbonylation of Tyrosine-Containing Oligopeptides with Alcohols

A method for the *ortho*-selective acylation of tyrosine (Tyr)-containing oligopeptides with alcohols was described by Correa using palladium-catalysis (Scheme 87).^264^ Peptide acetylation *via* Friedel–Crafts methods is generally unsuitable due to the tendency for racemization to occur and the need for stoichiometric amounts of AlCl_3_. The method used benign ethanol as an acetyl source, instead of the more hazardous acetyl chloride, meaning this process offers numerous advantages. To enable selectivity, however, this technique requires a 2-pyridyl ether as a directing group. The *ortho*-acetylation occurs with no bisacetylation or alkoxylation side reactions, and the scope was expanded to include five other aliphatic alcohols, as well as 19 Tyr-containing compounds with yields ranging from 37 to 74%, including two biologically relevant examples. The viability of the process on preparative scale was also demonstrated with two gram-scale syntheses using ethanol and *n*-butanol gave 62 and 74%, respectively. This manifold represents a notable advancement in classical Friedel–Crafts chemistry, offering monoselectivity, regioselectivity, scalability, and a broader substrate scope.

**Scheme 88:**
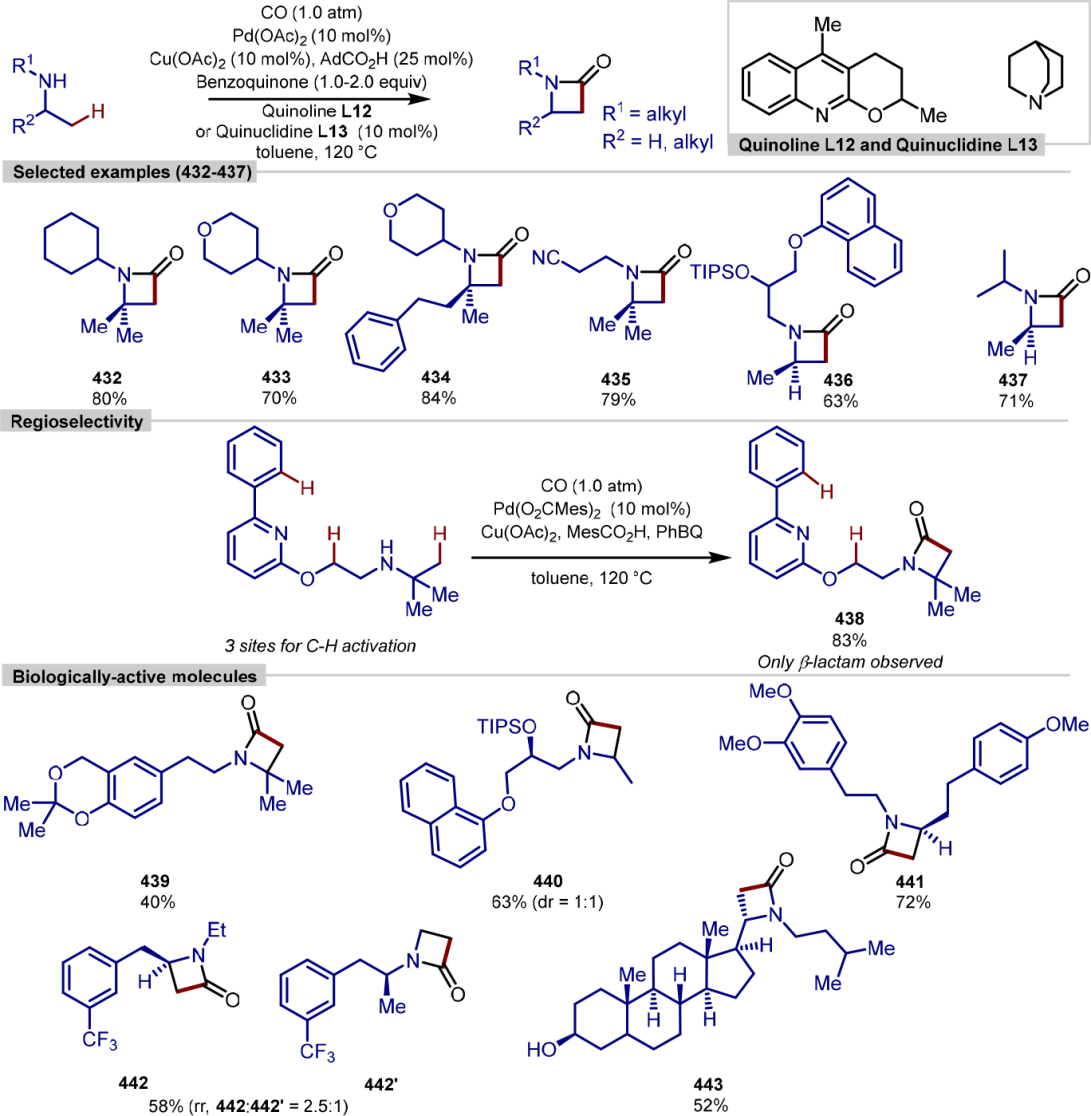
C(sp^3^)–H Carbonylation and Cyclization of Aliphatic Amines, Showing Regioselectivity of the Reaction and Modification of Biologically Active Molecules; R^1^, R^2^ = Alkyl

A method for the palladium-catalyzed carbonylation of aliphatic amines using carbon monoxide was described by Gaunt (Scheme 88).^265^ Success in the transformation was dependent on the use of sterically hindered carboxylate ligands allowed carbonylation of aliphatic amines without protection, enabling late-stage functionalization of pharmaceutically relevant compounds. Initial experiments resulted in the formation of CO_2_ and reduction of the palladium catalyst forming acetyl anhydride, which led to poor reactivity. However, increasing the steric demand of the carboxylic acid (to AdCO_2_H) led to preferential formation of a carbamoyl-palladium species, leading to the desired C–H activation. A broad scope of 42 amines were tested, giving yields up to 90%, and five examples of biologically active substrates were derivatized in 40–72% yields. The regioselectivity of the reaction was also exemplified using a substrate containing three possible sites for C–H activation that gave only β-lactam **438** in 83% yield. The utility of the β-lactam motif was also demonstrated in four derivatization experiments, including protecting group removal, reductive ring-opening, esterification, and reduction to the corresponding azetidine.

**Scheme 89:**
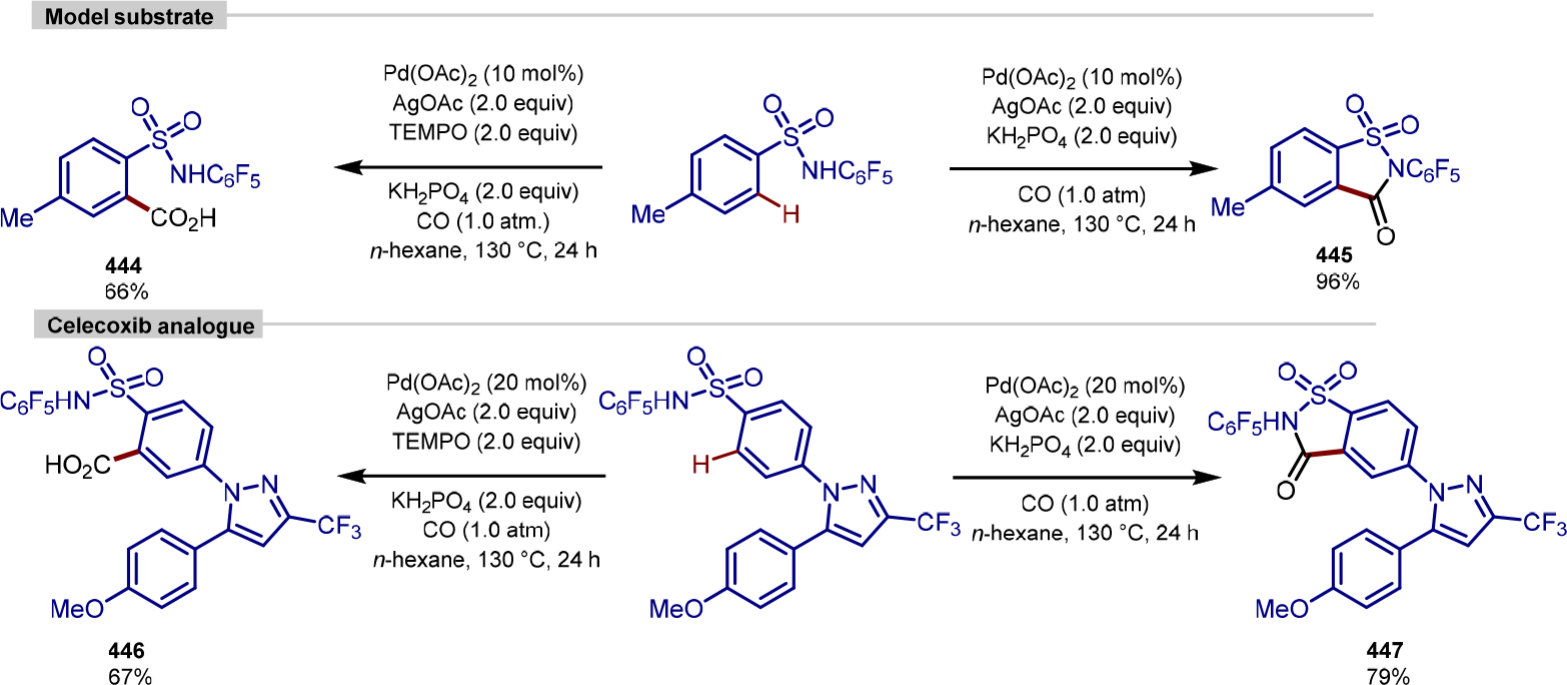
Palladium(II)-Catalyzed Carboxylation and Carbonylation of a Substrate Arene and Celecoxib Analogue *via* Two Reaction Pathways

Examples of arene C(sp^2^)–H carbonylation and carboxylation were demonstrated by Yu in the synthesis of pharmaceutical analogues, with emphasis on addressing the challenge of regioselectivity in substrates with more than one viable C–H bond (Scheme 89).^266^ In this context, the authors describe a palladium-catalyzed C–H activation, enabling six different reaction pathways, including carboxylation and carbonylation. To develop the reaction manifold, carbonylation and carboxylation conditions were applied inspired by previously described methods, using Pd(OAc)_2_ as a catalyst and CO as a carbonyl source achieving *ortho-*carboxylation and carbonylation in 66% and 96% yields, respectively, on a model substrate.^267,268^ The functionalization was demonstrated by extending these reactions to an analogue of COX-2 inhibitor, celecoxib. The substrate was amenable to all six catalytic reactions with selectivity for the sulfonamide-directed C–H activation, despite the potential for C–H activation by the pyrazole group, achieving 67% and 79% yields in carboxylation and carbonylation, respectively. This process demonstrates the potency of sulfonamides as directing groups for site-selective C–H activation, broadening the scope of methods for drug derivatization.

**Scheme 90:**
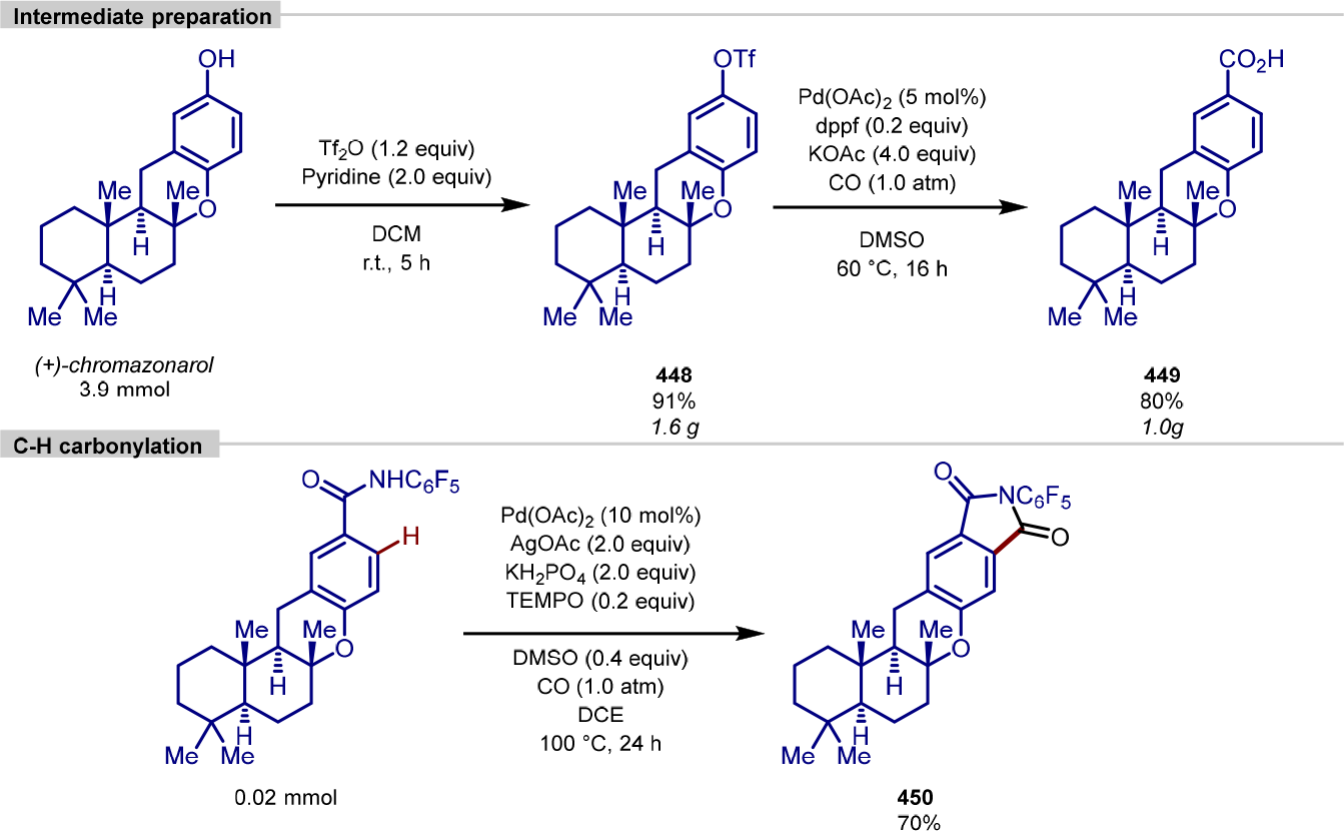
Palladium(II)-Catalyzed C(sp^2^)–H Carbonylation of an Intermediate Substrate Derived from (+)-Chromazonarol

Yu and Baran reported a method to synthesize (+)-hongoquercin A *via* two C–H functionalization’s of a key intermediate synthesized by carbonylation on gram-scale from (+)-chromazonarol (itself prepared in six steps from (+)-sclareolide, Scheme 90).^269^ The carboxylic intermediate was prepared by the formation of the aryl triflate **448** followed by palladium-catalyzed hydroxycarbonylation, and was used in seven different C–H functionalization reactions (alkylation, lactonization, oxidation, vinylation, amination, arylation, and carboxylation), to demonstrate the versatility of C–H functionalization in the preparation of valuable analogues. In the carbonylation process, the corresponding amide bearing electron-withdrawing pentafluorobenzene was used as the substrate (offering greater reactivity), and the corresponding phthalimide **450** was obtained in 70% yield. It was also noted that the presence of TEMPO was essential for the carbonylation to proceed, giving only a return of the amide in its absence.

Nitriles represent important building blocks to the synthetic chemist that can be readily converted to a range of different functional groups including, but not limited to, amides, amines, carboxylic acids, and tetrazoles. They are widely present in a number of important dyes, agrochemicals, pharmaceuticals, and natural products, making cyanation an advantageous transformation for the synthetic community.

**Scheme 91:**
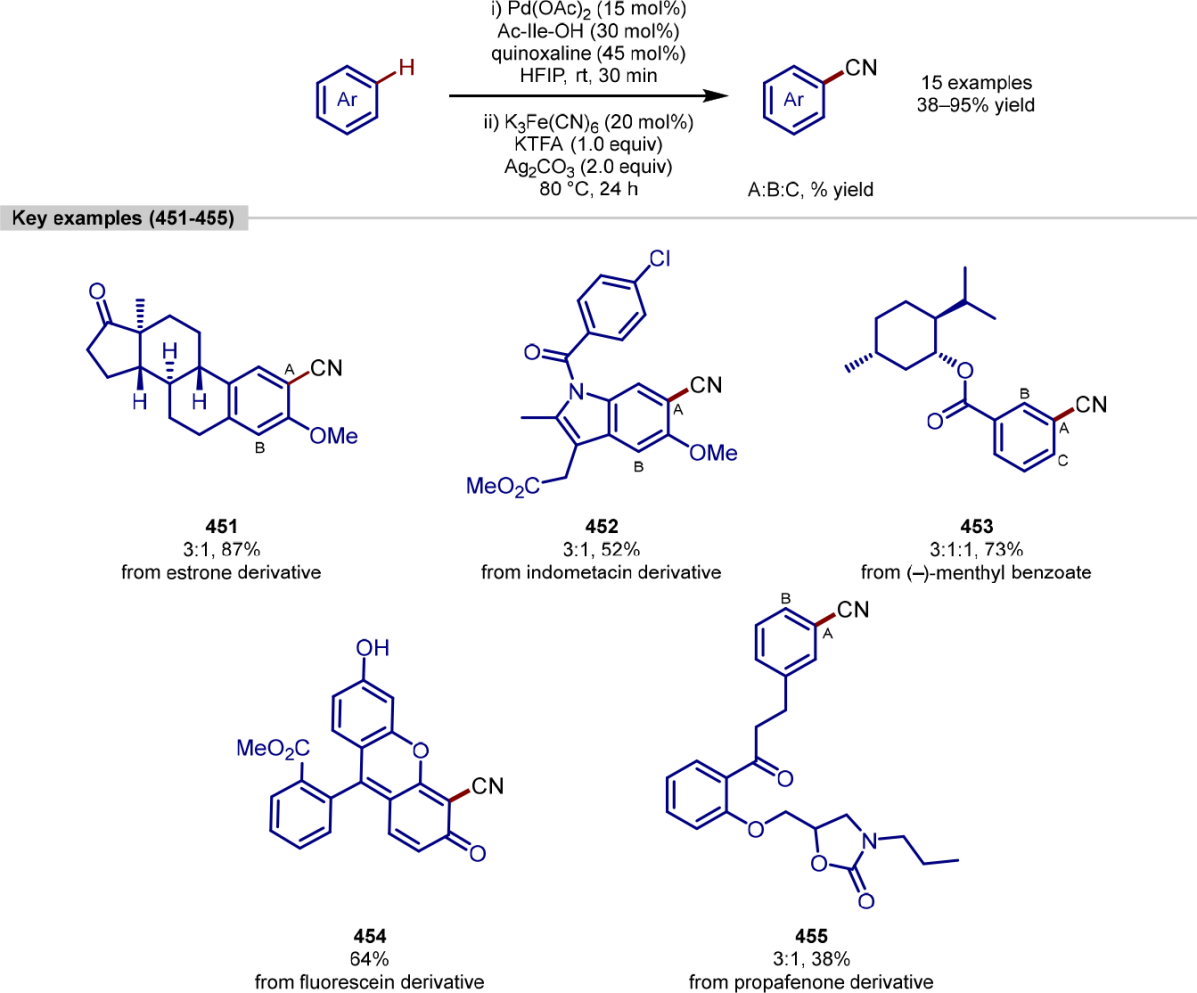
Ritter’s Palladium-Catalyzed Cyanation of C(sp^2^)–H Bonds Using K_3_Fe(CN)_6_ as a Cyanide Source

The first broadly applicable cyanation of complex molecules was reported by Ritter in 2019 using Pd(OAc)_2_, commercially available ligands, and a safe cyanating reagent K_3_Fe(CN)_6_ (Scheme 91).^270^ The methodology was widely applicable tolerating several sensitive functional groups such as free hydroxyl groups, ketones, and sulfonamides showing compatibility with a number of dyes and bioactive molecules with 15 examples of modification of complex substrates reported in 38–95% yield. This work represents the first example of directing-group-free cyanation compatible with a range of electron-rich and electron-deficient arenes. Catalytic cyanation has previously represented a challenging transformation due to the tendency of common cyanating reagents to form the free cyanide anion that can inhibit catalytic activity and prevent reactivity. This procedure makes use of a dual ligand system that is proposed to prevent this from occurring. The first ligand, an amino acid, forms a stable and reactive palladium center while the second ligand, quinoxaline, prevents the cyano-group binding without hindering the electrophilicity of the palladium center that is required for metalation to occur. Both Cu^II^ and Ag^I^ oxidants were compatible with the reaction conditions with Ag_2_CO_3_ used for each of the examples. The procedure gave a mixture of regioisomers, however, it is worth noting that these could be separated to give analytically pure product in each case. While poor regioselectivity is generally not desirable in such transformations, this could be found useful for diversification as it can rapidly produce a number of analogues to be tested rather than having to make each of these through longer *de novo* processes.

## 8.0 Conclusion and Summary

Overall, the discussed state-of-the-art examples highlight the possibility of using new synthetic transition-metal-catalyzed methods for the direct functionalization of a broad range of C–H bonds with high levels of both chemo- and regio-selectivity. The utility of these synthetic inventions highlights the importance for the future continued discovery and development of generic methods for the diversification of complex molecules. Considering the high levels of reactivity observed for many methods toward carbon–carbon bond formation, these precedents highlight the ability to use complex molecules as a proving ground for new reactivity and new transition metal-catalyzed reactions to highlight their broad applicability.

Powerful synergies between emerging artificial intelligence and late-stage functionalization methods may allow for expedited identification of biologically relevant structures. For example, the predictive utility of artificial intelligence may facilitate identification of synthetic methods applicable to core structures of interest, and LSF methods will then enable the targeted diversification of these structures. Similarly, the broad tolerance and generalizability of many methods applicable to LSF allows for the rapid generation of libraries of new compounds. Therefore, one approach where LSF is highly valuable is within combinatorically generated libraries, whereby a core structure can be simultaneously diversified by many reaction partners (e.g., encoded libraries).

A further high utility application of LSF lies within bioconjugation, whereby selective functionalization allows for the incorporation of biologically active molecules to biomolecules, such as proteins or antibodies. The resulting biomolecule-drug conjugates have potential use within therapeutic or diagnostic applications. In this area, LSF has already shown promise for attaching fluorescent tags, peptidic chains, or targeting groups to complex molecules that could potentially serve as probes within imaging or drug delivery. Further advances here would be illustrated using LSF to generate analogues of a biologically active molecule already bound at the requisite antibody or protein. The emergence of precision medicine is a potential area that LSF can directly impact. Within this area of medicinal discovery, an approved drug for a therapeutic target could be tailored in a late-stage manner to meet the genetic requirements of individual patients, thereby increasing efficacy and reducing the frequency of side-effects. We anticipate that the continued growth and diversity of possible transformations will enable the discovery of new biological applications and streamline efficiency within many areas of the scientific community.

## Abbreviations

AA: Amino acid
Ac: Acyl
acac: Acetylacetonate
Ad: Adamantyl
AMCB: Aminomethylcyclobutane
AMCP: Aminomethylcyclopropane
AQ: 8-Aminoquinoline
Ar: Aryl
Aux: Auxiliary
Bn: Benzyl
Boc: *tert*-Butyloxycarbonyl
BODIPY: Dipyrrometheneboron difluoride
BPCP: 1-(Biphenyl)-2,2-diphenylcyclopropanecarboxylate
bpy: 2,2′-Bipyridine
Bq: Benzoquinone
BTPCP: 1-(4-Bromophenyl)-2,2-diphenylcyclopropanecarboxylate
Cbz: Benzyloxycarbonyl
COD: 1,5-Cyclooctadiene
coe: Cyclooctene
Cp: Cyclopentadienyl
Cp*: Pentamethylcyclopentadienyl
Cy: Cyclohexyl
DABCO: 1,4-Diazabicyclo[2.2.2]octane
DAST: Diethylaminosulfur trifluoride
DCE: 1,2-Dichloroethane
DCM: Dichloromethane
dcype: 1,2-Bis(dicyclohexylphosphino)ethane
DG: Directing group
DIPEA: *N*,*N*-diisopropylethylamine
DMA: Dimethylacetamide
DMAP: 4-Dimethylaminopyridine
DME: Dimethoxyethane
DMF: *N*,*N-*Dimethylformamide
DMSO: Dimethylsulfoxide
DOSP: 1-[(4-Dodecylphenyl)sulfonyl]proline
dppf: 1,1′-Bis(diphenylphosphino)ferrocene
dppp: 1,3-Bis(diphenylphosphino)propane
*dr*: Diastereomeric ratio
EDG: Electron-donating group
equiv: Equivalents
EWG: Electron-withdrawing group
GABA: γ-Aaminobutyric acid
HAT: Hydrogen atom transfer
HATU: Hexafluorophosphate azabenzotriazole tetramethyl uronium
HFIP: 1,1,1,3,3,3-Hexafluoroisopropan-2-ol
HPLC: High-performance liquid chromatography
^*i*^Bu: *iso*-Butyl
^*i*^Pr: *iso-P*ropyl
KIE: Kinetic isotope effect
L: Ligand
LG: Leaving group
LSF: Late-stage functionalization
Mes: Mesityl
MQ: 5-Methoxy-8-aminoquinoline
NBE: Norbornene
NHC: *N*-Heterocyclic carbene
NHP: *N*-Hydroxyphthalimide
NMP: *N*-Methyl-2-pyrrolidone
NMR: Nuclear magnetic resonance
NOESY: Nuclear Overhauser effect spectroscopy
Oct: Octanoate
PA: Picolinamide
PBA: Phenyl benzoic acid
PBS: Phosphate-buffered saline
Phth: Phthalimide
Piv: Pivaloyl
ppy: 2-Phenylpyridine
PTAD: (1-Adamantyl)-(*N*-phthalimido)acetato
Py: Pyridyl
RP-HPLC: Reversed-phase high-performance liquid chromatography
SET: Single electron transfer
SPPS: Solid-phase peptide synthesis
TAM: Triazolydimethylmethyl
^*t*^Am: *tert*-Amyl
TBDMS: *tert*-Butyldimethylsilyl
TBME: *tert*-Butyl methyl ether
^*t*^Bu: *tert*-Butyl
TEMPO: 2,2,6,6-Tetramethylpiperidine 1-oxyl
Tf: Triflyl
TFA: Trifluoroacetate
THF: Tetrahydrofuran
TIPS: Triisopropylsilyl
TMS: Trimethylsilyl
TON: Turnover number
TPA: Triphenylacetate
